# Design, Synthesis, and Antifungal Activity of 3-Substituted-2(*5H*)-Oxaboroles

**DOI:** 10.1021/acsmedchemlett.3c00463

**Published:** 2024-02-22

**Authors:** Rose Campbell, Nicklas W. Buchbinder, Connor Szwetkowski, Yumeng Zhu, Karla Piedl, Mindy Truong, John B. Matson, Webster L. Santos, Emily Mevers

**Affiliations:** † Department of Chemistry, Virginia Tech, Blacksburg, Virginia 24061, United States

**Keywords:** Oxaboroles, tavaborole, MIC, antifungal, antibacterial

## Abstract

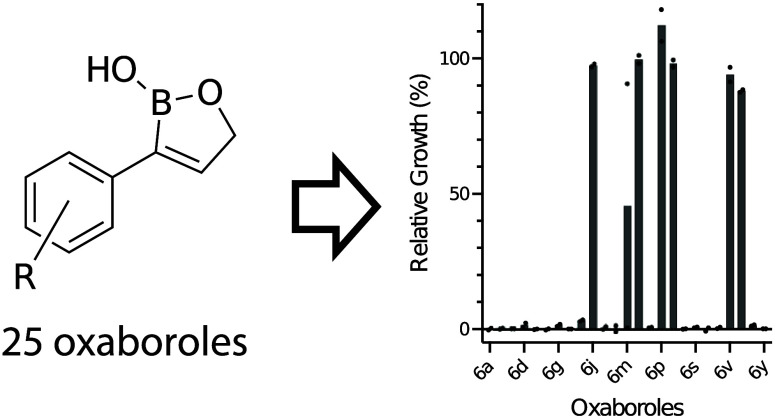

Next generation antimicrobial therapeutics are desperately needed as new pathogens with multiple resistance mechanisms continually emerge. Two oxaboroles, tavaborole and crisaborole, were recently approved as topical treatments for onychomycosis and atopic dermatitis, respectively, warranting further studies into this privileged structural class. Herein, we report the antimicrobial properties of 3-substituted-2(*5H*)-oxaboroles, an unstudied family of medicinally relevant oxaboroles. Our results revealed minimum inhibitory concentrations as low as 6.25 and 5.20 μg/mL against fungal (e.g., *Penicillium chrysogenum*) and yeast (*Saccharomyces cerevisiae*) pathogens, respectively. These oxaboroles were nonhemolytic and nontoxic to rat myoblast cells (H9c2). Structure–activity relationship studies suggest that planarity is important for antimicrobial activity, possibly due to the effects of extended conjugation between the oxaborole and benzene rings.

Over the past 70 years, only four classes of antifungal drugs have received FDA approval for the treatment of systemic infections: azoles,^1^ allylamines,^2^ polyenes,^3^ and echinocandins.^4,5^ Today’s treatments depend on new generations of these established classes; however, they have been unable to overcome the challenges posed by new, highly drug resistant pathogens.^6^ In particular, fungal infections represent an area where new treatments are desperately needed. Existing antifungals provide limited efficacy against *Candida auris*, *C. albicans*, *Cryptococcus neoformans*, and *Aspergillus fumigatus*, all of which are classified as critical pathogens by the World Health Organization.^7^ Roughly 1.7 million deaths per year worldwide are a result of fungal infections with immunocompromised patients being most at risk.^8^ In addition, most antifungal drugs have marked toxicity, which is incompatible with the long treatment regimen and increasingly higher doses that are required to combat infections caused by these multidrug resistant pathogens.^9^

In 2014, tavaborole (Kerydin, **1**), a benzoxaborole, was approved by the FDA for the topical treatment of onychomycosis (i.e., toenail fungal infections).^10,11^ Although it has only been approved to treat topical infections, there are four other boronic acid-containing drugs approved for the treatment of various diseases, including systemic diseases such as atopic dermatitis [crisaborole (Eucrisa), **2**],^12^ a β-lactamase inhibitor [vaborbactam (Vabomere), **3**]^13^ and multiple myeloma [bortezomib (Velcade), **4**, and ixazomib (Ninlaro), **5**],^14,15^ and with many more in various stages of clinical trials (Figure 1).^16^ Tavaborole and crisaborole are the only clinically approved benzoxaboroles, and both represent first-in-class drugs. Tavaborole inhibits tRNA-synthetase by trapping tRNA in the editing site,^17^ and crisaborole is a potent phosphodiesterase-4 (PDE4) inhibitor, which increases intracellular cyclic adenosine monophosphate (cAMP) levels, reducing inflammatory mediators.^12^ Structure–activity relationship (SAR) studies on tavaborole have shown that the boron is essential for activity, and crystal structures of tavaborole bound to tRNA synthetase reveal critical interactions between the boron’s unoccupied p-orbital and oxygen atoms (2′ and 3′) of the tRNA’s 3-terminal adenosine.^17^ Mutations that are predicted to destabilize this interaction (D487G or D487N) abolish the activity of tavaborole, confirming that this interaction is essential for the observed antifungal activity.^18^

**Figure 1:**
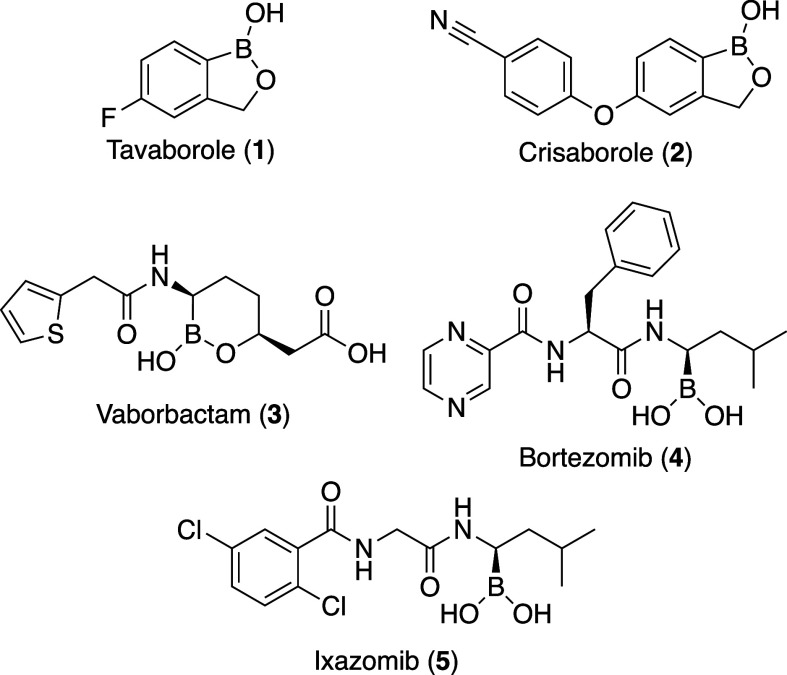
FDA approved boronic acid-containing drugs.

Although benzoxaboroles have been intensely studied for their potential to treat various diseases,^19−21^ no studies have focused on a related structural class such as 3-substituted-2(*5H*)-oxaboroles. Recent methodology works by our group and others have led to a tractable synthetic route to investigate the activity of this subclass of oxaboroles against a variety of fungal and bacterial pathogens.^22,23^ We hypothesize that the conformational freedom introduced from the bond rotation around the aryl and oxaborole rings could present structural features distinct from the benzoxaboroles that could be advantageous against pathogenic targets. In particular, this rotational freedom will provide another conformation to present the electrophile to a Lewis base. Herein, we report the synthesis and antimicrobial evaluation of oxaborole analogs. Our studies revealed that they have selective antifungal activity, exhibiting minimum inhibitory concentrations (MICs) as low as 5.20 μg/mL with no cytotoxicity against rat myoblast cells (H9c2) or hemolytic activity.

The synthesis of 3-substituted*-*2(*5H*)-oxaboroles (**6a**–**6y**) is shown in Scheme 1. Treatment of acetylenes (**7a**–**7j**, **7n**, **7p**–**7q**, and **7s**–**7y**) with *n*-butyllithium followed by methyl chloroformate afforded the corresponding alkynoates (**9a**–**9j**, **9n**, **9p**–**9q**, and **9s**–**9y**) in modest to excellent yields (42–92%, Figures S1–S5).^24^ Alternatively, Sonogashira cross coupling using PdCl_2_(Ph_3_)_2_, methylpropriolate, and a variety of aryl iodides produced alkynoates **9k**–**9m**, **9o**, and **9r** in 32–50% yields.^25^ A stereoselective *trans* hydroboration using pinacolborane and catalytic tributylphosphine gave the corresponding (*E*)-3-boryl-acrylates (**10a**–**10y**) (32–89% yields, Figures S6–S21). Reduction of methyl esters **10a**–**10y** with sodium borohydride yielded the desired 3-substituted-2(*5H*)-oxaboroles **6a**–**6y** in 19–78% yields (Figures S22–S126).^23^

**Scheme 1:**
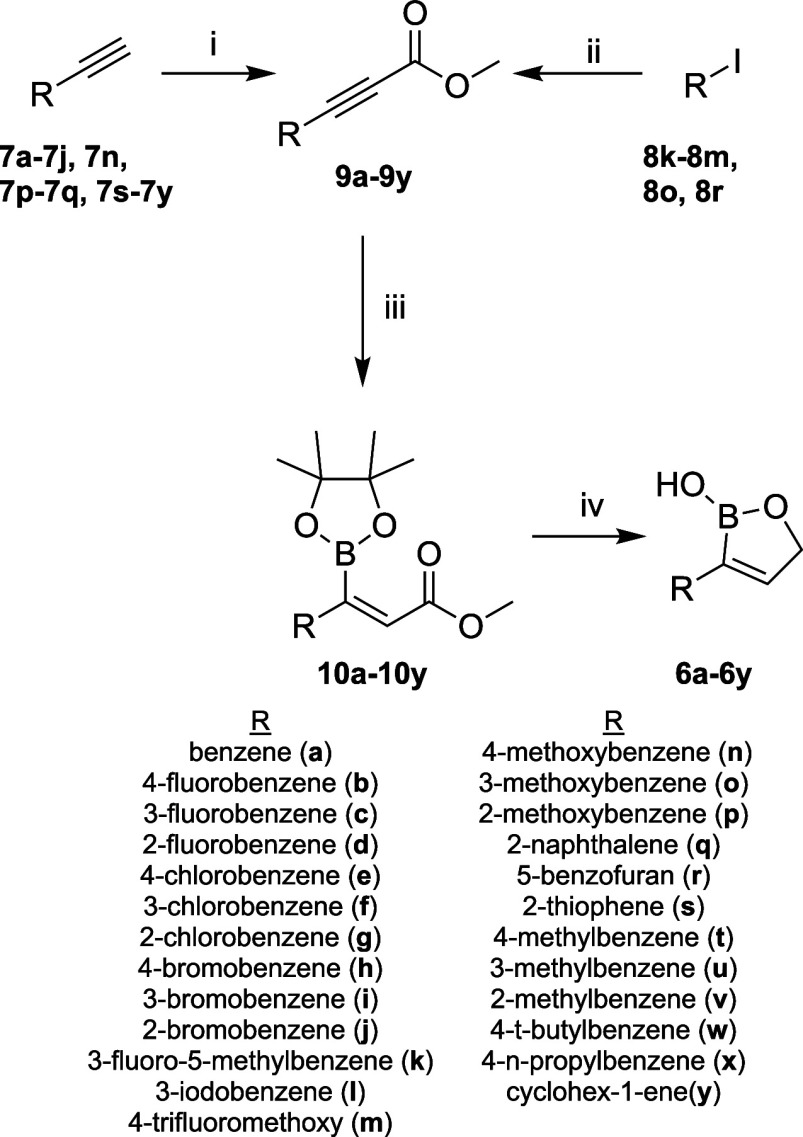
Synthesis of Derivatives **6a**–**6y** Reagents and conditions: (i) 2.5 M *n*-BuLi in hexanes (1.1 equiv), THF, −78 °C, 1 h then methyl chloroformate (1.1 equiv), THF, −78 °C, 1 h, 42–98%; (ii) PdCl_2_(Ph_3_)_2_ (5 mol %), CuI (10 mol %), TEA (3.0 equiv), methyl propiolate (1.2 equiv), THF, 70 °C, 16 h 32–50%; (iii) PBu_3_ (30 mol %), HBpin (1.2 equiv), neat, 25 °C, 1–3 h, 32–89%; (iv) NaBH_4_ (2.0 equiv), EtOH, 25 °C, 30 min, 19–78%.

With the oxaboroles in hand, a screen for growth inhibition at two concentrations (50 and 12.5 μg/mL) in liquid medium against nonspore forming pathogens was performed (Figures 2A and S127–S132). These included four bacterial pathogens [*Bacillus cereus* (Gram-positive), Methicillin resistant *Staphylococcus aureus* (Gram-positive), *Pseudomonas aeruginosa* (Gram-negative), and *Escherichia coli* (Gram-negative)], a fungal pathogen (*Candida albicans*), and a yeast (*Saccharomyces cerevisiae*). In general, these compounds exhibited significant growth inhibition against the fungal and yeast strains and were nearly completely inactive against the bacterial pathogens at the highest concentration evaluated (50 μg/mL) except for some moderate growth inhibition against *E. coli* (Figures S133–S151). Compounds exhibiting >95% growth inhibition at 50 μg/mL were then evaluated in a dose-dependent manner to determine MICs. Commercially available tavaborole, cycloheximide, and nystatin were also screened as benchmarks. Twenty-one compounds (**6a**–**6i**, **6k**–**6m**, **6o**, **6q**–**6u**, and **6w**–**6y**) exhibited growth inhibition profiles against the fungal and yeast pathogens and seven compounds (**6b**, **6e**, **6h**, **6s**, **6t**, and **6x**) inhibited the growth of *E. coli* and were prioritized for determination of MICs. In addition, the 3-substituted oxaboroles were screened in a dose-dependent manner on solid media against three spore-forming fungal pathogens: *Trichophyton mentagrophytes*, *Penicillium chrysogenum*, and *Aspergillus flavus* (Figures 2B and S152–S192). The compounds could not be screened for growth inhibition in liquid media against these pathogens due to clumping of the mycelia, which impeded the absorbance readings.

**Figure 2:**
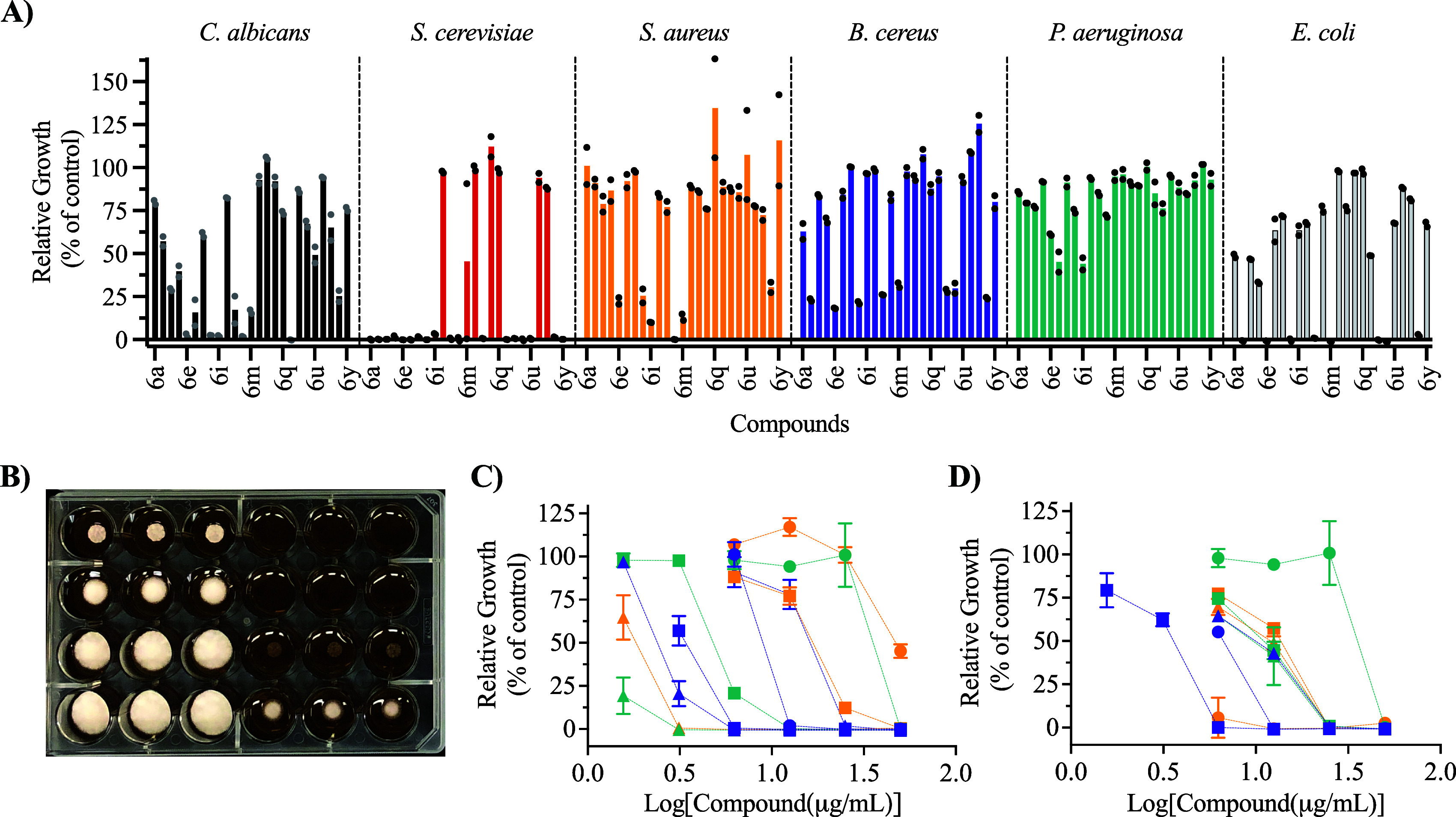
Biological data of the synthesized 3-substituted-2(*5H*)-oxaboroles. (A) Growth inhibition screen against nonspore forming microorganisms at 50 μg/mL, (B) an example of the solid MIC assay agar plates. Compounds **6v** (left three columns) and **6l** (right three columns) were screened against *T. mentagrophytes* at concentrations of 50 (top row), 25, 12.5, and 6.25 (bottom row) μg/mL. The white color is fungal mycelia, indicative of fungal growth, (C) liquid dose-dependency assay of the halogenated 3-substituted-2(*5H*)-oxaboroles against *S. cerevisiae* (**6b**: purple triangles; **6c**: purple square; **6d**: purple circle; **6e**: teal triangle; **6f**: teal square; **6g**: teal circle; **6h**: orange triangle; **6i**: orange square; **6j**: orange circle; **6l**: black square), and (D) liquid dose-dependency assay of the 3-substituted-2(*5H*)-oxaboroles against *E. coli* (**6b**: purple triangles; **6e**: purple square; **6g**: teal circle; **6h**: purple circle; **6k**: teal triangle; **6m**: teal square; **6s**: orange triangle; **6t**: orange square; **6x**: orange circle). Error bars represent standard deviation in (C) and (D).

MICs were calculated using either dose-dependent solid media assays (spore forming pathogens, Figure 2B) or microbroth dilutions (nonspore forming pathogens, Figure 2C,D) with biological replicates (Table 1). The greatest activity was observed against *S. cerevisiae* and *P. chrysogenum*, with MICs as low as 5.20 and 6.25 μg/mL, respectively. Analysis of the antifungal activity showed clear SARs, where the presence of halogen was overall favorable in the *meta* position (**6c**, **6f**, **6i**, and **6l**), less favorable in the *para* position (**6b**, **6e**, and **6h**), and were unfavorable in the *ortho* position (**6d**, **6g**, and **6j**). We hypothesize that a potential steric clash is present in the binding pocket of the target protein, or a preferred conformation is not established, because the substituent in the *ortho* position prevents coplanarity of the aryl and oxaborole rings. Of the halogen substitutions, iodine was the most favorable (**6l**) followed by bromine (**6i**), chlorine (**6f**), and then fluorine (**6c**), losing nearly 4-fold efficacy between **6l** (MIC = 6.25) and **6c** (MIC = 25) regardless of whether the halogen was in the *meta* or *para* position. However, this trend reversed for the substitution at the *ortho* position, further indicating the importance of coplanarity. Finally, bulky (**6q**–**6s**) and methoxy (**6n**–**6p**) substitutions to the aromatic ring were generally unfavorable regardless of position and caused nearly complete loss of growth inhibition. The control compound tavaborole was more potent compared to 3-substituted-2(*5H*)-oxaboroles under identical conditions, and attempts to determine if the 3-substituted-2(*5H*)-oxaboroles shared the same fungal target as **1** were unsuccessful.

**Table 1:**
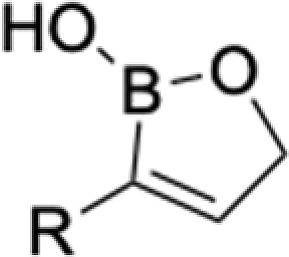
MICs of the 3-Subsutituted-2(*5H*)-oxaboroles^a^

Cmp	R	TM	PC	AF	CA	SC	BC	SA	PA	EC	Tox
**1**	tavaborole	0.78 ± 0 (5.14)	1.0 ± 0.3 (6.58)	0.78 ± 0 (5.14)	2.3 ± 0.8 (15.1)	0.26 ± 0.06 (1.71)	>50 (>329)	∼50 (>329)	>50 (>329)	17 ± 4 (112)	15.2 (100)
**6a**	benzene	>50 (>313)	50 ± 0 (313)	>50 (>313)	>50 (>313)	6.25 ± 0 (39.1)	>50 (313)	>50 (>313)	>50 (>313)	>50 (>313)	NT
**6b**	4-fluoro-benzene	>50 (>281)	25 ± 0 (140)	>50 (>281)	>50 (>281)	10.4 ± 2 (58.4)	>50 (>281)	>50 (>281)	>50 (>281)	42 ± 8 (236)	17.8 (100)
**6c**	3-fluoro-benzene	50 ± 0 (281)	25 ± 0 (140)	50 ± 0 (281)	>50 (>281)	10.4 ± 2 (58.4)	>50 (>281)	>50 (>281)	>50 (>281)	>50 (>281)	17.8 (100)
**6d**	2-fluoro-benzene	>50 (>281)	25 ± 0 (140)	>50 (>281)	>50 (>281)	21 ± 4 (118)	>50 (>281)	>50 (>281)	>50 (>281)	>50 (>281)	17.8 (100)
**6e**	4-chloro-benzene	50 ± 0 (258)	12.5 ± 0 (64.4)	50 ± 0 (258)	>50 (>258)	5.2 ± 1 (26.8)	>50 (>258)	>50 (>258)	>50 (>258)	25 ± 0 (129)	19.4 (100)
**6f**	3-chloro-benzene	25 ± 0 (129)	12.5 ± 0 (64.4)	25 ± 0 (129)	>50 (>258)	21 ± 4 (108)	>50 (>258)	>50 (>258)	>50 (>258)	>50 (>258)	19.4 (100)
**6g**	2-chloro-benzene	>50 (>258)	>50 (>258)	>50 (>258)	>50 (>258)	50 ± 0 (258)	>50 (>258)	>50 (>258)	>50 (>258)	>50 (>258)	NT
**6h**	4-bromo-benzene	33 ± 8 (139)	12.5 ± 0 (52.5)	33 ± 8 (139)	>50 (>210)	12.5 ± 0 (52.5)	>50 (>210)	>50 (>210)	>50 (>210)	21 ± 4 (88.2)	23.9 (100)
**6i**	3-bromo-benzene	25 ± 0 (105)	10.4 ± 2 (43.7)	25 ± 0 (105)	>50 (>210)	50 ± 0 (210)	>50 (>210)	>50 (>210)	>50 (>210)	>50 (>210)	23.9 (100)
**6j**	2-bromo-benzene	>50 (>210)	>50 (>210)	>50 (>210)	>50 (>210)	>50 (>210)	>50 (>210)	>50 (>210)	>50 (>210)	>50 (>210)	NT
**6k**	3-fluoro-5-methyl-benzene	42 ± 8 (219)	12.5 ± 0 (65.1)	42 ± 8 (219)	>50 (>260)	12.5 ± 0 (65.1)	>50 (>260)	>50 (>260)	>50 (>260)	>50 (>260)	19.2 (100)
**6l**	3-iodo-benzene	25 ± 0 (87.4)	6.25 ± 0 (21.9)	25 ± 0 (87.4)	>50 (>175)	>50 (>175)	>50 (>175)	>50 (>175)	>50 (>175)	>50 (>175)	28.6 (100)
**6m**	4-trifluoro-methoxy-benzene	50 ± 0 (205)	12.5 ± 0 (51.2)	50 ± 0 (205)	>50 (>205)	29 ± 11 (119)	>50 (>205)	>50 (>205)	>50 (>205)	>50 (>205)	24.4 (100)
**6n**	4-methoxy-benzene	>50 (>263)	>50 (>263)	>50 (>263)	>50 (>263)	>50 (>263)	>50 (>263)	>50 (>263)	>50 (>263)	>50 (>263)	NT
**6o**	3-methoxy-benzene	>50 (>263)	50 ± 0 (263)	>50 (>263)	>50 (>263)	50 ± 0 (263)	>50 (>263)	>50 (>263)	>50 (>263)	>50 (>263)	NT
**6p**	2-methoxy-benzene	>50 (>263)	>50 (>263)	>50 (>263)	>50 (>263)	>50 (>263)	>50 (>263)	>50 (>263)	>50 (>263)	>50 (>263)	NT
**6q**	naphthalene	>50 (>238)	12.5 ± 0 (59.5)	>50 (>238)	>50 (>238)	>50 (>238)	>50 (>238)	>50 (>238)	>50 (>238)	>50 (>238)	21 (100)
**6r**	5-benzo-furan	50 ± 0 (250)	25 ± 0 (125)	50 ± 0 (250)	>50 (>250)	5.2 ± 1 (26.0)	>50 (>250)	>50 (>250)	>50 (>250)	>50 (>250)	20 (100)
**6s**	2-thiophene	>50 (>301)	50 ± 0 (301)	>50 (>301)	>50 (>301)	10 ± 2 (60.2)	>50 (>301)	>50 (>301)	>50 (>301)	42 ± 8 (253)	NT
**6t**	4-methyl-benzene	>50 (>287)	21 ± 4 (121)	>50 (>287)	>50 (>287)	10.4 ± 2 (59.8)	>50 (>287)	>50 (>287)	>50 (>287)	42 ± 8 (241)	17.4 (100)
**6u**	3-methyl-benzene	42 ± 8 (241)	25 ± 0 (144)	33 ± 8 (190)	>50 (>287)	21 ± 4 (121)	>50 (>287)	>50 (>287)	>50 (>287)	>50 (>287)	17.4 (100)
**6v**	2-methyl-benzene	>50 (>287)	>50 (>287)	>50 (>287)	>50 (>287)	>50 (>287)	>50 (>287)	>50 (>287)	>50 (>287)	>50 (>287)	NT
**6w**	4-t-butyl-benzene	>50 (>232)	12.5 ± 0 (57.9)	>50 (>232)	>50 (>232)	>50 (>232)	>50 (>232)	>50 (>232)	>50 (>232)	>50 (>232)	21.6 (100)
**6x**	4-n-propyl-benzene	50 ± 0 (248)	12.5 ± 0 (61.9)	>50 (>248)	>50 (>248)	10.4 ± 2 (51.5)	>50 (>248)	>50 (>248)	>50 (>248)	21 ± 4 (104)	20.2 (100)
**6y**	cyclohex-1-ene	>50 (>305)	>50 (>305)	>50 (>305)	>50 (>305)	21 ± 4 (128)	>50 (>305)	>50 (>305)	>50 (>305)	>50 (>305)	NT
Cycloheximide		>50 (>178)	12.5 ± 0 (44.4)	>50 (>178)	>50 (>178)	1.56 ± 0 (5.54)	>50 (>178)	>50 (>178)	>50 (>178)	>50 (>178)	NT
Nystatin		3.13 ± 0 (3.38)	1.56 ± 0 (1.68)	2.6 ± 0.5 (2.81)	0.78 ± 0 (0.84)	1.56 ± 0 (1.68)	>50 (>54.0)	>50 (>54.0)	>50 (>54.0)	>50 (>54.0)	NT

a Values are in μg/mL (μM). TM = *T. mentagrophytes*; PC = *P. chrysogenum*; AF = *A. flavus*; CA = *C. albicans*; SC = *S. cerevisiae*; BC = *B. cereus*; SA = *S. aureus*; PA = *P. aeruginosa*; EC = *E. coli*; Tox = concentration screened against H9c2 and hemolysis assay.

In general, very little growth inhibition was observed against either Gram-positive or Gram-negative bacterial pathogens. None of the 3-substituted oxaborole derivatives exhibited activity against *B. cereus*, *S. aureus* MRSA, or *P. aeruginosa*. There was modest activity against *E. coli*, with several compounds possessing MICs between 20 and 50 μg/mL (**6b**, **6e**, **6h**, **6k**, **6s**, **6t**, and **6x**; Figure 2D). The SAR for the antibacterial is dissimilar to that observed for the antifungal properties. Analogs with aromatic substitutions in the *para* position were more active than those with substitutions in the *meta* or *ortho* positions. For example, compounds **6b**, **6e**, and **6h** are all *para*-substituted aryl halides and were the only aryl halides possessing activity against *E. coli* (MIC 21–42 μg/mL). The compounds that exhibited MICs ≤ 25 μg/mL against at least one pathogen, excluding *S. cerevisiae*, were subsequently evaluated for mammalian cytotoxicity and hemolytic activity. To our delight, none of the compounds (**6b**–**6f**, **6h**, **6i**, **6k**–**6m**, **6q**, **6r**, **6t**, **6u, 6w**, and **6x**) had cytotoxic effects against rat myoblast cells (H9c2) and none possessed hemolytic activity at concentrations up to 100 μM (Figure 3).

**Figure 3:**
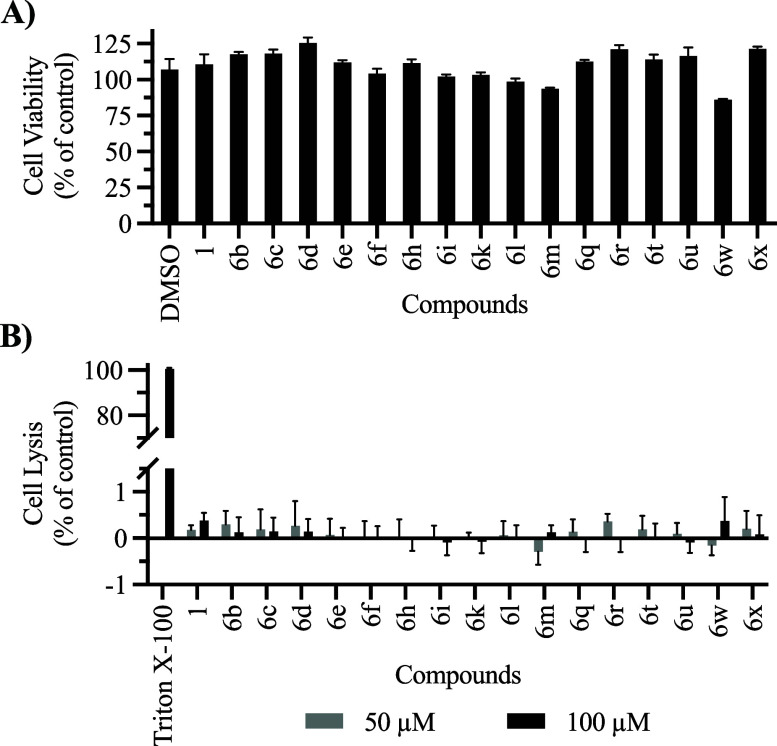
(A) Cell viability assay against H9c2 cell line at 100 μM for 24 h (normalized to PBS no treatment), (B) Hemolytic assay at 50 and 100 μM for 1 h (normalized to triton X-100 positive control). Error bars in both graphs represent standard error of mean (SEM) of replicates (*n* = 3).

In summary, a series of 3-substituted-2(*5H*)-oxaboroles were synthesized and evaluated for growth inhibition against a panel of microbial pathogens. Our studies revealed growth inhibition of fungi (*T. mentagrophytes*, *P. chrysogenum*, *A. flavus*) and yeast (*S. cerevisiae*) with MICs as low as 6.25 and 5.20 μg/mL, respectively. Structure–activity profiling suggests a strong preference for halogens in the *meta* position in fungal pathogens. Furthermore, we demonstrated that 3-substituted oxaboroles are nonhemolytic and nontoxic to mammalian cells. Current efforts are directed toward understanding the mechanism of action of these novel compounds.

## Supporting Information Available

Experimental sections (materials and methods), compound characterization, NMR spectra, chromatograms of 3-substituted-2(*5H*)-oxaboroles, antimicrobial data, and hemolytic results (PDF)

## Abbreviations

SAR: Structure–activity relationship
MIC: minimum inhibitory concentration
PDE4: phosphodiesterase 4
cAMP: cyclic adenosine monophosphate
MRSA: methicillin-resistant *Staphylococcus aureus*
H9c2: rat myoblast cells
