# Supplementary material for: Design, Synthesis, and Antifungal Activity of 3-Substituted-2(5H)-Oxaboroles
Source: ACS Med Chem Lett. 2024 Feb 22;15(3):349–54. doi: 10.1021/acsmedchemlett.3c00463 (PMC10945556; doi:10.1021/acsmedchemlett.3c00463)
Supplement: Supplementary file 1 — ml3c00463_si_001.pdf [file ml3c00463_si_001.pdf]

Supporting Information

Design, synthesis, and antifungal activity of 3-substituted-2(*5H*)-Oxaboroles

Rose Campbell,[†,#] Nicklas W. Buchbinder,[†,#] Connor Szwetkowski,[†] Yumeng Zhu,[†] Karla Piedl,[†] Mindy Truong,[†] John B. Matson,[†] Webster L. Santos,[†] Emily Mevers[†,]*

[†]*Department of Chemistry, Virginia Tech, Blacksburg, Virginia, 24061 USA*

Table of Contents

Experimental Procedures S2

References S5

Table S1: References for 3-substituted-2(*5H*)-oxaborole substrates S6

Chemical descriptions S8

NMR spectra (Figs. S1 – S101) S15

HPLC chromatograms of the oxaboroles (Figs. S102 – S126) S78

Preliminary antimicrobial assays (Figs. S127 – S132) S94

Liquid dose-dependent assays (Figs. S133 – S151) S97

Solid MIC assays (Figs. S152 – S194) S107

1) EXPERIMENTAL PROCEDURES

General Experimental Procedures

NMR spectra were recorded with deuterated methanol and d6-DMSO with the residual solvent peak as internal standards (δ_C 49.15, δ_H 3.31 for CH_2DOD, and δ_C 39.52, δ_H 2.50 for d_6-DMSO) on Bruker Avance III 600 MHz instrument equipped with a triple resonance inverse (CP-TCI) Prodigy N2 cooled CryoProbe (600 and 150 MHz for ^{1}H and ^{13}C NMR, respectively). LR-LCMS data were obtained using an Agilent 1200 series HPLC system equipped with a photo-diode array detector and a Thermo LTQ mass spectrometer. HR-ESIMS was carried out using a Shimadzu LC-q-TOF Mass Spectrometer equipped with an uHPLC system. Purity assessments were performed using a Waters UPLC or an Agilent 1200 series HPLC.

Safety Statement

No unexpected or unusually high safety hazards were encountered. All chemicals were used in accordance with their corresponding safety data sheets. Specifically, *n*-butyllithium was only used under inert atmosphere and in the fume hood. Syringes used to transfer *n*-butyllithium were quenched before disposal. *n*-Tributylphosphine was only used under inert atmosphere and in the fume hood. Always read chemical safety data sheets when replicating the reported synthetic protocols.

General Synthetic Procedures

*General Synthesis of Alkynoates **9a** - **9j**, **9n**, **9p** - **9q**, and **9r** - **9y**:* Modified from literature procedure.[1] To a flame dried round bottom flask: a stir bar and acetylene (1.0 equiv) **7a** - **7j**, **7n**, **7p** - **7q** and **7r** - **7y** was added. The reaction was put under an inert atmosphere using standard *Schlenk* technique. Dry THF was then added to the round bottom flask. The reaction flask was then put into a dry ice/acetone bath (-78 °C). *n*-butyllithium (2.5 M in hexanes, 1.1 equiv) was then added dropwise and the reaction was allowed to stir for 1 hour. Next, methyl chloroformate (1.1 equiv) was added dropwise at -78 °C. After 1 h, the reaction flask was opened and diluted with ethyl acetate followed by water. The organic layer was washed with brine (aq) and dried over sodium sulfate. Volatiles were removed under negative pressure and the the reaction mixture was loaded onto silica gel and purified via flash chromatography (0 - 10% EtOAc:Hex) to afford alkynoates **9a** - **9j**, **9n**, **9p** - **9q**, and **9r** - **9y**.

*General Synthesis of Alkynoates **9k** - **9m**, **9o**, and **9s**:* Modified from literature procedure.[2] To a flame dried six-dram vial: a stir bar, palladium bis(triphenylphosphine) dichloride (5 mol%) and copper iodide (10 mol%) were added. The reaction was put under an inert atmosphere using standard *Schlenk* technique. Next, dry THF, aryl iodides (1.0 equiv) **8k** - **8m**, **8o**, **8s**, methyl propiolate (1.2 equiv) and triethylamine (3.0 equiv) were added. The reaction was allowed to stir for 16 h at 70 °C. The reaction was diluted with ethyl acetate followed by water. The organic layer was washed with brine (aq) and dried over sodium sulfate. Volatiles were removed under negative pressure and the reaction mixture was loaded onto silica gel and purified via flash chromatography (0 - 10% EtOAc:Hex) to afford alkynoates **9k** - **9m**, **9o**, and **9s.**

*General Synthesis of (E)-3-boryl-acrylates (**10a** - **10y**):* Modified from literature procedure.[3] To a flame dried six-dram vial: a stir bar and alkynoate (1.0 equiv) **9a** - **9y** were added. The reaction flask was put under an inert atmosphere using standard *Schlenk* technique. Dry THF was then added for solid alkynoates while oils were reacted neat. Subsequently, n-tributylphosphine (0.3

equiv) and pinacolborane (1.2 equiv) were added. Reaction progress was followed via TLC analysis. Upon completion or after 3 h, volatiles were removed under negative pressure and the reaction mixture was loaded onto silica gel and purified via flash chromatography (0 - 20% EtOAc:Hex) to afford (*E*)-3-boryl-acrylates **10a** - **10g, 10i - 10j, 10l, 10n, 10p - 10q, 10t - 10w, 10y**. In some cases, (**10h**, **10k**, **10m**, **10o**, **10r**, **10s**, **10x)** (*E*)-3-boryl-acrylates were not purified and instead the crude reaction mixtures were used in the next step.

*General Synthesis of 3-substituted 2(5H)-oxaboroles (**6a** - **6y**):* Modified from literature procedure.[3] To a six-dram vial charged with a stir bar: (*E)*-3-borylacrylates (1.0 equiv) **10a** - **10y** were dissolved in ethanol and sodium borohydride (2.0 equiv) was added. The reaction was stirred at room temperature until starting material was consumed via TLC analysis. Upon completion, 5 mL of H_2O was added to the reaction flask. The mixture was extracted with 5 mL of EtOAc (3x). The organic layers were combined and dried over sodium sulfate. After decanting, volatiles were removed under negative pressure and the 3-substituted *2(5H)*-oxaboroles **6a** - **6y** were purified via flash column chromatography.

Microbiological Experimental Details

Screening for Antibacterial and Antifungal Activity: All compounds except for **6l**, **6p**, **6v**, and tavaborole (**1**) were first screened in technical duplicates for activity against *Candida albicans, Saccharomyces cerevisiae, Bacillus cereus, Escherichia coli* ATCC 25922, *Pseudomonas aeruginosa* ΔMexAB-OprM, and methicillin resistant *Staphylococcus aureus* (MRSA). Both *C. albicans* and *S. cerevisiae* were tested in yeast extract, peptone, and dextrose (YPD) liquid media with an initial inoculation of pathogen at 0.0024 OD_{600}. All bacterial strains (*B. cereus*, *E. coli*, *P. aeruginosa*, and *S. aureus*) were screened using Luria-Bertani (LB) liquid media with an initial bacterial inoculation of an OD_{600} of 0.004. Stock solutions of each oxaborole were prepared in DMSO at 4 and 1 mg/mL. An aliquot (2.5 µL) of each compound was transferred to a 96-well plate followed by 197.5 µL of liquid media containing the pathogen, for final screening concentrations of 50 and 12.5 µg/mL. Inoculated plates were incubated for 24 h statically at 30ºC for *S. cerevisiae* and 37ºC for all other microbes. After incubation, settled cells in each well were resuspended by pipetting, and absorbances were read at 600 nm on a BioTek S16 Synergy Mx plate reader (BioTek, Winooski, VT). Absorbance of media control (negative control) was subtracted from all wells, and OD_{600} readings were normalized to the growth control containing vehicle (DMSO) alone.

Minimum Inhibitory Concentration (MIC) Liquid Assays: To determine the MIC of each active compound, all compounds exhibiting >95% growth inhibition at 50 µg/mL in the screening assay, **6l**, **6p**, **6v**, **1** (MedChemExpress, Monmouth Junction, NJ), cycloheximide (Sigma-Aldrich, St. Louis, MO), and nystatin (Alfa Aesar, Ward Hill, MA) were tested using the same method as above, but in biological triplicate, each with technical replicates (triplicates) and at 50, 25, 12.5, and 6.25 µg/mL. Follow-up MIC assays at lower concentrations were performed in cases where growth inhibition >95% was seen at 6.25 µg/mL. MIC values were determined as the lowest concentration exhibiting ≥95% growth inhibition. MICs of all biological replicates were averaged and standard error of mean was calculated.

Minimum Inhibitory Concentration (MIC) Solid Assays: Solid MIC assays were performed with all compounds (**6a - y**), **1**, cycloheximide, and nystatin in biological triplicate against the following

spore-forming fungi: *Trichophyton mentagrophytes* var. *Quinckeanum* NRRL Y-14*, Penicillium chrysogenum* NRRL 807*,* and *Aspergillus flavus* NRRL 1957 and followed a previously reported method.[4] Working stocks were prepared in DMSO at four serial dilutions: 5, 2.5, 1.25, and 0.625 mg/mL. An aliquot (100 µL) of each working stock was added to 9.9 mL of YPD cooled to 55 ºC (1.5% agar) in a 15-mL falcon tube yielding final concentrations of 50, 25, 12.5, and 6.25 µg/mL. Falcon tubes were inverted several times to homogenize the mixture, then 1 mL was transferred to each well of a 24-well plate (Greiner Bio-One, Monroe, NC). An aliquot (5 µL) of spore stocks of each of the spore-forming pathogens was serially diluted by suspending it first in 120 µL of sterile water, then transferring 10 µL to 390 µL of sterile water. Each well was inoculated with 2 µL of this spore suspension. Inoculated plates were incubated at 30 ºC for 48 h, at which point they were photographed and analyzed for growth inhibition. Wells were prepared in technical replicates (triplicate) with vehicle controls (DMSO). MICs of all biological replicates were averaged and standard error of mean was calculated.

Hemolytic Assay: The most active 2(*5H*)oxaboroles (**1**, **6b** - **6f**, **6h**, **6i**, **6k** - **6m**, **6q**, **6r**, **6t**, **6u**, **6w** - **6x**) were screened for hemolytic activity at 50 and 100 µM in biological triplicates, each with technical duplicates using defibrinated sheep blood (Lampire Biological Laboratories, Pipersville, PA) and following previously reported methods.[5] Briefly, the defibrinated sheep blood was centrifuged (4000 rpm, 10 min), then the plasma layer was aspirated and replaced with an equal volume of 150 mM NaCl solution; this wash step was repeated three more times, with the plasma layer being replaced with phosphate buffer (PBS, pH = 7.4) in the final two wash steps. The blood was centrifuged one final time and the plasma layer was aspirated, then 1 mL of the remaining erythrocytes was transferred to 49 mL of PBS and mixed gently. Cells were allowed to settle to ensure no cell lysis had occurred, then resuspended by gently inverting before using in remaining steps. Test compounds were resuspended at 5 and 10 mM in DMSO and 2 µL were transferred to wells of a 96-well V-bottom plate in technical duplicates for each compound at both concentrations, as well as 8 µL of PBS. Compounds were then diluted with 190 µL of the prepared blood cell mixture for final concentrations of 50 and 100 µM. Negative controls were prepared using 10 µL of PBS, and positive controls were prepared by adding 10 µL of 20% Triton X-100 solution. The 96-well plate was incubated at 37ºC for one hour, then centrifuged (4000 rpm, 10 min). Finally, 100 µL of the supernatant from each well was transferred to a 96-well flat-bottom plate and absorbance was measured at 415 nm. Data were graphed using GraphPad Prism 9. Mean values are reported together with the standard error of mean (SEM) representing the combination of 3 biological replicates.

Mammalian Cell Culture: Cell studies were conducted on an adherent H9C2 line of rat embryonic cardiomyocytes (ATCC, Manassas, VA, USA). Cultures were grown in Dulbecco's modified Eagle medium (DMEM, VWR, Radnor, PA), supplemented with 10% fetal bovine serum (FBS, VWR, Radnor, PA), 50 IU/mL penicillin, and 50 µg/mL streptomycin (MP Biomedicals, Santa Ana, California, USA). Cells were cultured at 37 °C in 5% CO_2-air. The cultures were passaged after 70–80% confluence was achieved. Cells were rinsed with 1X PBS solution three times, and then released with 0.05% trypsin-0.53 mM EDTA solution (VWR, Radnor, PA). The suspension of released cells was centrifuged at 1000 rpm for 5 min. Dulbecco's phosphate-buffered saline (DPBS) was purchased from Gibco (Thermo Fisher Scientific, Inc., Waltham, MA, USA).

Cell Viability Assays: H9C2 cells were plated in a 96-well plate at a density of 5000 cells per well in 200 μL complete DMEM (with phenol red and serum) per well. After culturing for 24 h, cells were washed with 1X PBS three times before 90 μL serum-containing DMEM media was added to each well. Next, 10 μL of antibiotic solution (1 mM, 10% DMSO in water) was added to each well to create a final concentration of 100 μM, 1% DMSO. After incubation for 24 h, cells were washed three times with PBS and then treated with serum-free DMEM media (100 μL) and 10 μL Cell counting kit 8 solution (CCK-8, Dojindo, Rockville, MD). After incubation for another 3 h to allow for development of the CCK8 dye, absorbance was recorded at 450 and 750 nm using a BioTek S16 Synergy Mx plate reader (BioTek, Winooski, VT). Final absorbance was calculated by subtracting Abs_{750nm} from Abs_{450nm}. Data were then graphed using GraphPad InStat, version 3 (GraphPad Software, Inc., San Diego, CA). Mean values are reported together with the standard error of mean (SEM) representing the combination of 3 different experimental runs.

2) REFERENCES

(1) Cacchi, S.; Fabrizi, G.; Moro, L.; Pace, P. Stereo- and Regioselective Palladium-Catalysed Hydroarylation and Hydrovinylation of Functionalised Alkynes: A Route to Substituted Z-2-Cinnamyl Esters, 3-Chromen-2-Ols, and Coumarins. *Synlett* **1997**, *12* (12), 1367–1370.

(2) Eckert, T.; Ipaktschi, J. A New Method for Synthesis of Methyl Arylpropiolates by Direct Heck Coupling of Aryl Iodide and Methyl Propiolate in Presence of K2CO3. *Synth. Commun.* **1998**, *28* (2), 327–335.

(3) Fritzemeier, R.; Gates, A.; Guo, X.; Lin, Z.; Santos, W. L. Transition Metal-Free Trans Hydroboration of Alkynoic Acid Derivatives: Experimental and Theoretical Studies. *J. Org. Chem.* **2018**, *83* (17), 10436–10444.

(4) Mevers, E.; Chouvenc, T.; Su, N.-Y.; Clardy, J. Chemical Interaction among Termite-Associated Microbes. *J. Chem. Ecol.* **2017**, *43* (11-12), 1078–1085.

(5) Evans, B. C.; Nelson, C. E.; Yu, S. S.; Beavers, K. R.; Kim, A. J.; Li, H.; Nelson, H. M.; Giorgio, T. D.; Duvall, C. L. Ex Vivo Red Blood Cell Hemolysis Assay for the Evaluation of pH-Responsive Endosomolytic Agents for Cytosolic Delivery of Biomacromolecular Drugs. *J. Vis. Exp.* **2013**, No. 73, e50166.

Table S1: References for 3-substituted-2(*5H*)-oxaborole substrates

**Substrate**	**R**	**Reference**
**Alkynoates**		
**9a**	benzene	DOI: 10.1021/acs.joc.8b01493
**9b**	4-fluorobenzene	DOI: 10.1021/acs.joc.8b01493
**9c**	3-fluorobenzene	DOI: 10.1002/anie.202008572
**9d**	2-fluorobenzene	DOI: 10.1021/acs.joc.8b01493
**9e**	4-chlorobenzene	DOI: 10.1021/acs.joc.8b01493
**9f**	3-chlorobenzene	DOI: 10.1021/acs.joc.8b01493
**9g**	2-chlorobenzene	DOI: 10.1021/acs.joc.8b01493
**9h**	4-bromobenzene	DOI: 10.1021/acs.joc.2c02610
**9i**	3-bromobenzene	DOI: 10.1021/acs.joc.2c02610
**9j**	2-bromobenzene	DOI: 10.1002/anie.202008572
**9m**	4-trifluoromethoxybenzene	DOI: 10.1021/acs.joc.8b01493
**9n**	4-methoxybenzene	DOI: 10.1021/acs.joc.8b01493
**9o**	3-methoxybenzene	DOI: 10.1021/acs.joc.2c02610
**9p**	2-methoxybenzene	DOI: 10.1021/acs.joc.8b01493
**9q**	2-naphthalene	DOI: 10.1021/acs.joc.8b01493
**9r**	5-benzofuran	DOI: 10.1021/acs.joc.2c02610
**9s**	2-thiophene	DOI: 10.1002/anie.202006096
**9t**	4-methylbenzene	DOI: 10.1021/acs.joc.8b01493
**9u**	3-methylbenzene	DOI: 10.1021/acs.joc.8b01493
**9v**	2-methylbenzene	DOI: 10.1002/anie.202006096
**9w**	4-t-butylbenzene	DOI: 10.1021/acs.joc.8b01493

**Cont. Table S1:** References for 3-substituted-2(*5H*)-oxaborole substrates		
**Substrate**	**R**	**Reference**
**9x**	4-n-propylbenzene	DOI: 10.1002/chem.201800526
**9y**	cyclohex-1-ene	DOI: 10.1021/acs.joc.8b01493
**(*E*)-3-boryl-acrylates**		
**10a**	benzene	DOI: 10.1021/acs.joc.8b01493
**10b**	4-fluorobenzene	DOI: 10.1021/acs.joc.8b01493
**10d**	2-fluorobenzene	DOI: 10.1021/acs.joc.8b01493
**10e**	4-chlorobenzene	DOI: 10.1021/acs.joc.8b01493
**10f**	3-chlorobenzene	DOI: 10.1021/acs.joc.8b01493
**10g**	2-chlorobenzene	DOI: 10.1021/acs.joc.8b01493
**10h**	4-bromobenzene	DOI: 10.1039/C8OB01343H
**10n**	4-methoxybenzene	DOI: 10.1021/acs.joc.8b01493
**10p**	2-methoxybenzene	DOI: 10.1021/acs.joc.8b01493
**10q**	naphthalene	DOI: 10.1021/acs.joc.8b01493
**10t**	4-methylbenzene	DOI: 10.1021/acs.joc.8b01493
**10u**	3-methylbenzene	DOI: 10.1021/acs.joc.8b01493
**10w**	4-t-butylbenzene	DOI: 10.1021/acs.joc.8b01493
**10y**	cyclohex-1-ene	DOI: 10.1021/acs.joc.8b01493

3) CHEMICAL DESCRIPTIONS

*3-Phenyl-1,2-oxaborol-2(5H)-ol (**6a**):* 870.1 mg, 5.44 mmol, 77%, white solid. **^{1}H NMR** (400 MHz, $CDCl_3$) δ_H 7.65 (d, *J* = 8.4 Hz, 2H), 7.43–7.32 (m, 3H), 7.30–7.23 (m, 1H), 6.45 (s, 1H), 4.71 (s, 2H). **^{13}C NMR** (101 MHz, $CDCl_3$): δ_C 147.4, 135.9, 128.7, 127.6, 126.9, 71.8, (*C*-B not observed). **^{11}B NMR** (128 MHz, $CDCl_3$) δ_B 32.6. **HRMS**: (ESI-TOF) [M-H]$^-$ calc. for $C_9H_8BO_2^-$: 159.0623 m/z; Found 159.0621 m/z. Δ -1.2574 ppm.

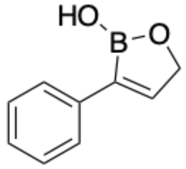

*3-(4-fluorophenyl)-1,2-oxaborol-2(5H)-ol (**6b**)*: 114.0 mg, 0.641 mmol, 70%, white solid. **^{1}H NMR** (400 MHz, $CDCl_3$) δ_H 7.65 – 7.60 (m, 2H), 7.37 (s, 1H), 7.04 (t, *J* = 8.8 Hz, 2H), 5.23 (s, 1H), 4.69 (d, *J* = 1.3 Hz, 2H). **^{13}C NMR** (101 MHz, $CDCl_3$) δ_C 162.5 (d, J = 246.5 Hz), 146.9 (d, J = 1.9 Hz), 131.9 (d, J = 3.3 Hz), 128.6 (d, J = 7.8 Hz), 115.5 (d, J = 21.3 Hz). 71.8**,** (*C*-B not observed). **^{19}F NMR** (376 MHz, $CDCl_3$) δ_F -115.4. **^{11}B NMR** ($CDCl_3$, 128 MHz) δ_B 32.5. **HRMS** (ESI-TOF) [M-H]$^-$ calc. for $C_9H_7BFO_2^-$: 177.0523 m/z; Found 177.0518 m/z. Δ -2.8240 ppm.

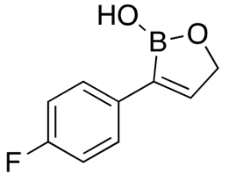

*3-(3-fluorophenyl)-1,2-oxaborol-2(5H)-ol (**6c**)*: 37.8 mg, 0.317 mmol, 67%, white solid. **^{1}H NMR** (400 MHz, $CDCl_3$) δ_H 7.46 (s, 1H), 7.43 – 7.40 (m, 1H), 7.35 – 7.29 (m, 2H), 6.96 (ddt, *J* = 8.7 Hz, 2.8 Hz, 1.1 Hz, 1H), 4.72 (s, 1H), 4.69 (d, *J* = 1.7 Hz, 2H). **^{13}C NMR** (126 MHz, $CDCl_3$) δ_C 163.2 (d, *J* = 245.1 Hz), 149.0, 138.1 (d, *J* = 8.0 Hz), 130.1 (d, *J* = 8.4 Hz), 122.6 (d, *J* = 2.8 Hz), 114.4 (d, *J* = 21.2 Hz), 113.8 (d, *J* = 21.4 Hz), 71.8, (*C*-B not observed). **^{11}B NMR** (128 MHz, $CDCl_3$): δ_B 32.4. **^{19}F NMR** (376 MHz, $CDCl_3$) δ_F -113.4 (s). **HRMS**: (ESI-TOF) [2M+CH_3COO]$^-$ calc. for $C_{20}H_{19}B_2F_2O_6^-$: 415.1341 m/z; Found 415.1360 m/z. Δ 4.5768 ppm.

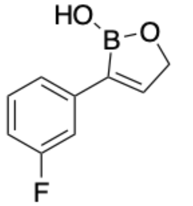

*3-(2-fluorophenyl)-1,2-oxaborol-2(5H)-ol (**6d**)*: 6.02 mg, 0.033 mmol, 26%, white solid. **^{1}H NMR** (500 MHz, $CDCl_3$) δ_H 7.71 (t, *J* = 7.9 Hz, 1H), 7.63 (s, 1H), 7.24 (dd, *J* = 10.5, 4.8 Hz, 1H), 7.15 (t, *J* = 7.5 Hz, 1H), 7.08 (dd, *J* = 11.6, 8.1 Hz, 1H), 5.88 – 5.83 (m, 1H), 4.74 (s, 2H).**^{13}C NMR** (126 MHz, $CDCl_3$) δ_C 160.8 (d, *J* = 248.4 Hz), 152.3 (d, *J* = 8.6 Hz), 130.4 (d, *J* = 4.5 Hz), 128.9 (d, *J* = 8.6 Hz), 124.5 (d, *J* = 3.6 Hz), 123.7 (d, *J* = 12.7 Hz), 115.9 (d, *J* = 22.7 Hz), 72.2, (*C*-B not observed). **^{11}B NMR** (128 MHz, $CDCl_3$) δ_B 32.6. **^{19}F NMR** (376 MHz, $CDCl_3$) δ_F -114.3 (s). **HRMS**: (ESI-TOF) [2M-H]$^-$ calc. for $C_{18}H_{15}B_2F_2O_4^-$: 355.1130 m/z; Found 355.1122 m/z. Δ -2.2528 ppm.

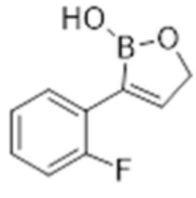

*3-(4-chlorophenyl)-1,2-oxaborol-2(5H)-ol (**6e**):* 653.9 mg, 3.37 mmol, 72%, white solid. **^{1}H NMR** (400 MHz, $CDCl_3$) δ_H 7.58 (d, *J* = 8.5 Hz, 2H), 7.42 (s, 1H), 7.32 (d, *J* = 8.5 Hz, 2H), 5.42 (s, 1H), 4.70 (s, 2H). **^{13}C NMR** ($CDCl_3$, 100 MHz) δ_C 148.1, 134.3, 133.4, 128.9, 128.3, 71.8, (*C*-B not observed). **^{11}B NMR** ($CDCl_3$, 128 MHz) δ_B 32.5. **HRMS:** (ESI-TOF) $[M-H]^-$ calc. for $C_9H_7BClO_2^-$: 193.0228 m/z; Found 193.0227 m/z. Δ -0.5181 ppm.

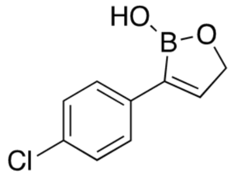

*3-(3-chlorophenyl)-1,2-oxaborol-2(5H)-ol (**6f**)*: 117.0 mg, 0.600 mmol, 78%, white solid. **^{1}H NMR** (400 MHz, $CDCl_3$) δ_H 7.63 (t, *J* = 1.9 Hz, 1H), 7.52 (dt, *J* = 7.5, 1.5 Hz, 1H), 7.46 (s, 1H), 7.30 (d, *J* = 7.9 Hz, 1H), 7.24 (dt, *J* = 8.1, 1.6 Hz, 1H), 5.11 (s, 1H), 4.70 (d, *J* = 1.5 Hz, 2H).**^{13}C NMR** (101 MHz, $CDCl_3$) δ_C 148.9, 137.5, 134.4, 129.8, 127.4, 126.9, 124.9, 71.6, (*C*-B not observed). **^{11}B NMR** (128 MHz, $CDCl_3$) δ_B 32.5. **HRMS:** (ESI-TOF) $[M-H]^-$ calc. for $C_9H_7BClO_2^-$: 193.0233 m/z; Found 193.0228 m/z. Δ -2.5904 ppm.

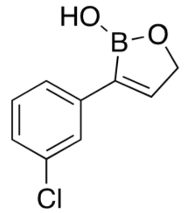

*3-(2-chlorophenyl)-1,2-oxaborol-2(5H)-ol (**6g**)*: 10.9 mg, 0.167 mmol, 34%, white solid. **^{1}H NMR** (500 MHz, $CDCl_3$) δ_H 7.43 (s, 1H), 7.40 (dd, *J* = 7.9, 1.4 Hz, 1H), 7.36 (dd, *J* = 7.6, 1.9 Hz, 1H), 7.27 – 7.19 (m, 2H), 5.29 (s, 1H), 4.73 (d, *J* = 1.5 Hz, 2H).**^{13}C NMR** (126 MHz, $CDCl_3$) δ_C 153.3, 135.7, 132.1, 130.1, 129.8, 128.4, 127.0, 71.9, (*C*-B not observed). **^{11}B NMR** (128 MHz, $CDCl_3$) δ_B 32.3. HRMS: (ESI-TOF) $[M-H+H_2O]^-$ calc. for $C_9H_9BClO_3^-$: 211.0339 m/z; Found 211.0331 m/z. Δ -3.7909 ppm.

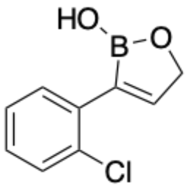

*3-(4-bromophenyl)-1,2-oxaborol-2(5H)-ol (**6h**)*: 206.7 mg, 0.865 mmol, 65%, white solid.**^{1}H NMR** (400 MHz, $CDCl_3$) δ_H 7.52 (d, *J* = 8.7 Hz, 2H), 7.47 (d, *J* = 8.7 Hz, 2H), 7.44 (s, 1H), 4.95 (s, 1H), 4.68 (d, *J* = 1.7 Hz, 2H). **^{13}C NMR** (101 MHz, $CDCl_3$) δ_C 148.1, 134.1, 131.7, 128.4, 121.4, 71.7, (*C*-B not observed). **^{11}B NMR** (128 MHz, $CDCl_3$) δ_B 32.5. **HRMS** (ESI-TOF) $[M-H]^-$ calc. for $C_9H_7BBrO_2^-$: 236.9730 m/z; Found 236.9737 m/z. Δ 2.9539 ppm.

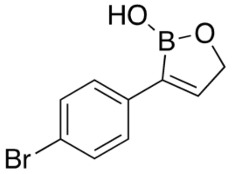

*3-(3-bromophenyl)-1,2-oxaborol-2(5H)-ol (**6i**):* 159.0 mg, 0.665 mmol, 50%, white solid. **^{1}H NMR** (600 MHz, $CDCl_3$) δ_H 7.79 (t, *J* = 1.8 Hz, 1H), 7.58 (dt, *J* = 7.7, 1.4 Hz, 1H), 7.44 (d, *J* = 1.7 Hz, 1H), 7.39 (ddd, *J* = 8.0, 2.1, 1.1 Hz, 1H), 7.22 (t, *J* = 7.8 Hz, 1H), 5.91 (s, 1H), 4.72 (d, *J* = 1.7 Hz, 2H). **^{13}C NMR** (151 MHz, $CDCl_3$) δ_C 148.8, 130.5, 130.2, 129.9, 125.6, 122.9, 71.8, (*C*-B not observed). **^{11}B NMR** (128 MHz, $CDCl_3$) δ_B 32.3. **HRMS**: (ESI-TOF) $[M-H]^-$ calc. for $C_9H_7BBrO_2^-$: 236.9722 m/z; Found 236.9716 m/z. Δ -2.5320 ppm.

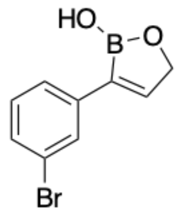

*3-(2-bromophenyl)-1,2-oxaborol-2(5H)-ol (**6j**):* 143.1 mg, 0.600 mmol, 45%, white solid. **^{1}H NMR** (500 MHz, $CDCl_3$) δ_H 7.59 (d, *J* = 7.5 Hz, 1H), 7.34 (s, 1H), 7.30 (dd, *J* = 6.8, 1.8 Hz, 2H), 7.16 – 7.10 (m, 1H), 4.95 (s, 1H), 4.72 (d, *J* = 1.5 Hz, 2H). **^{13}C NMR** (126 MHz, $CDCl_3$) δ_C 153.3, 138.2, 133.0, 130.1, 128.7, 127.7, 122.0, 71.9, (*C*-B not observed). **^{11}B NMR** (128 MHz, $CDCl_3$) δ_B 32.1 **HRMS**: (ESI-TOF) [M-H]$^-$ calc. for $C_9H_7BBrO_2^-$: 236.9728 m/z; Found 236.9734 m/z. Δ 2.5319 ppm.

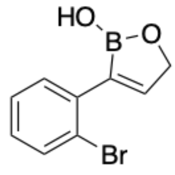

*3-(3-fluoro-5-methylphenyl)-1,2-oxaborol-2(5H)-ol (**6k**):* 103.3 mg, 0.539 mmol, 59%, white solid **^{1}H NMR** (400 MHz, $CDCl_3$) δ_H 7.40 (s, 1H), 7.31 (d, *J* = 7.8 Hz, 2H), 7.15 (t, *J* = 7.9 Hz, 1H), 5.22 (s, 1H), 4.69 (d, *J* = 1.7 Hz, 2H), 2.27 (s, 3H). **^{13}C NMR** (100 MHz, $CDCl_3$) δ_C 161.6 (d, J = 244.1 Hz), 147.9, 131.6 (d, J = 5.6 Hz), 124.1 (d, J = 17.5 Hz), 122.4 (d, J = 3.2 Hz), 113.3 (d, J = 22.4 Hz), 71.7, 14.5 (d, J = 3.6 Hz), (*C*-B not observed). **^{19}F NMR** (376 MHz, $CDCl_3$) δ_F –118.4. **^{11}B NMR** (128 MHz $CDCl_3$) δ_B 32.5. **HRMS** (ESI-TOF) [M-H]$^-$ calc. for $C_{10}H_9BFO_2^-$: 191.0680 m/z; Found 191.0685 m/z. Δ 2.6169 ppm.

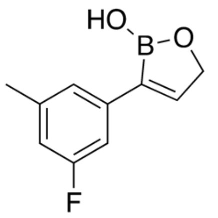

*3-(3-iodophenyl)-1,2-oxaborol-2(5H)-ol (**6l**):* 22.0 mg, 0.076 mmol, 58%, white solid. **^{1}H NMR** (500 MHz, $CDCl_3$) δ_H 7.98 (d, *J* = 1.7 Hz, 1H), 7.60 (dt, *J* = 7.2, 1.5 Hz, 2H), 7.44 (s, 1H), 7.09 (t, *J* = 7.8 Hz, 1H), 4.90 (s, 1H), 4.69 (d, *J* = 1.7 Hz, 2H). **^{13}C NMR** (126 MHz, $CDCl_3$) δ_C 149.1, 138.1, 136.5, 135.8, 130.4, 126.3, 94.9, 71.8, (*C*-B not observed). **^{11}B NMR** (128 MHz, $CDCl_3$) δ_B 32.4. **HRMS**: (ESI-TOF) [M-H]$^-$ calc. for $C_9H_7BIO_2^-$: 284.9589 m/z; Found 284.9596 m/z. Δ 2.4565 ppm.

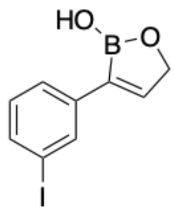

*3-(4-(trifluoromethoxy)phenyl)-1,2-oxaborol-2(5H)-ol (**6m**):* 135.2 mg, 0.554 mmol, 69%, white solid. **^{1}H NMR** (400 MHz, $CDCl_3$) δ_H 7.68 (d, J = 8.7 Hz, 2H), 7.41 (s, 1H), 7.20 (d, J = 8.2 Hz, 2H), 6.54 (s, 1H), 4.73 (d, J = 1.3 Hz, 2H). **^{13}C NMR** (101 MHz, $CDCl_3$) δ_C 148.5 (q, *J* = 1.9 Hz), 147.8, 134.4, 128.2, 121.0, 120.5 (q, *J* = 256.9 Hz), 71.6, (*C*-B not observed). **^{19}F NMR** (376 MHz, $CDCl_3$) δ_F -57.8 .**^{11}B NMR** ($CDCl_3$, 128 MHz) δ_B 32.4. **HRMS** (ESI-TOF) [M-H]$^-$ calc. for $C_{10}H_7BF_3O_3^-$: 243.0440 m/z; Found 243.0437 m/z. Δ -1.2343 ppm.

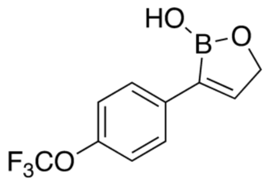

*3-(4-methoxyphenyl)-1,2-oxaborol-2(5H)-ol (**6n**):* 73.7 mg, 0.388 mmol, 63%, white solid. **^{1}H NMR** (400 MHz, $CDCl_3$) δ_H 7.59 (d, *J* = 8.8 Hz, 2H), 7.31 (s, 1H), 6.90 (d, *J* = 8.8 Hz, 2H), 5.10 (s, 1H), 4.67 (d, *J* = 1.5 Hz, 2H), 3.82 (s, 3H). **^{13}C NMR** ($CDCl_3$, 100 MHz) δ_C 159.2, 145.7, 128.2, 114.1, 71.8, 55.4, (*C*-B not observed). **^{11}B NMR** ($CDCl_3$, 160 MHz) δ_B 32.7. **HRMS** (ESI-TOF) [3M-H]$^-$ calc. for $C_{30}H_{32}B_3O_9^-$ 569.2325 m/z; Found 569.2328 m/z. Δ 0.5270 ppm.

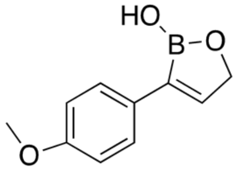

3-(3-methoxyphenyl)-1,2-oxaborol-2(5H)-ol (***6o***): 91.3 mg, 0.480 mmol, 78%, white solid. **^{1}H NMR** (400 MHz, $CDCl_3$) δ_H 7.42 (s, 1H), 7.26-6.85 (m, 3H), 6.84 (d, J = 7.5 Hz, 1H), 6.10 (s, 1H), 4.74 – 4.70 (d, J = 2.0 Hz, 2H), 3.85 (s, 3H). **^{13}C NMR** ($CDCl_3$, 100 MHz) δ_C 159.9, 147.8, 137.3, 129.7, 119.5, 113.2, 112.5, 71.7, 55.3, (*C*-B not observed). **^{11}B NMR** ($CDCl_3$, 160 MHz) δ_B 32.5. **HRMS** (ESI-TOF) [M-H]$^-$ calc. for $C_{10}H_{10}BO_3^-$ 189.0723 m/z; Found 189.0715 m/z. Δ -4.2312 ppm.

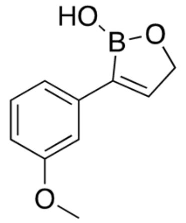

3-(2-methoxyphenyl)-1,2-oxaborol-2(5H)-ol (***6p***)*:* 17.0 mg, 0.895 mmol, 43%, white solid **^{1}H NMR** (500 MHz, $CDCl_3$) δ_H 7.52 (dd, J = 7.6, 1.8 Hz, 1H), 7.47 (s, 1H), 7.31 – 7.27 (m, 1H), 7.01 (td, J = 7.5, 1.1 Hz, 1H), 6.96 (d, J = 8.4 Hz, 1H), 6.03 (s, 1H), 4.72 (d, J = 1.7 Hz, 2H), 3.92 (s, 3H). **^{13}C NMR** (126 MHz, $CDCl_3$) δ_C 156.3, 150.7, 129.1, 129.0 125.6, 122.0, 112.1, 72.3, 56.3. (*C*-B not observed). **^{11}B NMR** (128 MHz $CDCl_3$) δ_B 32.9 **HRMS**: (ESI-TOF) [M-H]$^-$ calc. for $C_{10}H_{10}BO_3^-$ 189.0723 m/z; Found 189.0718 m/z. Δ -2.6445 ppm.

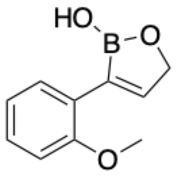

3-(naphthalen-2-yl)-1,2-oxaborol-2(5H)-ol (***6q***)*:* 34.2 mg, 0.163 mmol, 55%, white solid. **^{1}H NMR** (400 MHz, $CDCl_3$) δ_H 8.13 (s, 1H), 7.89 – 7.85 (m, 1H), 7.88-7.80 (m, 2H), 7.74 (dd, J = 8.5, 1.7 Hz, 1H), 7.56 (s, 1H), 7.49 – 7.43 (m, 2H), 4.86 (s, 1H), 4.74 (d, J = 1.6 Hz, 2H). **^{13}C NMR** ($CDCl_3$, 100 MHz) δ_C 148.2, 133.8, 133.3, 133.0, 128.4, 128.3, 127.8, 126.3, 126.3, 126.0, 124.8, 71.9, (*C*-B not observed). **^{11}B NMR** ($CDCl_3$, 128 MHz) δ_B 32.8. **HRMS** (ESI-TOF) [M-H]$^-$ calc. for $C_{13}H_{10}BO_2^-$ 209.0774 m/z; Found 209.0781 m/z. Δ 3.3480 ppm.

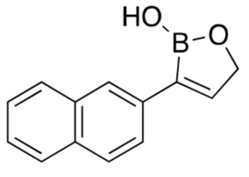

3-(benzofuran-5-yl)-1,2-oxaborol-2(5H)-ol (***6r***)*:* 44.5 mg, 0.223 mmol, 73%, white solid. **^{1}H NMR** (400 MHz, $CDCl_3$) δ_H 7.90 (d, J = 1.3 Hz, 1H), 7.61 (d, J = 2.2 Hz, 1H), 7.58 (d, J = 1.6 Hz, 1H), 7.48 (d, J = 8.6 Hz, 1H), 7.42 (s, 1H), 6.78 (d, J = 1.7 Hz, 1H), 5.29 (s, 1H), 4.72 (d, J = 1.5 Hz, 2H). **^{13}C NMR** (100 MHz, $CDCl_3$) δ_C 154.7, 146.8, 145.5, 131.0, 127.9, 123.4, 119.8, 111.5, 107.0, 71.9, (*C*-B not observed). **^{11}B NMR** ($CDCl_3$, 128 MHz) δ_B 32.7. **HRMS** (ESI-TOF) [M-H]$^-$ calc. for $C_{11}H_8BO_3^-$ 199.0566 m/z; Found 199.0559 m/z. Δ -3.5166 ppm.

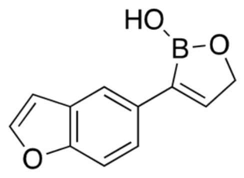

3-(thiophen-2-yl)-1,2-oxaborol-2(5H)-ol (***6s***)*:* 49.7 mg, 0.300 mmol, 71% , white solid. **^{1}H NMR** (400 MHz, $CDCl_3$) δ_H 7.34 (d, J = 3.3 Hz, 1H), 7.26 – 7.21 (m, 2H), 7.02 (dd, J = 5.1, 3.6 Hz, 1H), 5.86 (s, 1H), 4.71 (d, J = 1.4 Hz, 2H). **^{13}C NMR** ($CDCl_3$, 100 MHz) δ_C 145.4, 139.5, 127.8, 126.3, 125.0, 72.0, (*C*-B not observed). **^{11}B NMR** ($CDCl_3$, 128 MHz) δ_B 32.0. **HRMS** (ESI-TOF) [M-H]$^-$ calc. for $C_7H_6BO_2S^-$ 165.0182 m/z; Found 165.0174 m/z. Δ -4.8480 ppm.

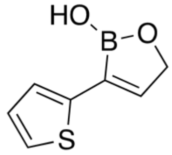

3-(p-tolyl)-1,2-oxaborol-2(5H)-ol (***6t***)*:* 604.4 mg, 3.472 mmol, 70%, white solid. **^{1}H NMR** (400 MHz, $CDCl_3$) δ_H 7.53 (d, J = 8.0 Hz, 2H), 7.38 (s, 1H), 7.17 (d, J = 8.0 Hz, 2H), 5.32 (s, 1H), 4.69 (d, J = 2.0 Hz, 2H), 2.35 (s, 3H). **^{13}C NMR** (101 MHz, $CDCl_3$) δ_C 146.8, 137.2, 132.9, 129.3, 126.7, 71.6, 21.2, (*C*-B not observed). **^{11}B NMR** (128MHz, $CDCl_3$) δ_B 32.7. **HRMS** (ESI-TOF) [M-H]$^-$ calc. for $C_{10}H_{10}BO_2^-$ 173.0774 m/z; Found 173.0779 m/z. Δ 2.8889 ppm.

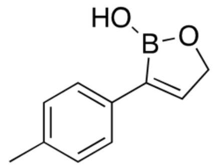

*3-(m-tolyl)-1,2-oxaborol-2(5H)-ol (**6u**):* 572.2 mg, 3.29 mmol, 66%, white solid. **^{1}H NMR** (400 MHz, $CDCl_3$) δ_H 7.48 – 7.46 (m, 2H), 7.41 (s, 1H), 7.29 - 7.28 (m, 1H), 7.27 (t, J = 7.5 Hz, 1H), 7.11 (d, J = 7.4 Hz, 1H), 4.73 (d, J = 1.4 Hz, 2H), 2.40 (s, 3H). **^{13}C NMR** ($CDCl_3$, 100 MHz) δ_C 147.8, 138.3, 135.9, 128.6, 128.4, 127.6, 124.0, 71.8, 21.6, (*C*-B not observed). **^{11}B NMR** ($CDCl_3$, 128 MHz) δ 32.6. **HRMS** (ESI-TOF) [M-H]$^-$ calc. for $C_{10}H_{10}BO_2^-$ 173.0774 m/z; Found 173.0772 m/z. Δ -1.1556 ppm.

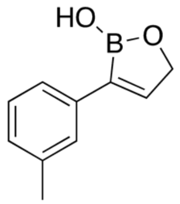

*3-(o-tolyl)-1,2-oxaborol-2(5H)-ol (**6v**):* 34.2 mg, 0.197 mmol, 79%, white solid **^{1}H NMR** (500 MHz, $CDCl_3$) δ_H 7.21 – 7.14 (m, 5H), 4.71 (d, *J* = 1.5 Hz, 2H), 4.57 (s, 1H), 2.35 (s, 3H).**^{13}C NMR** (126 MHz, $CDCl_3$) δ_C 151.6, 136.6, 135.2, 130.2, 128.3, 127.0, 125.8, 71.9, 20.4, (*C*-B not observed). **^{11}B NMR** (128 MHz, $CDCl_3$) δ_B 32.5. **HRMS**: (ESI) [M-H]$^-$ calc. for $C_{10}H_{10}BO_2^-$ 173.0779 m/z; Found 173.0816 m/z. Δ -1.1556 ppm.

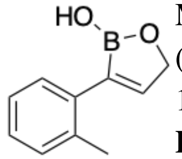

*3-(4-(tert-butyl)phenyl)-1,2-oxaborol-2(5H)-ol (**6w**):* 41.4 mg, 0.191 mmol, 66%, **w**hite solid. **^{1}H NMR** (400 MHz, $CDCl_3$) δ_H 7.64 (d, *J* = 8.3 Hz, 2H), 7.43 (d, *J* = 8.4 Hz, 2H), 7.40 (s, 1H), 6.68 (s, 1H), 4.76 – 4.73 (m, 2H), 1.37 (s, 9H). **^{13}C NMR** ($CDCl_3$, 100 MHz) δ_C 150.6, 146.5, 133.1, 126.7, 125.6, 71.8, 34.7, 31.4, (*C*-B not observed). **^{11}B NMR** ($CDCl_3$, 128 MHz) δ_B 32.7. **HRMS** (ESI-TOF) [M-H]$^-$ calc. for $C_{13}H_{16}BO_2^-$ 215.1243 m/z; Found 215.1234 m/z. Δ -4.1836 ppm.

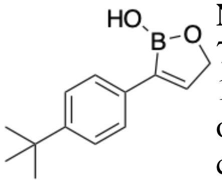

*3-(4-propylphenyl)-1,2-oxaborol-2(5H)-ol (**6x**):* 48.3 mg, 0.239 mmol, 79%, white solid. **^{1}H NMR** (400 MHz, $CDCl_3$) δ_H 7.56 (d, *J* = 8.1 Hz, 2H), 7.38 (s, 1H), 7.18 (d, *J* = 8.1 Hz, 2H), 5.88 (s, 1H), 4.71 (d, *J* = 1.4 Hz, 2H), 2.62 – 2.56 (m, 2H), 1.71 – 1.58 (m, 2H), 0.95 (t, *J* = 7.3 Hz, 3H). **^{13}C NMR** ($CDCl_3$, 100 MHz) δ_C 146.7, 142.2, 133.4, 128.9, 126.8, 71.8, 37.9, 24.7, 13.9, (*C*-B not observed). **^{11}B NMR** ($CDCl_3$, 128 MHz) δ_B 32.7. **HRMS** (ESI-TOF) [M-H]$^-$ calc. for $C_{12}H_{14}BO_2^-$ 201.1087 m/z; Found 201.1087 m/z. Δ -4.1836 ppm.

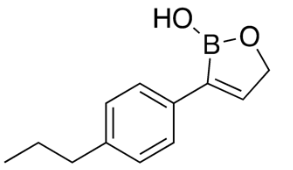

*3-(cyclohex-1-en-1-yl)-1,2-oxaborol-2(5H)-ol (**6y**):* 623.1 mg, 3.80 mmol, 74%, white solid. **^{1}H NMR** (400 MHz, $CDCl_3$) δ_H 6.86 (s, 1H), 6.36 (s, 1H), 6.04 (s, 1H), 4.58 (s, 2H), 2.24 – 2.13 (m, 4H), 1.75 – 1.66 (m, 2H), 1.64 – 1.56 (m, 2H). **^{13}C NMR** ($CDCl_3$, 100 MHz) δ_C 143.1, 133.5, 128.8, 71.5, 26.2, 25.9, 22.8, 22.4, (*C*-B not observed). **^{11}B NMR** ($CDCl_3$, 128 MHz) δ_B 32.5. **HRMS** (ESI-TOF) [M-H]$^-$ calc. for $C_9H_{12}BO_2^-$ 163.0930 m/z; Found 163.0936 m/z. Δ 3.6789 ppm.

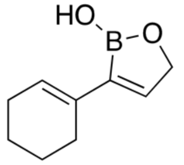

methyl 3-(3-fluoro-5-methylphenyl)propiolate (***9k***) - 17.4 mg, 0.091 mmol, 21%, yellow oil - **^{1}H NMR** (500 MHz, $CDCl_3$) δ_H δ 7.19 (s, 1H), 7.07 (d, *J* = 8.7 Hz, 1H), 6.97 (d, *J* = 9.5 Hz, 1H), 3.84 (s, 3H), 2.35 (s, 3H). **^{13}C NMR** ($CDCl_3$, 126 MHz) δ_C 162.2 (d, *J* = 247.2 Hz), 154.2, 141.0 (d, *J* = 8.3 Hz), 129.5 (d, *J* = 2.8 Hz), 120.8 (d, *J* = 10.1 Hz), 118.9 (d, *J* = 21.2 Hz), 116.7 (d, *J* = 23.3 Hz), 85.1 (d, *J* = 3.6 Hz), 80.4, 52.9, 21.1 (d, *J* = 1.9 Hz), **^{19}F NMR** (376 MHz, $CDCl_3$) δ_F -112.94. **HRMS** (ESI-TOF) $[M+H]^+$ calc. for $C_{11}H_{10}FO_2^+$ 193.0665 m/z; Found 193.0656 m/z. Δ -4.6616 ppm.

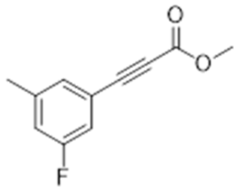

*methyl 3-(3-iodophenyl)propiolate (**9l**)* - 854.0 mg, 2.99 mmol, 49%, yellow solid - **^{1}H NMR** (400 MHz, $CDCl_3$) δ_H 7.96 – 7.90 (m, 1H), 7.81 – 7.76 (m, 1H), 7.57 – 7.52 (m, 1H), 7.12 (d, *J* = 7.9 Hz, 1H), 3.84 (s, 3H). **^{13}C NMR** ($CDCl_3$, 100 MHz) δ_C 154.3, 141.5, 139.8, 132.1, 130.2, 121.7, 93.8, 84.4, 81.4, 53.1. **HRMS** (ESI-TOF) $[M+H]^+$ calc. for $C_{10}H_8IO_2^+$ 286.9569 m/z; Found 286.9570 m/z. Δ 0.3485 ppm.

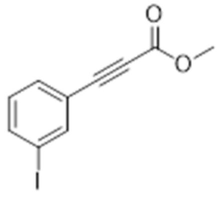

*methyl (Z)-4-(3-fluorophenyl)-3-(4,4,5,5-tetramethyl-1,3,2-dioxaborolan-2-yl)but-3-enoate (**10c**)*: 100.4 mg, 0.328 mmol, 58%, yellow oil (*E*:*Z* - >99:1) - **^{1}H NMR** (400 MHz, $CDCl_3$) δ_H = 7.34 – 7.33 (m, 1H), 7.27 – 7.26 (m, 1H), 7.19 (ddt, *J* = 10.1, 2.7, 1.4 Hz, 1H), 7.04 (tdt, *J* = 8.2, 2.5, 1.3 Hz, 1H), 6.42 (s, 1H), 3.80 (s, 3H), 1.41 (s, 12H). **^{13}C NMR** ($CDCl_3$, 100 MHz) δ_C 168.3, 163.1 (d, *J* = 246.0 Hz), 141.0, 130.4 (d, *J* = 8.4 Hz), 126.8, 123.1, 116.1 (d, *J* = 21.0 Hz), 114.1 (d, *J* = 22.1 Hz), 84.7, 52.2, 25.2, (*C*-B not observed). **^{11}B NMR** ($CDCl_3$, 128 MHz) δ_B δ 30.3. **HRMS** (ESI-TOF) $[M+H]^+$ calc. for $C_{16}H_{21}BFO_4^+$ 307.1517 m/z; Found 307.1519 m/z. Δ 0.6511 ppm.

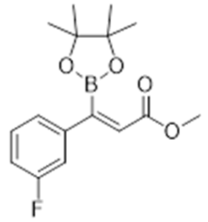

*methyl (E)-3-(3-bromophenyl)-3-(4,4,5,5-tetramethyl-1,3,2-dioxaborolan-2-yl)acrylate (**10i**):* 61.5 mg, 0.168 mmol, 40%, yellow oil (*E*:*Z* - >99:1) - **^{1}H NMR** (500 MHz, $CDCl_3$) δ_H *δ 7.63 (t, J = 1.8 Hz, 1H), 7.47 (ddd, J = 7.9, 2.0, 1.1 Hz, 1H), 7.41 (dt, J = 7.8, 1.4 Hz, 1H), 7.23 (t, J = 7.9 Hz, 1H), 6.41 (s, 1H), 3.80 (s, 3H), 1.41 (s, 12H).* **^{13}C NMR** ($CDCl_3$, 126 MHz) δ_C 168.5, 141.0, 130.6, 130.6, 127.1, 126.1, 123.3, 85.0, 52.5, 25.4, (*C*-B not observed). **^{11}B NMR** ($CDCl_3$, 128 MHz) δ_B 30.2. **HRMS** (ESI-TOF) $[M+H]^+$ calc. for $C_{16}H_{21}BBrO_4^+$ 367.0716 m/z; Found 367.0725 m/z. Δ 2.4518 ppm.

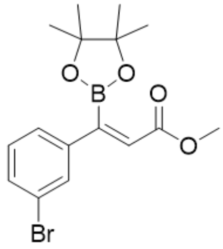

*methyl (E)-3-(2-bromophenyl)-3-(4,4,5,5-tetramethyl-1,3,2-dioxaborolan-2-yl)acrylate (**10j**):*

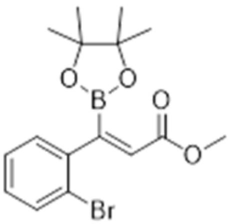

64.0 mg, 0.170 mmol,19%, yellow oil, *(E:Z - 66:34)* - **^{1}H NMR** (400 MHz, $CDCl_3$) δ_H 7.58 (d, *J* = 7.9 Hz, 0.38 H, *minor*), 7.51 – 7.49 (m, 0.57 H, *major*), 7.49 – 7.47 (m, 0.44 H, *minor*) 7.38 – 7.36 (m, 0.56 H, *major*), 7.35 (d, *J* = 5.0, 1H), 7.28 (d, *J* = 4.4 Hz, 0.63 H, *major*), 7.14 (dt, *J* = 7.8, 4.7 Hz, 0.39 H, *minor*), 6.43 (s, 0.61 H, *major*), 6.32 (s, 0.32 H, *minor*), 3.79 (s, 3H), 1.42 (s, 8.16 H, *major*), 1.33 (s, 3.62 H, *minor*). **^{13}C NMR** ($CDCl_3$, 100 MHz) δ_C 168.5, 133.4, 131.2, 129.7, 129.3, 128.9, 127.3, 125.8, 84.6, 52.0, 25.2, (*C*-B not observed). **^{11}B NMR** ($CDCl_3$, 128 MHz) δ_B 30.2. **HRMS** (ESI-TOF) $[M+H]^+$ calc. for $C_{16}H_{21}BBrO_4^+$ 367.0716 m/z; Found 367.0723 m/z. Δ 1.9070 ppm.

*methyl (E)-3-(3-iodophenyl)-3-(4,4,5,5-tetramethyl-1,3,2-dioxaborolan-2-yl)acrylate (**10l**):*

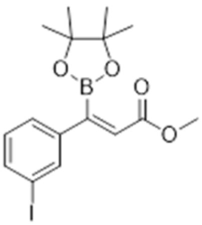

145.5 mg, 0.351 mmol, 50%, yellow oil (*E*:*Z* - >99:1) - **^{1}H NMR** (400 MHz, $CDCl_3$) δ_H 7.84 (t, *J* = 1.5 Hz, 1H), 7.70 – 7.63 (m, 1H), 7.50 – 7.41 (m, 1H), 7.09 (t, *J* = 7.9, 1.3 Hz, 1H), 6.40 (s, 1H), 3.79 (s, 3H), 1.41 (s, 12H). **^{13}C NMR** ($CDCl_3$, 100 MHz) δ_C 168.1, 140.7, 137.8, 136.1, 130.3, 126.6, 126.2, 94.6, 84.6, 52.0, 25.0, (*C*-B not observed).**^{11}B NMR** ($CDCl_3$, 160 MHz) δ_B 30.3. **HRMS** (ESI-TOF) m/z $[M+H]^+$ calc. for $C_{16}H_{21}BIO_4^+$ 415.05780 m/z; Found 415.0592 m/z. Δ 3.3730 ppm.

*methyl (E)-3-(4,4,5,5-tetramethyl-1,3,2-dioxaborolan-2-yl)-3-(o-tolyl)acrylate (**10v**):* 109.5 mg,

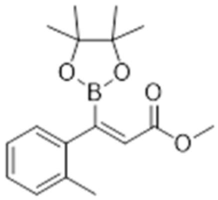

0.362 mmol, 63%, yellow oil (*E*:*Z* - >99:1) - **^{1}H NMR** ($CDCl_3$, 400MHz) δ_H 7.23 – 7.12 (m, 4H), 6.14 (s, 1H), 3.79 (s, 3H), 2.36 (s, 3H), 1.32 (s, 12H). **^{13}C NMR** ($CDCl_3$, 100 MHz) δ_C 168.2, 139.3, 135.1, 130.7, 129.1, 127.9, 127.8, 125.8, 84.4, 52.0, 24.9, 20.6, (*C*-B not observed). **^{11}B NMR** ($CDCl_3$, 128 MHz) δ_B 29.8. **HRMS** (ESI-TOF) m/z $[M+H]^+$ Calcd for $C_{17}H_{24}BO_4^+$ 303.1768 m/z; Found 303.1771 m/z. Δ 0.9895 ppm.

4) NMR SPECTRA

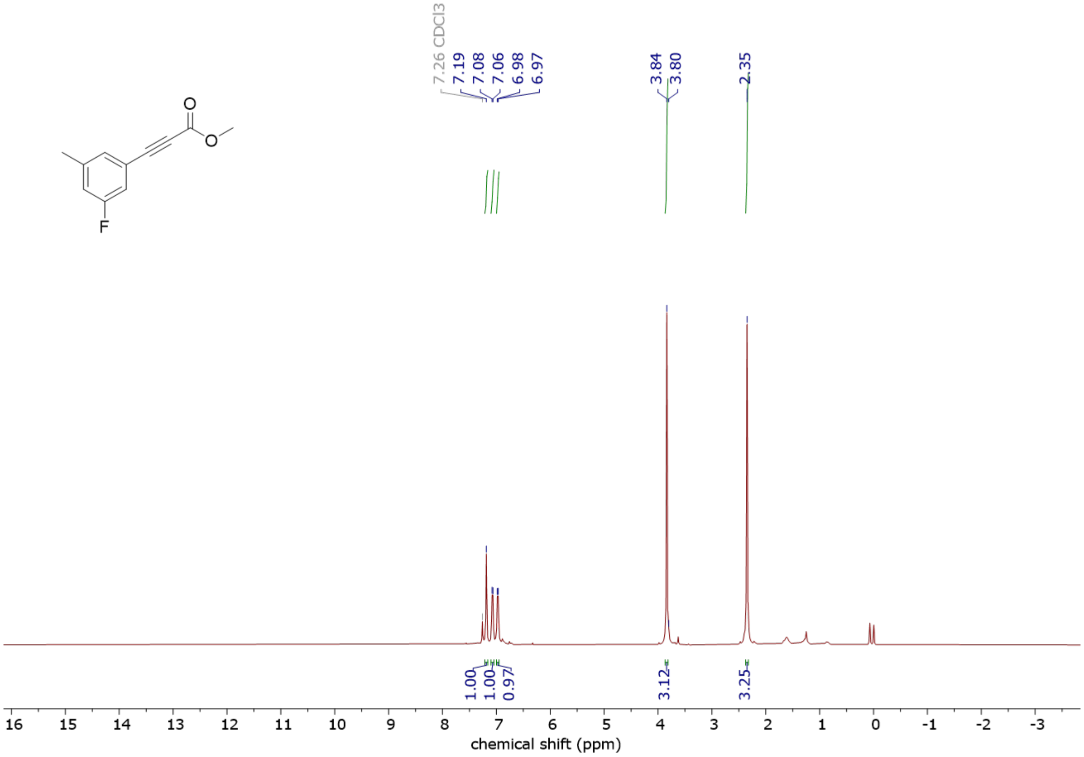

Figure S1. ^{1}H NMR of **9k** ($CDCl_3$, 400 MHz)

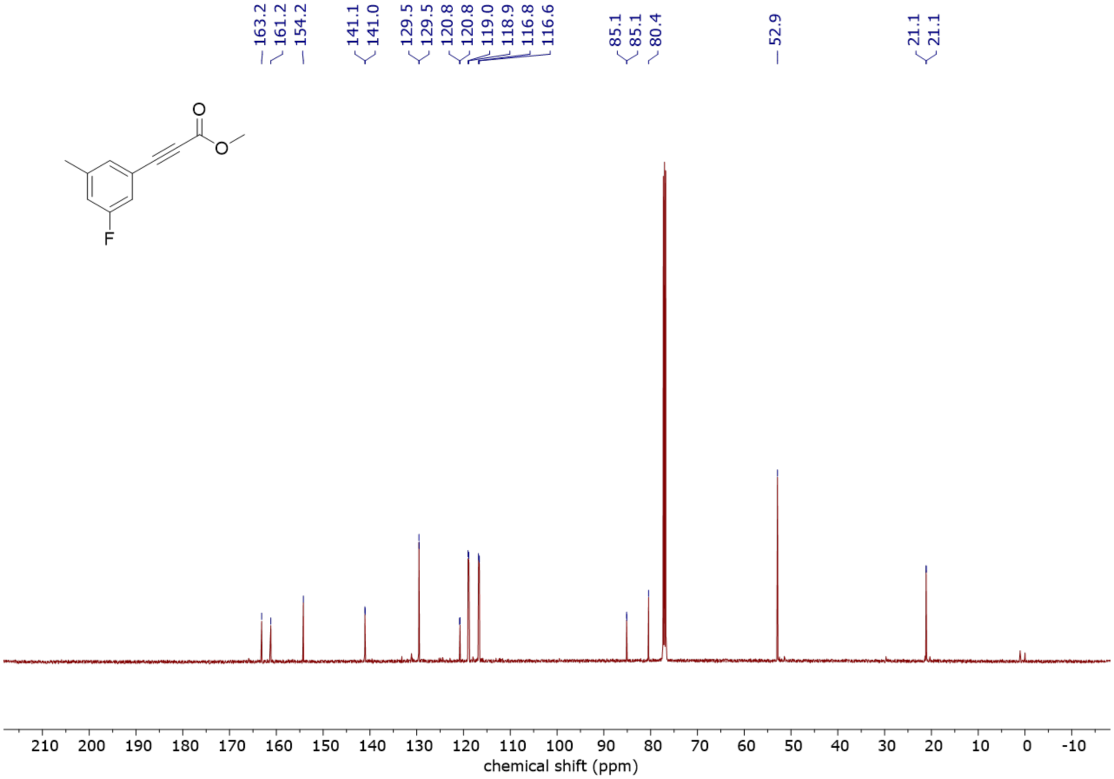

Figure S2. ^{13}C NMR of **9k** ($CDCl_3$, 100 MHz)

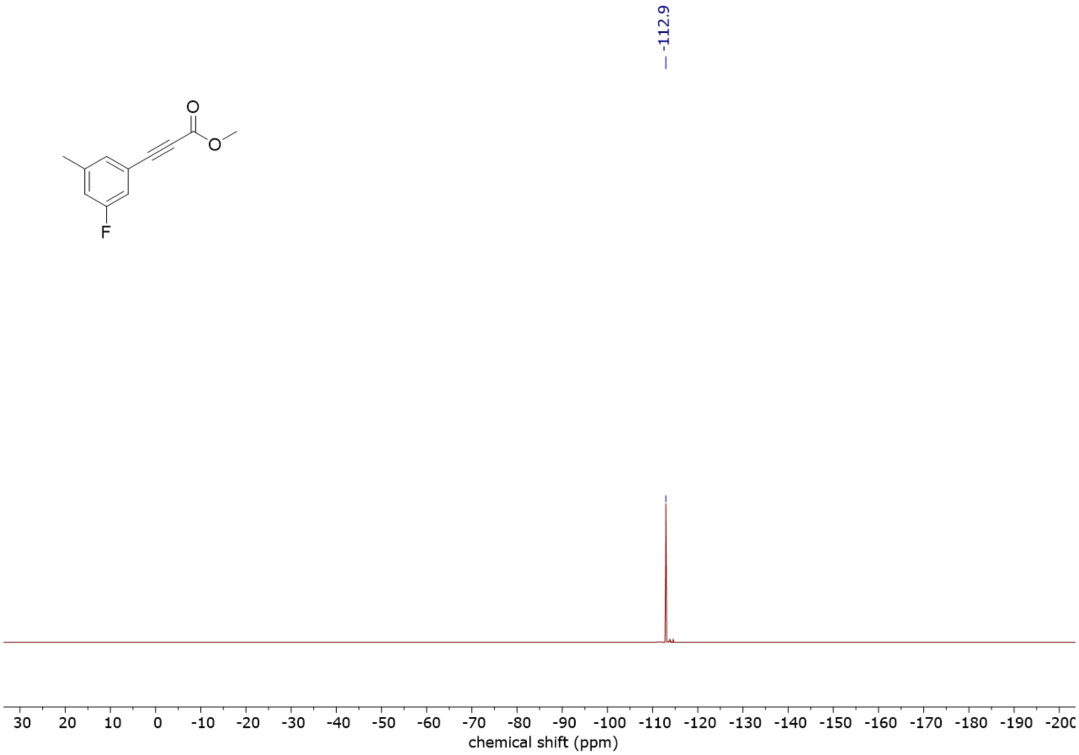

Figure S3. ^{19}F NMR of **9k** ($CDCl_3$, 376 MHz)

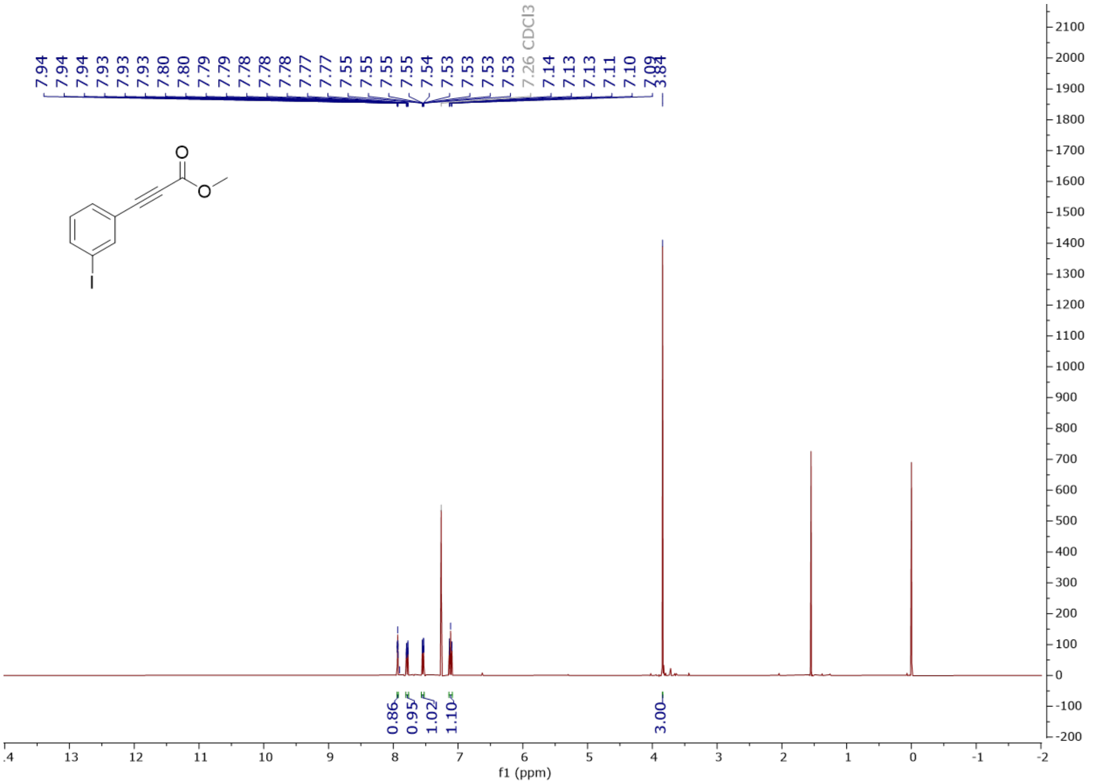

Figure S4. ^{1}H NMR of **9l** ($CDCl_3$, 400 MHz)

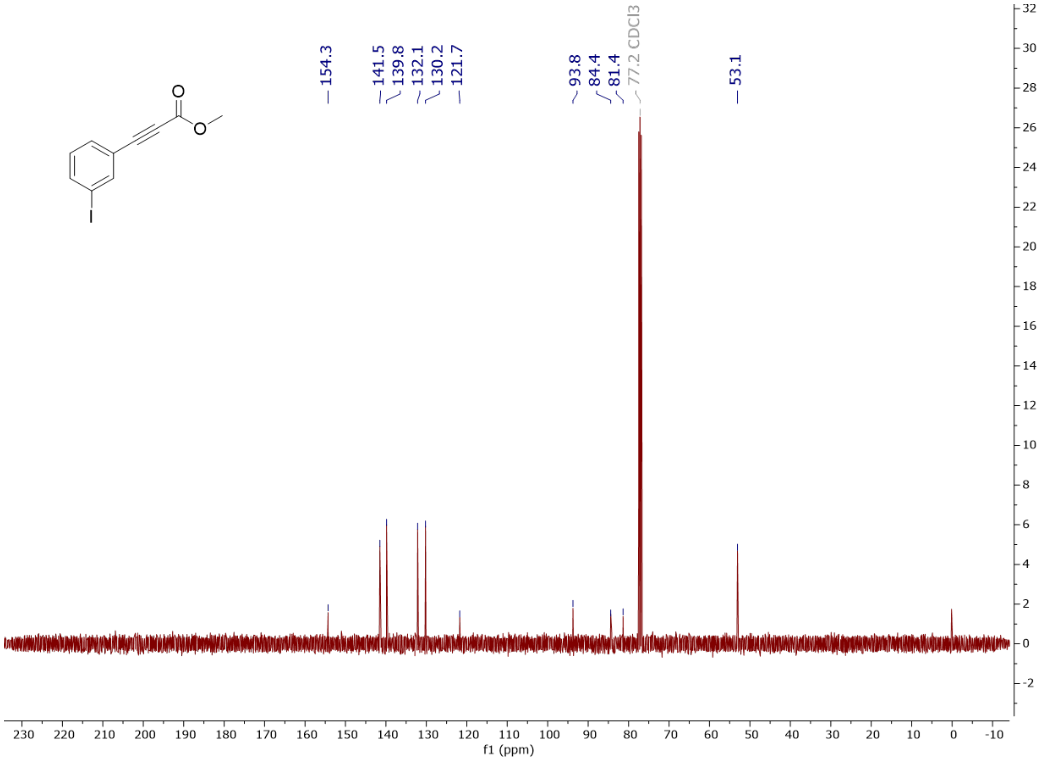

Figure S5. ^{13}C NMR of **9l** ($CDCl_3$, 100 MHz)

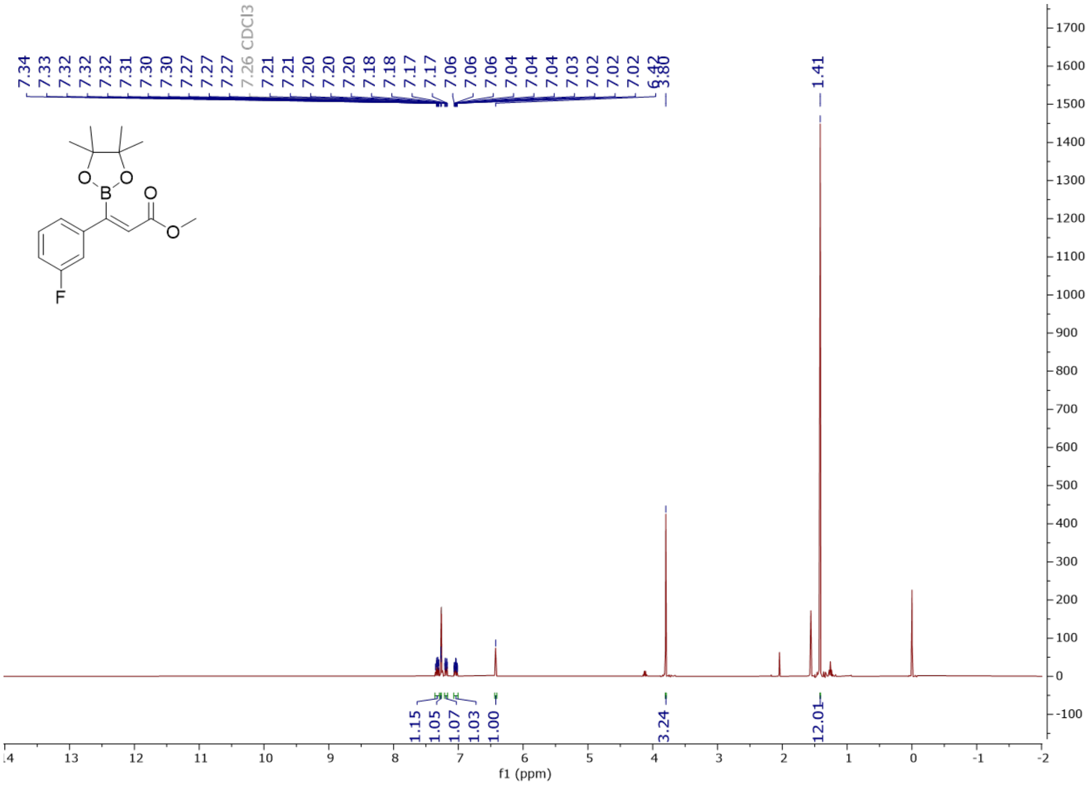

Figure S6. ^{1}H NMR of **10c** ($CDCl_3$, 400 MHz)

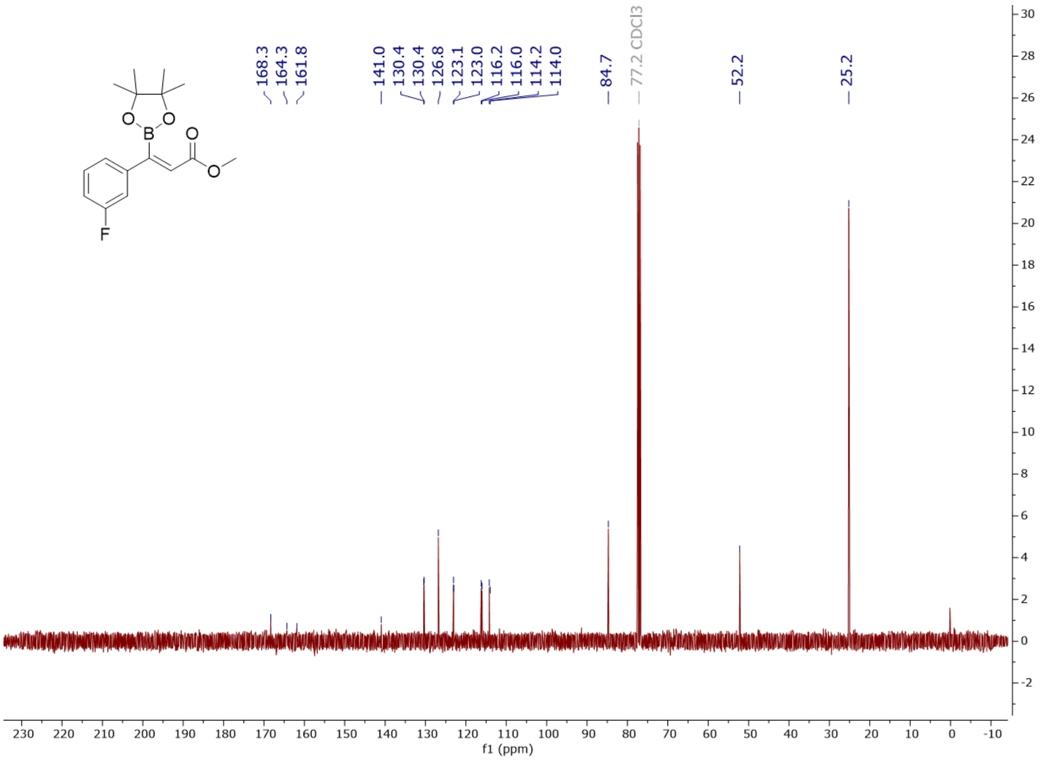

Figure S7. ^{13}C NMR of **10c** ($CDCl_3$, 100 MHz)

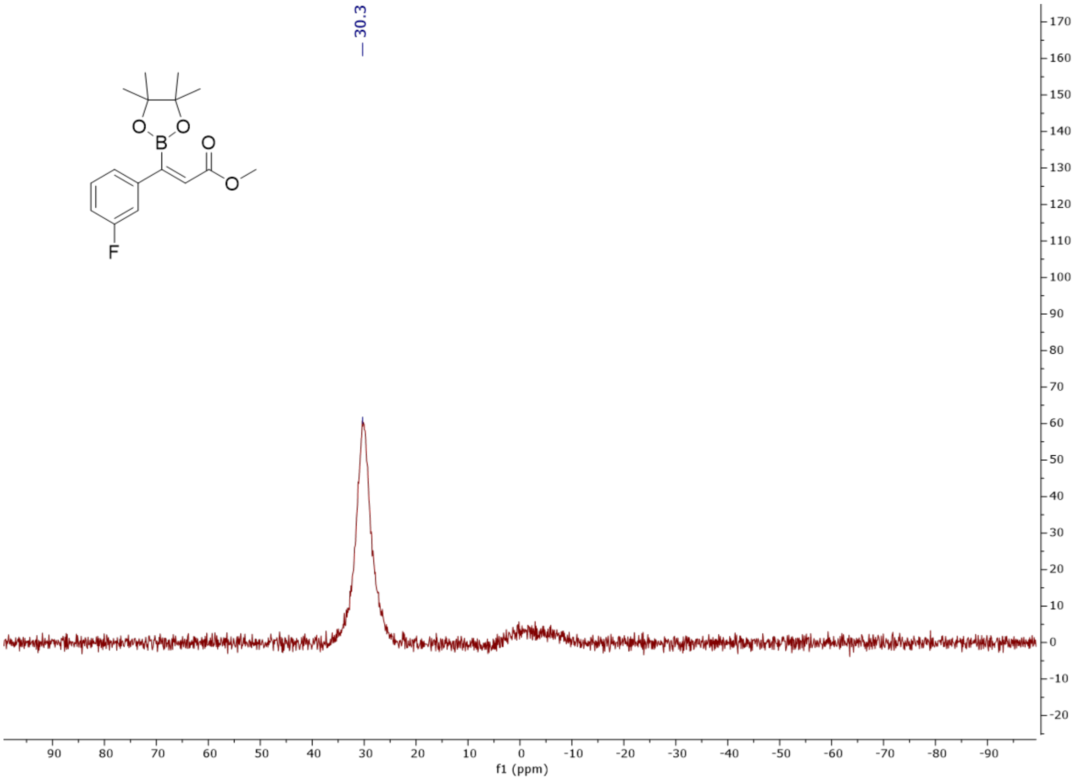

Figure S8. ^{11}B NMR of **10c** ($CDCl_3$, 128 MHz)

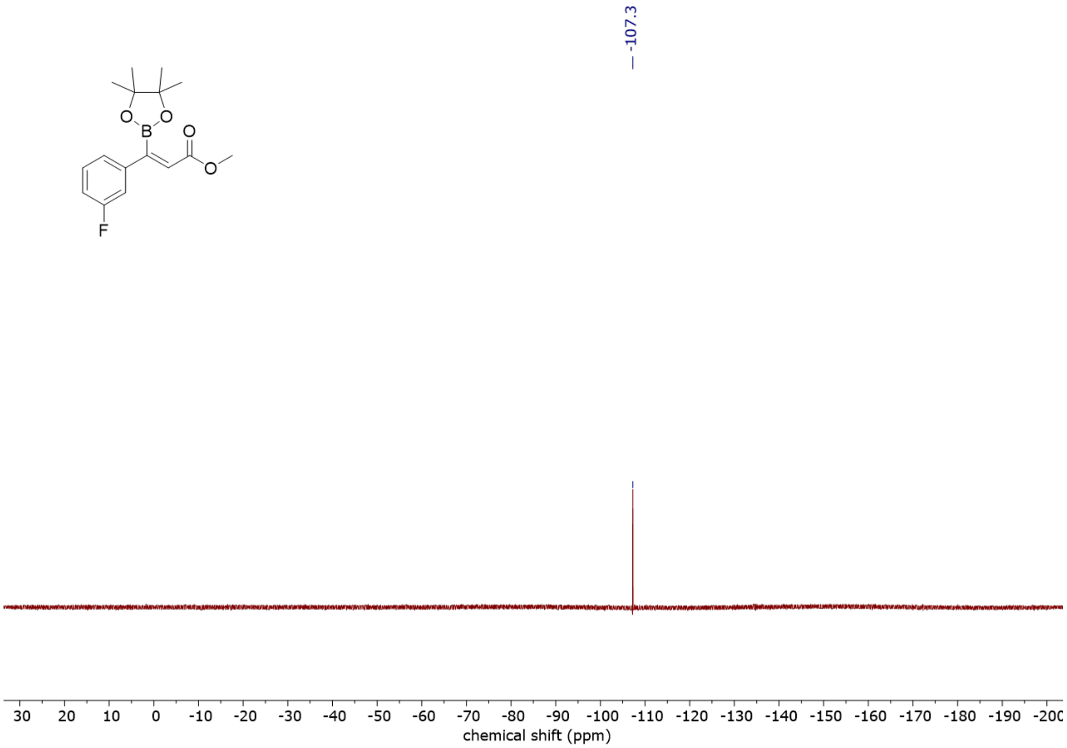

Figure S9. ^{19}F NMR of **10c** ($CDCl_3$, 376 MHz)

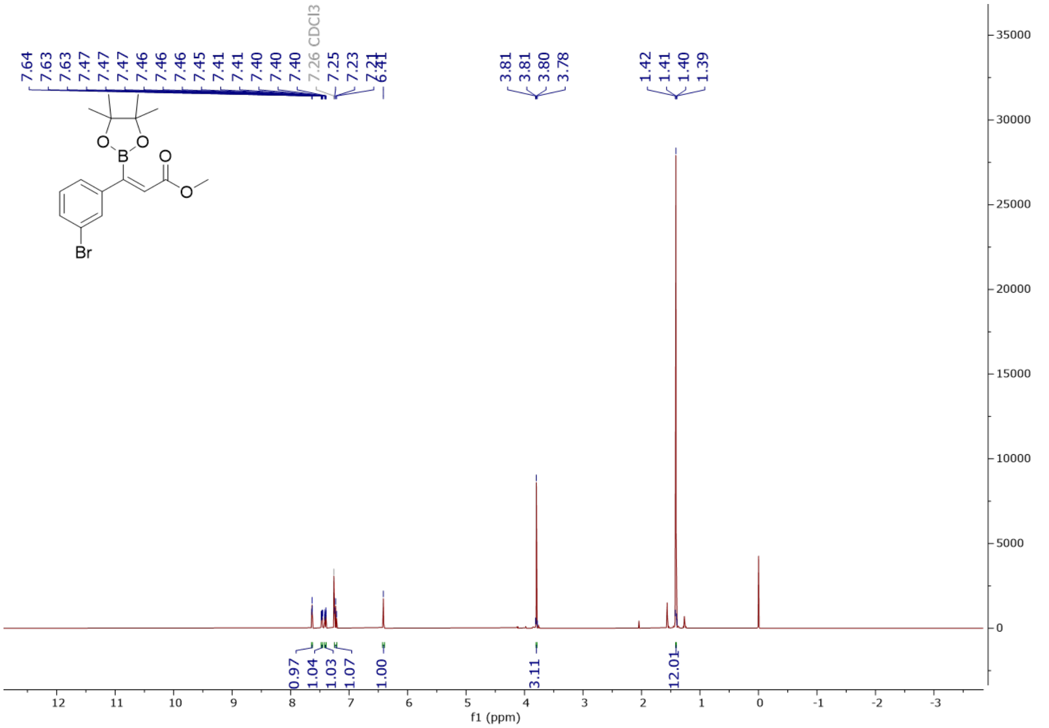

Figure S10. ^{1}H NMR of **10i** ($CDCl_3$, 500 MHz)

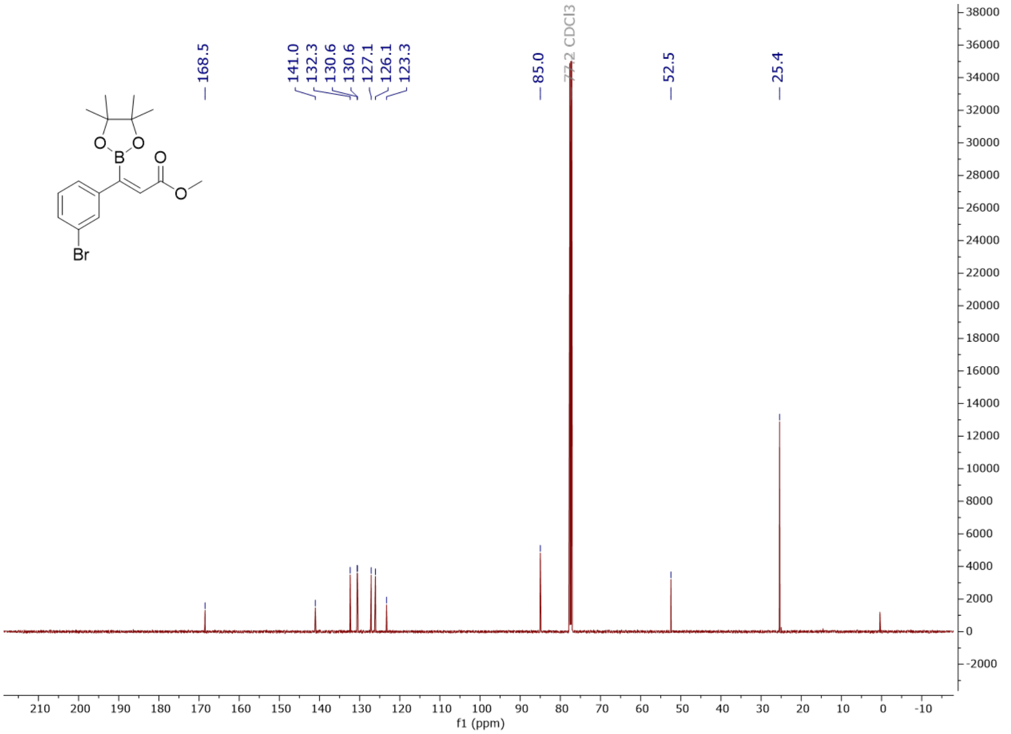

Figure S11. ^{13}C NMR of **10i** ($CDCl_3$, 126 MHz)

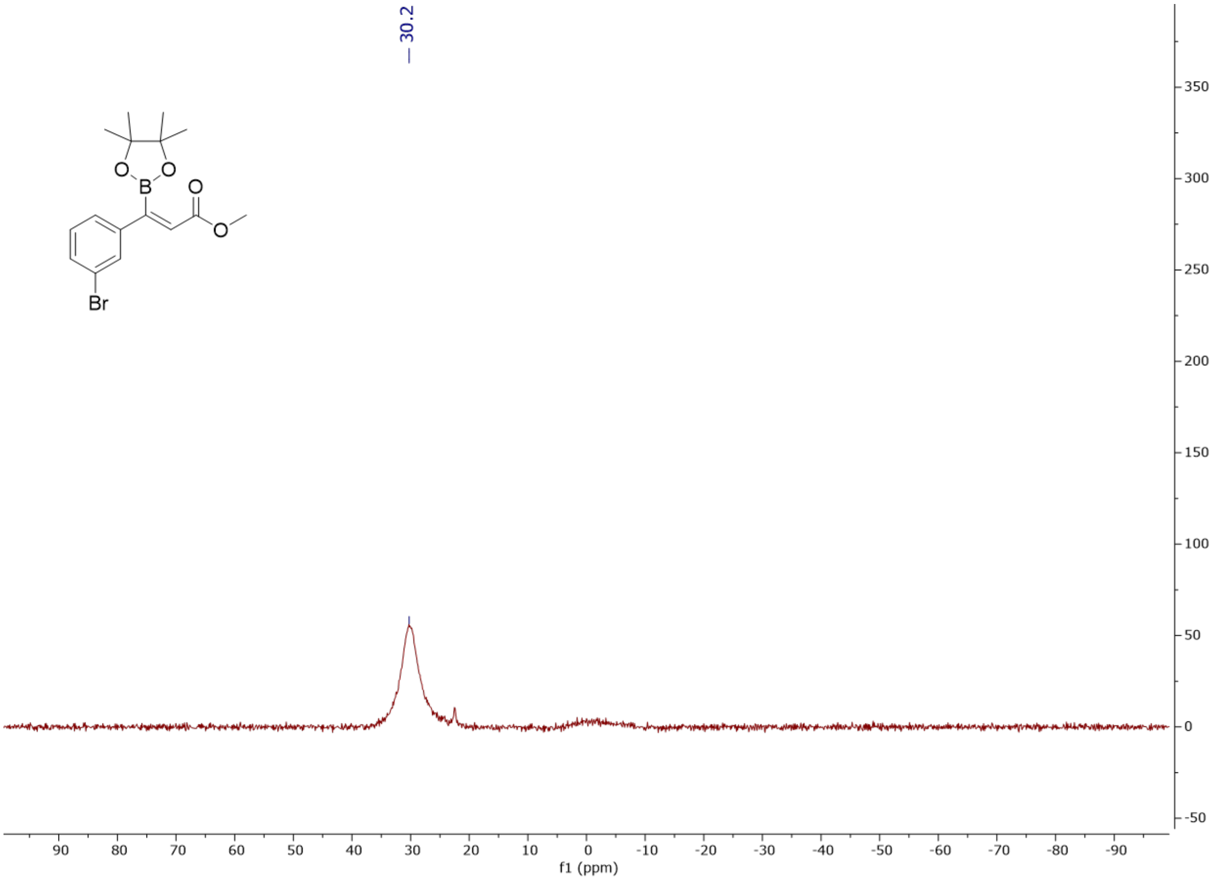

Figure S12. ^{11}B NMR of **10i** ($CDCl_3$, 128 MHz)

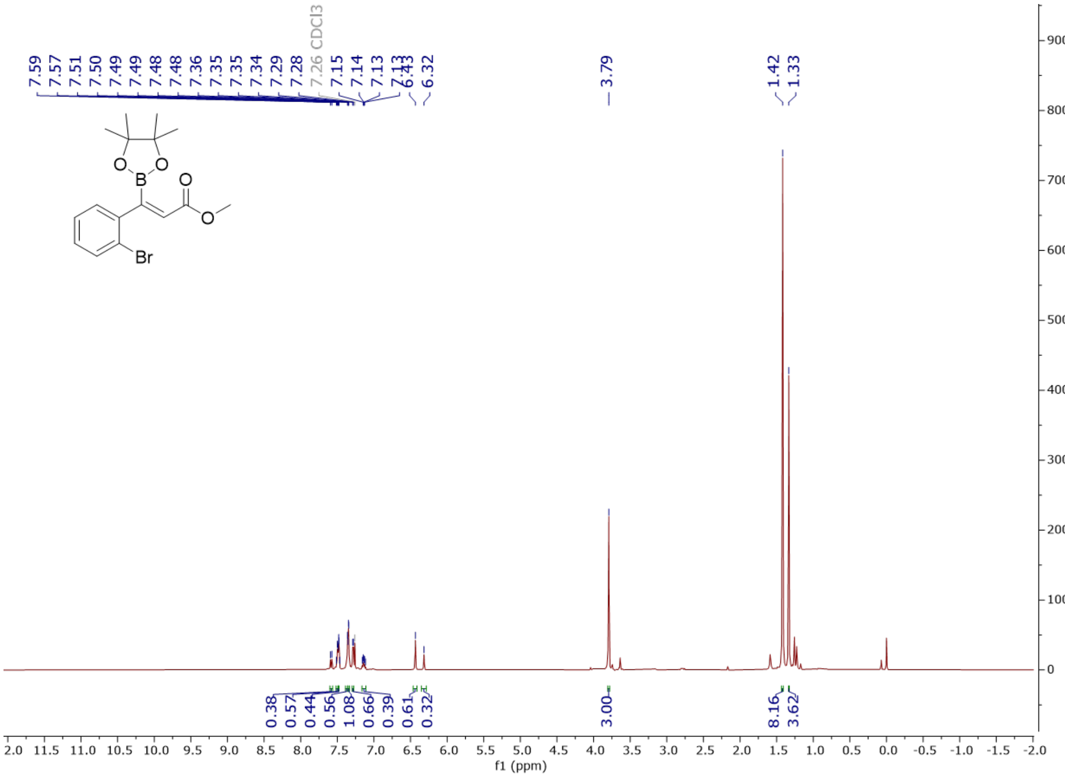

Figure S13. ^{1}H NMR of **10j** ($CDCl_3$, 400 MHz)

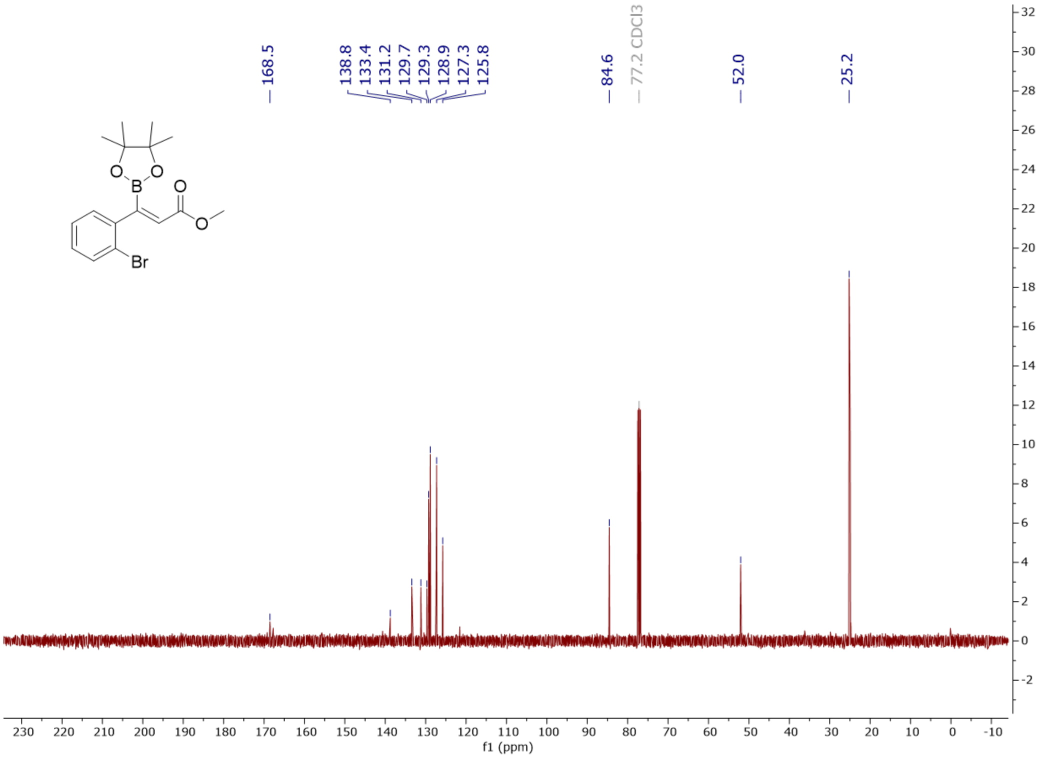

Figure S14. ^{13}C NMR of **10j** ($CDCl_3$, 100 MHz)

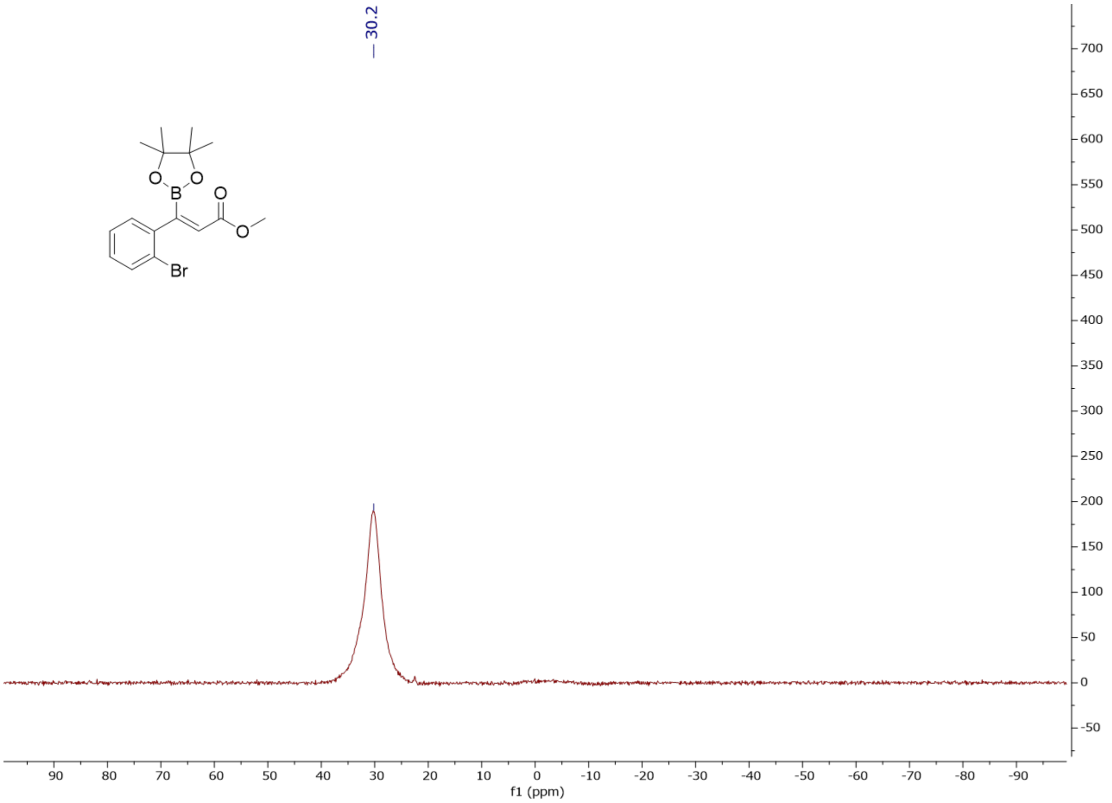

Figure S15. ^{11}B NMR of **10j** ($CDCl_3$, 128 MHz)

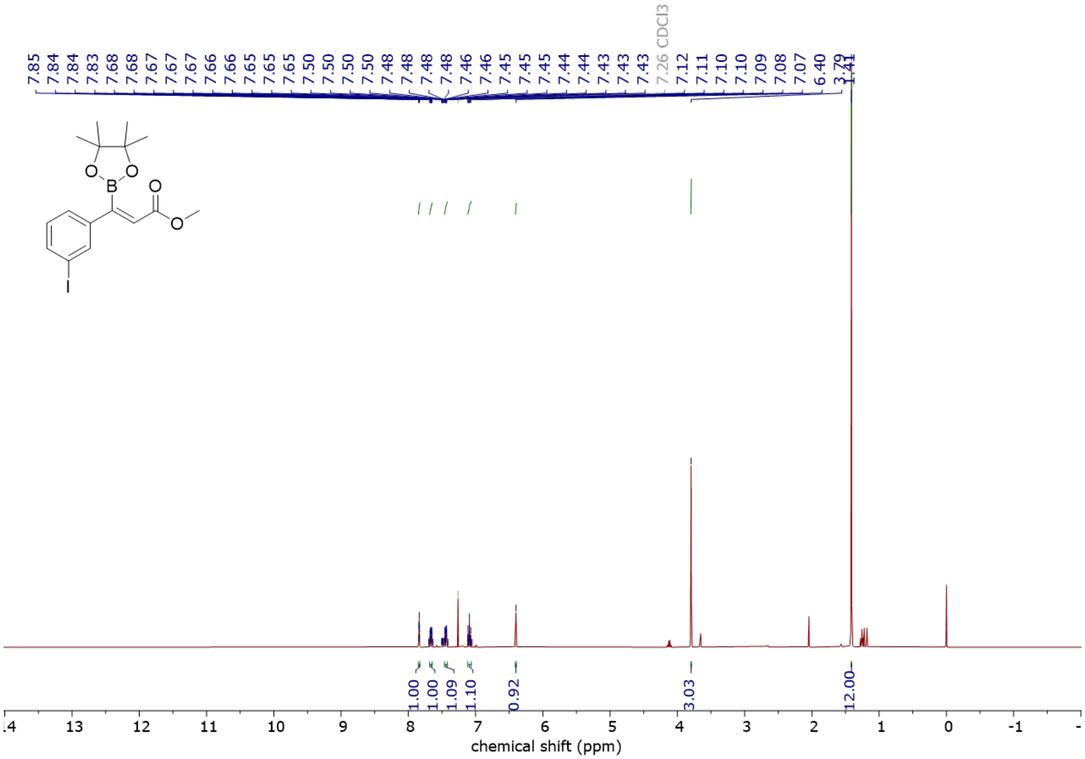

Figure S16. ^{1}H NMR of **10l** ($CDCl_3$, 400 MHz)

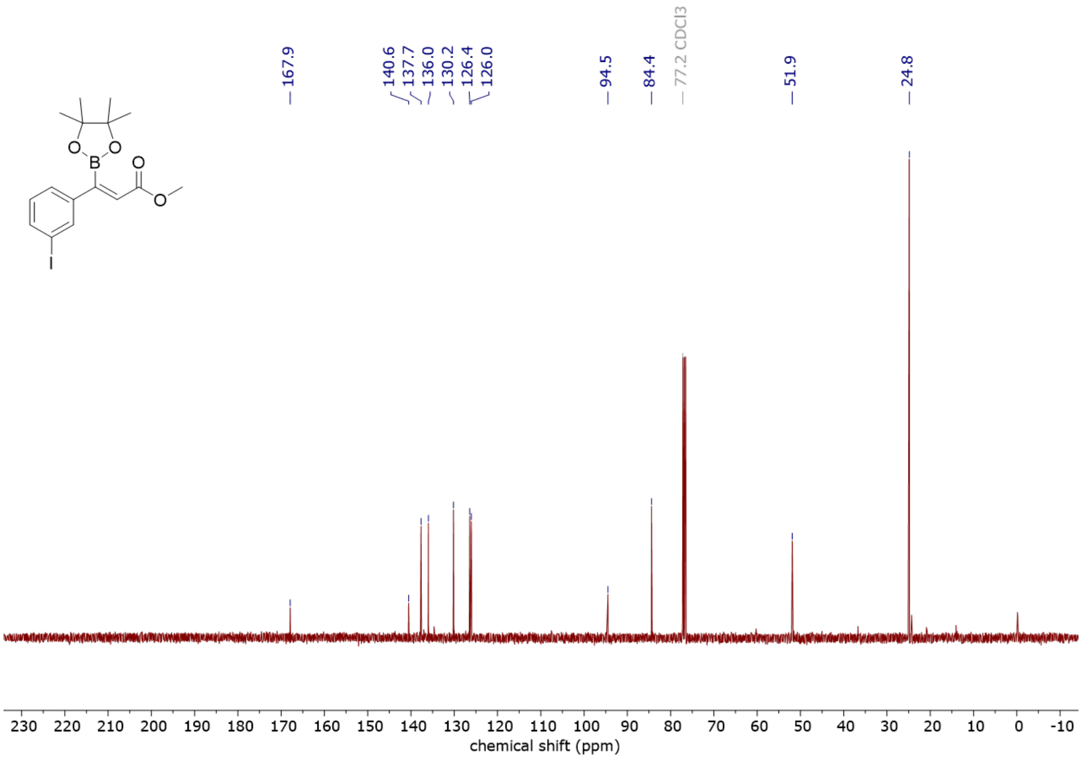

Figure S17. ^{13}C NMR of **10l** ($CDCl_3$, 100 MHz)

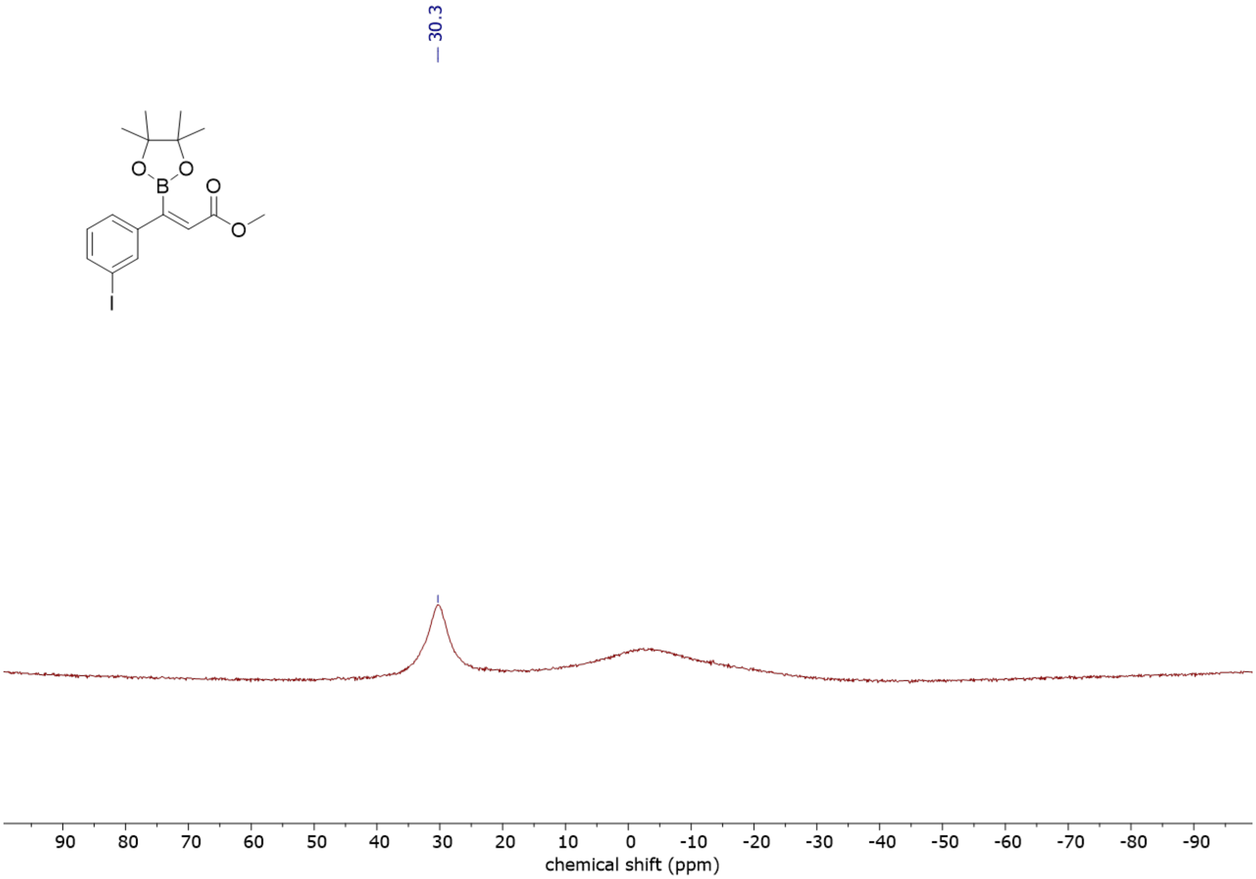

Figure S18. ^{11}B NMR of **10l** ($CDCl_3$, 128 MHz)

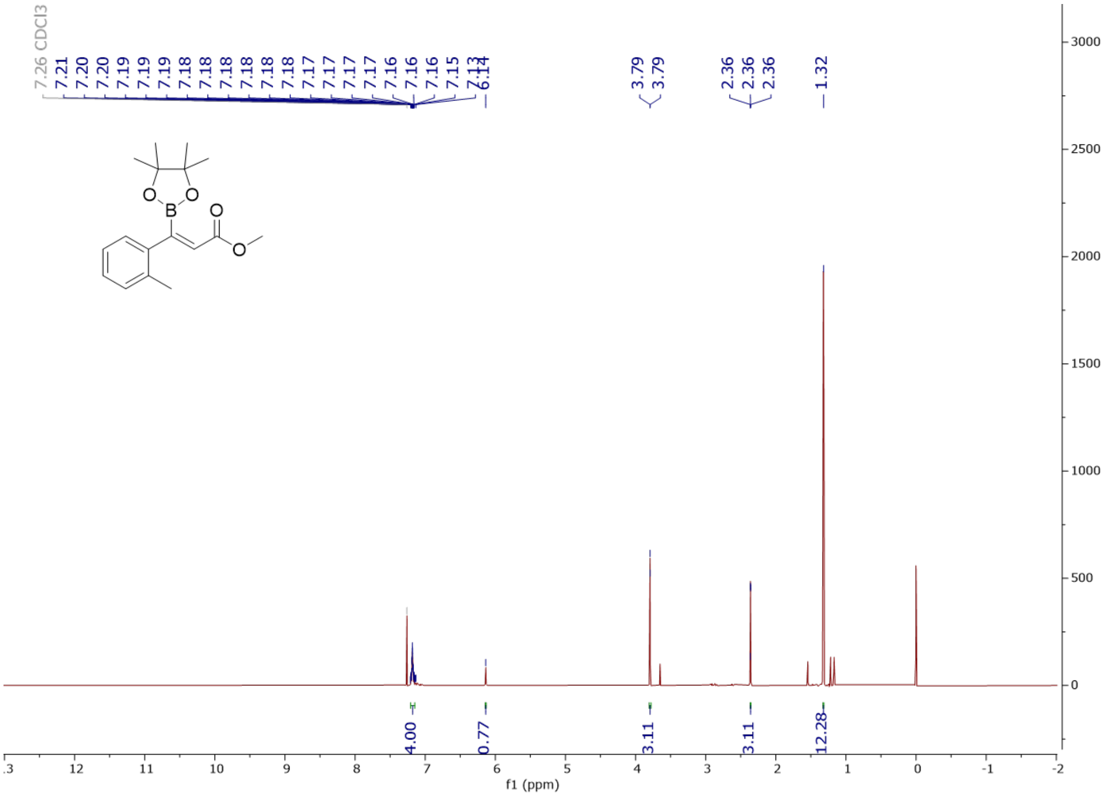

Figure S19. ^{1}H NMR of **10v** ($CDCl_3$, 400 MHz)

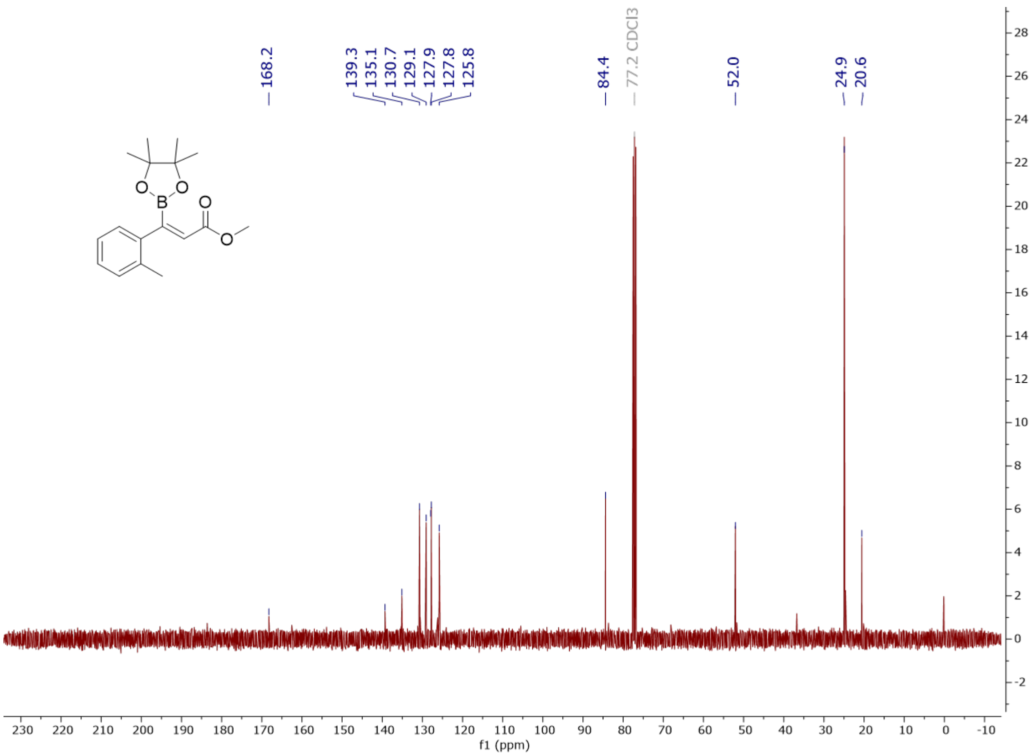

Figure S20. ^{13}C NMR of **10v** ($CDCl_3$, 100 MHz)

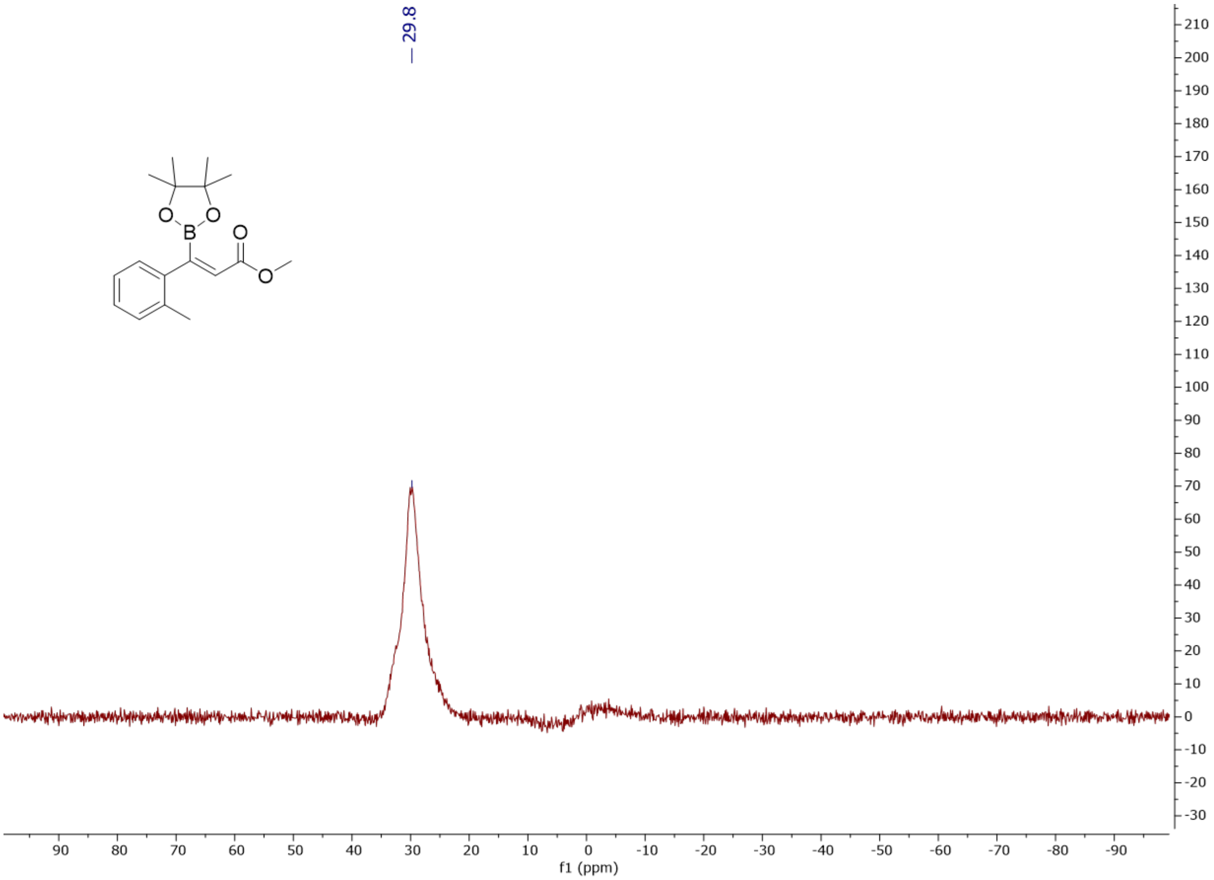

Figure S21. ^{11}B NMR of **10v** ($CDCl_3$, 128 MHz)

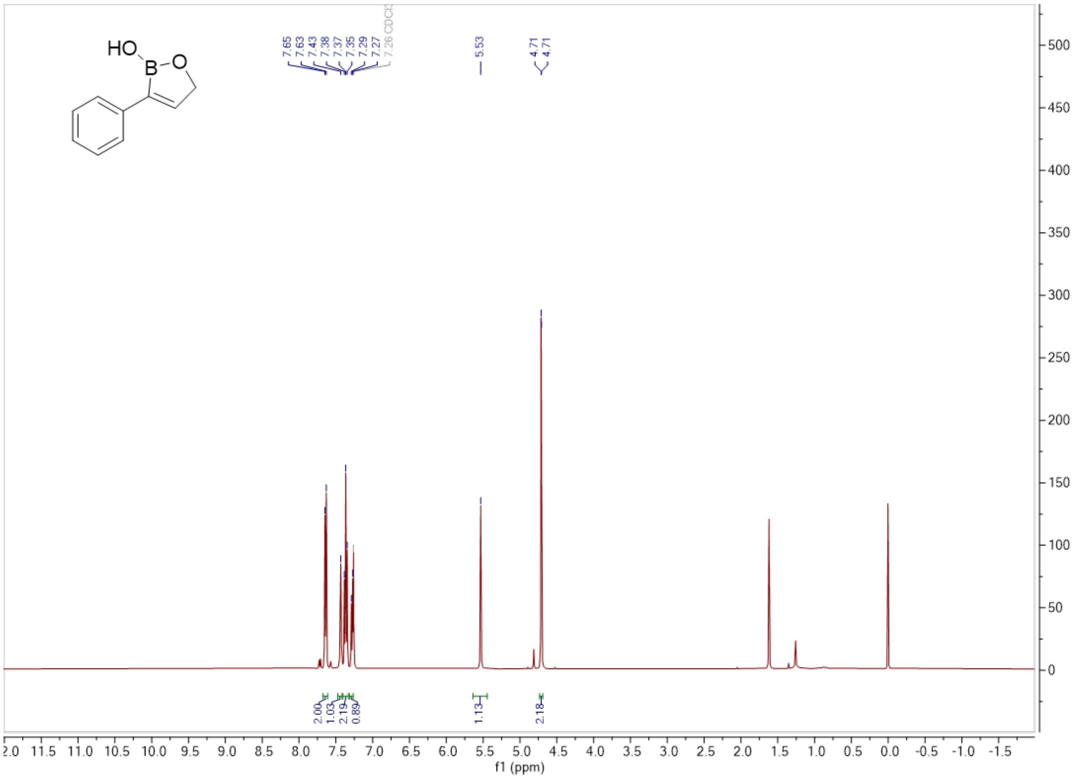

Figure S22. ^{1}H NMR of **6a** ($CDCl_3$, 400 MHz)

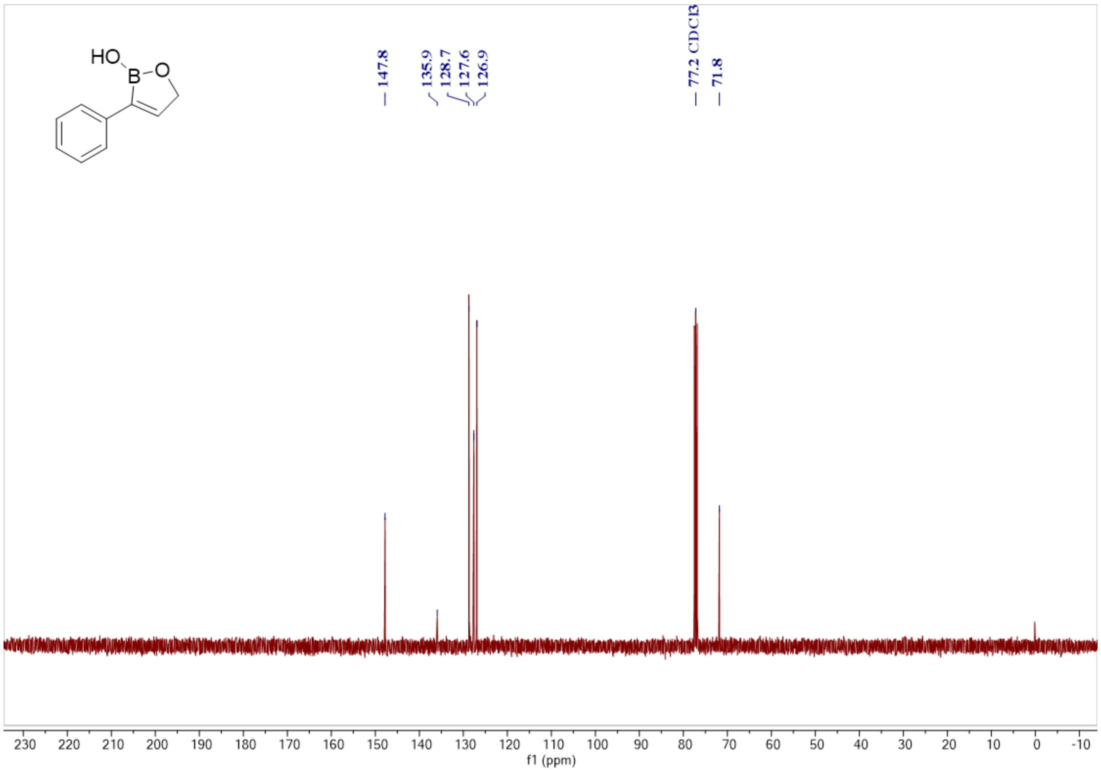

Figure S23. ^{13}C NMR of **6a** ($CDCl_3$, 100 MHz)

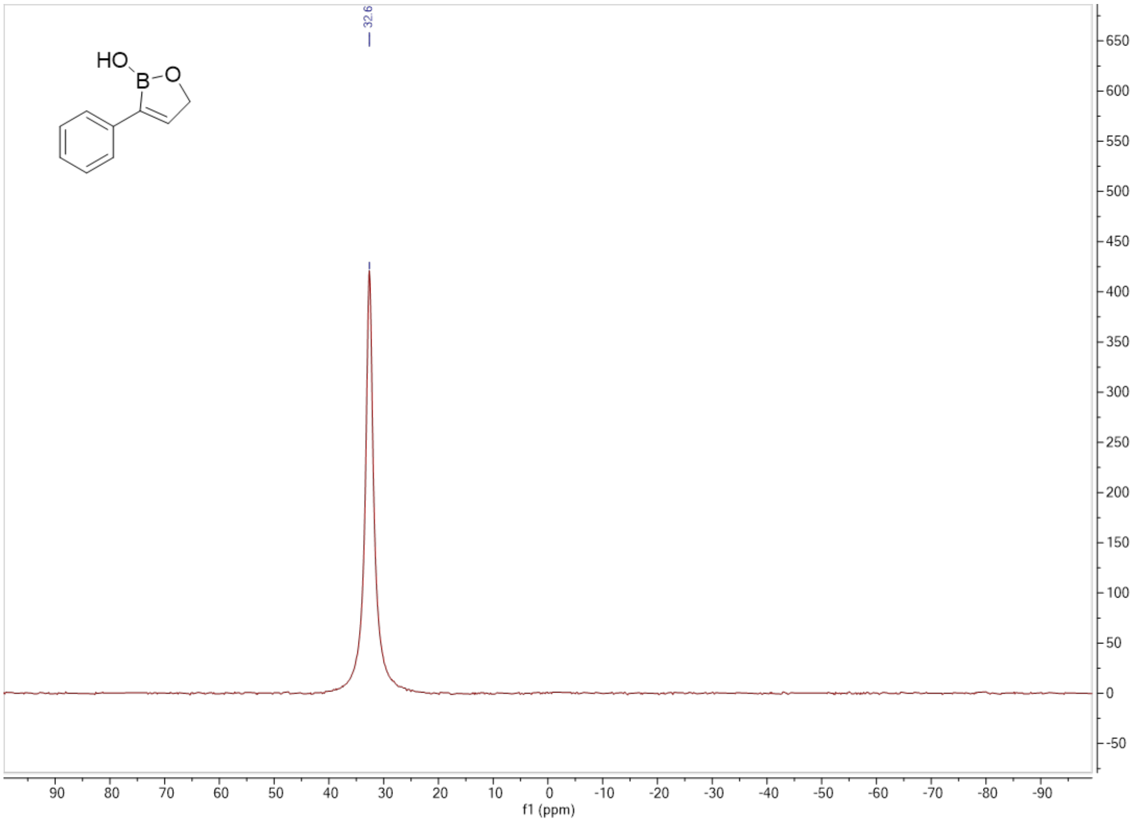

Figure S24. ^{11}B NMR of **6a** ($CDCl_3$, 128 MHz)

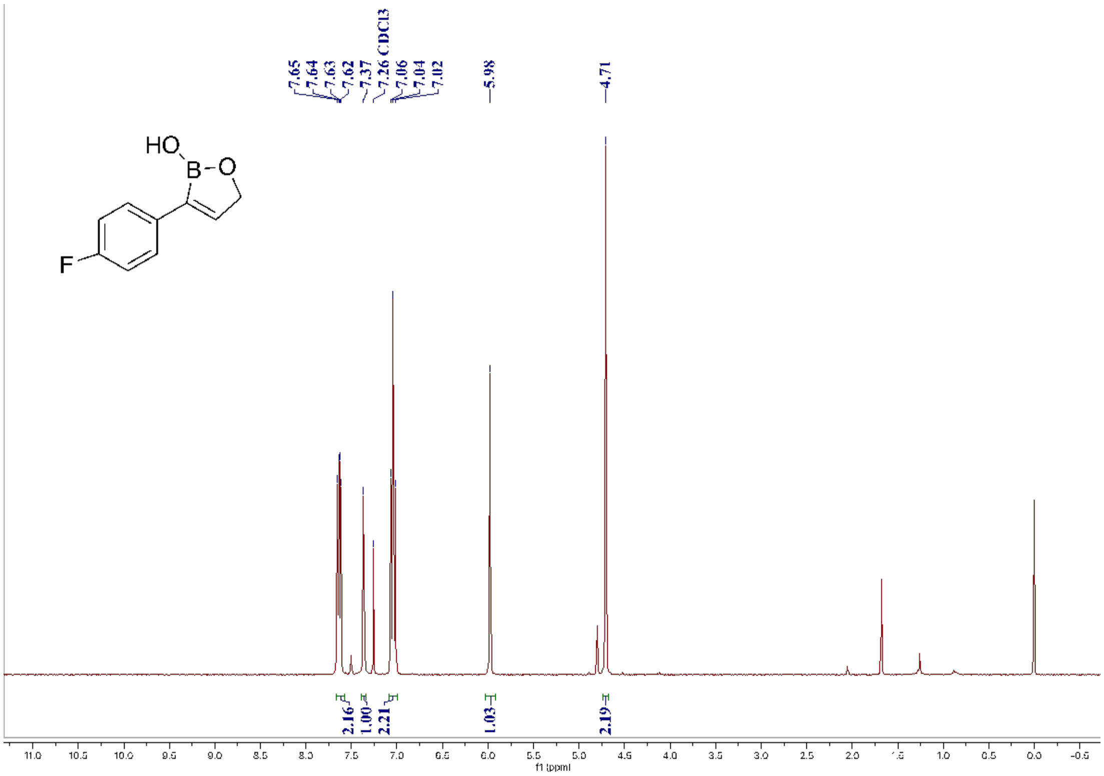

Figure S25. ^{1}H NMR of **6b** ($CDCl_3$, 400 MHz)

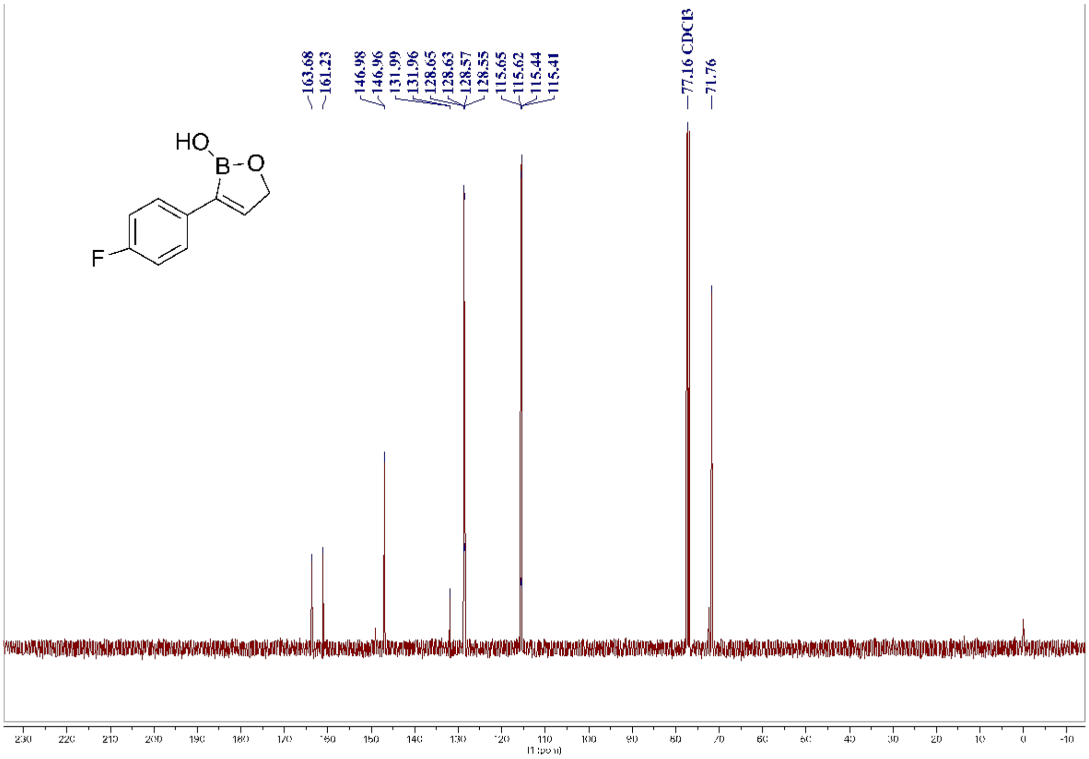

Figure S26. ^{13}C NMR of **6b** ($CDCl_3$, 100 MHz)

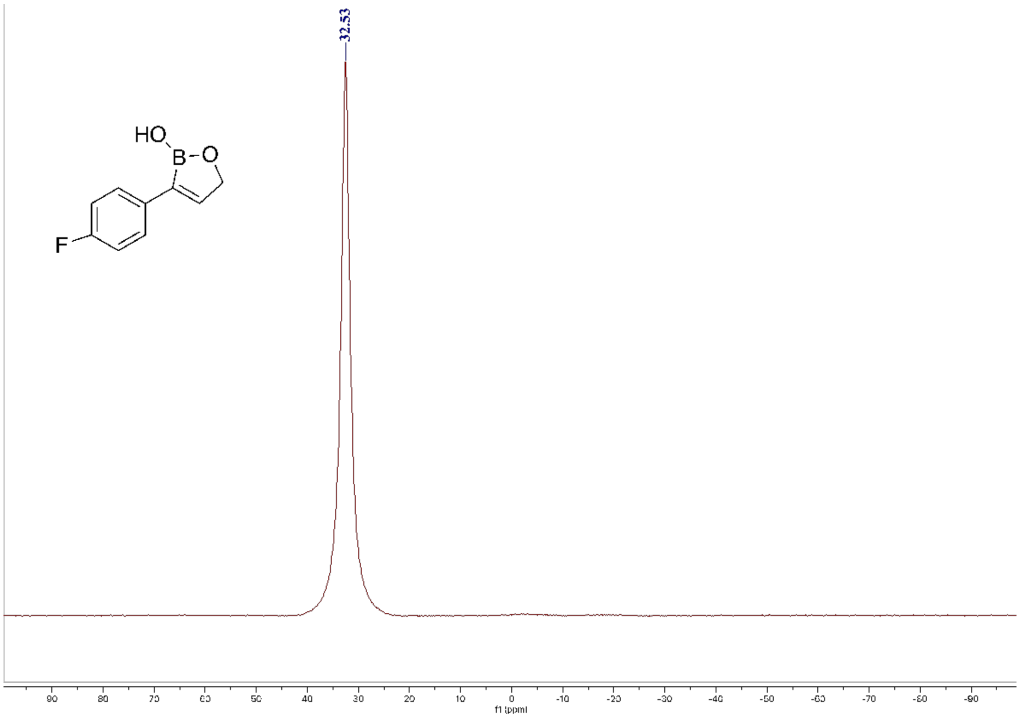

Figure S27. ^{11}B NMR of **6b** ($CDCl_3$, 128 MHz)

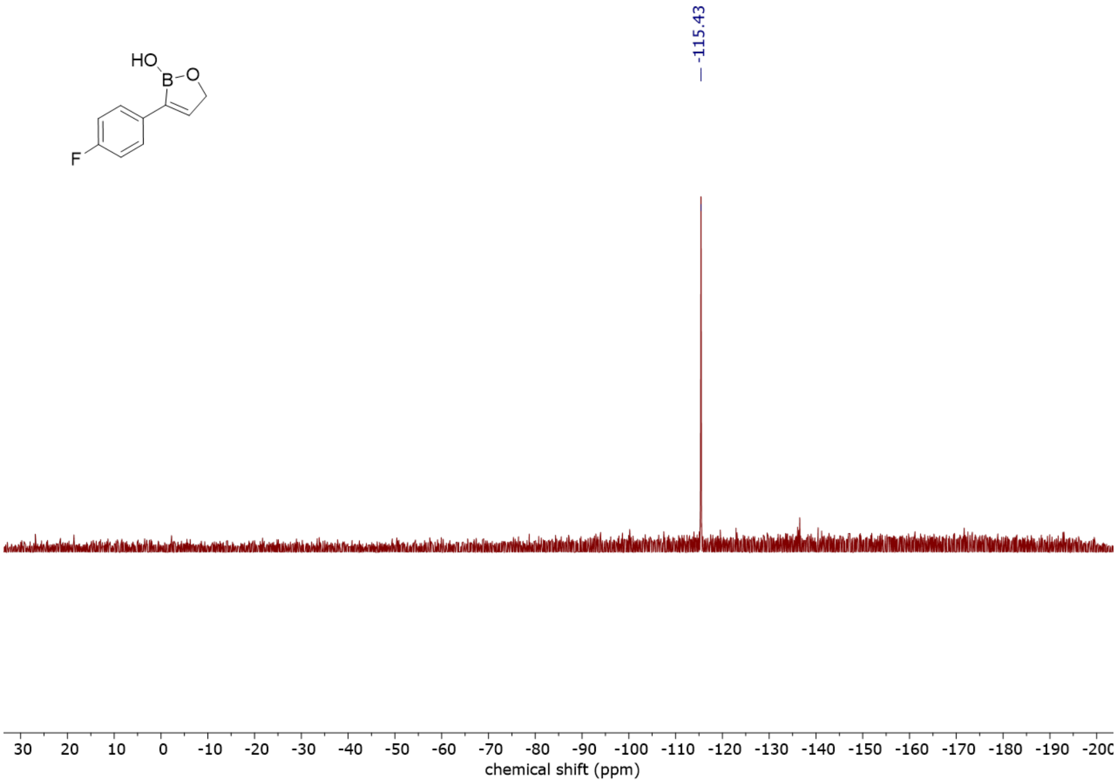

Figure S28. ^{19}F NMR of **6b** ($CDCl_3$, 376 MHz)

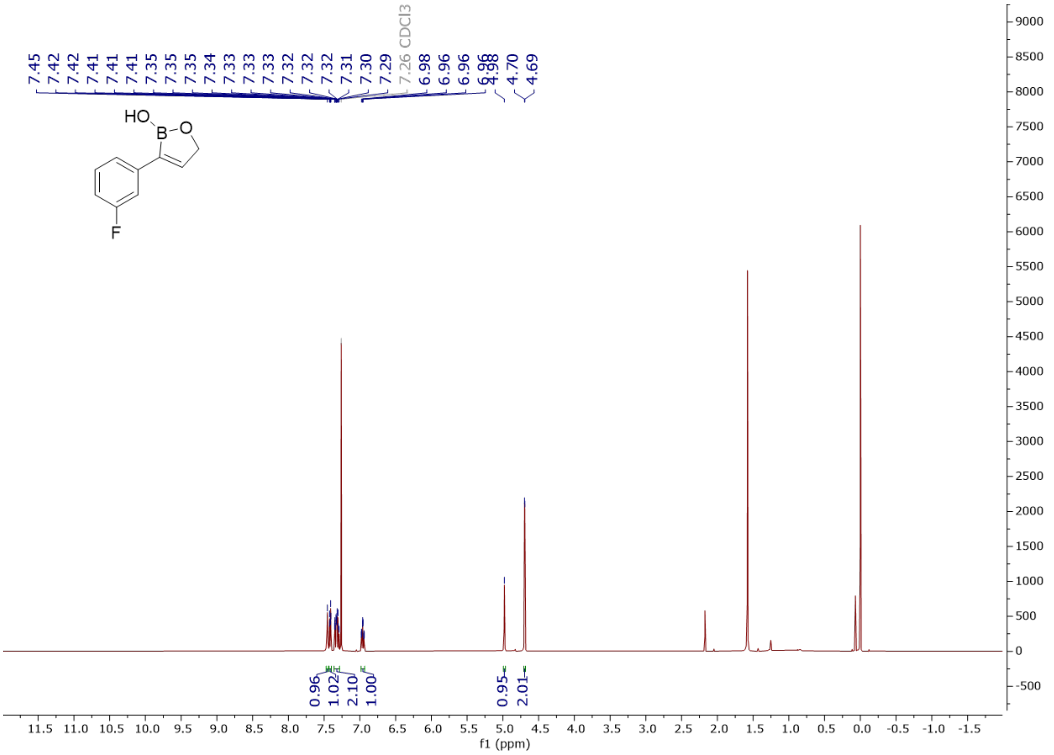

Figure S29. ^{1}H NMR of **6c** ($CDCl_3$, 400 MHz)

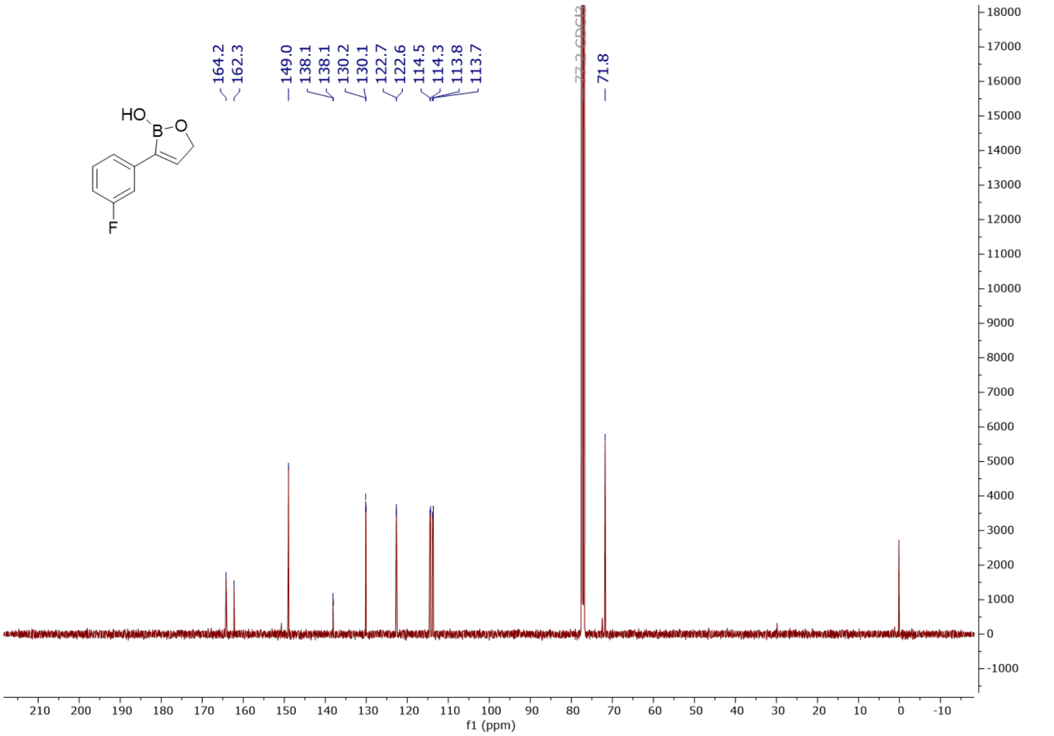

Figure S30. ^{13}C NMR of **6c** ($CDCl_3$, 100 MHz)

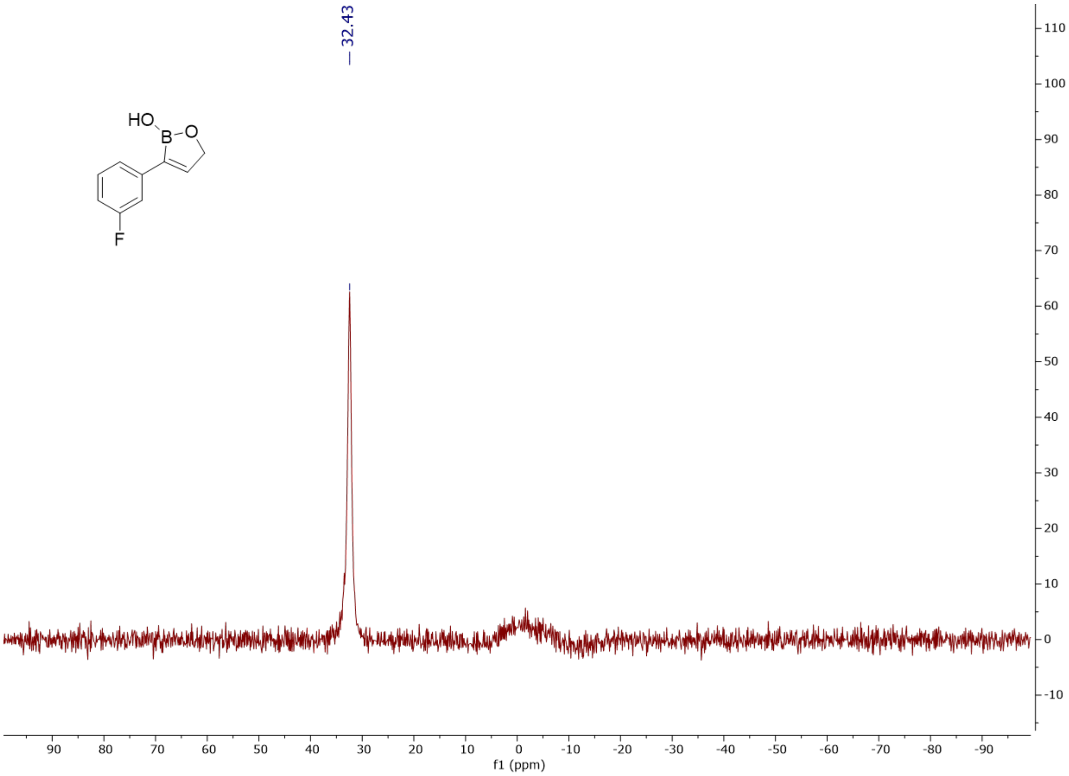

Figure S31. ^{11}B NMR of **6c** ($CDCl_3$, 128 MHz)

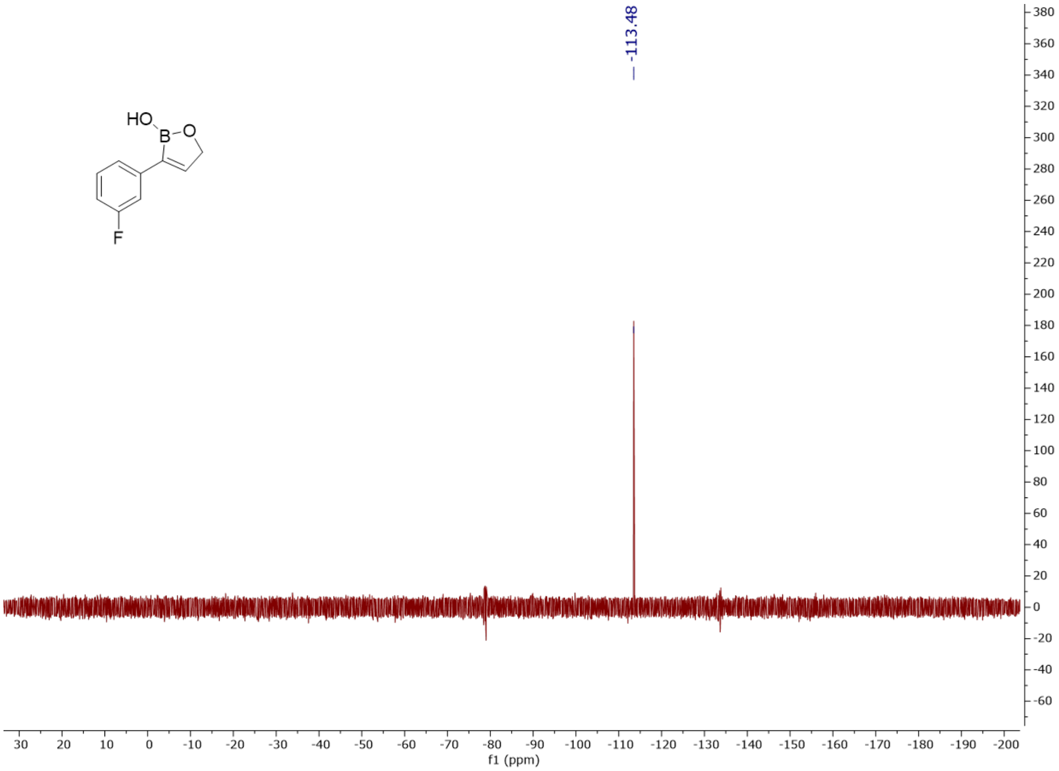

Figure S32. ^{19}F NMR of **6c** ($CDCl_3$, 376 MHz)

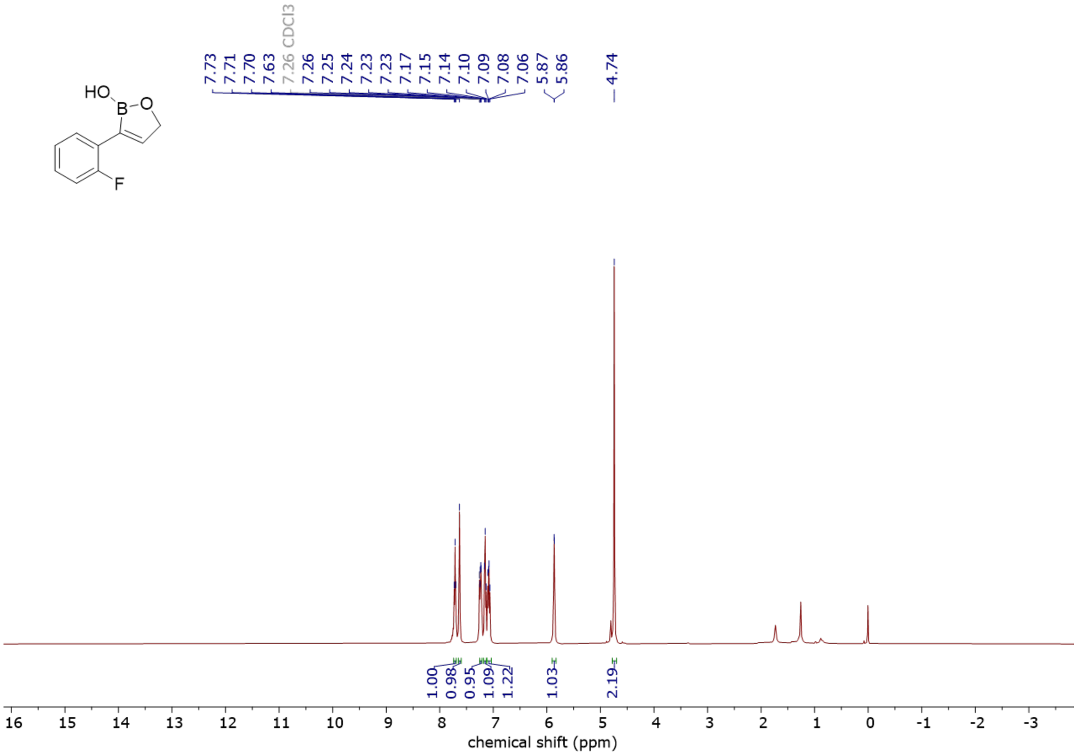

Figure S33. ^{1}H NMR of **6d** ($CDCl_3$, 400 MHz)

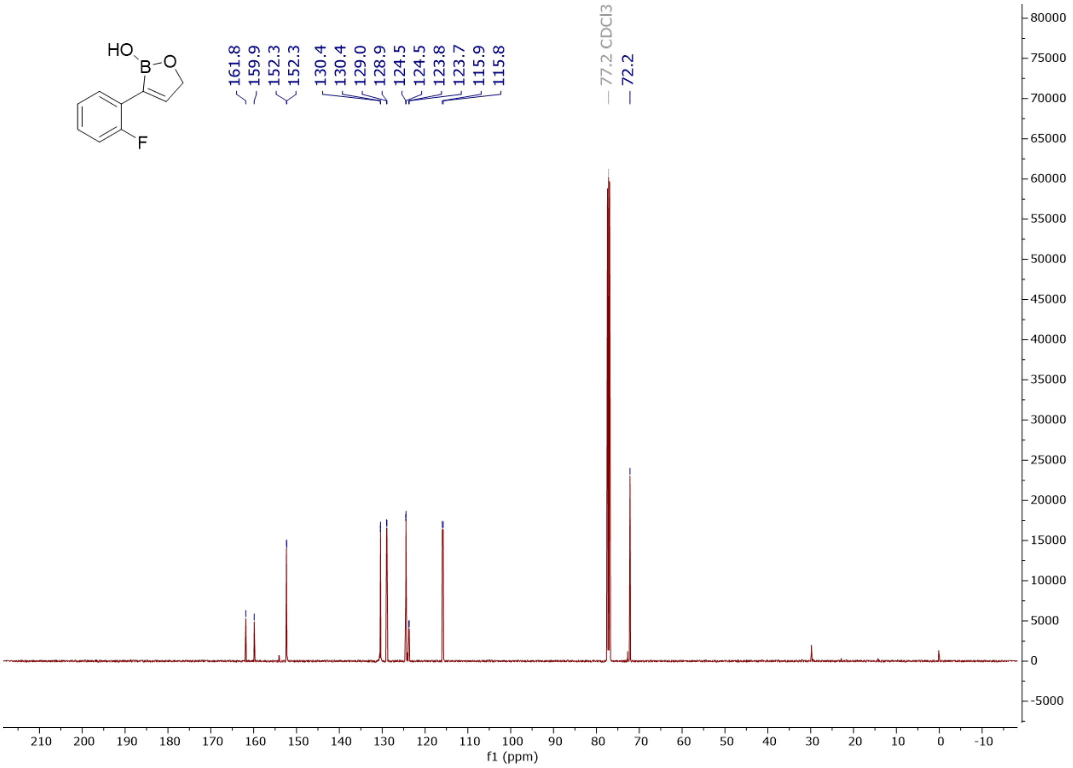

Figure S34. ^{13}C NMR of **6d** ($CDCl_3$, 100 MHz)

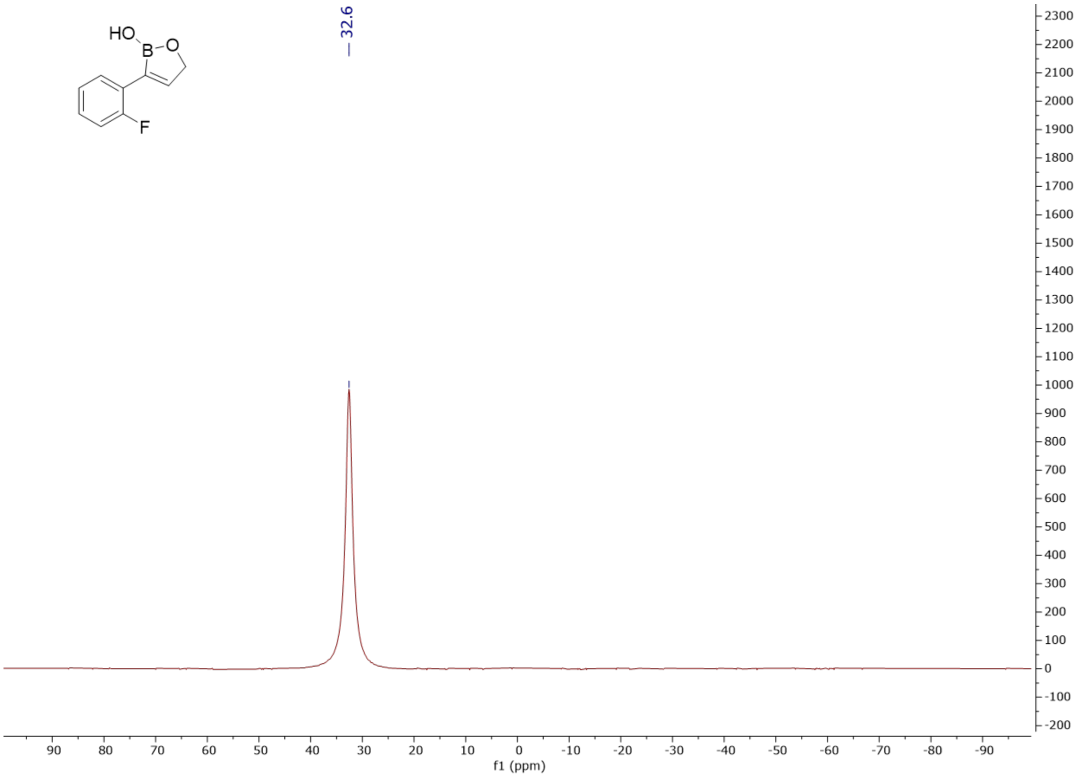

Figure S35. ^{11}B NMR of **6d** ($CDCl_3$, 128 MHz)

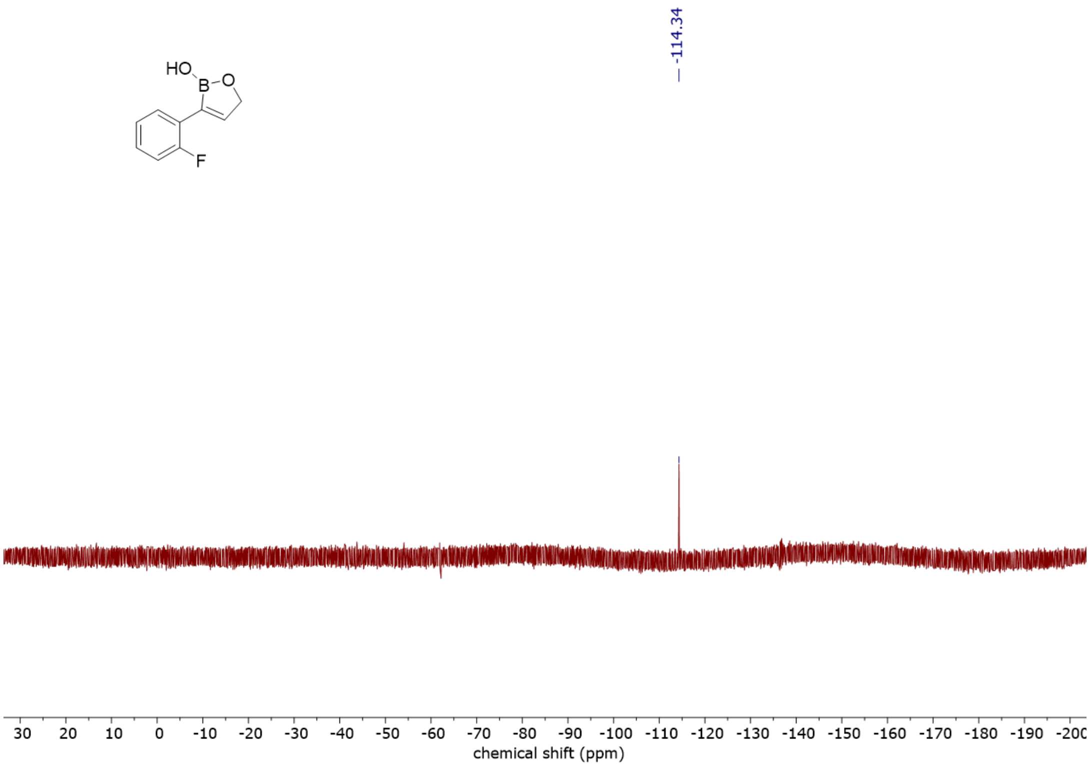

Figure S36. ^{19}F NMR of **6d** ($CDCl_3$, 376 MHz)

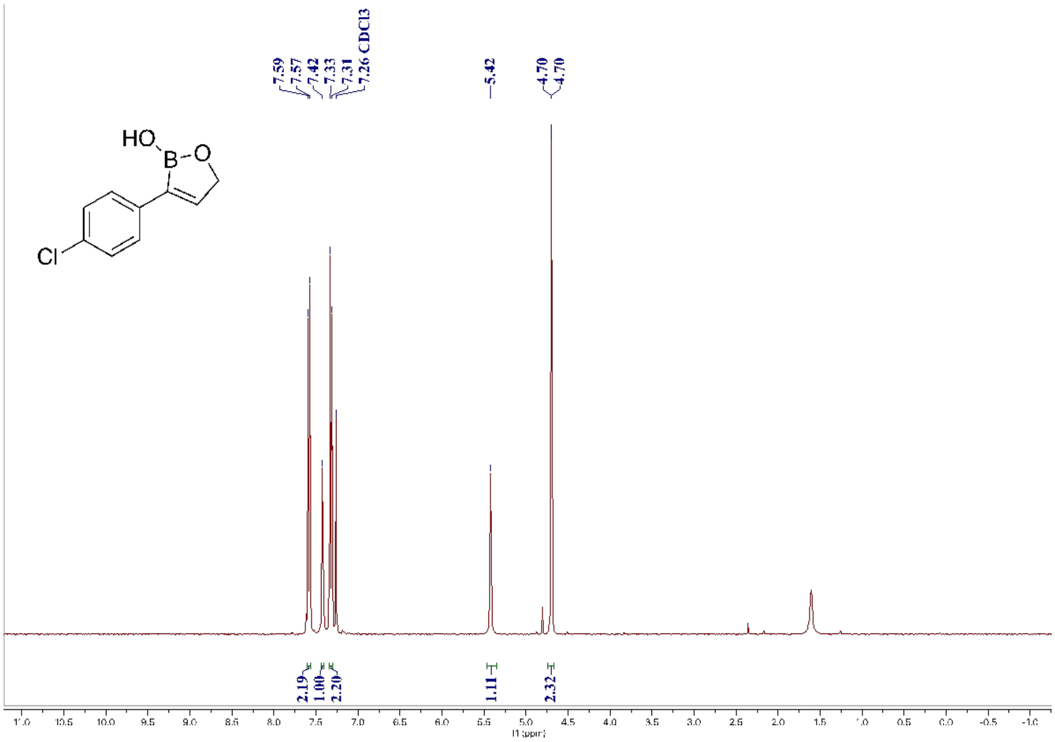

Figure S37. ^{1}H NMR of **6e** ($CDCl_3$, 400 MHz)

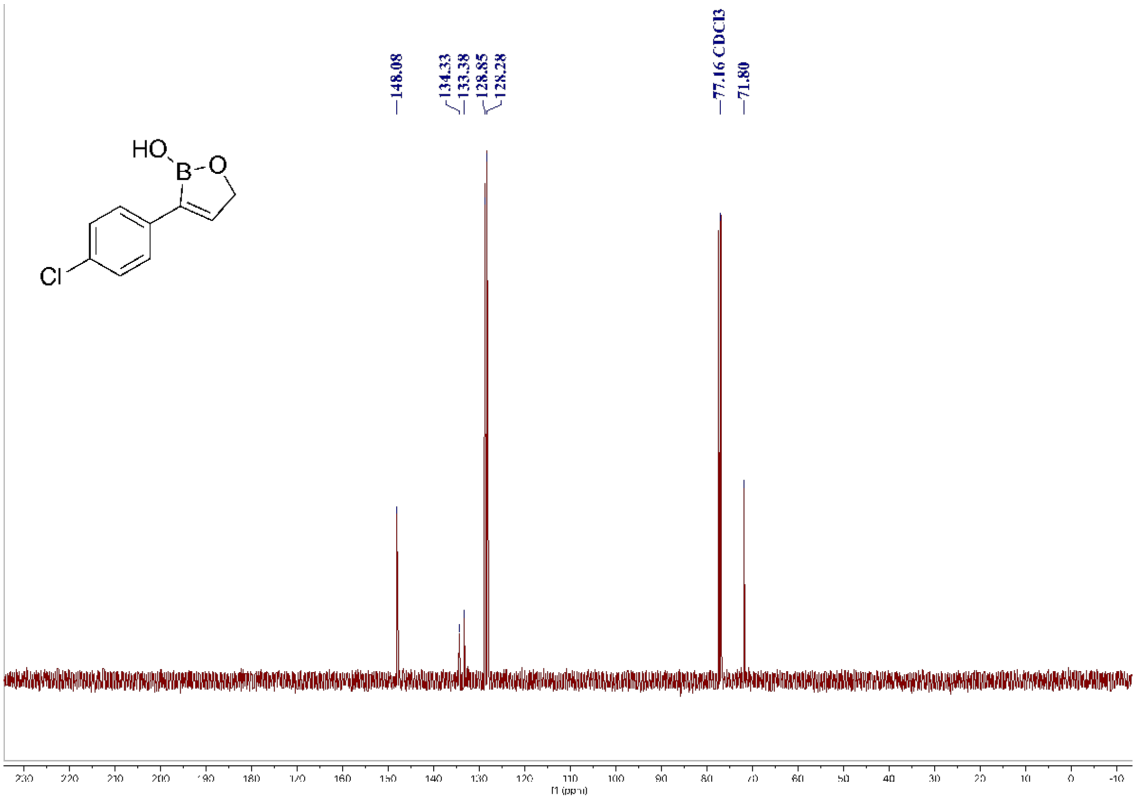

Figure S38. ^{13}C NMR of **6e** ($CDCl_3$, 100 MHz)

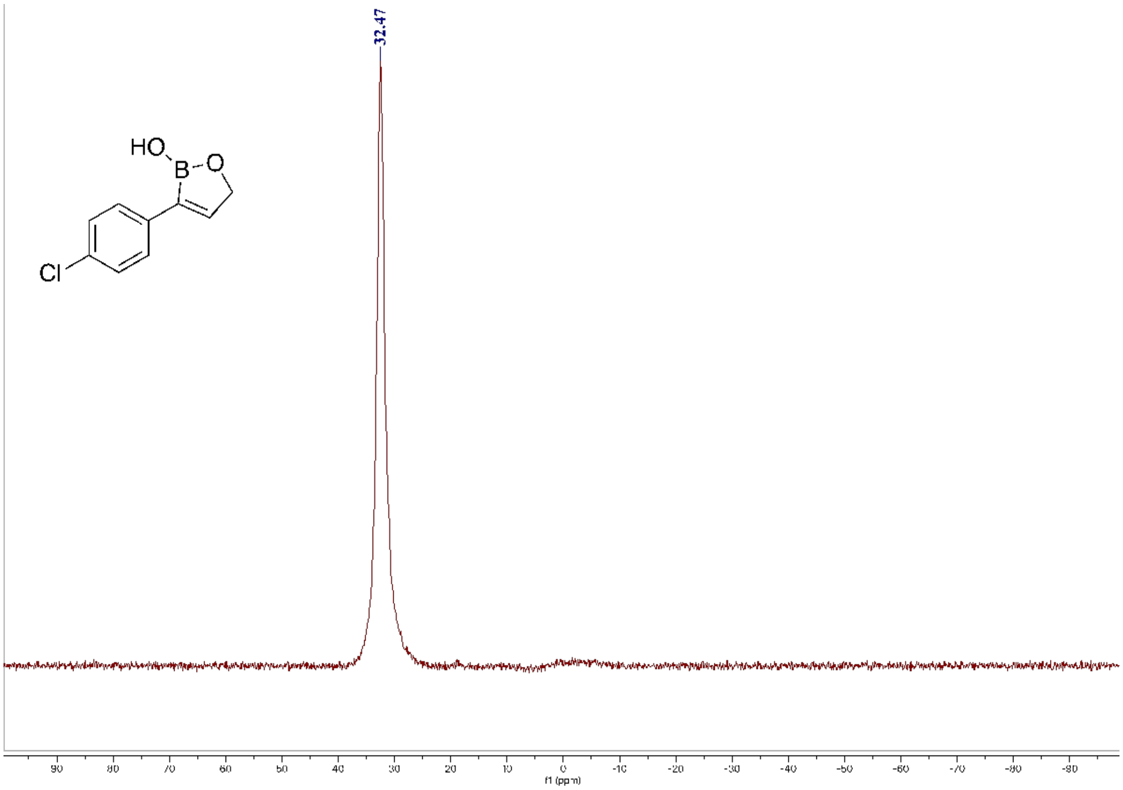

Figure S39. ^{11}B NMR of **6e** ($CDCl_3$, 128 MHz)

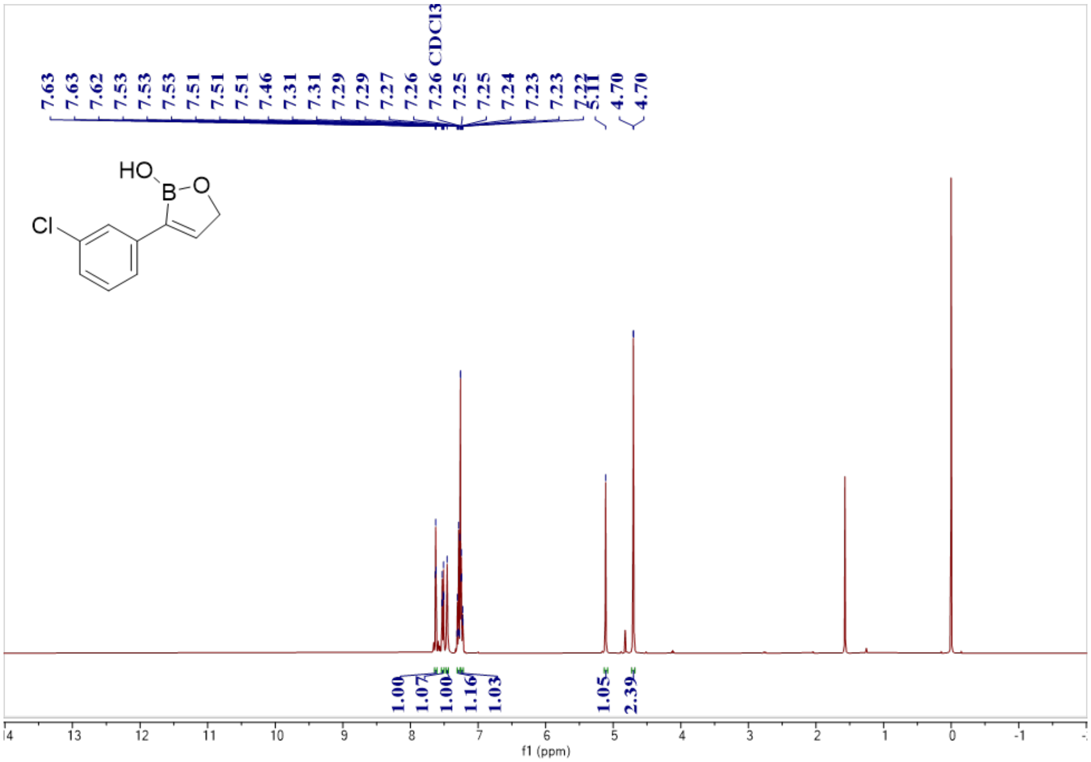

Figure S40. ^{1}H NMR of **6f** ($CDCl_3$, 400 MHz)

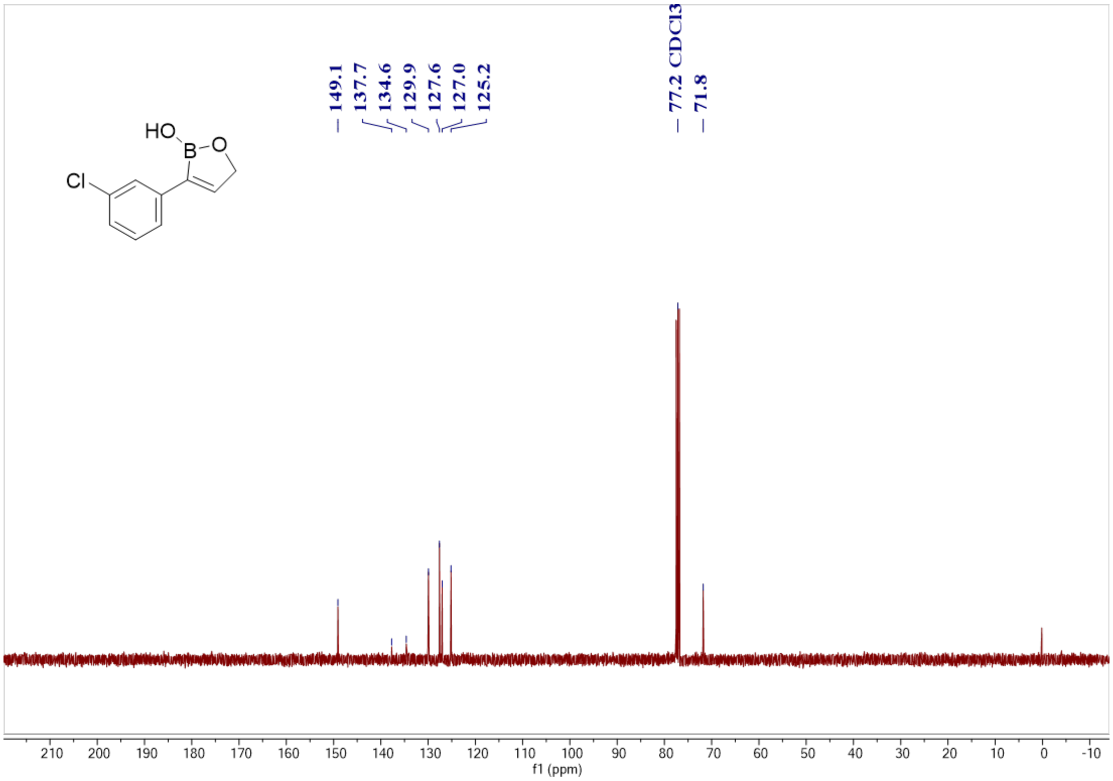

Figure S41. ^{13}C NMR of **6f** ($CDCl_3$, 100 MHz)

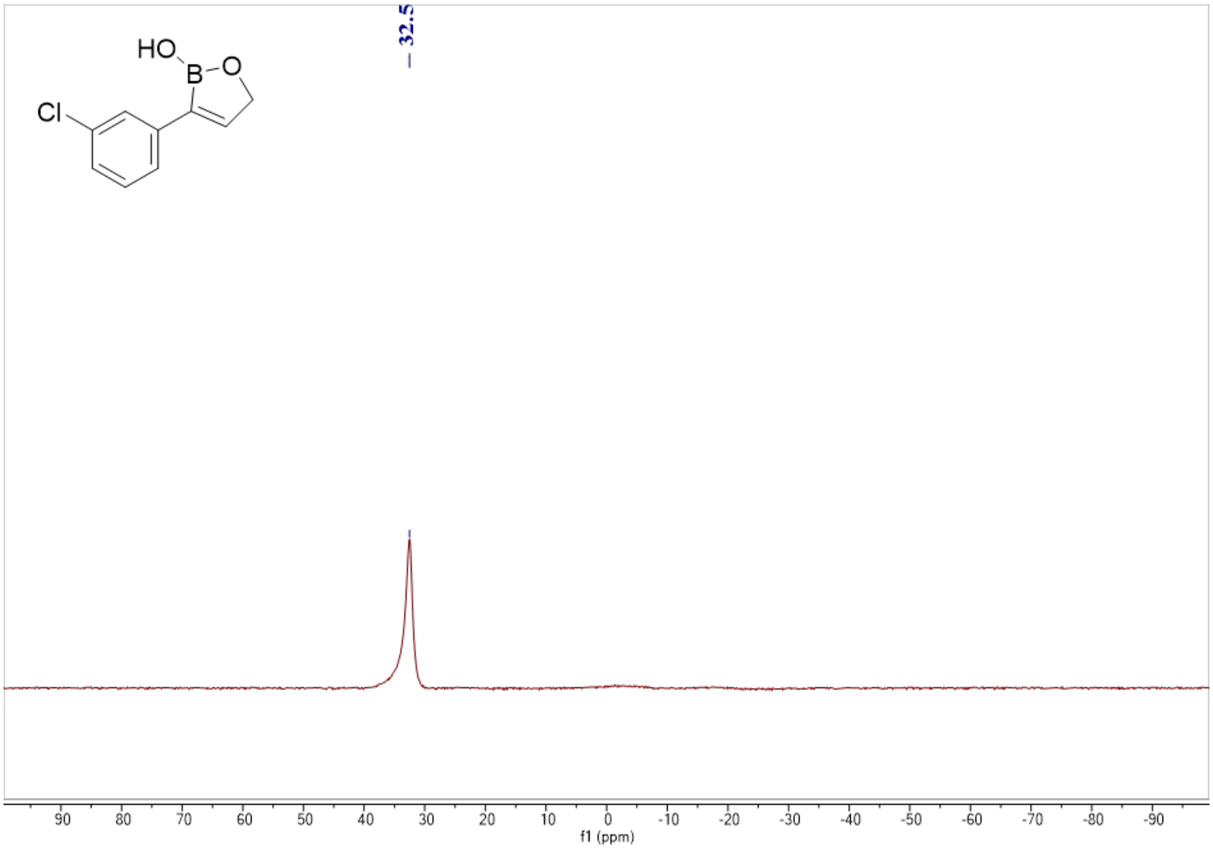

Figure S42. ^{11}B NMR of **6f** ($CDCl_3$, 128 MHz)

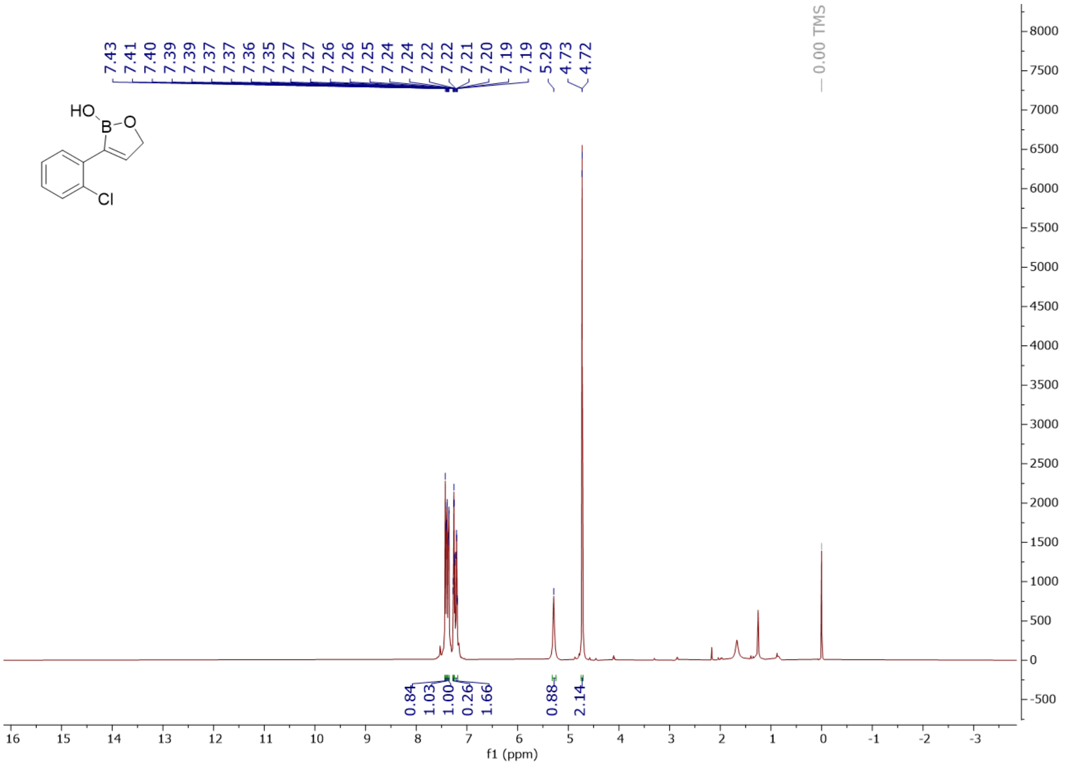

Figure S43. ^{1}H NMR of **6g** ($CDCl_3$, 400 MHz)

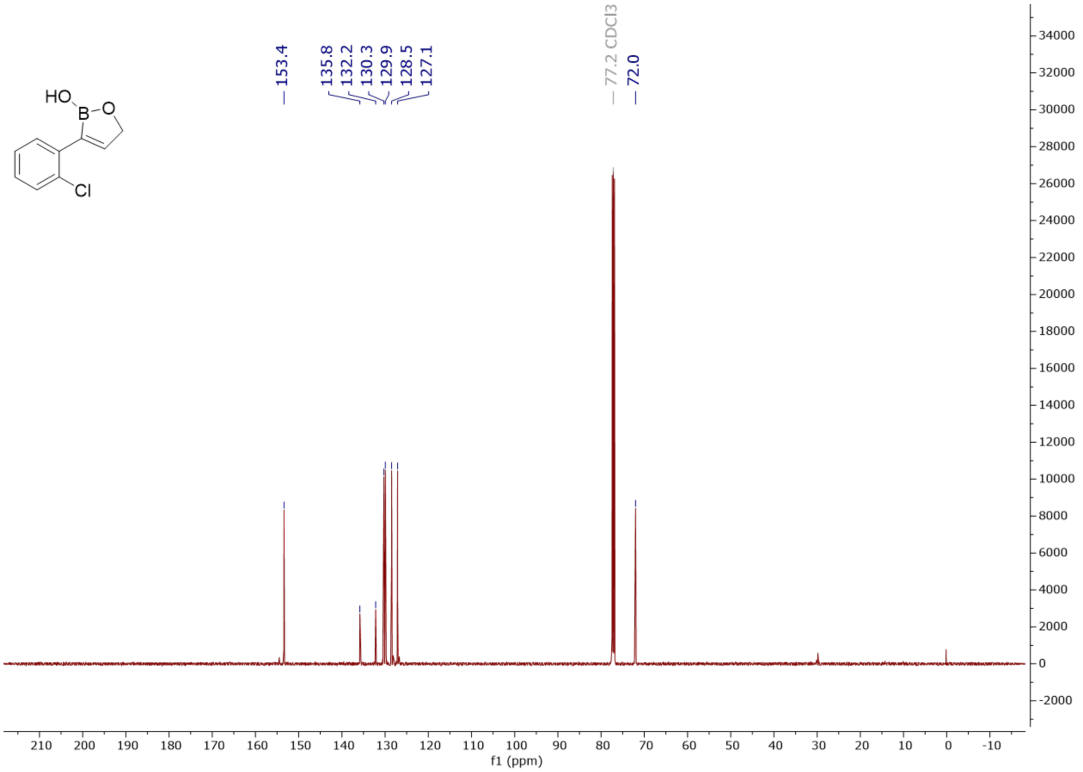

Figure S44. ^{13}C NMR of **6g** ($CDCl_3$, 100 MHz)

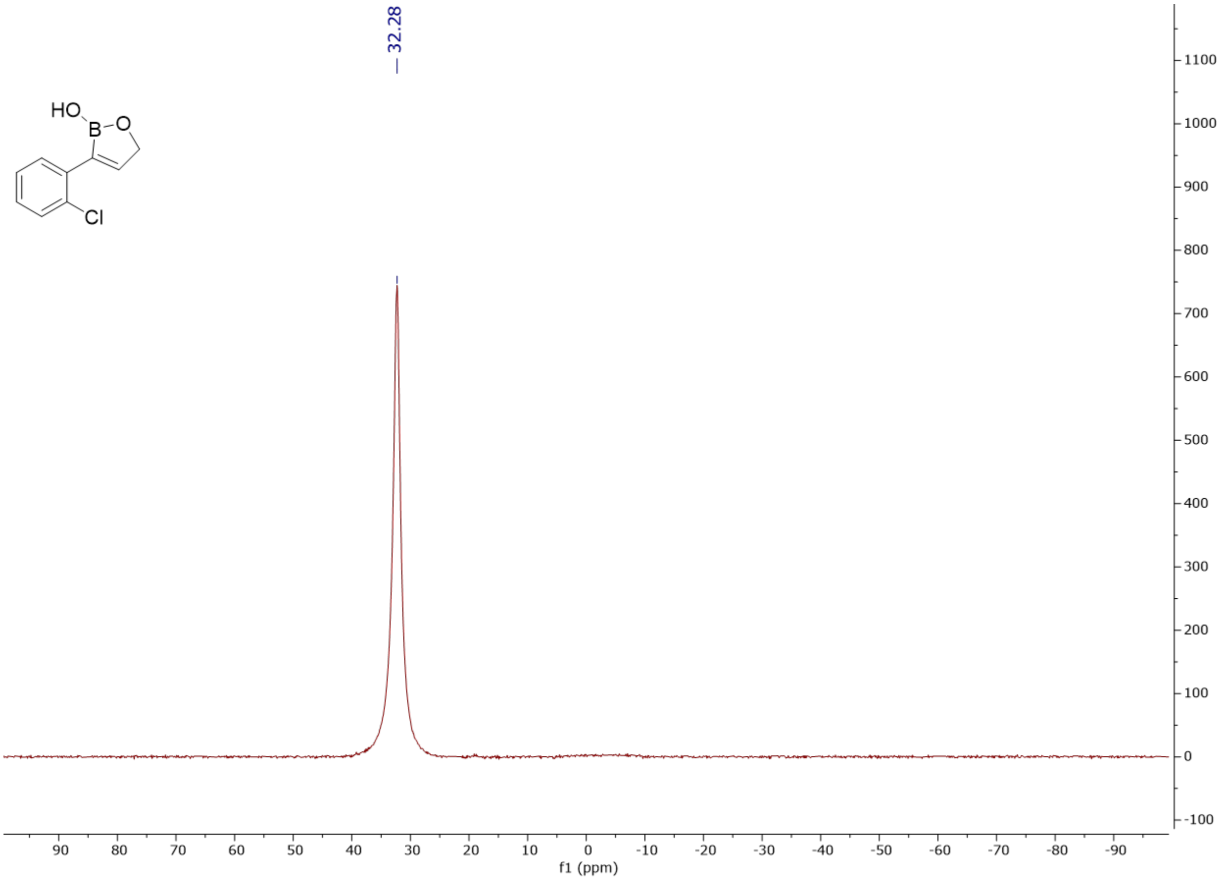

Figure S45. ^{11}B NMR of **6g** ($CDCl_3$, 128 MHz)

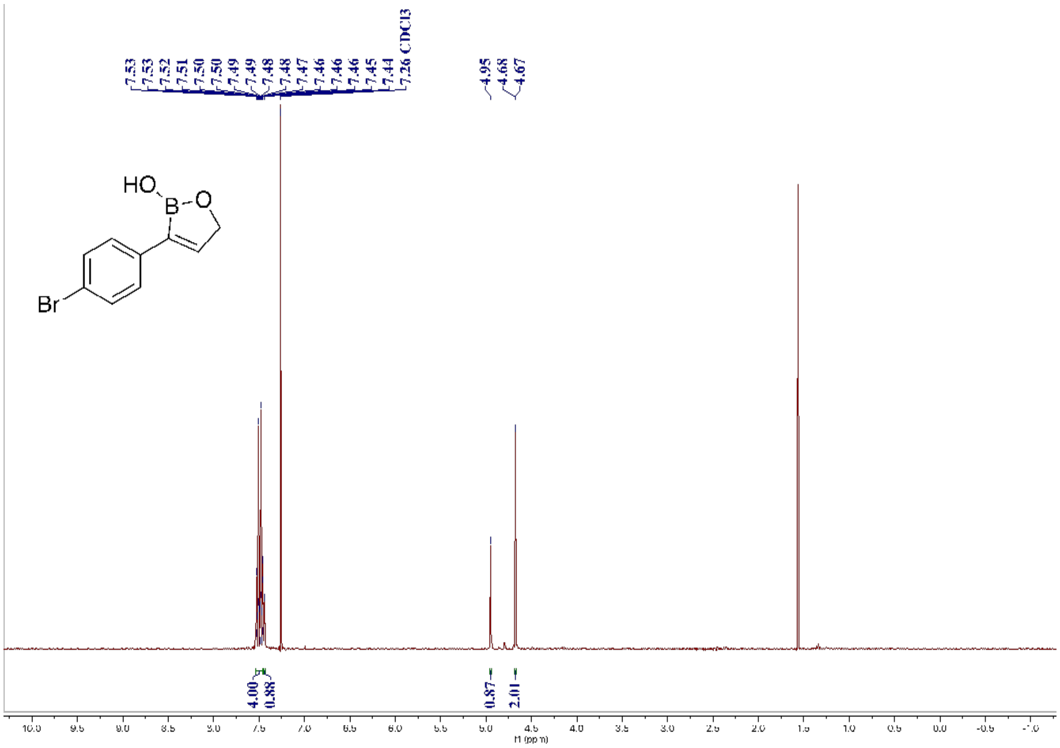

Figure S46. ^{1}H NMR of **6h** ($CDCl_3$, 400 MHz)

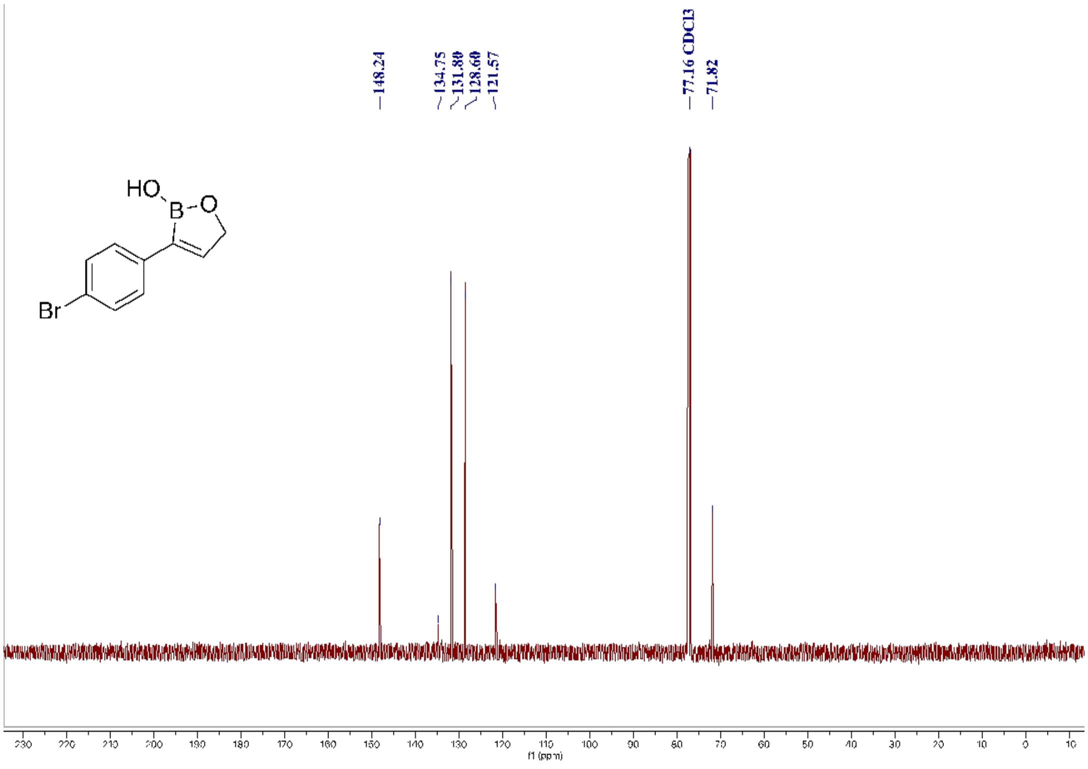

Figure S47. ^{13}C NMR of **6h** ($CDCl_3$, 100 MHz)

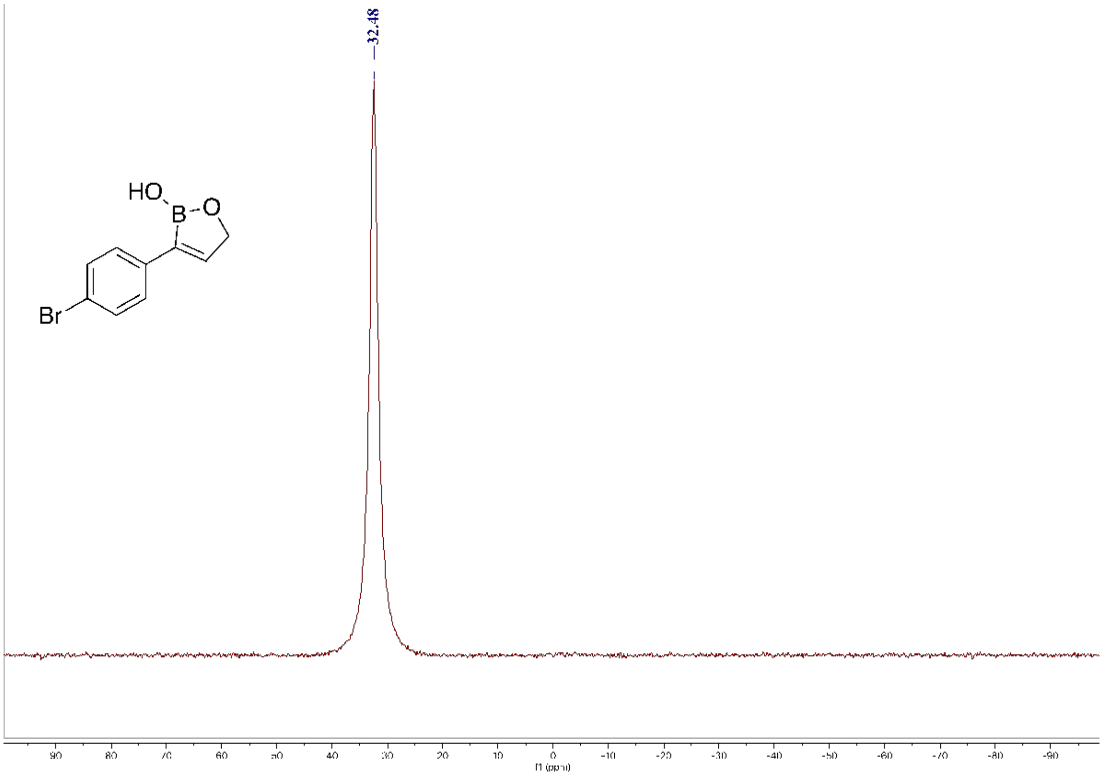

Figure S48. ^{11}B NMR of **6h** ($CDCl_3$, 128 MHz)

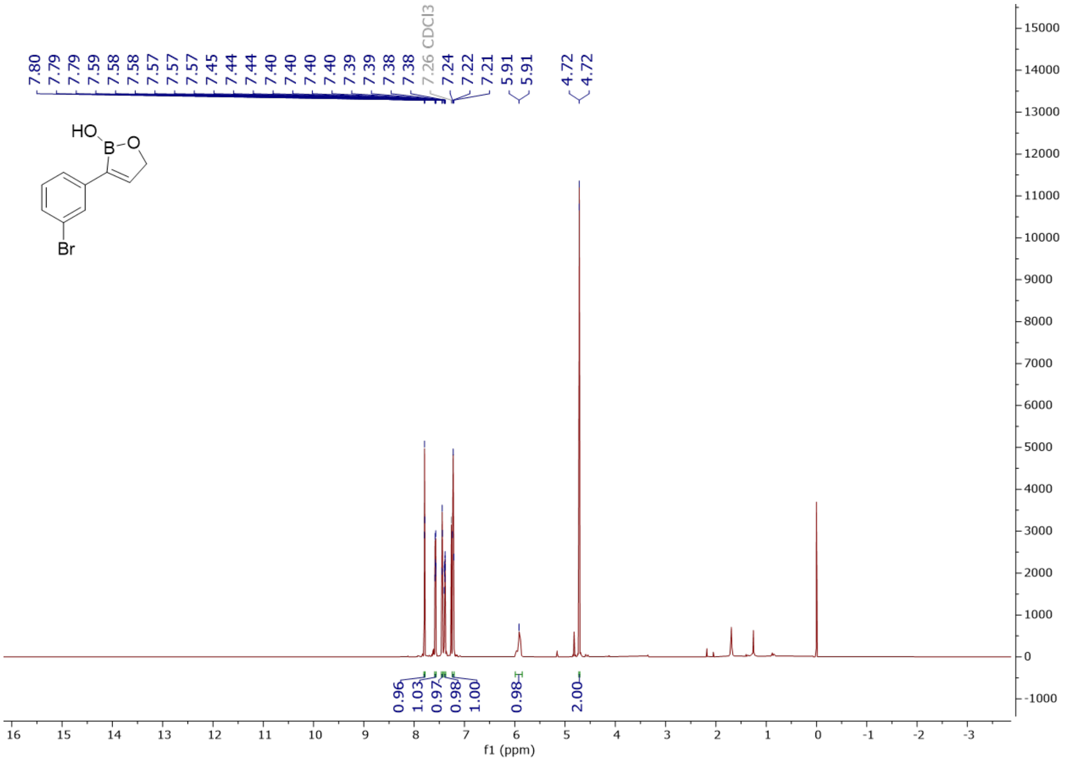

Figure S49. ^{1}H NMR of **6i** ($CDCl_3$, 600 MHz)

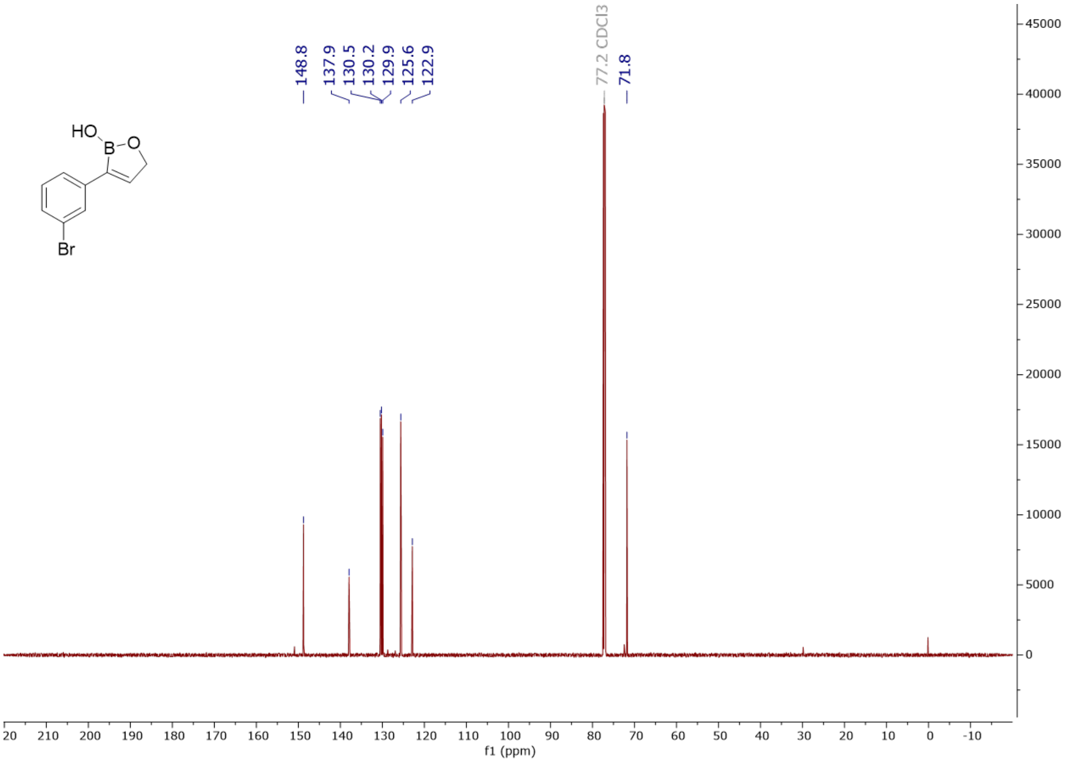

Figure S50. ^{13}C NMR of **6i** ($CDCl_3$, 151 MHz)

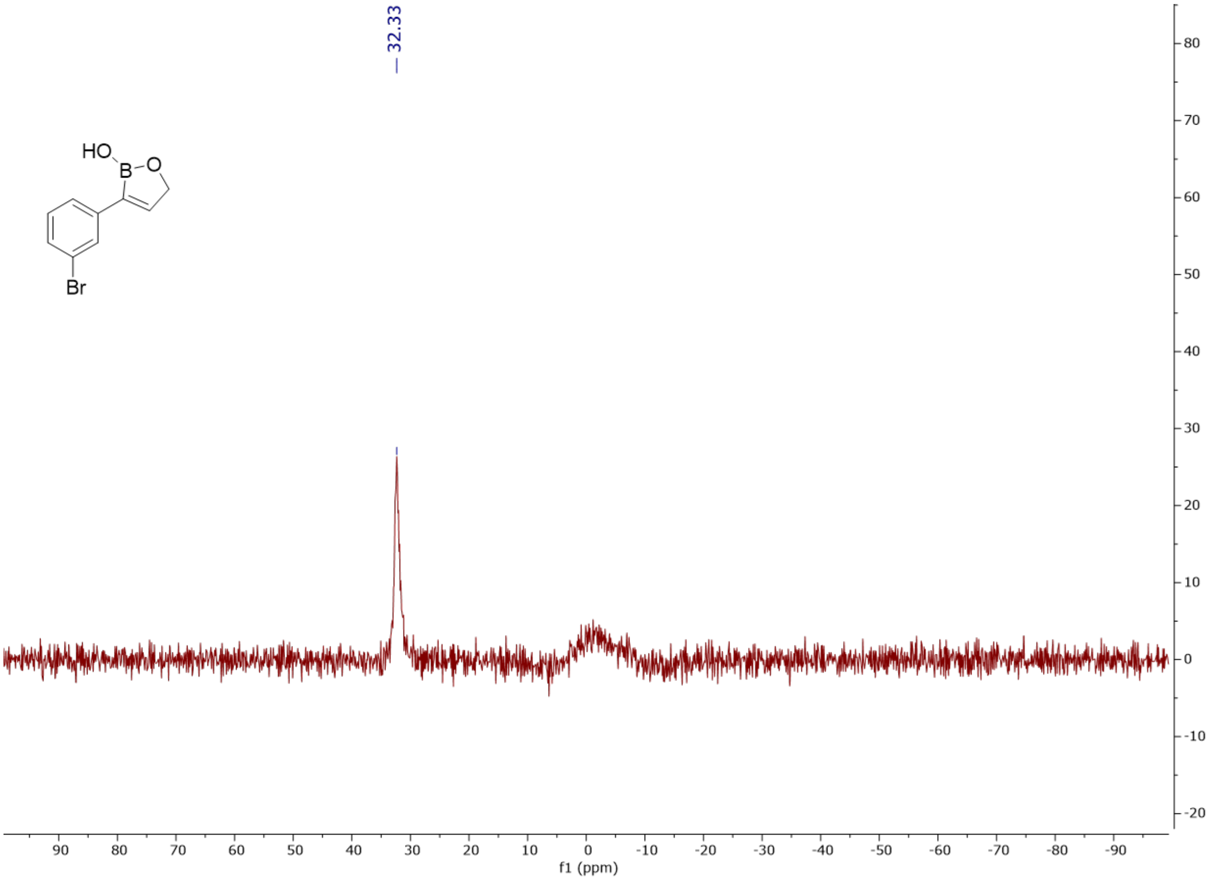

Figure S51. ^{11}B NMR of **6i** ($CDCl_3$, 128 MHz)

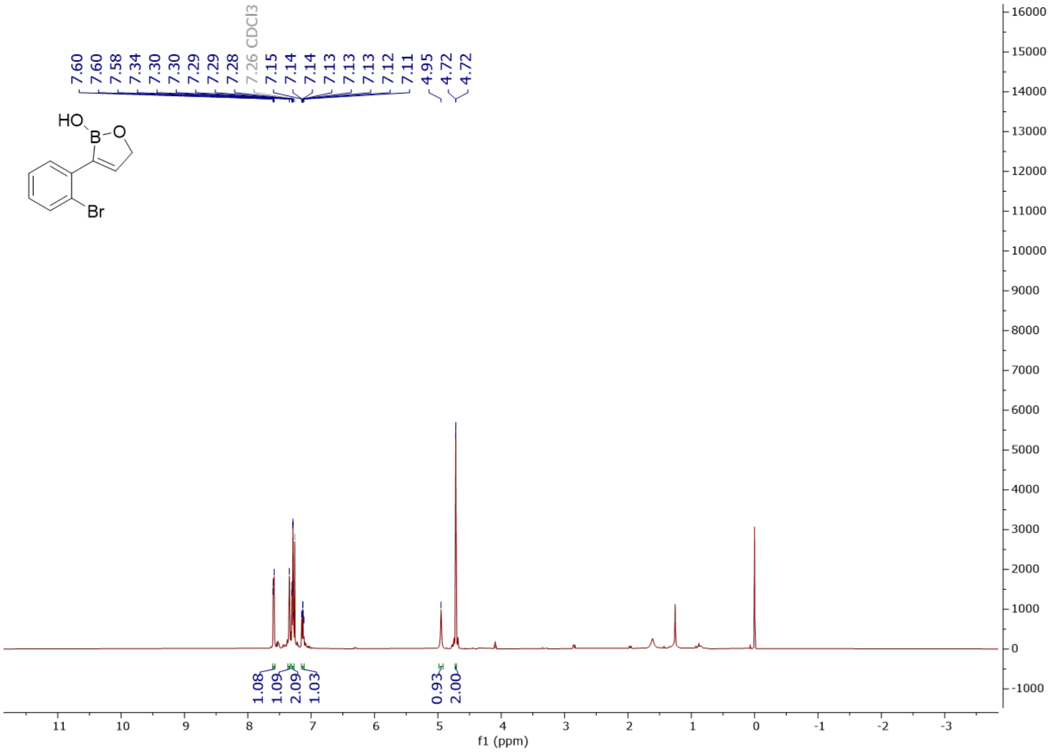

Figure S52. ^{1}H NMR of **6j** ($CDCl_3$, 500 MHz)

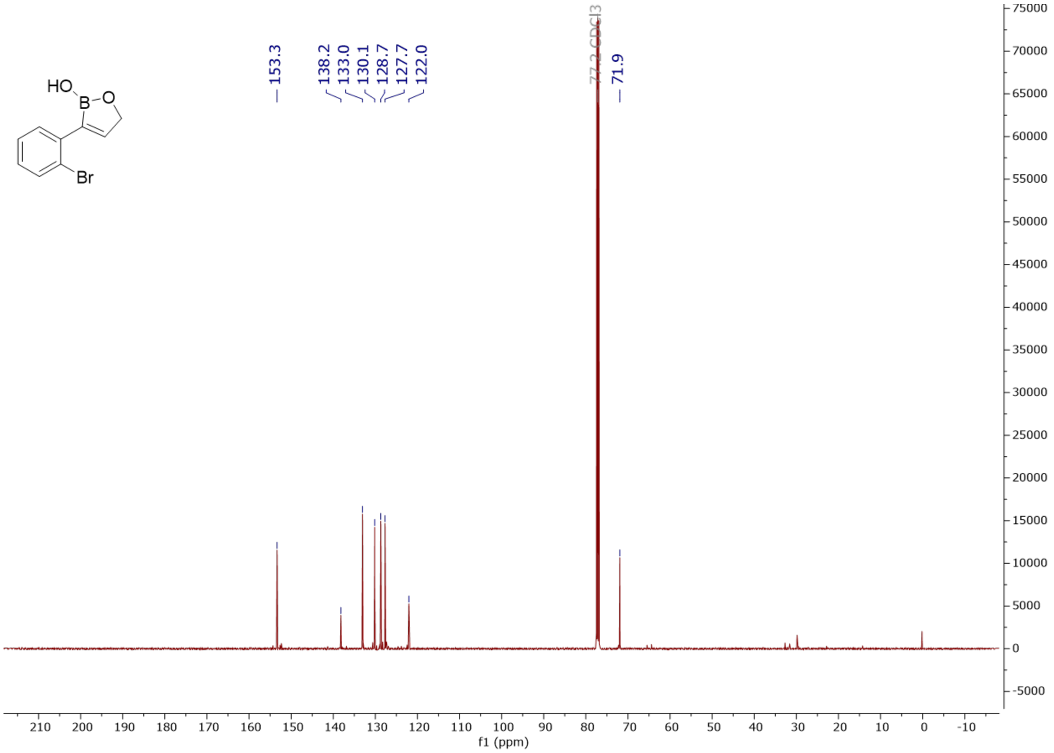

Figure S53. ^{13}C NMR of **6j** ($CDCl_3$, 126 MHz)

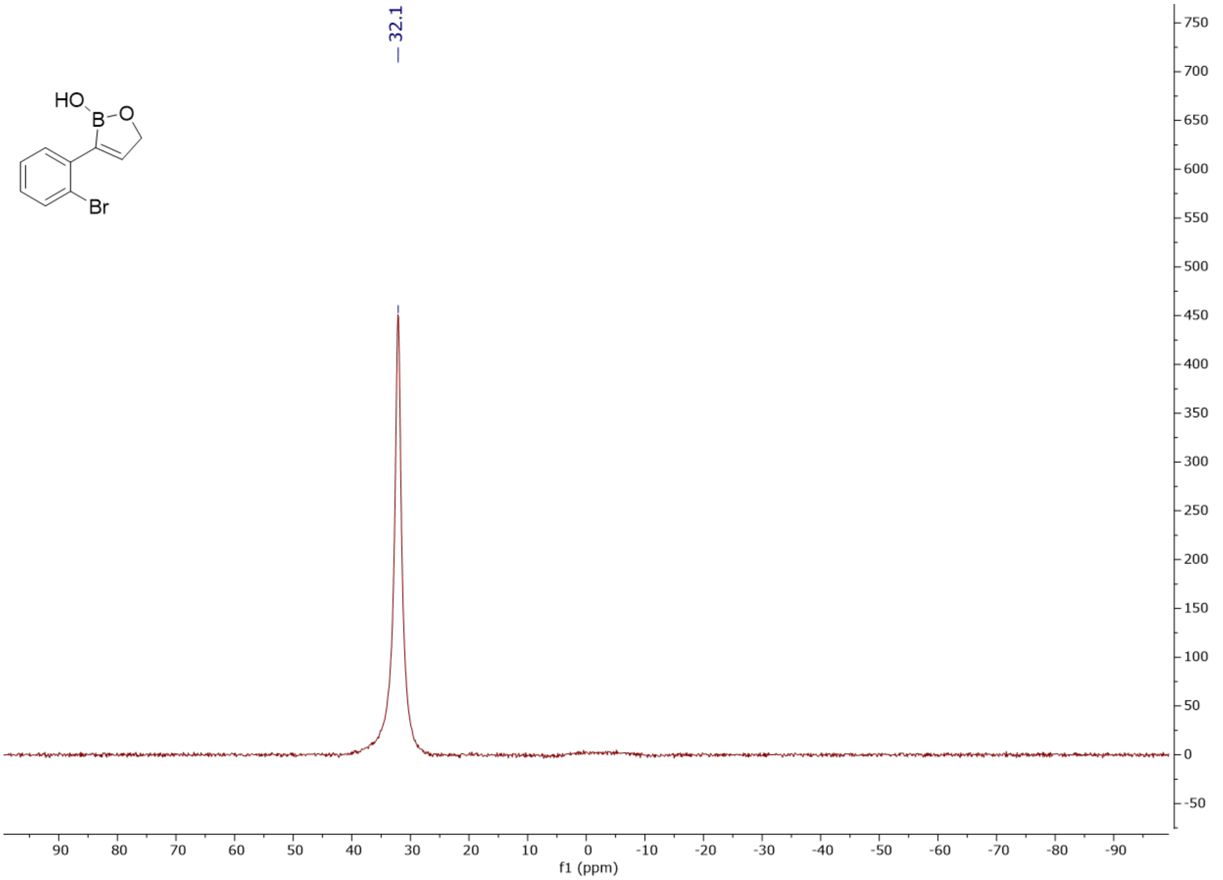

Figure S54. ^{11}B NMR of **6j** ($CDCl_3$, 128 MHz)

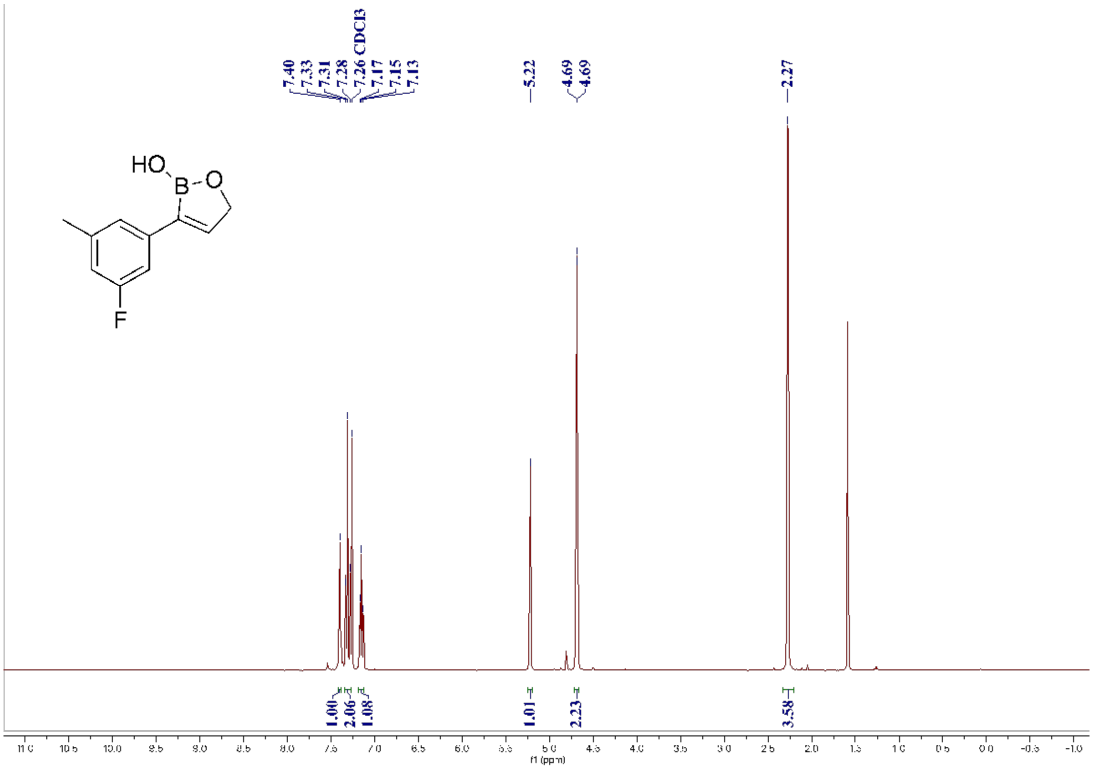

Figure S55. ^{1}H NMR of **6k** ($CDCl_3$, 400 MHz)

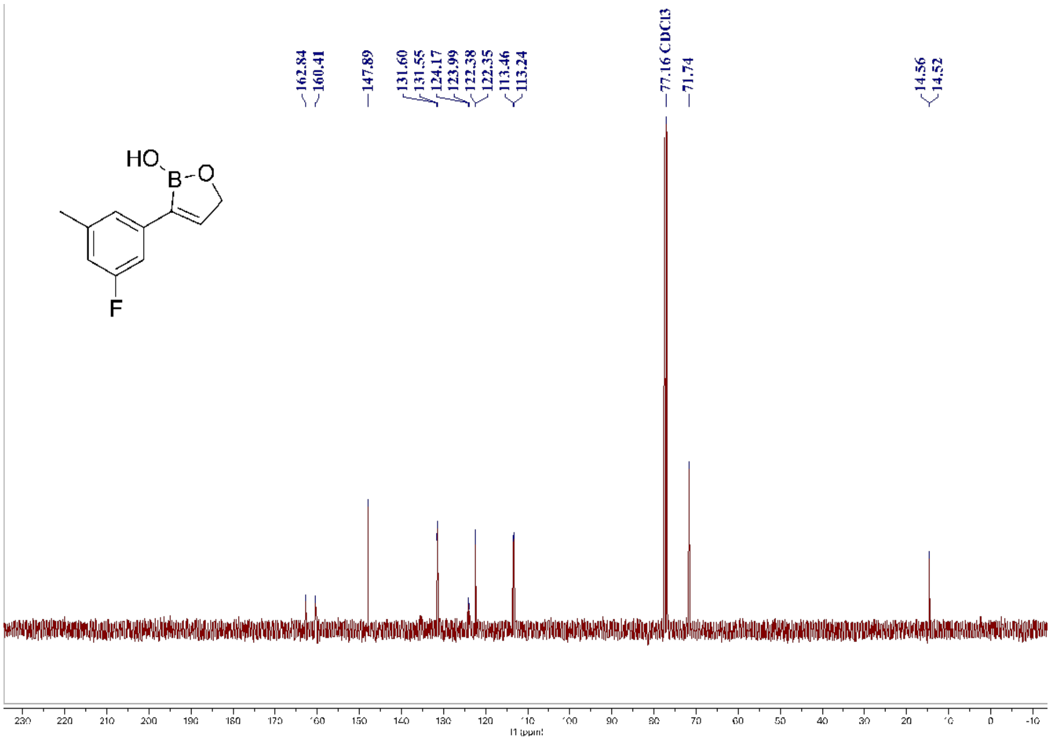

Figure S56. ^{13}C NMR of **6k** ($CDCl_3$, 100 MHz)

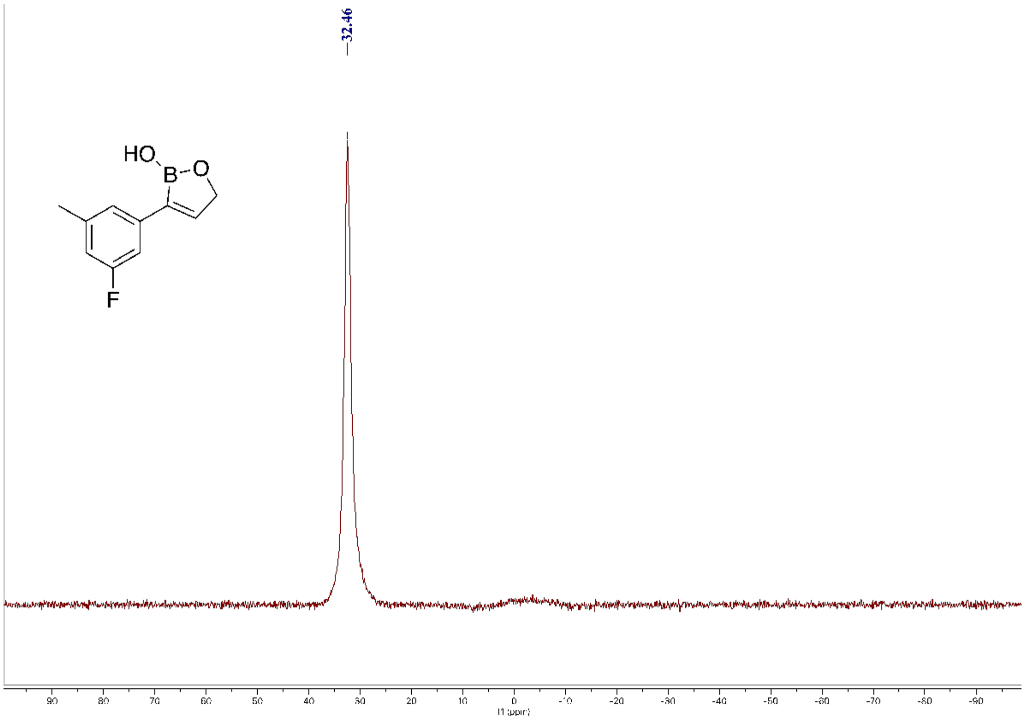

Figure S57. ^{11}B NMR of **6k** ($CDCl_3$, 128 MHz)

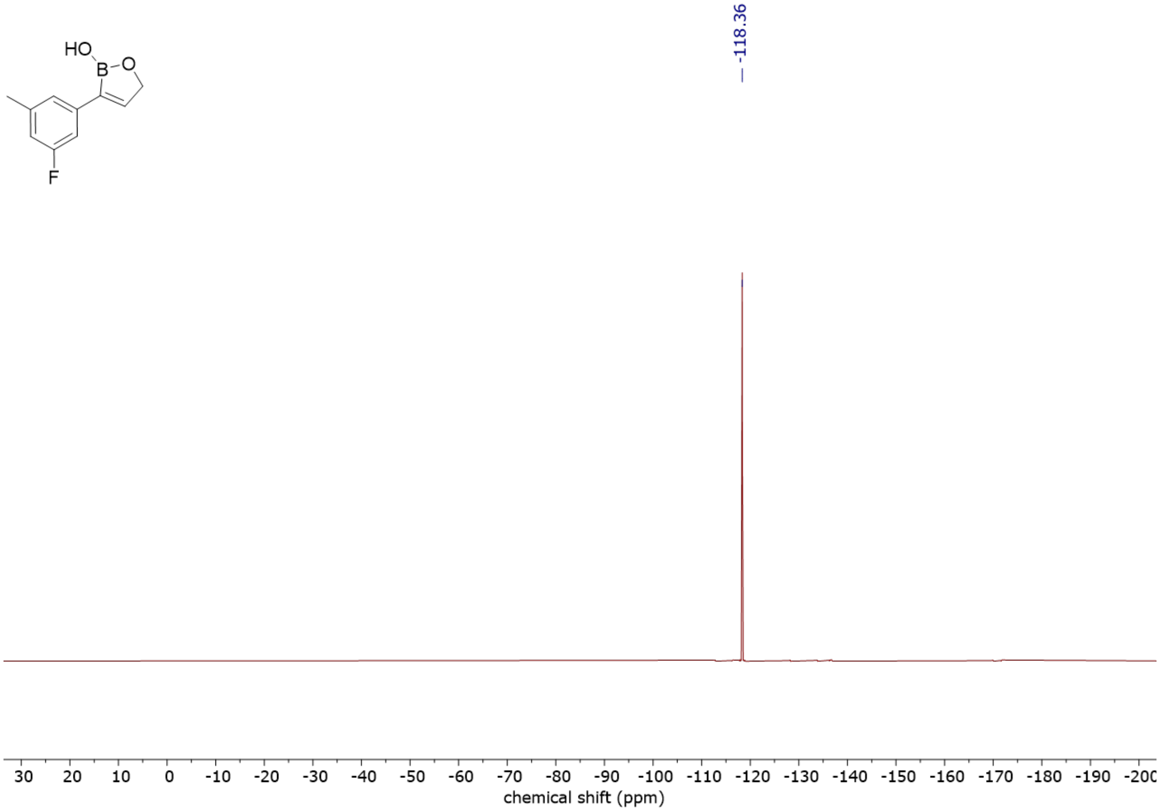

Figure S58. ^{19}F NMR of **6k** ($CDCl_3$, 376 MHz)

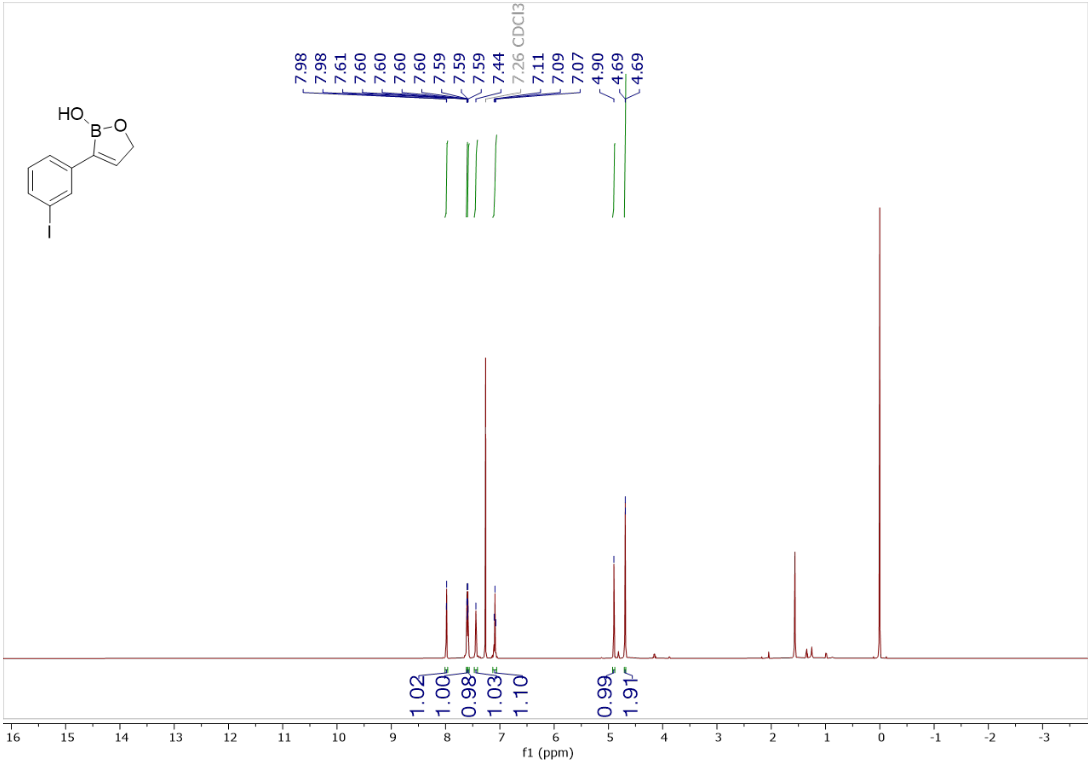

Figure S59. ^{1}H NMR of **6l** ($CDCl_3$, 500 MHz)

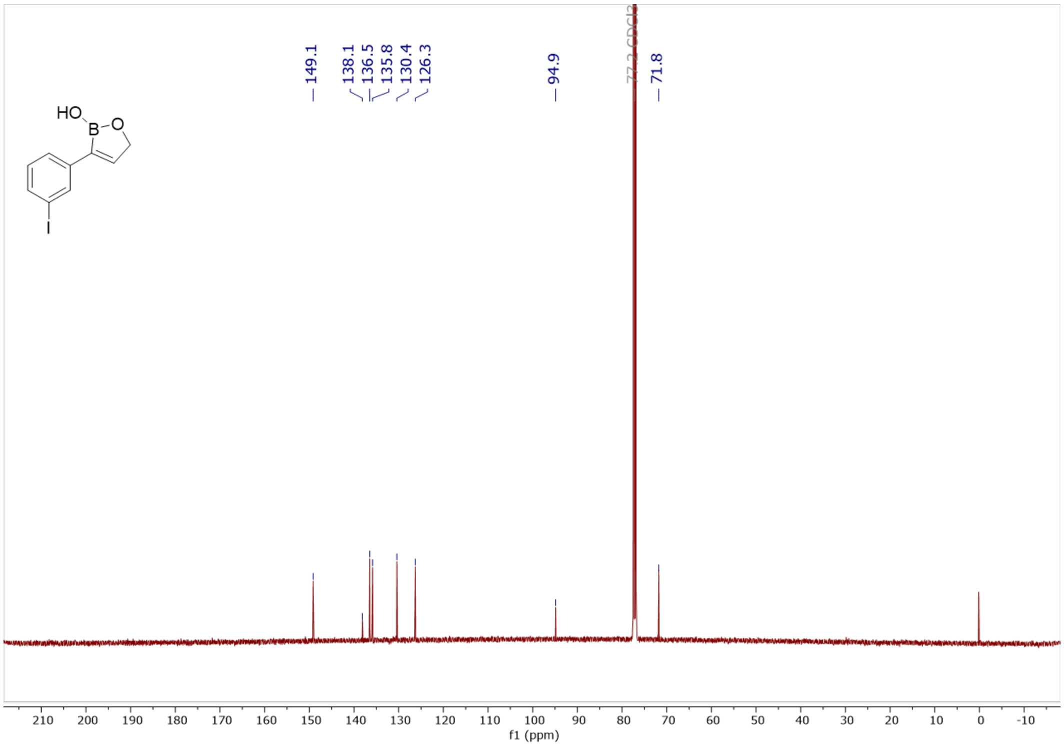

Figure S60. ^{13}C NMR of **6l** ($CDCl_3$, 126 MHz)

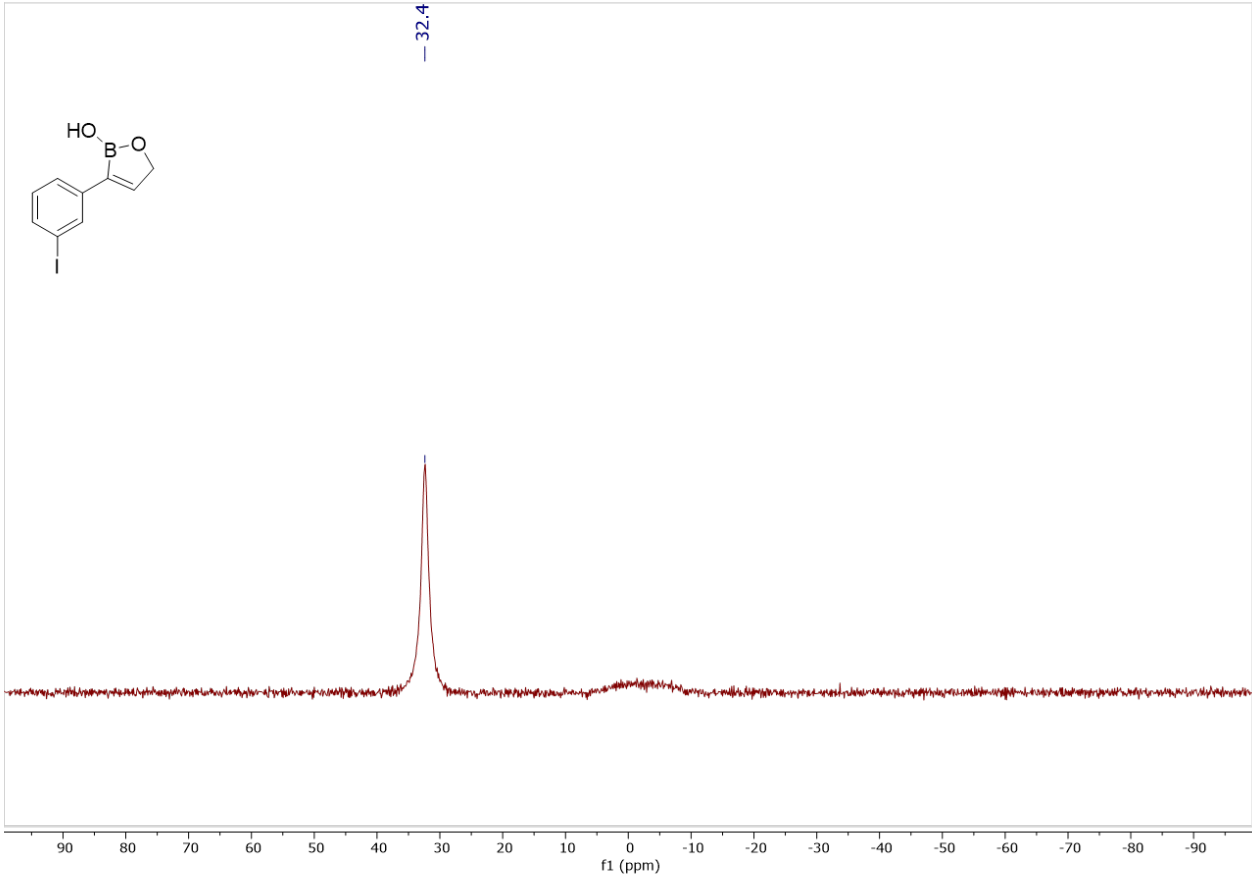

Figure S61. ^{11}B NMR of **6l** ($CDCl_3$, 128 MHz)

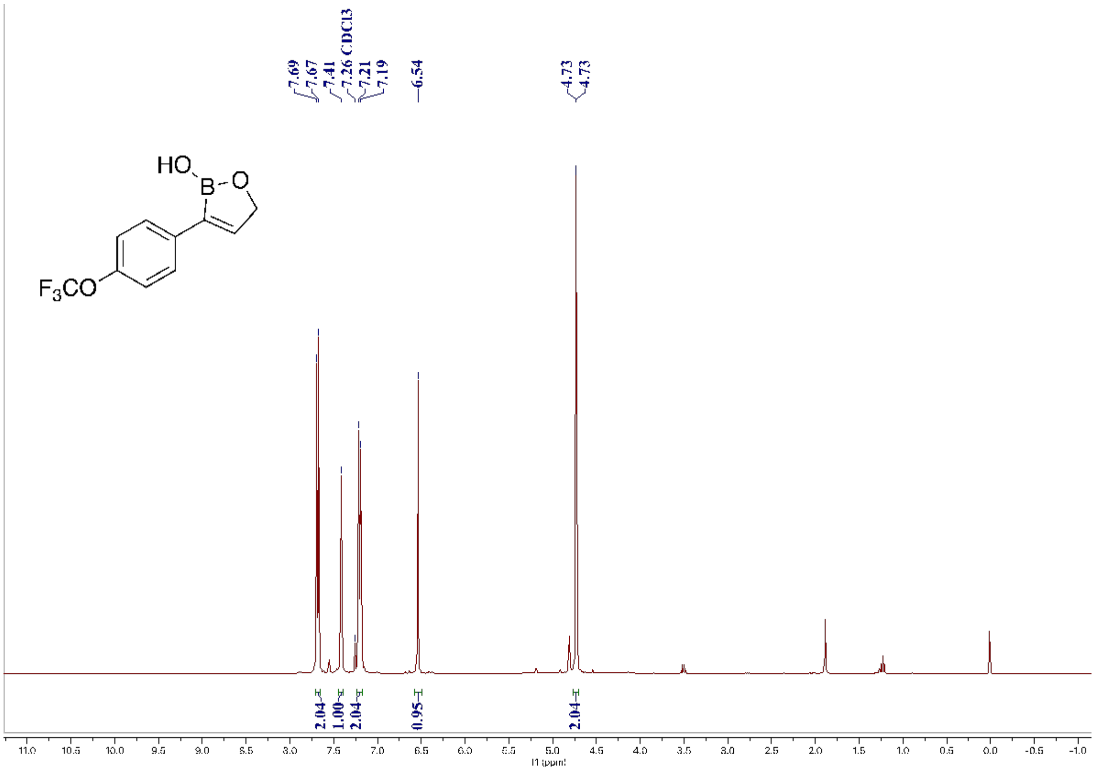

Figure S62. ^{1}H NMR of **6m** ($CDCl_3$, 400 MHz)

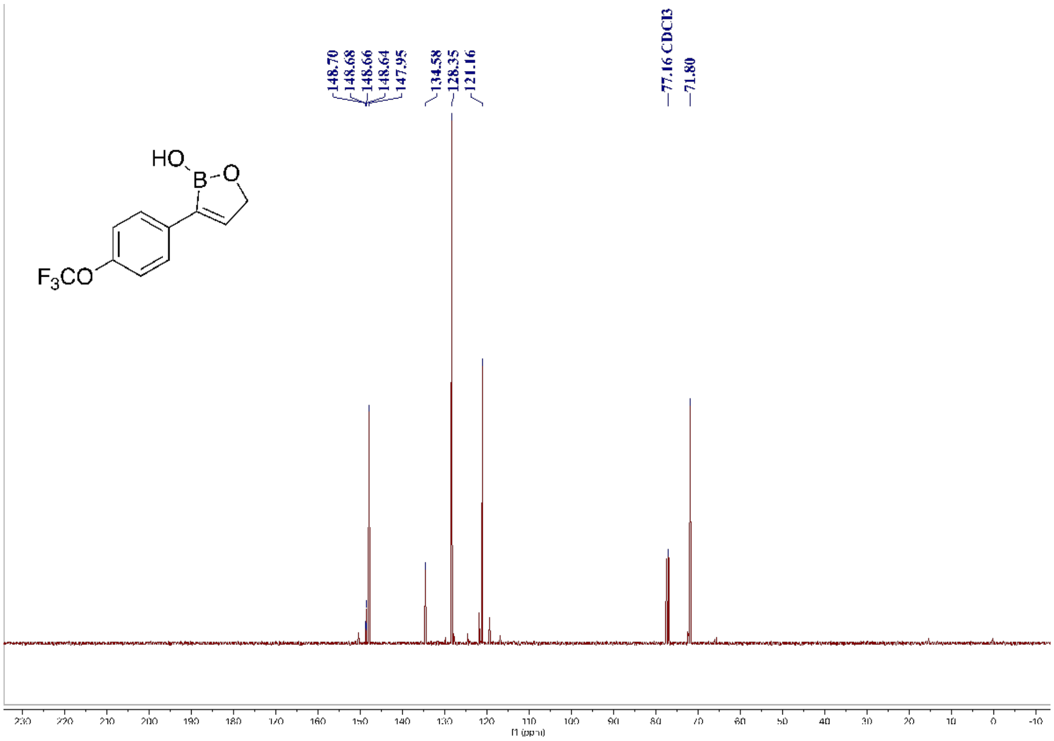

Figure S63. ^{13}C NMR of **6m** ($CDCl_3$, 100 MHz)

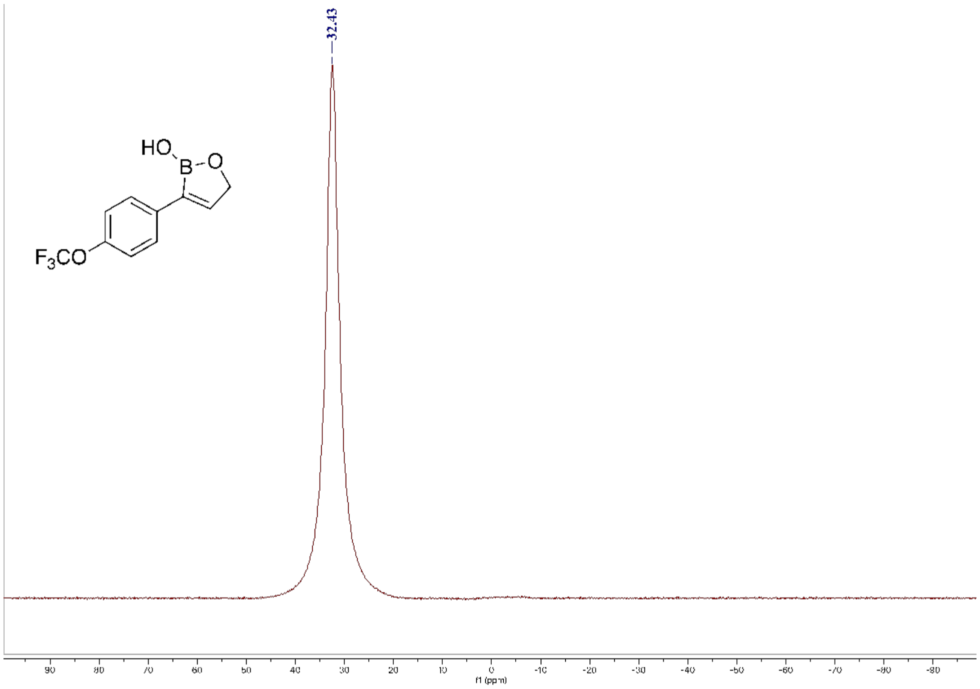

Figure S64. ^{11}B NMR of **6m** ($CDCl_3$, 128 MHz)

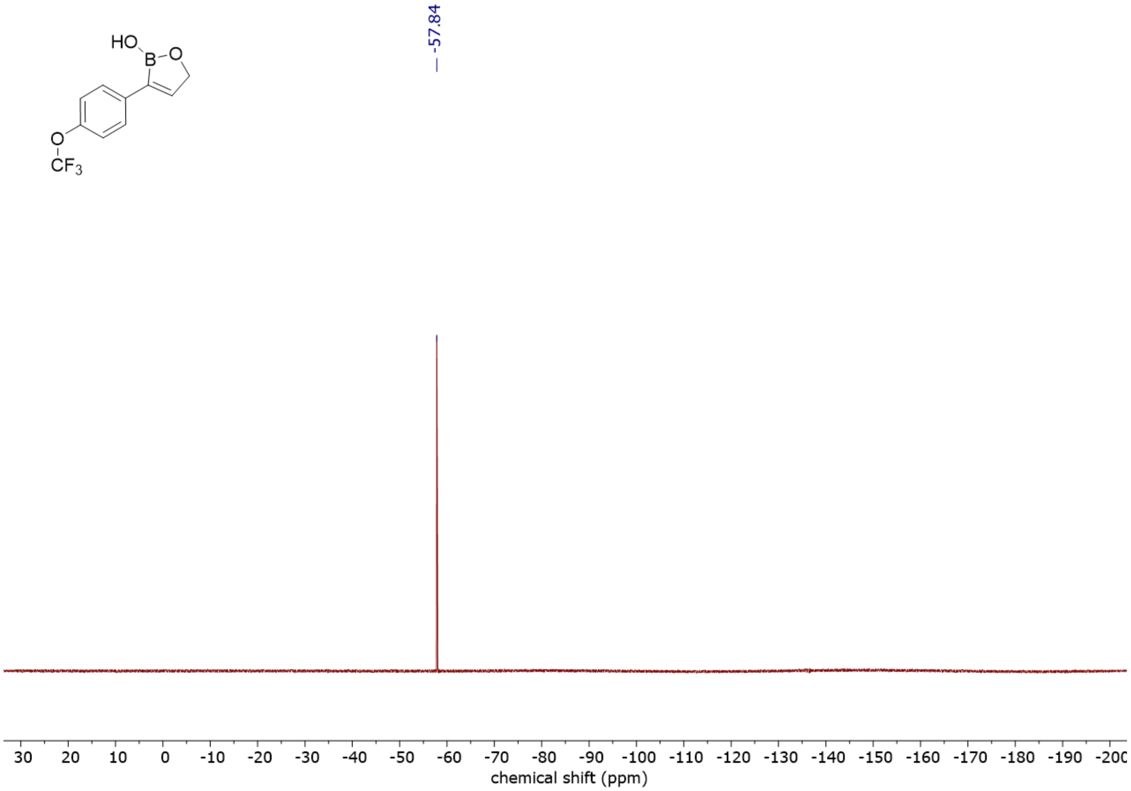

Figure S65. ^{19}F NMR of **6m** ($CDCl_3$, 376 MHz)

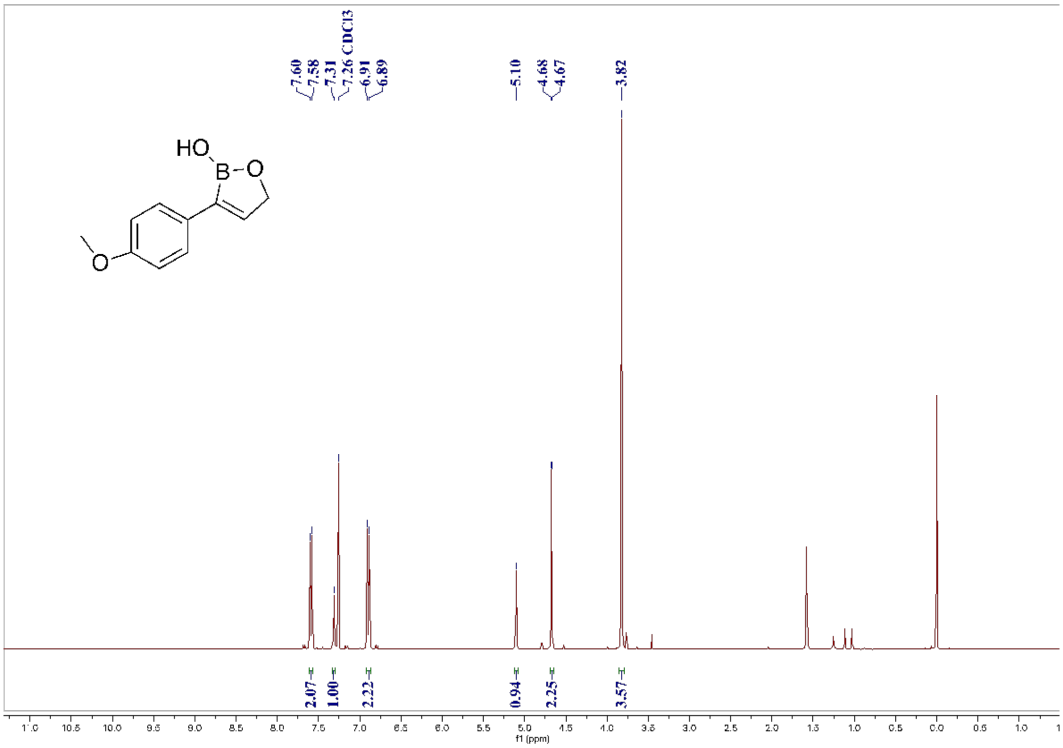

Figure S66. ^{1}H NMR of **6n** ($CDCl_3$, 400 MHz)

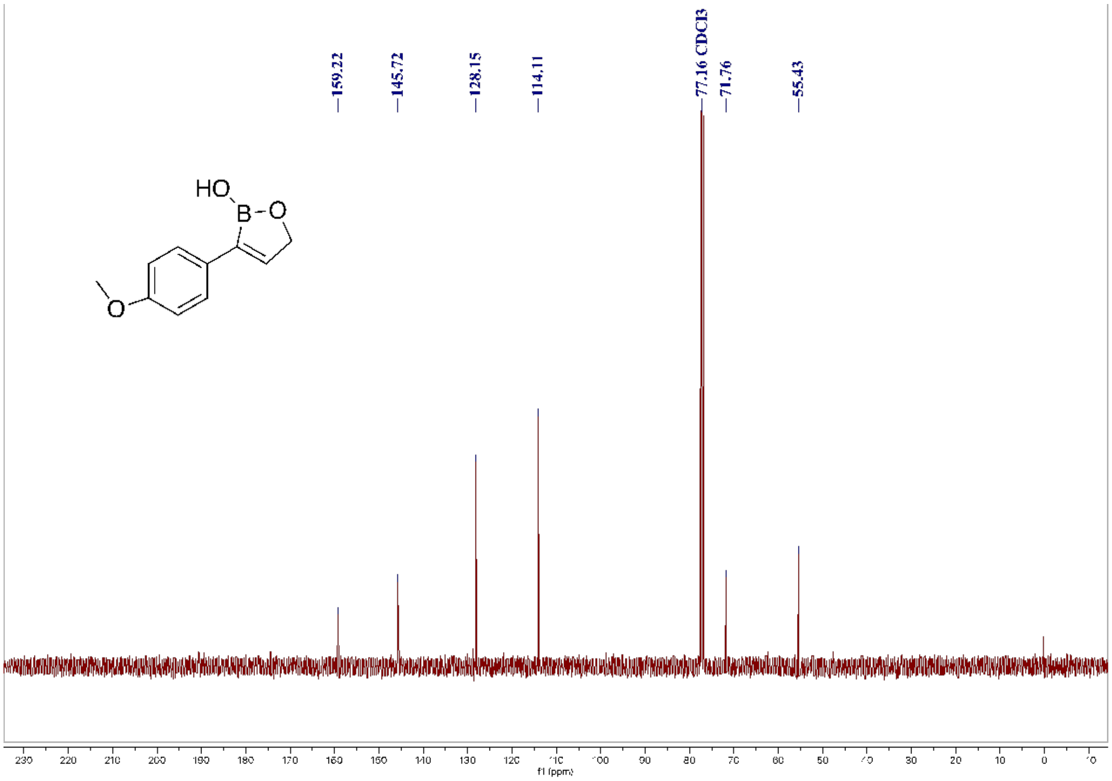

Figure S67. ^{13}C NMR of **6n** ($CDCl_3$, 100 MHz)

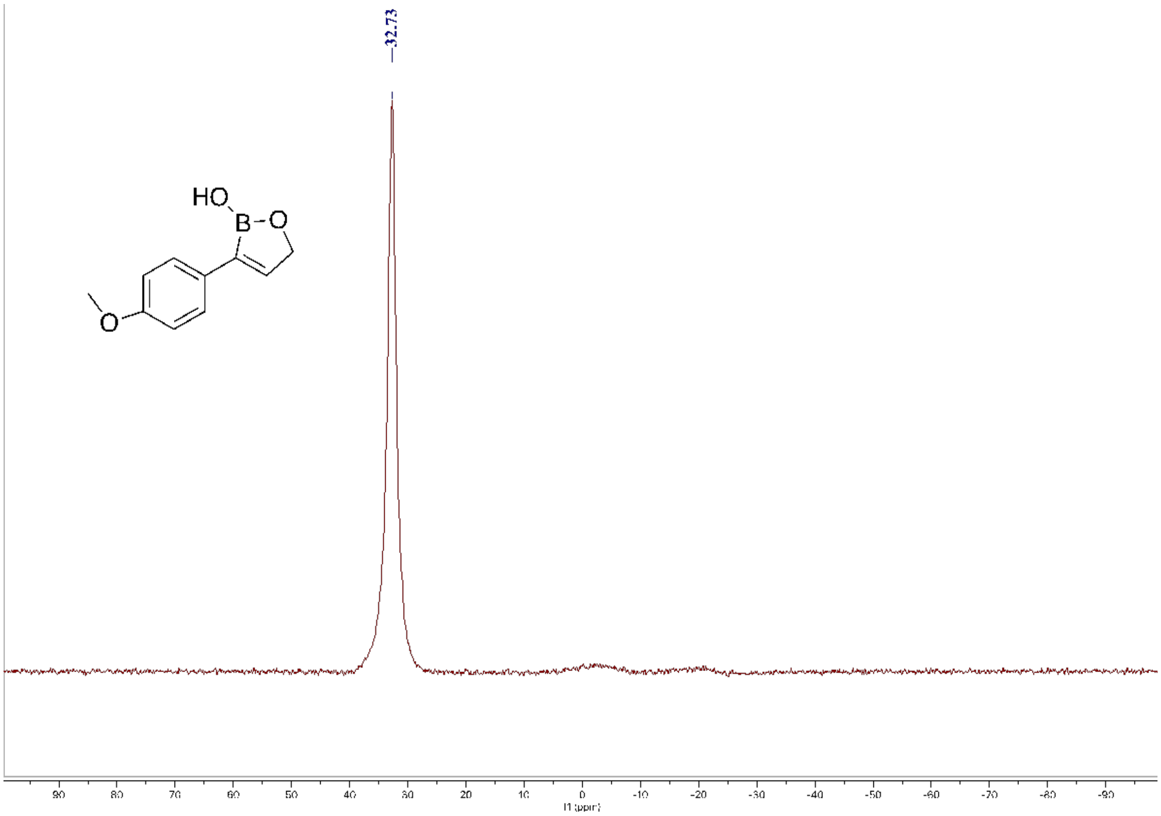

Figure S68. ^{11}B NMR of **6n** ($CDCl_3$, 128 MHz)

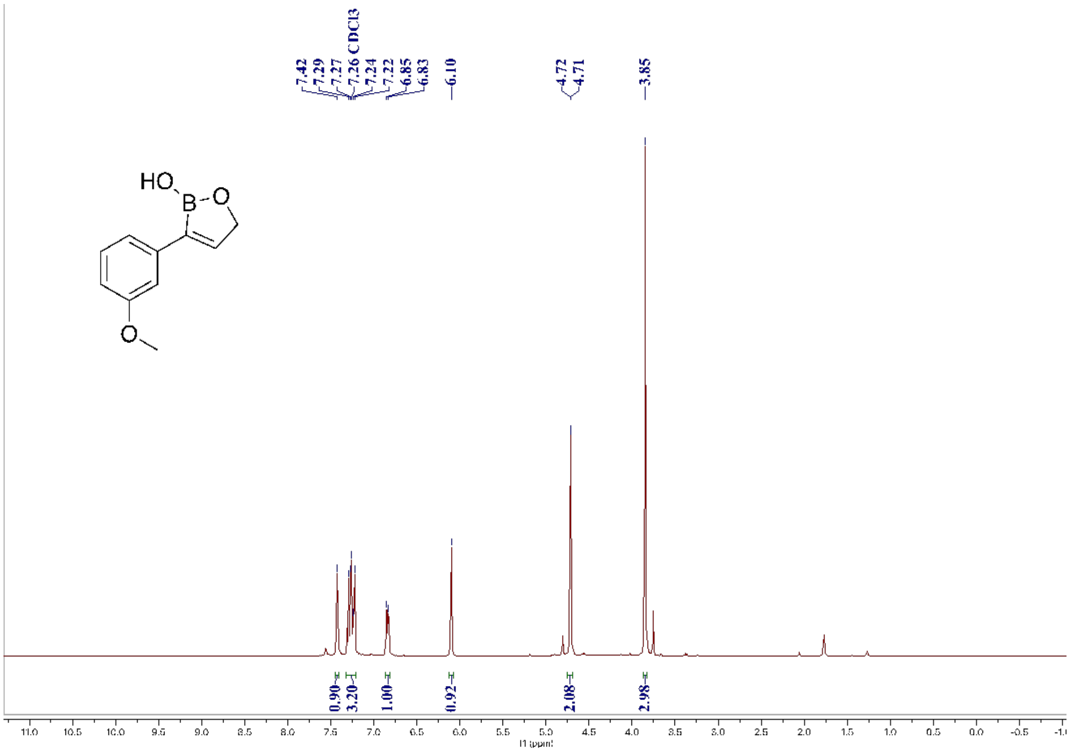

Figure S69. ^{1}H NMR of **6o** ($CDCl_3$, 400 MHz)

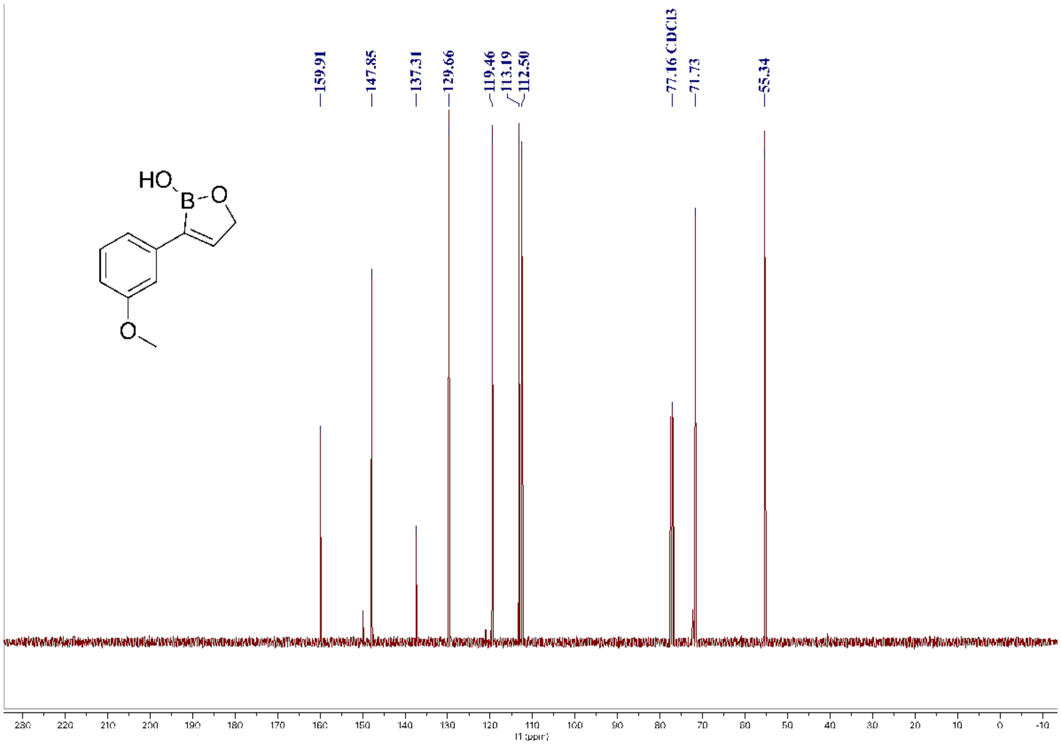

Figure S70. ^{13}C NMR of **6o** ($CDCl_3$, 100 MHz)

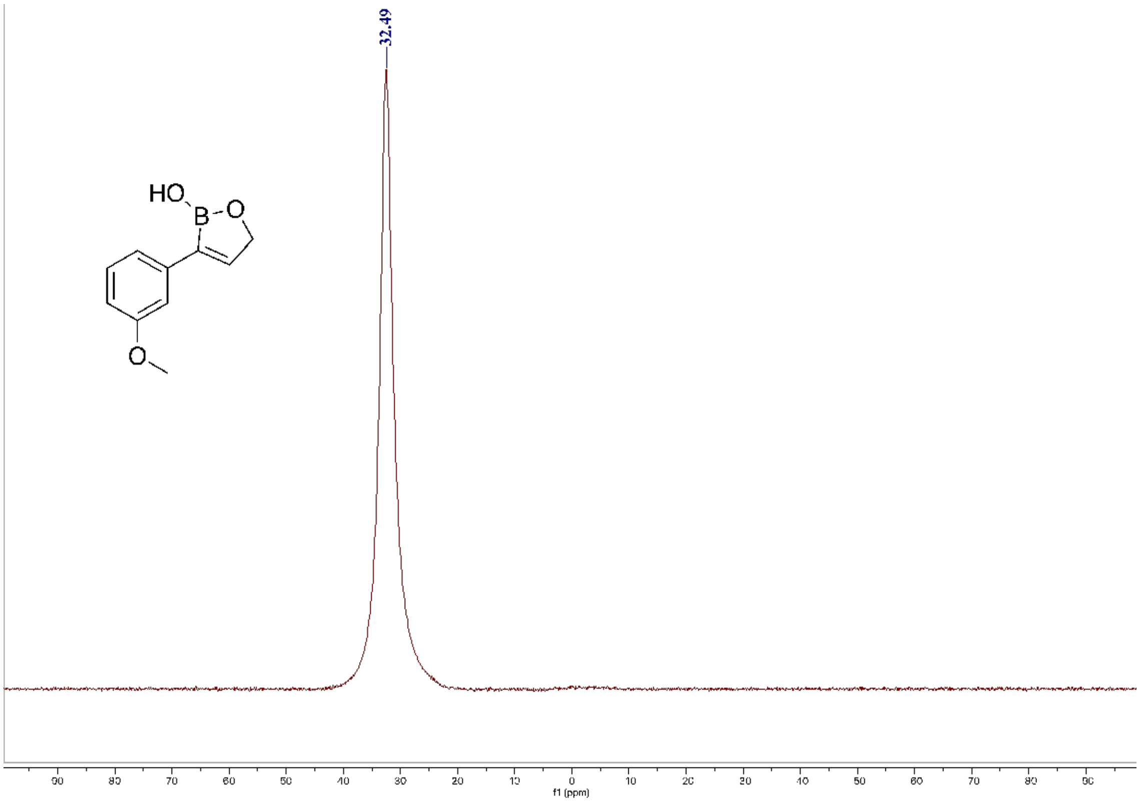

Figure S71. ^{11}B NMR of **6o** ($CDCl_3$, 128 MHz)

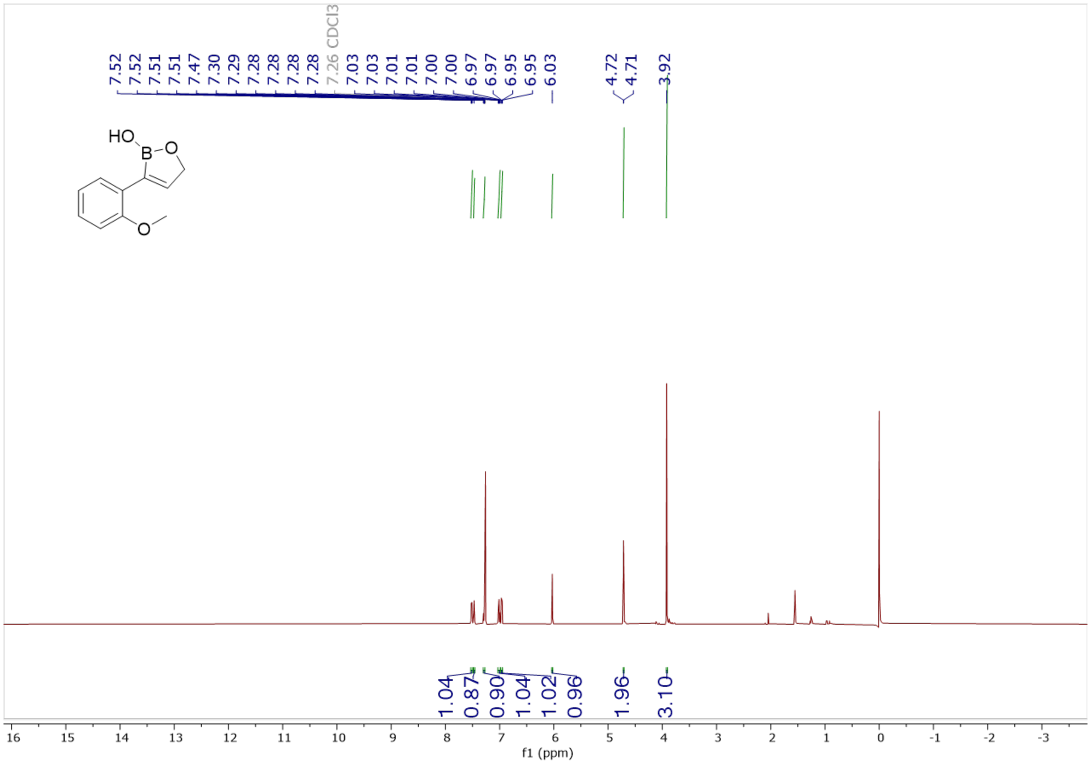

Figure S72. ^{1}H NMR of **6p** ($CDCl_3$, 500 MHz)

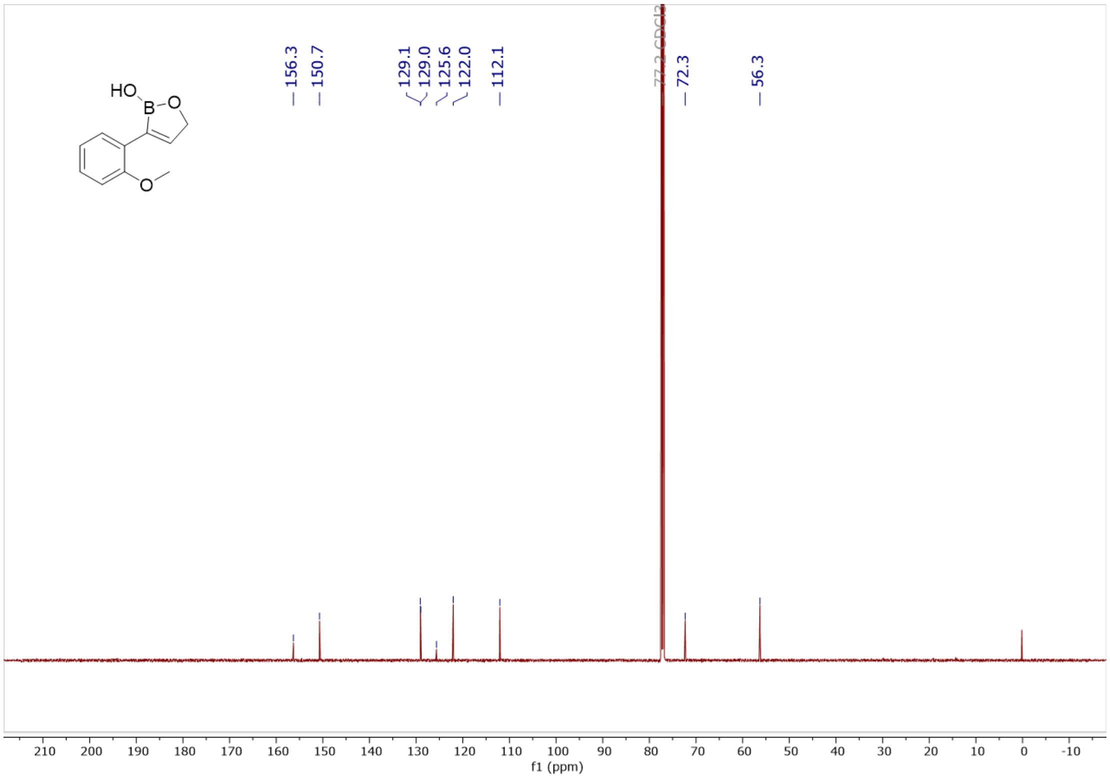

Figure S73. ^{13}C NMR of **6p** ($CDCl_3$, 126 MHz)

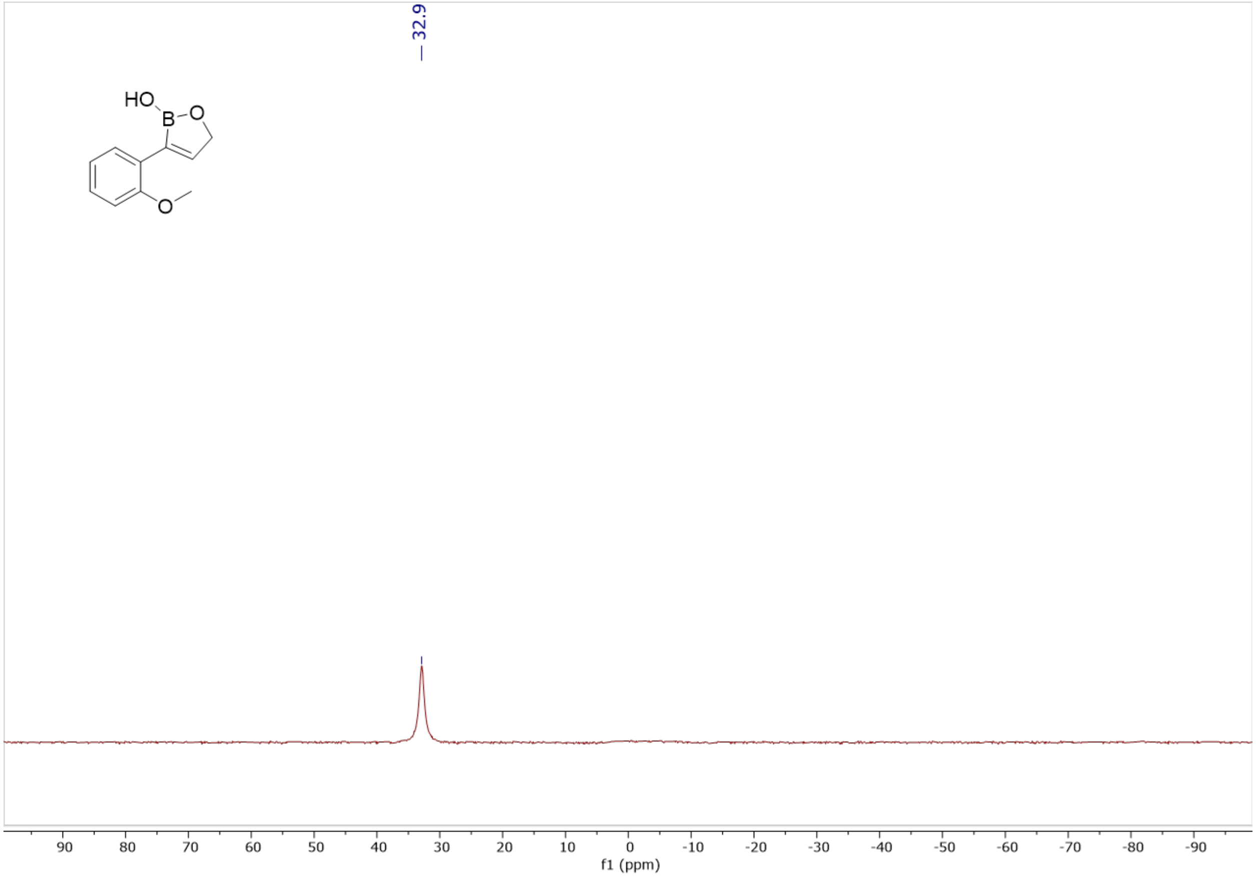

Figure S74. ^{11}B NMR of **6p** ($CDCl_3$, 128 MHz)

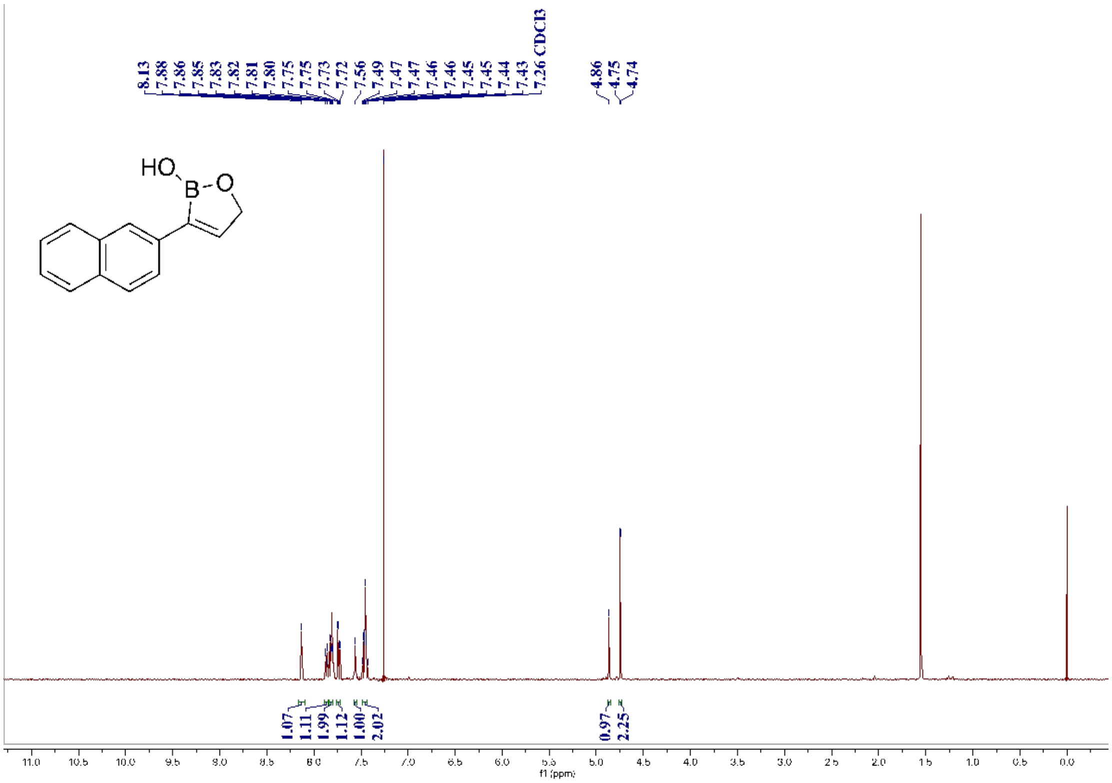

Figure S75. ^{1}H NMR of **6q** ($CDCl_3$, 400 MHz)

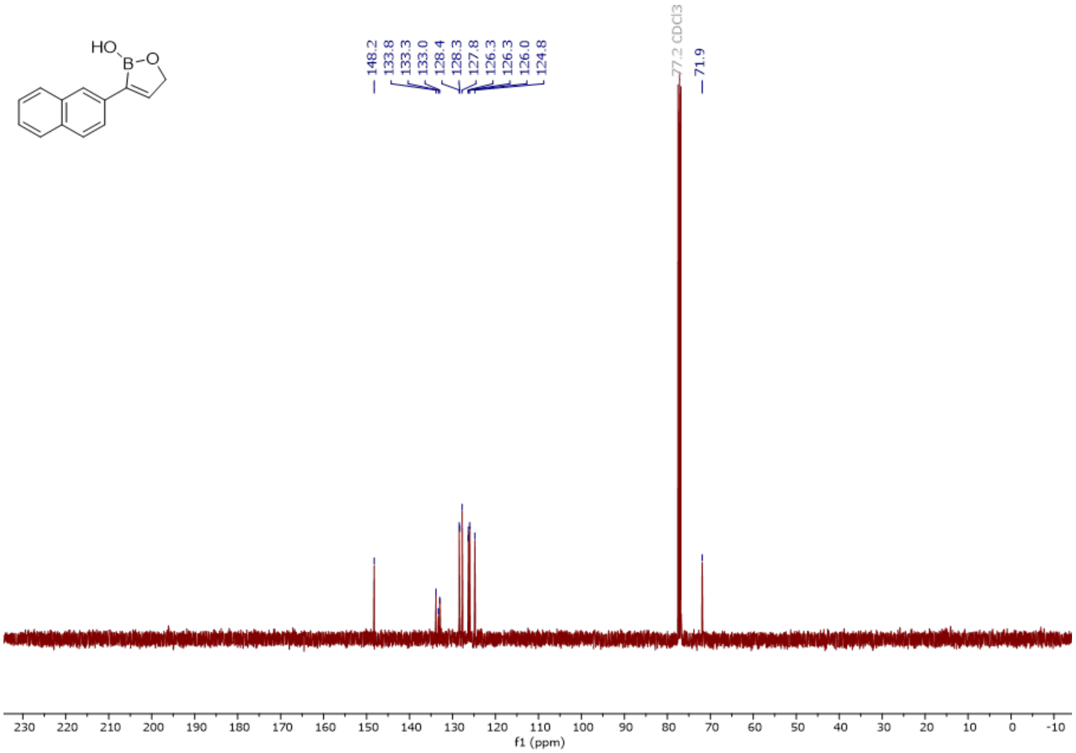

Figure S76. ^{13}C NMR of **6q** ($CDCl_3$, 100 MHz)

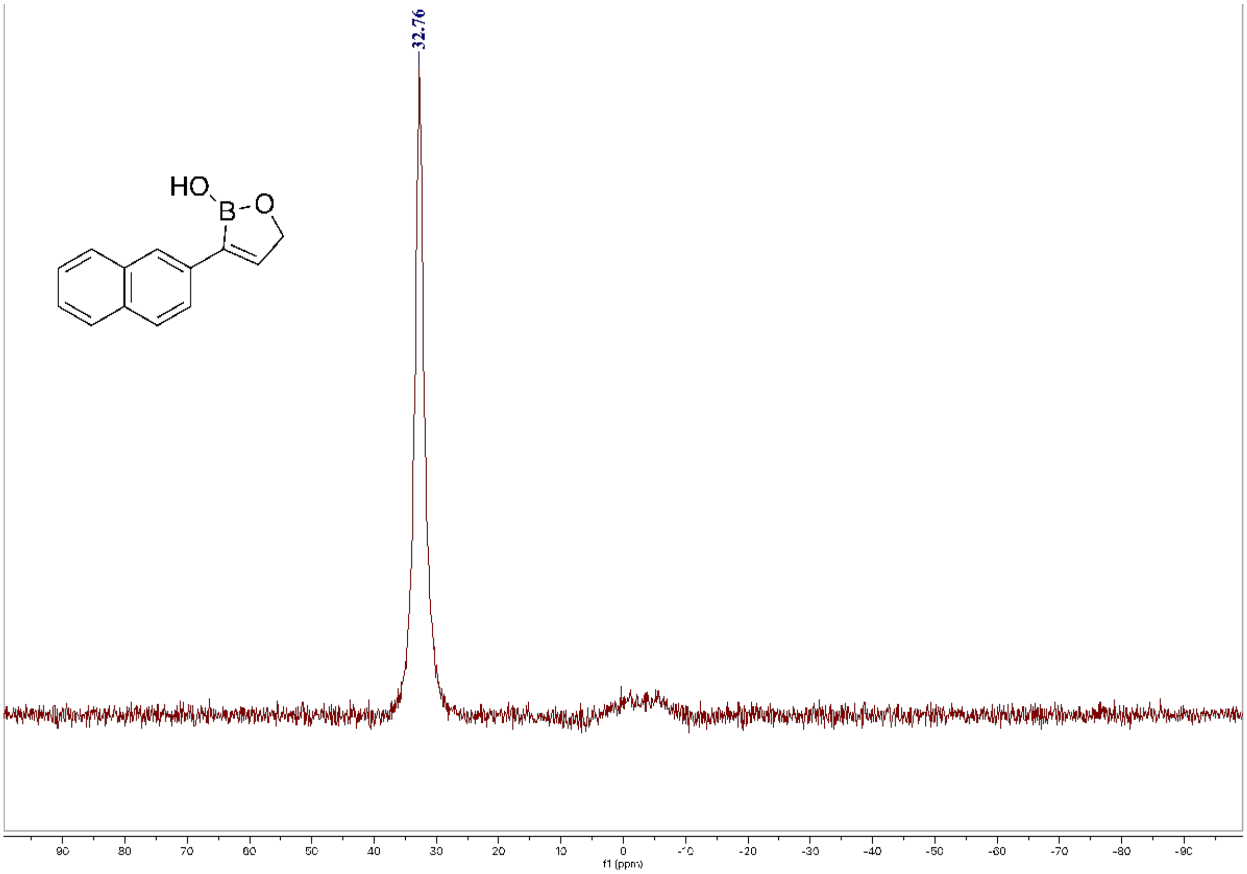

Figure S77. ^{11}B NMR of **6q** ($CDCl_3$, 128 MHz)

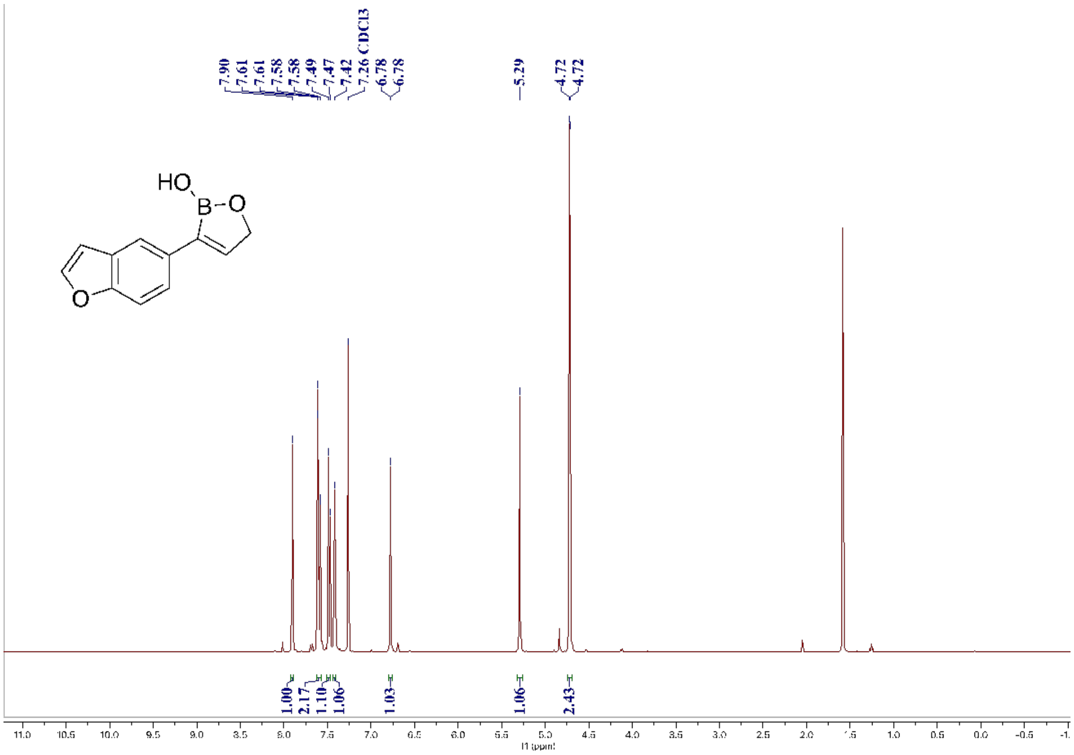

Figure S78. ^{1}H NMR of **6r** ($CDCl_3$, 400 MHz)

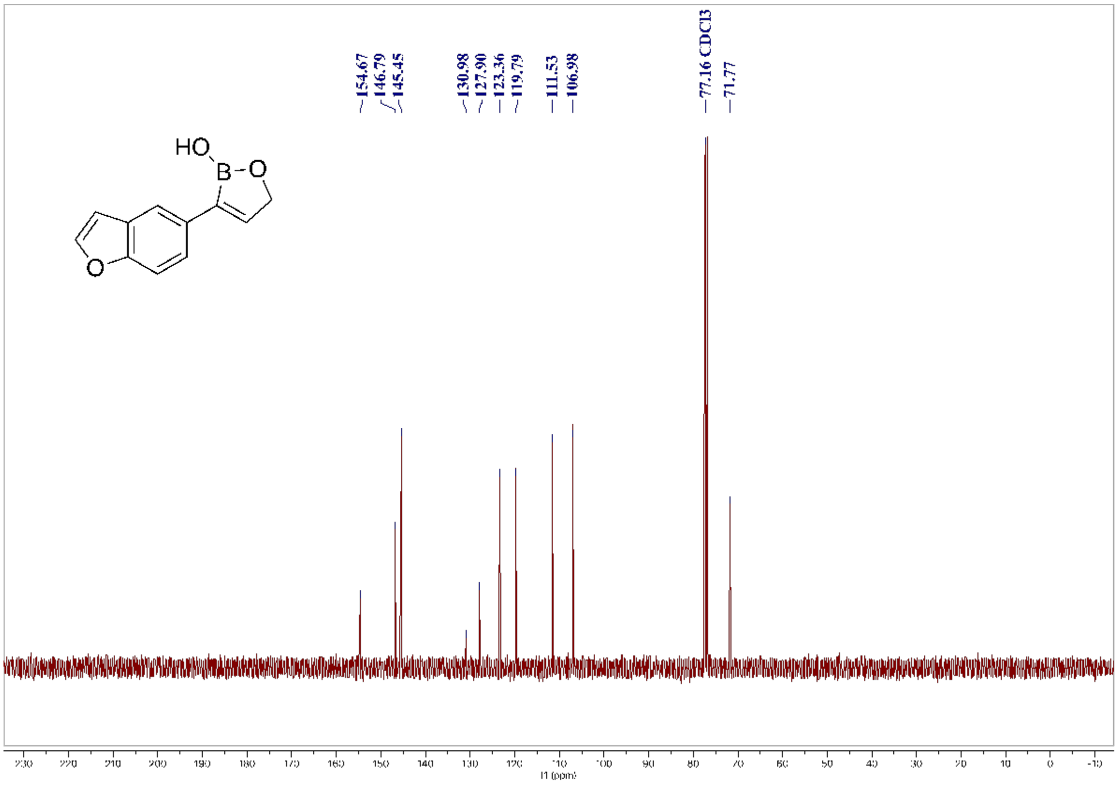

Figure S79. ^{13}C NMR of **6r** ($CDCl_3$, 100 MHz)

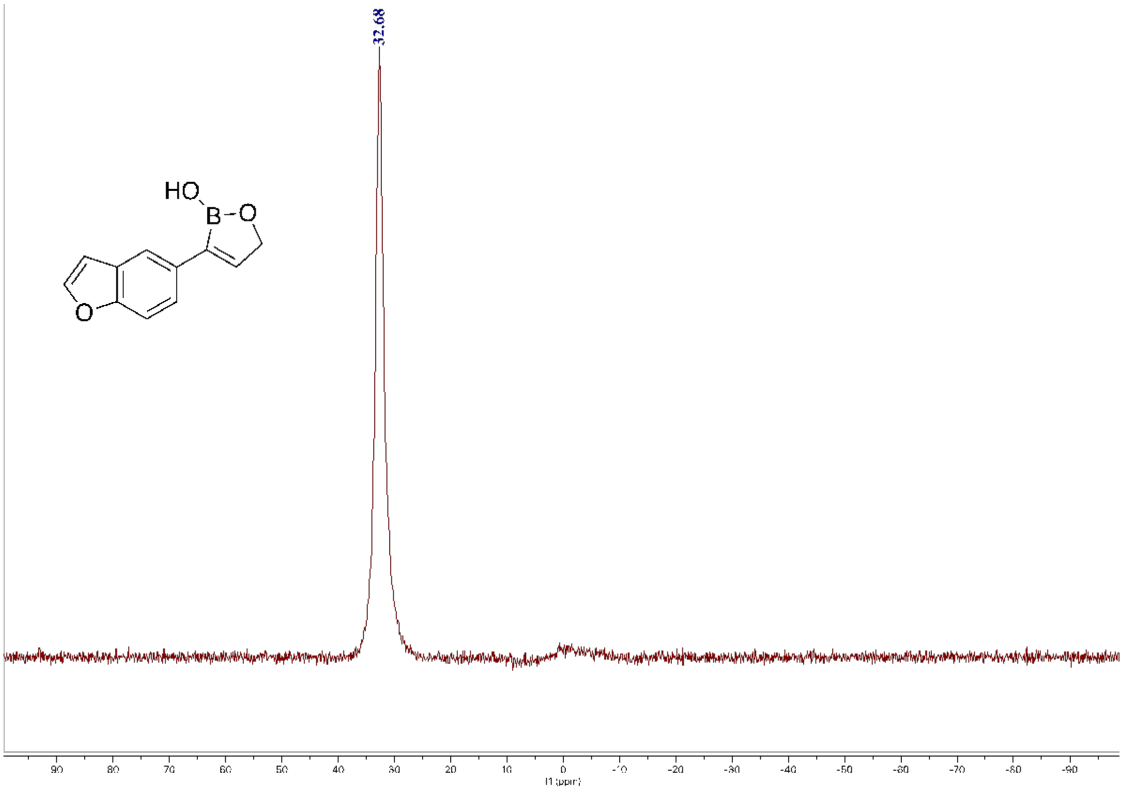

Figure S80. ^{11}B NMR of **6r** ($CDCl_3$, 128 MHz)

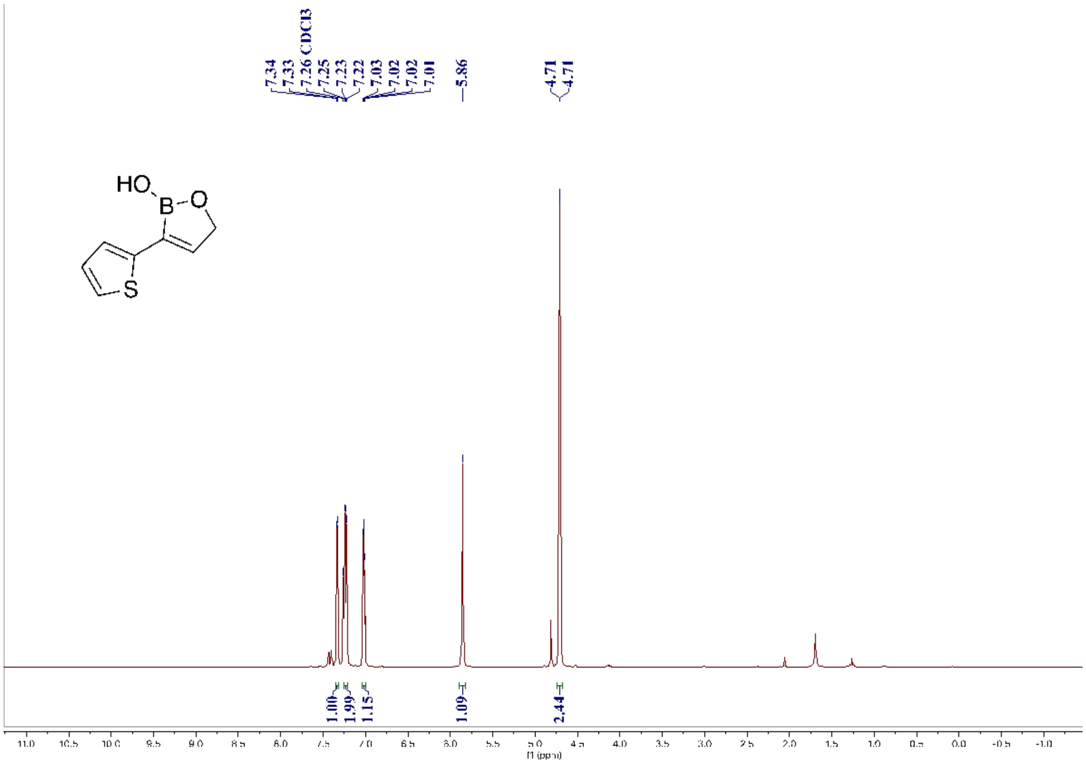

Figure S81. ^{1}H NMR of **6s** ($CDCl_3$, 400 MHz)

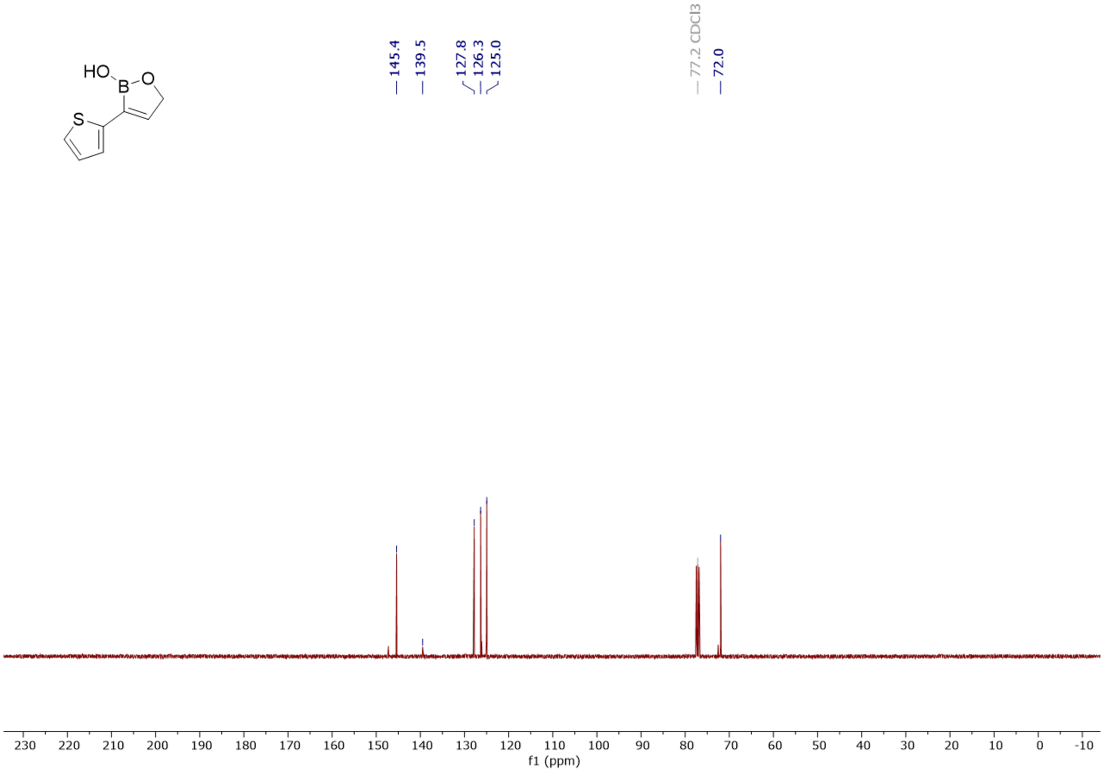

Figure S82. ^{13}C NMR of **6s** ($CDCl_3$, 100 MHz)

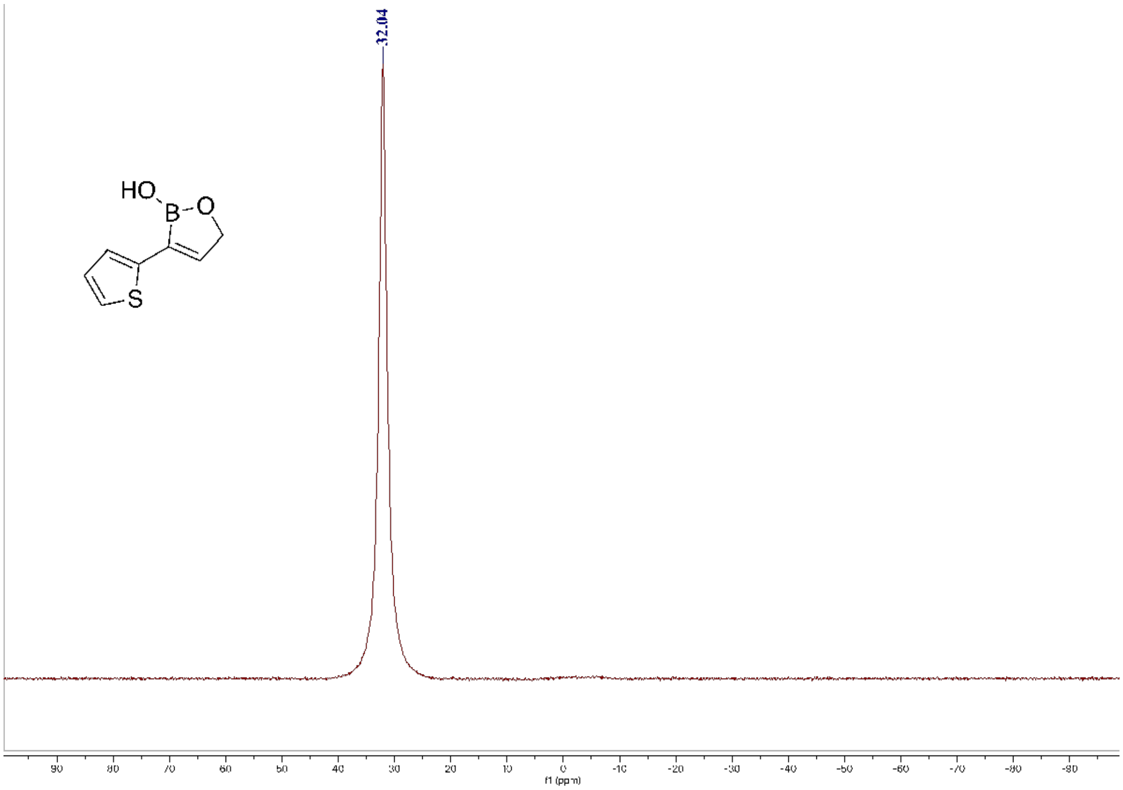

Figure S83. ^{11}B NMR of **6s** ($CDCl_3$, 128 MHz)

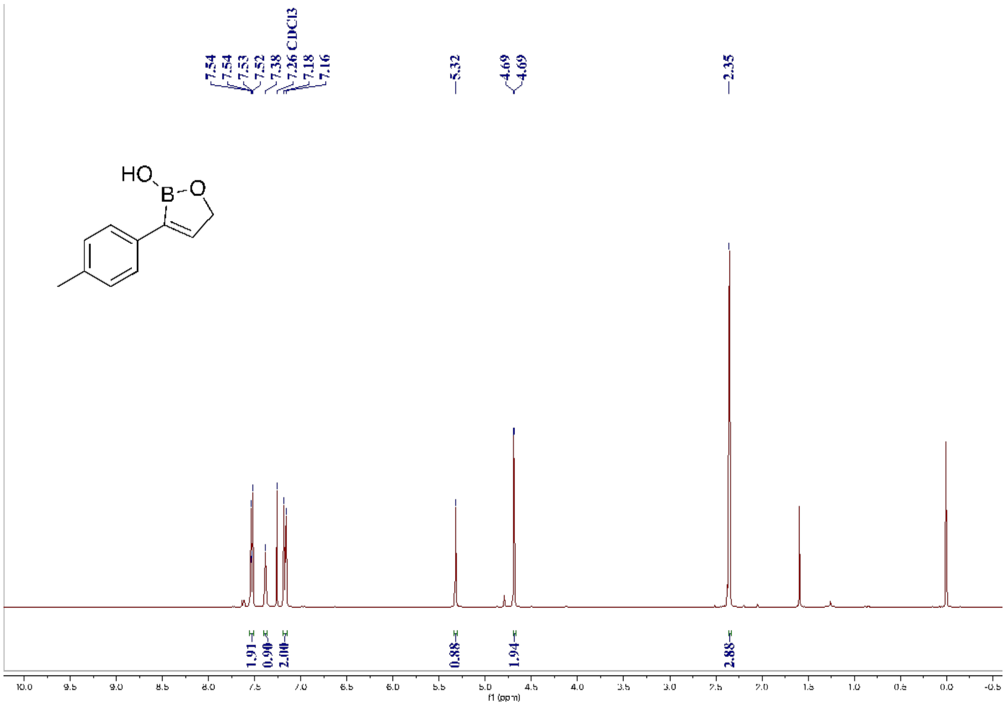

Figure S84. ^{1}H NMR of **6t** ($CDCl_3$, 400 MHz)

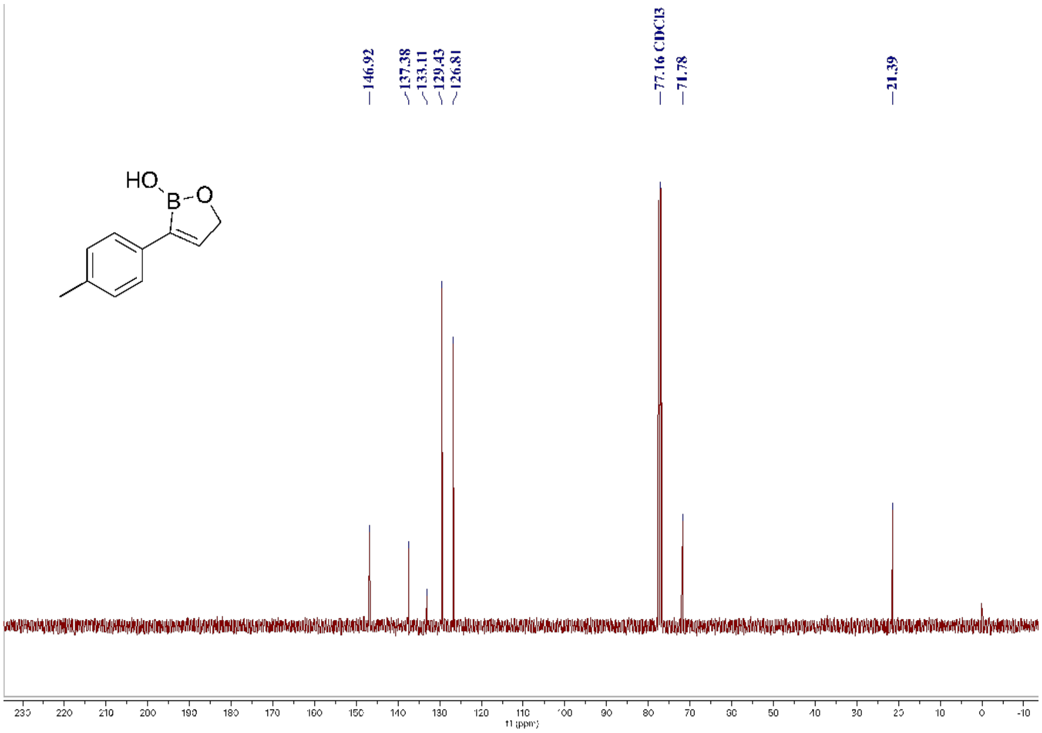

Figure S85. ^{13}C NMR of **6t** ($CDCl_3$, 100 MHz)

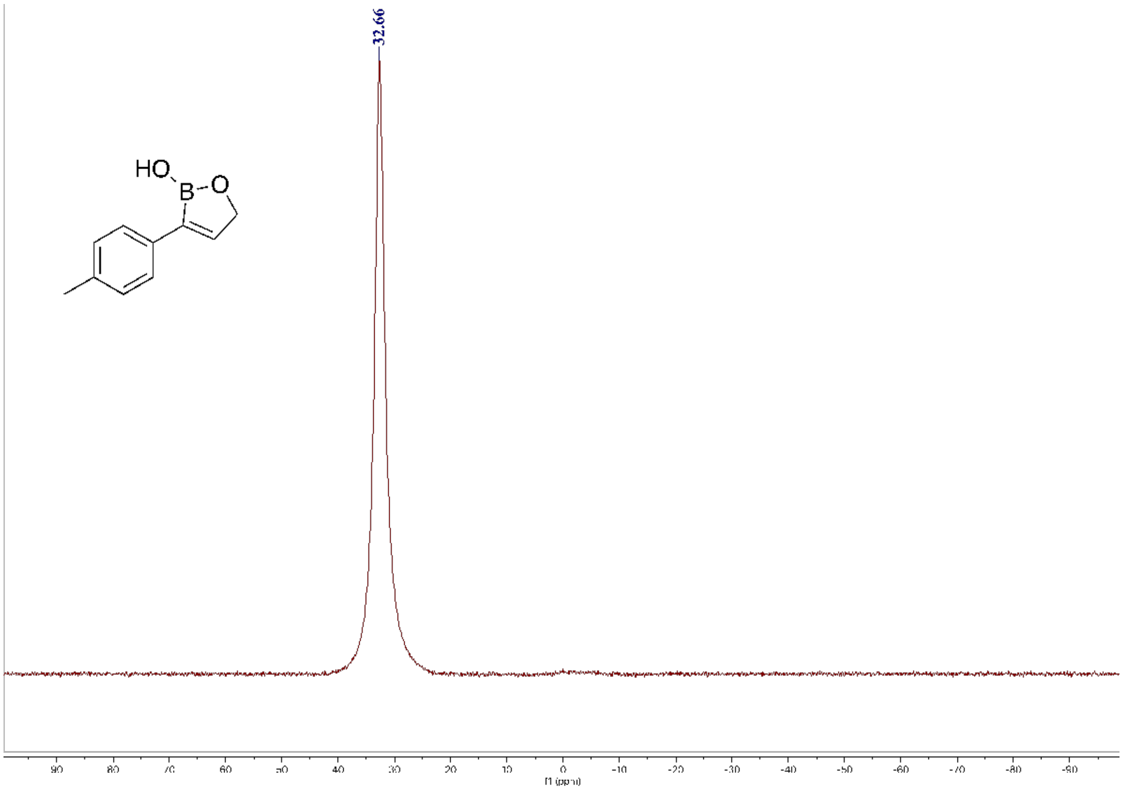

Figure S86. ^{11}B NMR of **6t** ($CDCl_3$, 128 MHz)

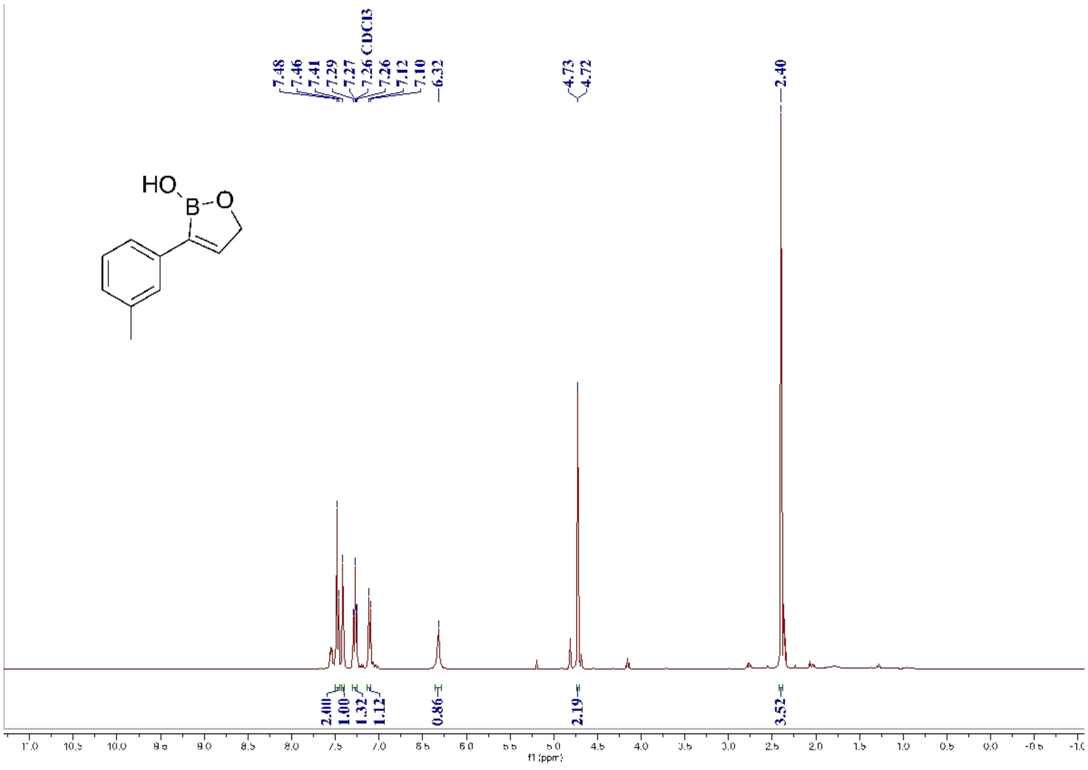

Figure S87. ^{1}H NMR of **6u** ($CDCl_3$, 400 MHz)

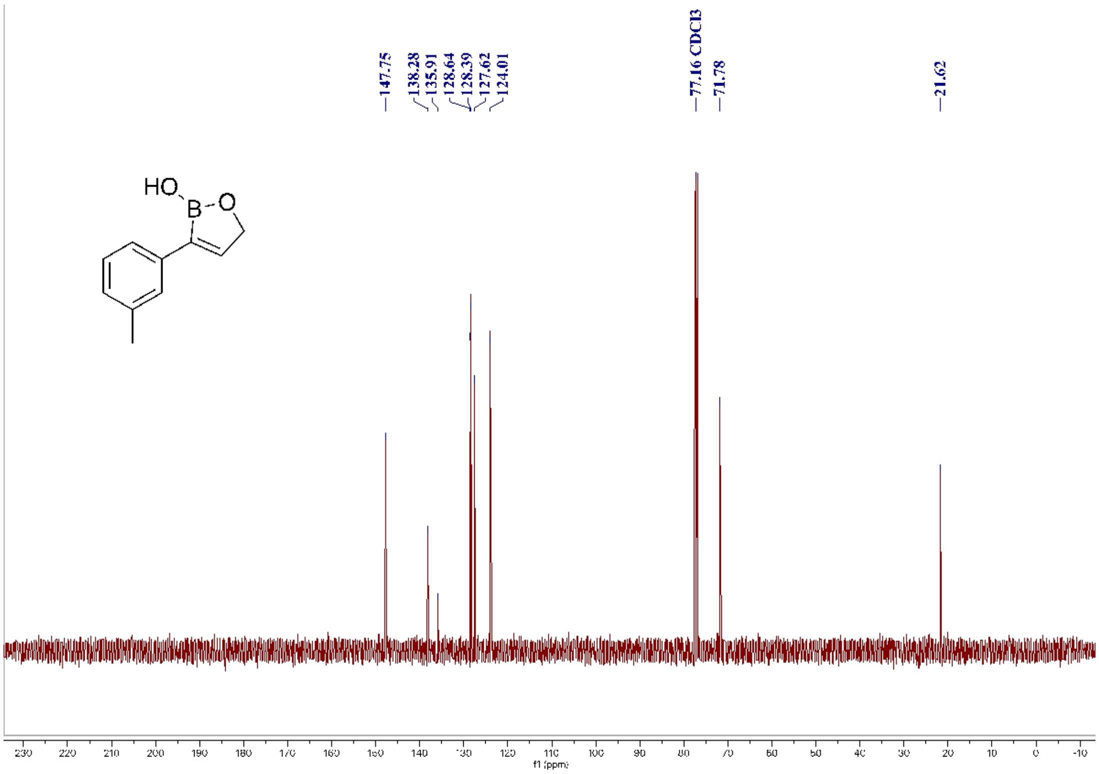

Figure S88. ^{13}C NMR of **6u** ($CDCl_3$, 100 MHz)

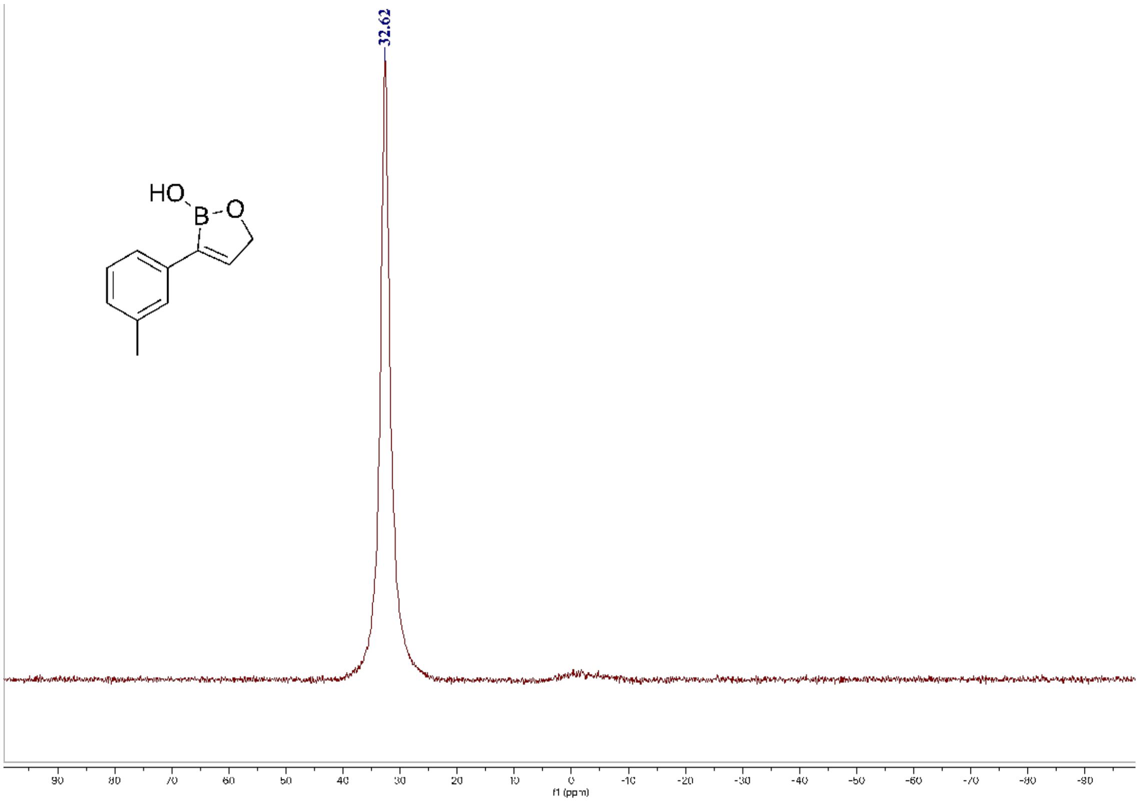

Figure S89. ^{11}B NMR of **6u** ($CDCl_3$, 128 MHz)

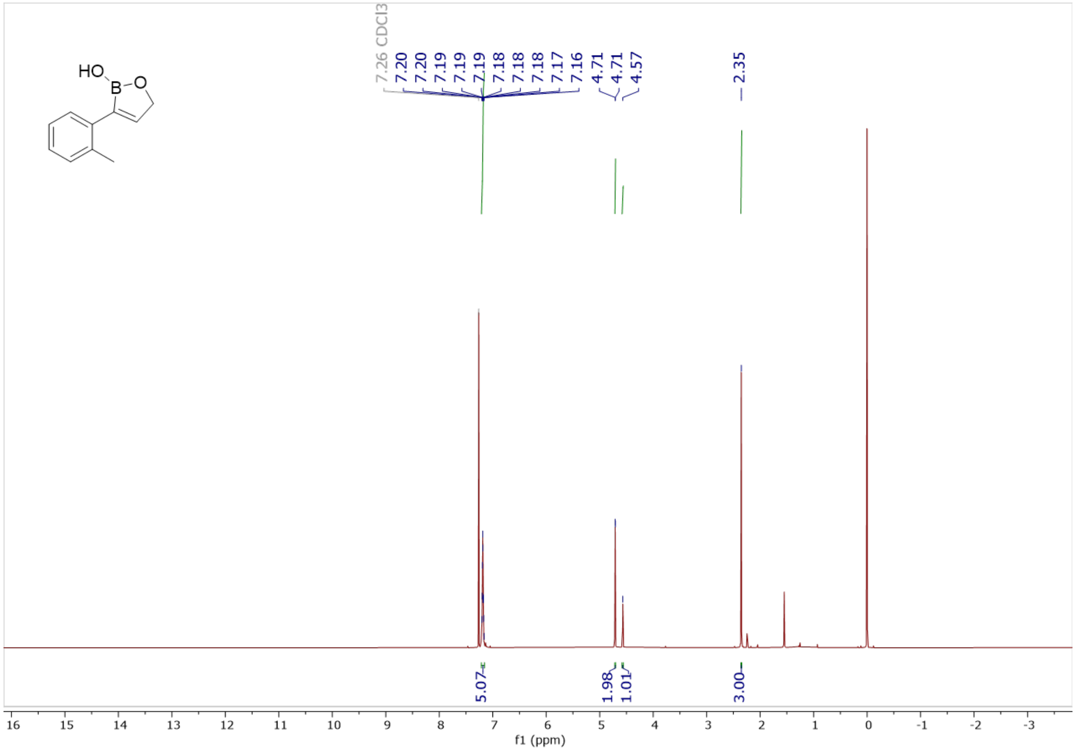

Figure S90. ^{1}H NMR of **6v** ($CDCl_3$, 500 MHz)

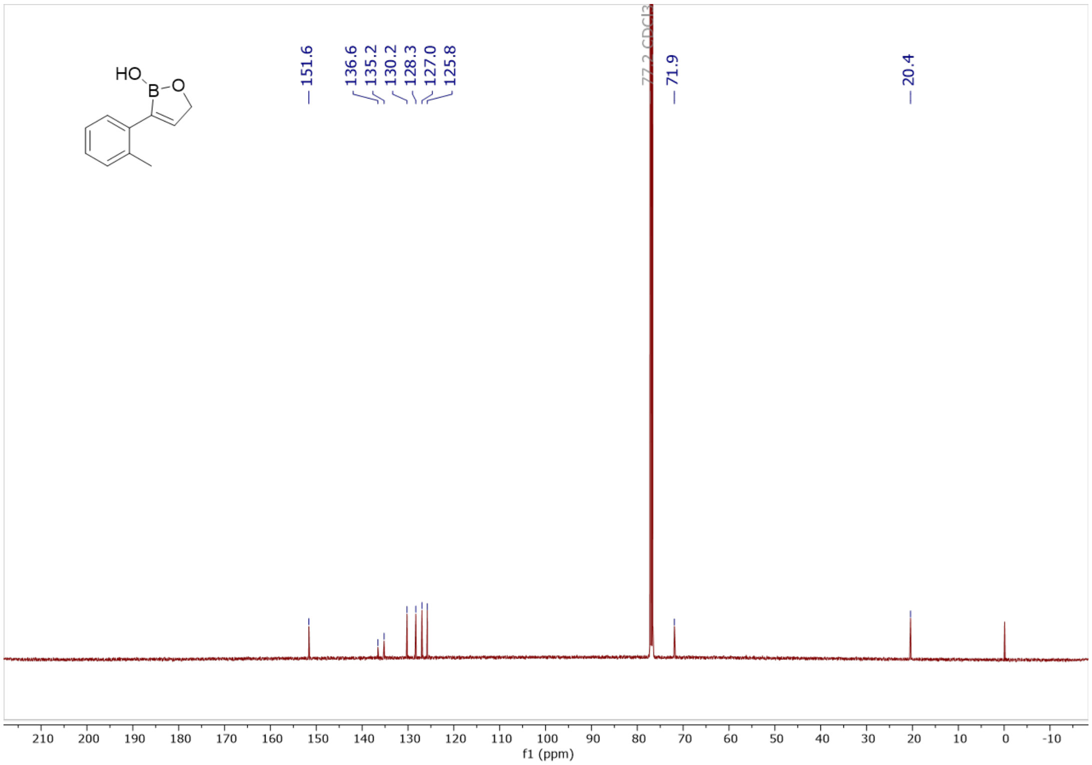

Figure S91. ^{13}C NMR of **6v** ($CDCl_3$, 126 MHz)

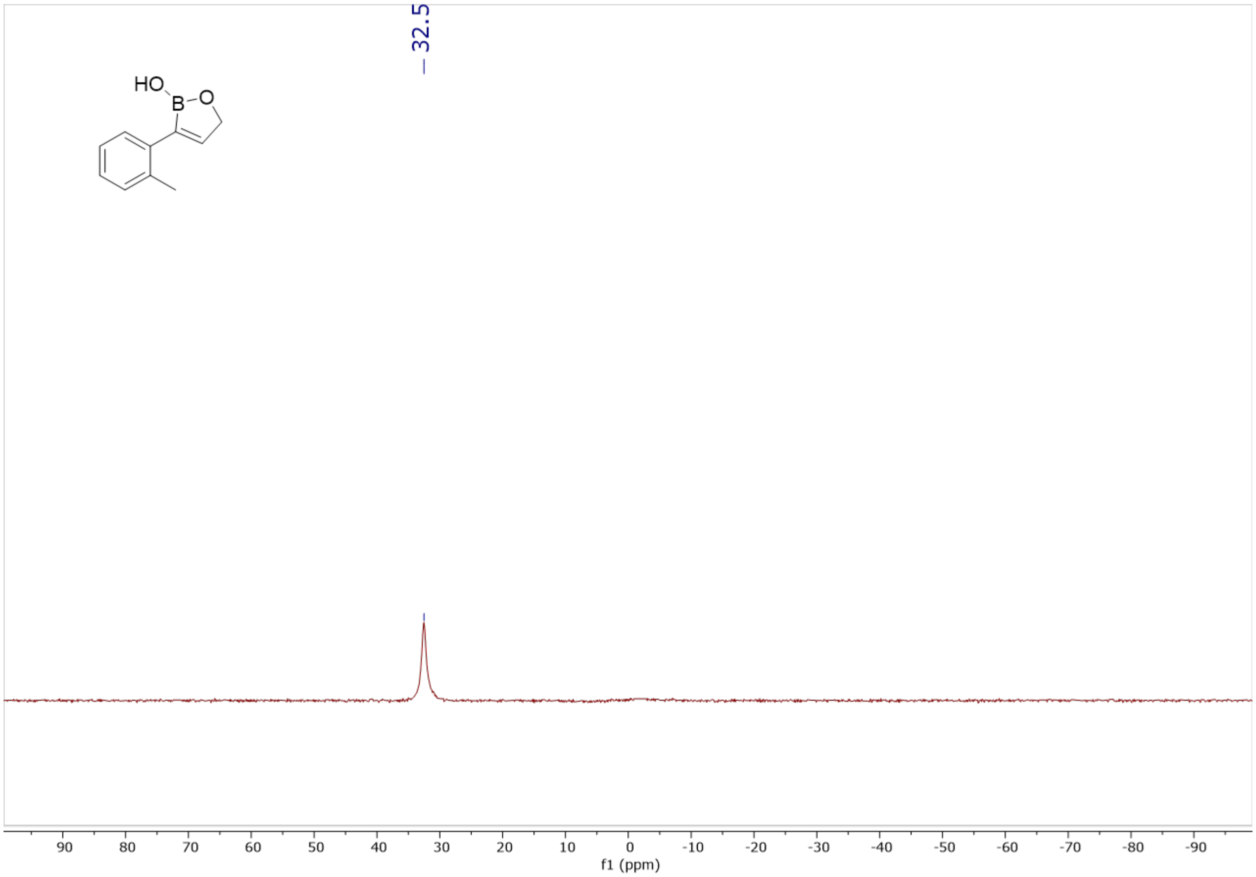

Figure S92. ^{11}B NMR of **6v** ($CDCl_3$, 128 MHz)

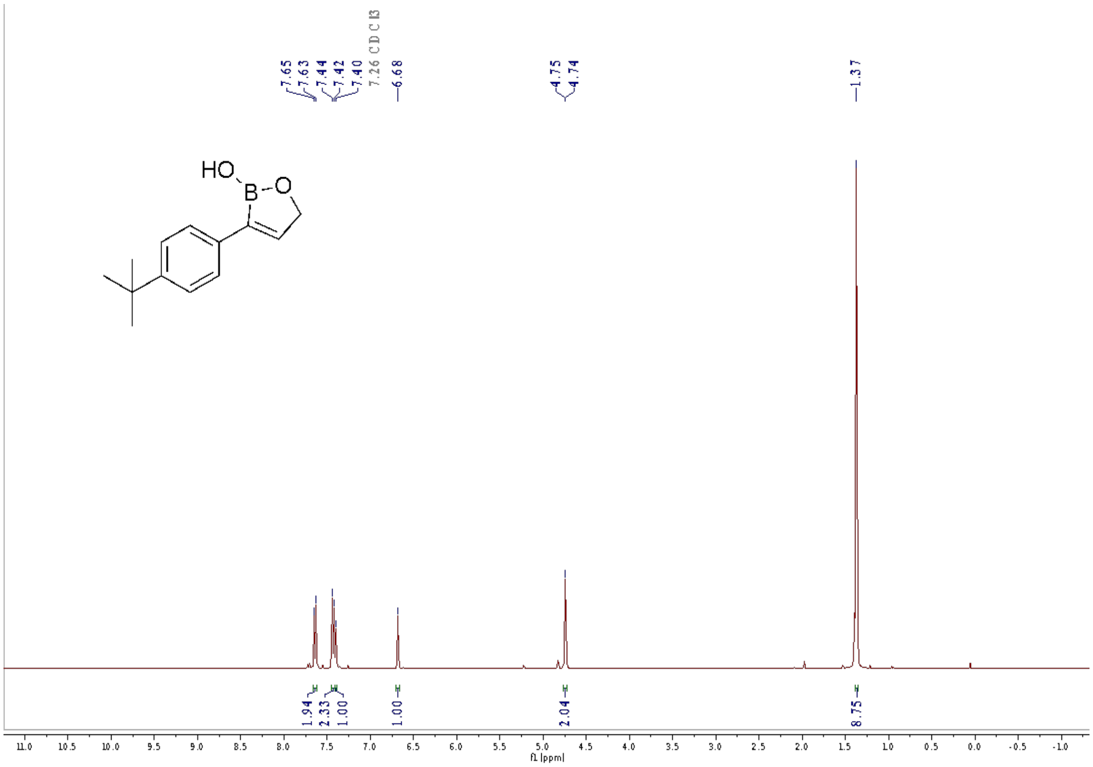

Figure S93. ^{1}H NMR of **6w** ($CDCl_3$, 400 MHz)

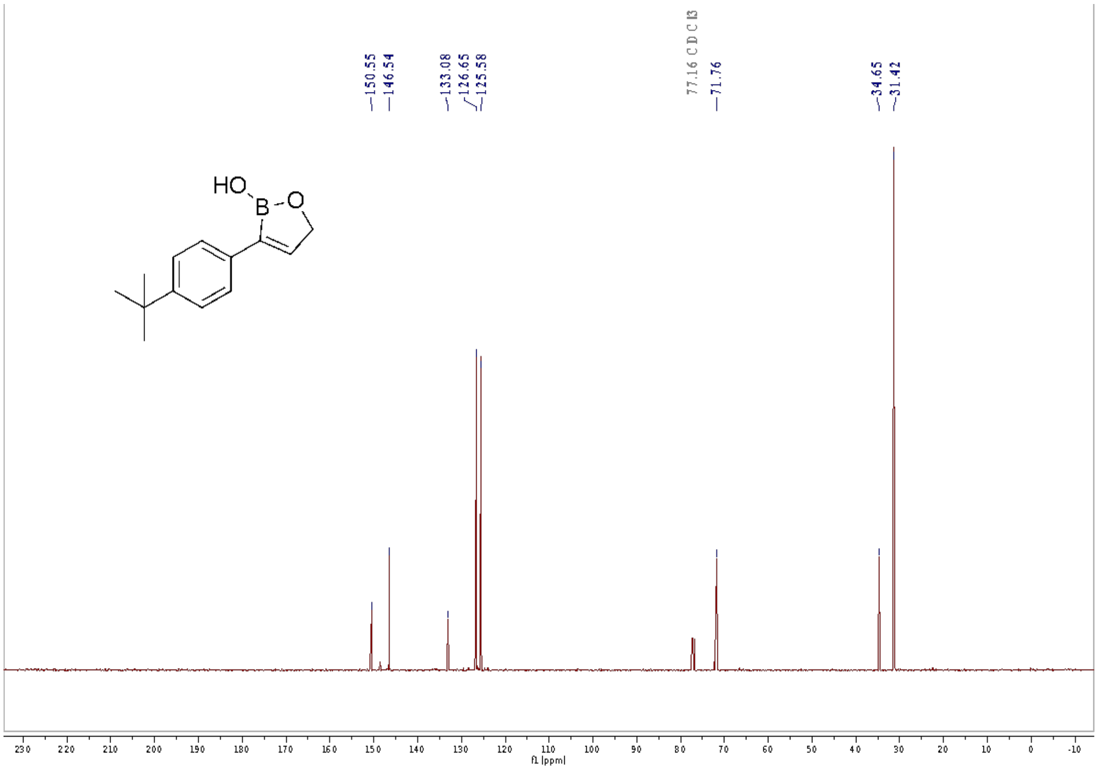

Figure S94. ^{13}C NMR of **6w** ($CDCl_3$, 100 MHz)

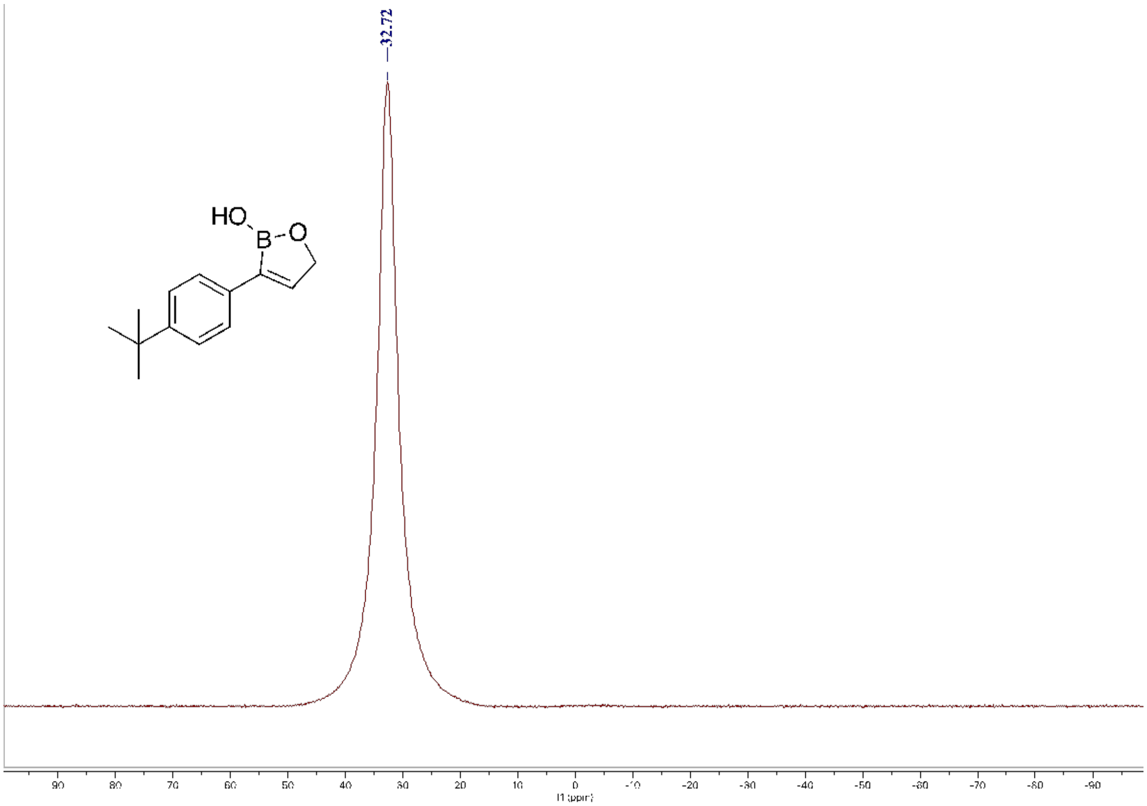

Figure S95. ^{11}B NMR of **6w** ($CDCl_3$, 128 MHz)

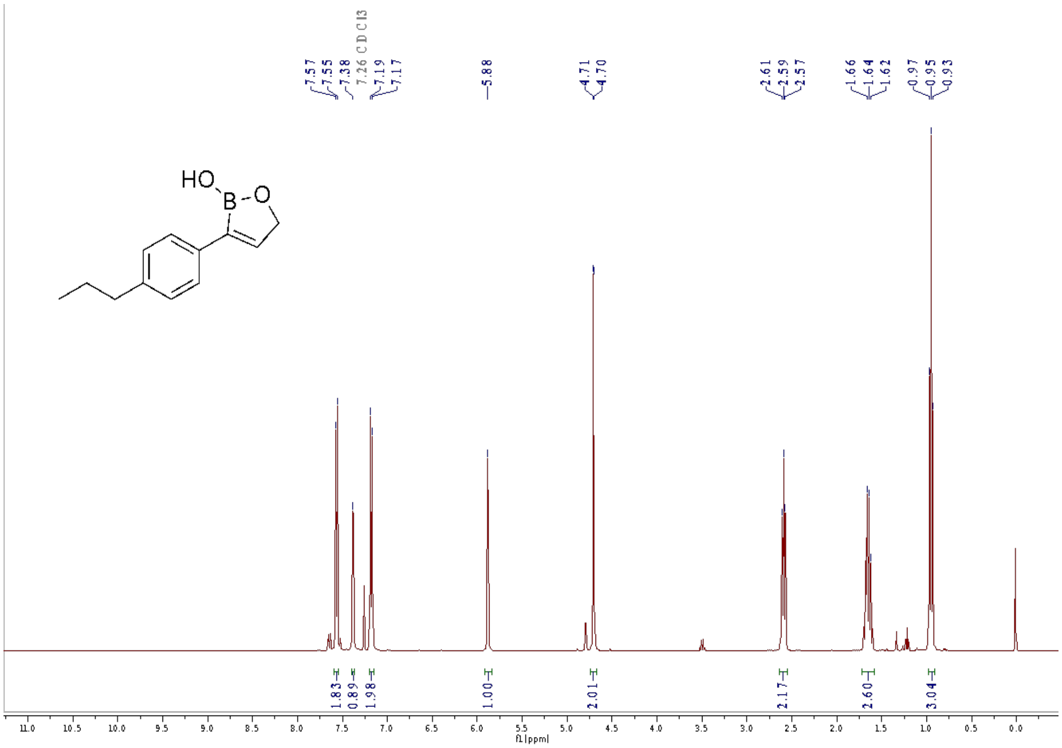

Figure S96. ^{1}H NMR of **6x** ($CDCl_3$, 400 MHz)

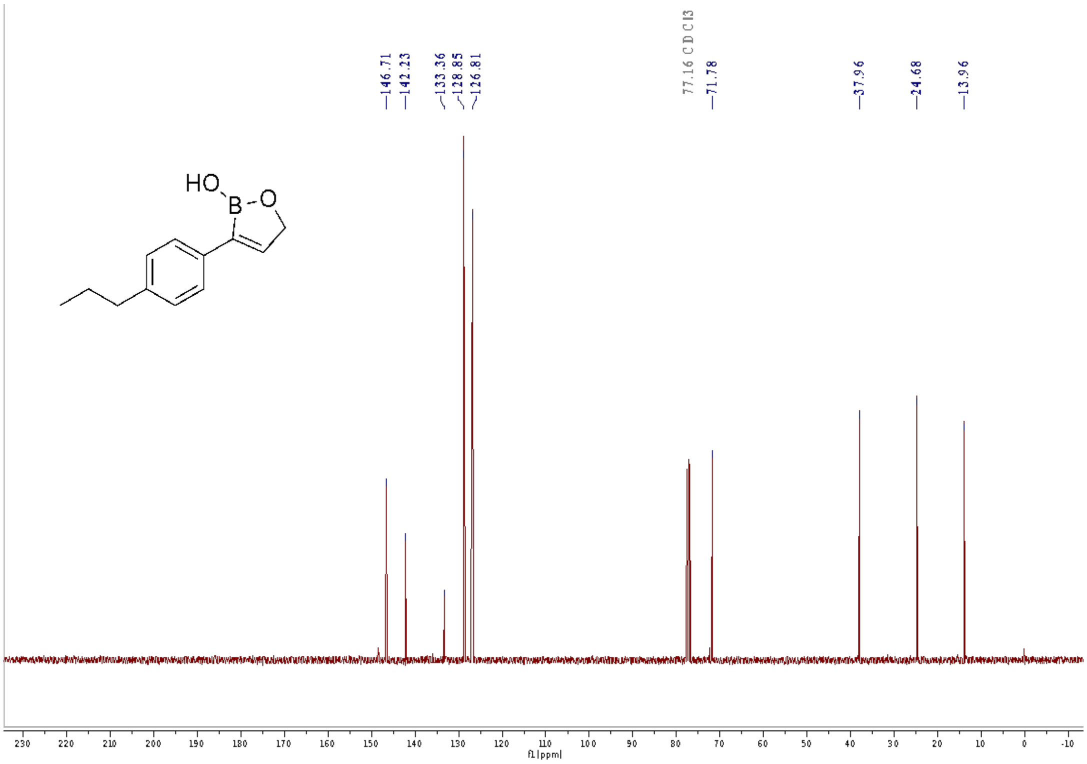

Figure S97. ^{13}C NMR of **6x** ($CDCl_3$, 100 MHz)

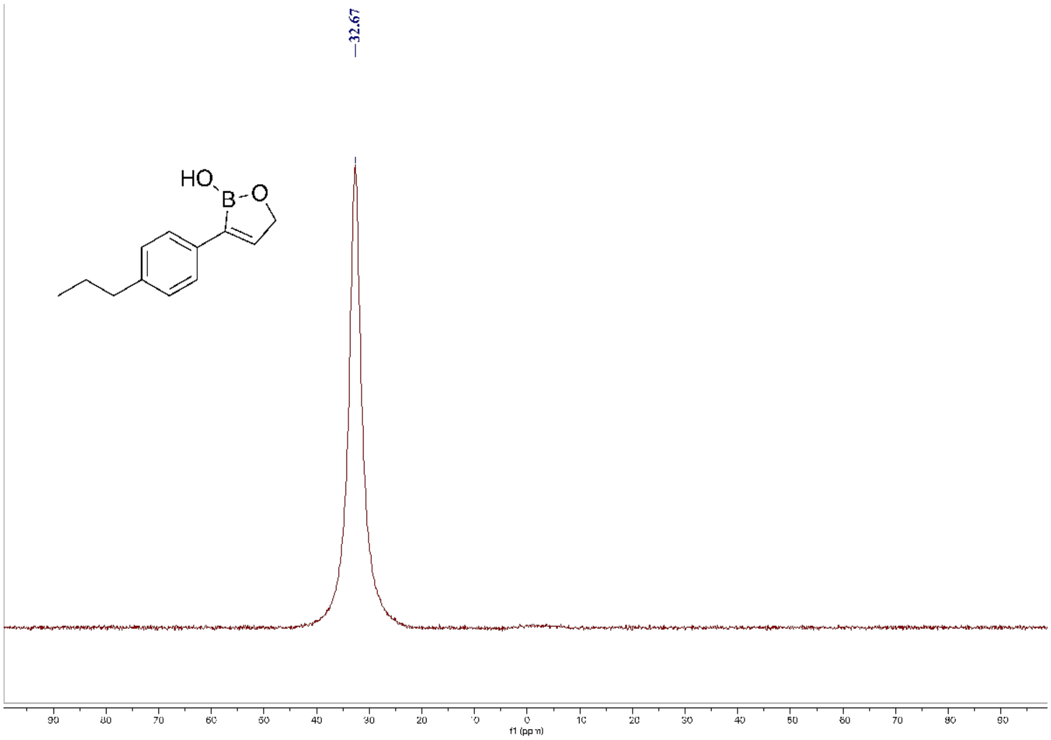

Figure S98. ^{11}B NMR of **6x** ($CDCl_3$, 128 MHz)

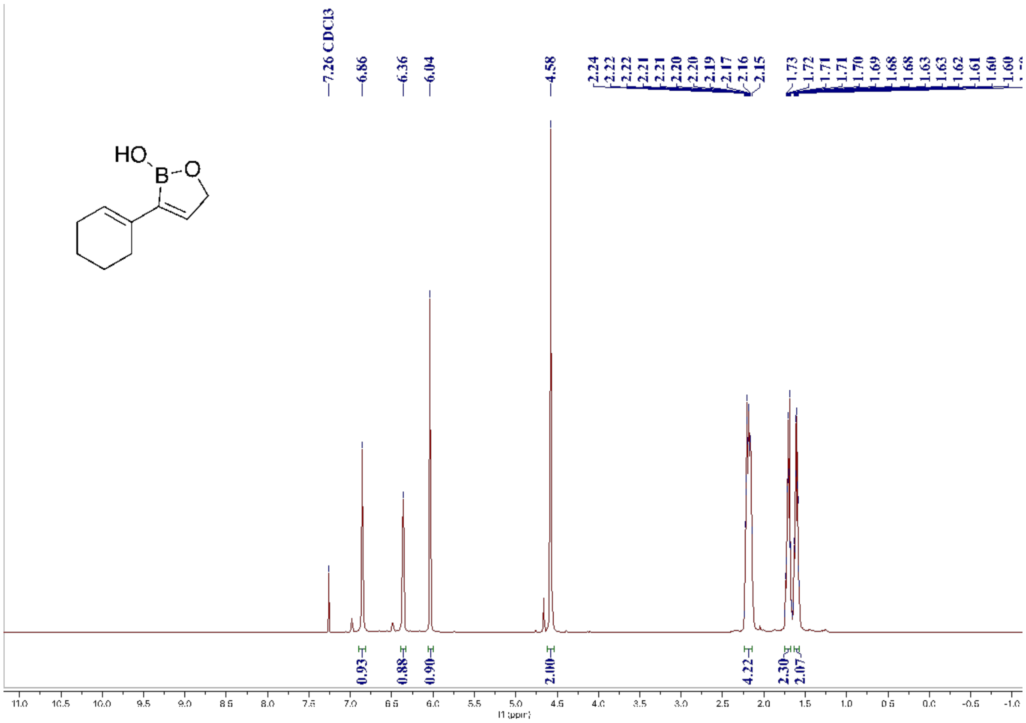

Figure S99. ^{1}H NMR of **6y** ($CDCl_3$, 400 MHz)

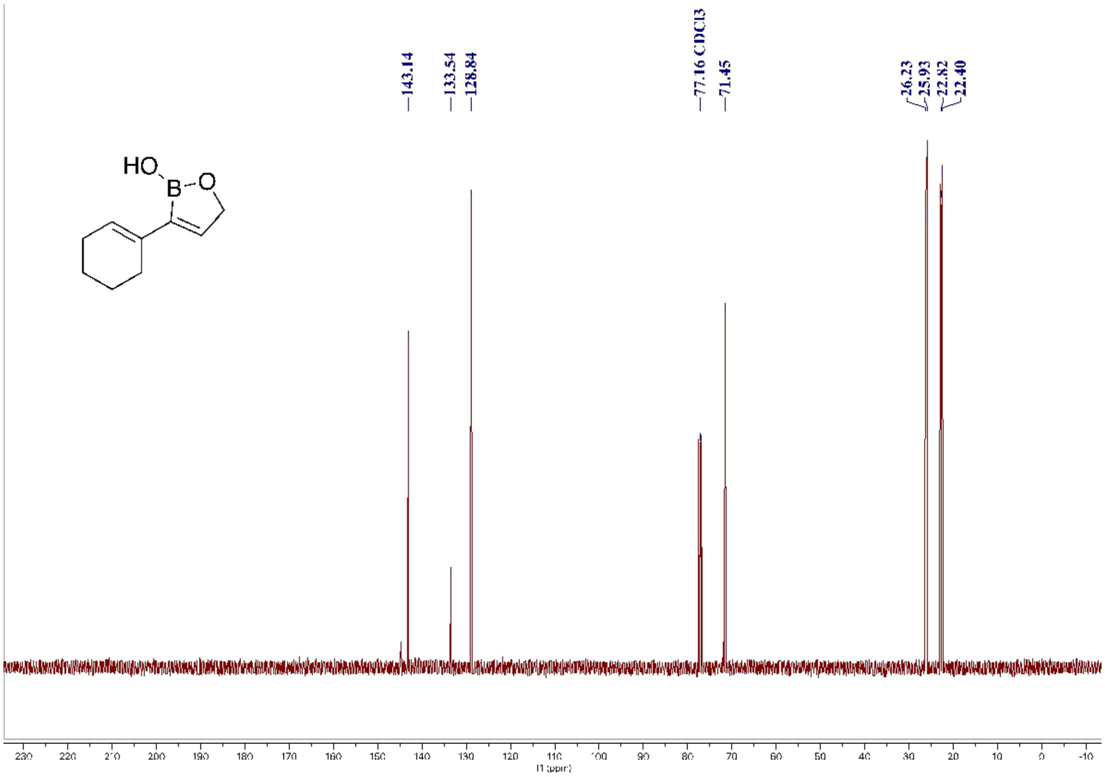

Figure S100. ^{13}C NMR of **6y** ($CDCl_3$, 100 MHz)

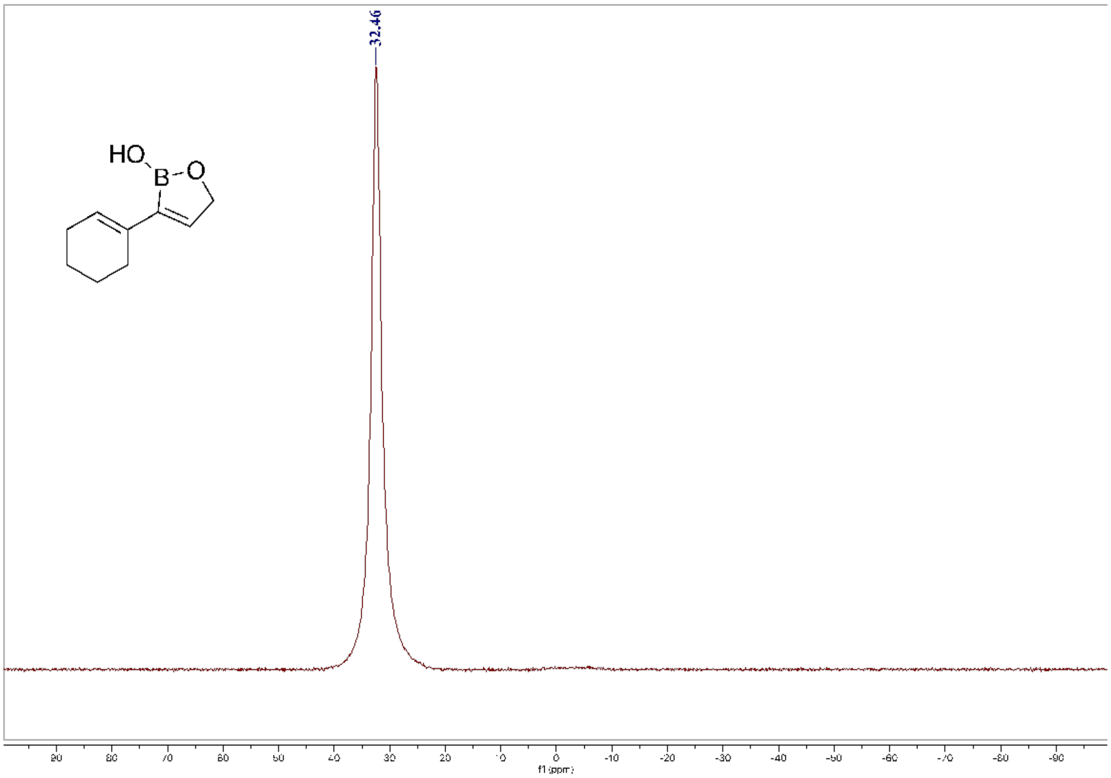

Figure S101. ^{11}B NMR of **6y** ($CDCl_3$, 128 MHz)

9) HPLC CHROMATOGRAMS OF THE OXABOROLES

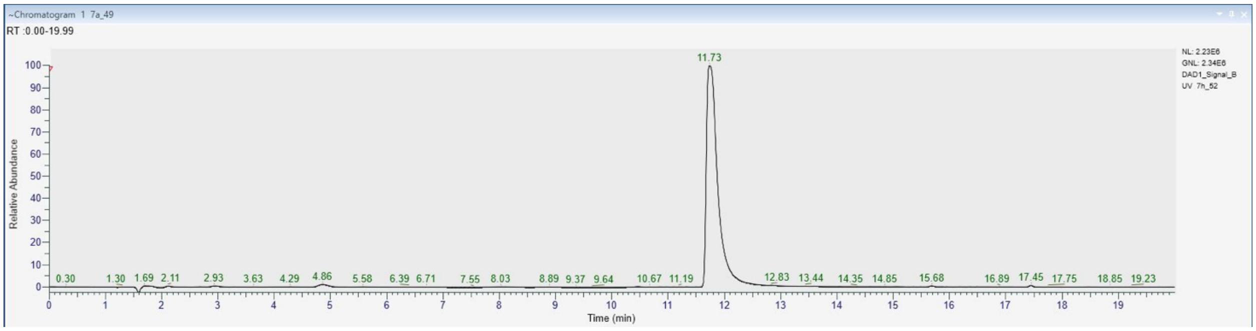

Figure S102. HPLC chromatogram for **6a**

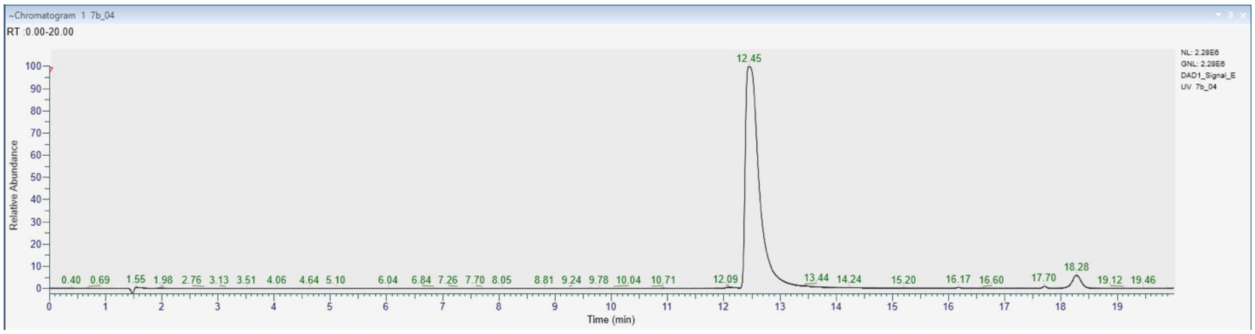

Figure S103. HPLC chromatogram for **6b**

Empower 3

Reporting Method v1

SAMPLE INFORMATION

Sample Name:	NB-9-19 1	Acquired By:	Chris Shrader
Sample Type:	Unknown	Sample Set Name:	NB_9_19
Vial:	1:A,8	Acq. Method Set:	Justin Peak Purity
Injection #:	1	Processing Method	Processing v1 PM
Injection Volume:	4.00 ul	Channel Name:	PDA Ch2 254nm@4.8nm
Run Time:	8.0 Minutes	Proc. Chnl. Descr.:	PDA Ch2 254nm@4.8nm
Date Acquired:	7/5/2022 2:48:20 PM EDT		
Date Processed:	7/6/2022 1:03:56 PM EDT		

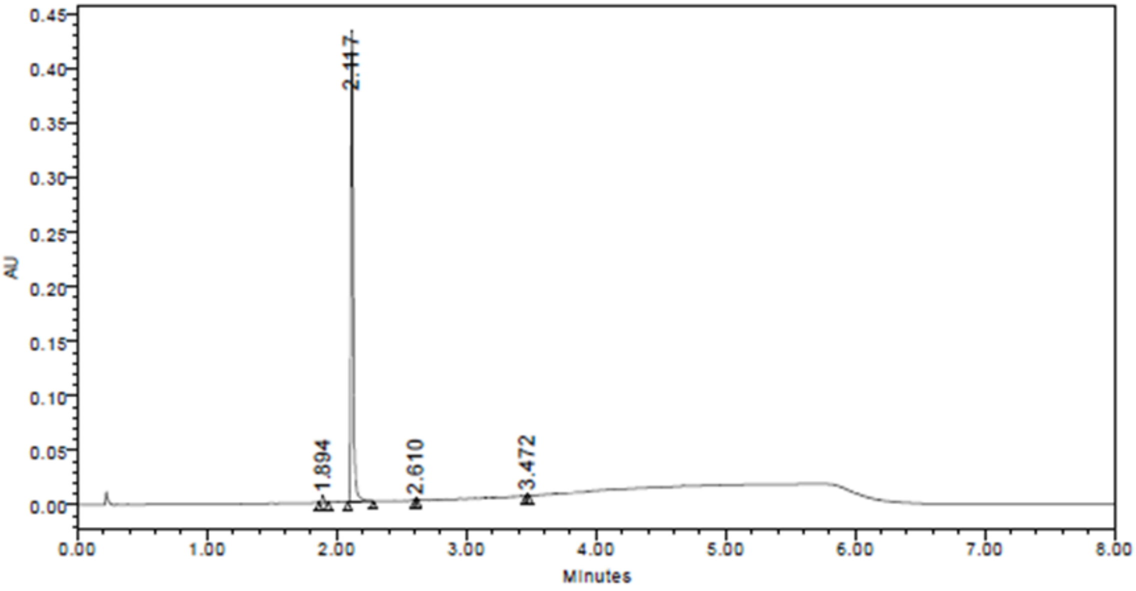

	RT	Area	% Area
1	1.894	7501	1.44
2	2.117	512265	98.09
3	2.610	1147	0.22
4	3.472	1334	0.26

Reported by User: chrisshrader (Chris Shrader)
Report Method: Reporting Method v1
Report Method ID 12720
Page: 1 of 1

Project Name: Sphingosine
Date Printed:
7/6/2022
1:04:47 PM US/Eastern

Figure S104. HPLC chromatogram for **6c**

Empower 3

Reporting Method v1

SAMPLE INFORMATION

Sample Name:	NB_9_31_2,NB_9_34_2 2	Acquired By:	ChrisShrader
Sample Type:	Unknown	Sample Set Name:	NB_9_342
Vial:	1:B,8	Acq. Method Set:	Justin Peak Purity
Injection #:	1	Processing Method	unknown
Injection Volume:	1.00 ul	Channel Name:	PDA Ch3 280nm@4.8nm
Run Time:	8.0 Minutes	Proc. Chnl. Descr.:	PDA Ch3 280nm@4.8nm
Date Acquired:	8/18/2022 5:45:52 PM EDT		
Date Processed:	8/18/2022 6:00:04 PM EDT		

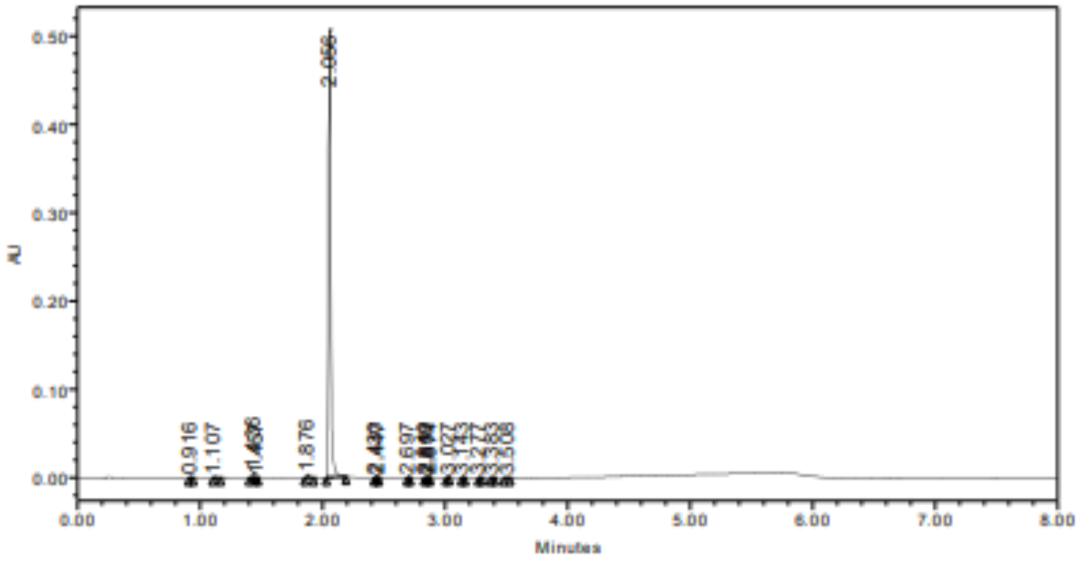

	RT	Area	% Area
1	0.916	52	0.01
2	1.107	340	0.06
3	1.436	5156	0.89
4	1.457	497	0.09
5	1.876	2416	0.42
6	2.056	566401	98.11
7	2.430	594	0.10
8	2.447	409	0.07
9	2.697	197	0.03

	RT	Area	% Area
10	2.840	125	0.02
11	2.852	154	0.03
12	2.871	30	0.01
13	3.027	279	0.05
14	3.143	212	0.04
15	3.277	28	0.00
16	3.383	216	0.04
17	3.508	230	0.04

Reported by User: chrisshrader (ChrisShrader)
Report Method: Reporting Method v1
Report Method ID 15325
Page: 1 of 1

Project Name: Sphingosine
Date Printed:
8/18/2022
6:00:25 PM US/Eastern

Figure S105. HPLC chromatogram for **6d**

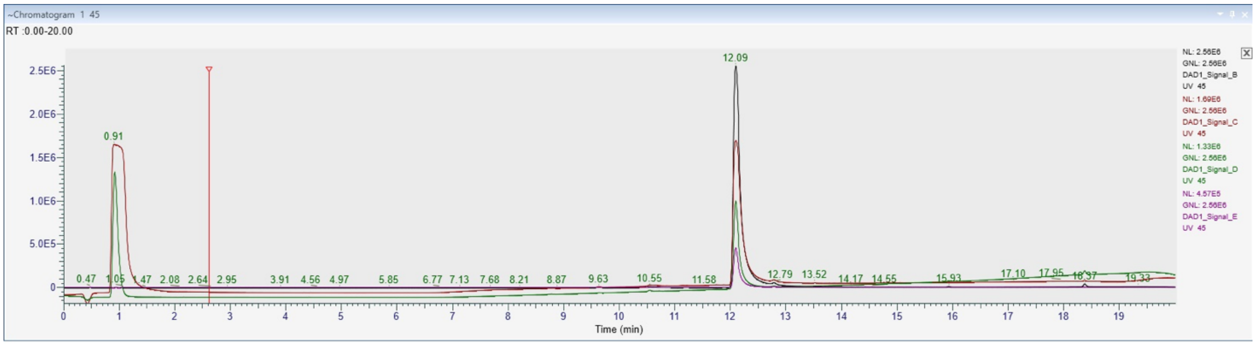

Figure S106. HPLC chromatogram for **6e**

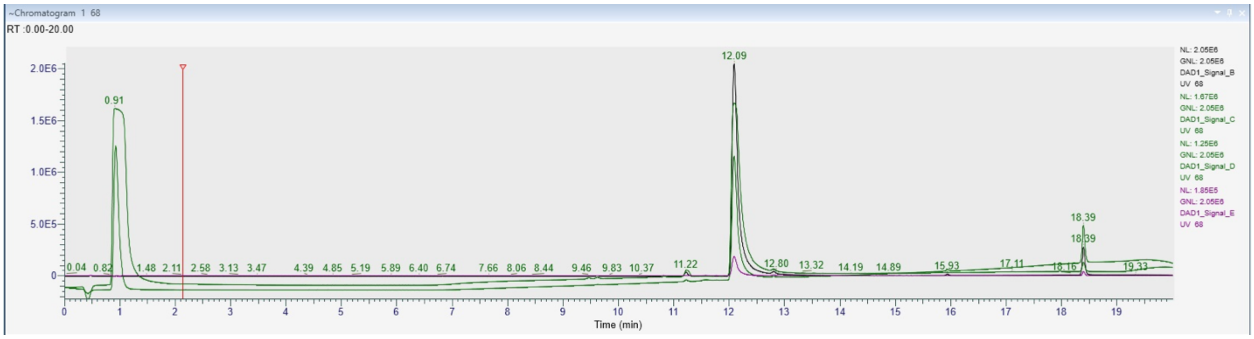

Figure S107. HPLC chromatogram for **6f**

Empower 3 SOFTWARE

Reporting Method v1

SAMPLE INFORMATION

Sample Name:	NB_9_18 1	Acquired By:	Chris Shrader
Sample Type:	Unknown	Sample Set Name:	NB_9_18
Vial:	1:B,6	Acq. Method Set:	Justin Peak Purity
Injection #:	1	Processing Method	unknown
Injection Volume:	1.00 ul	Channel Name:	PDA Ch2 254nm@4.8nm
Run Time:	8.0 Minutes	Proc. Chnl. Descr.:	PDA Ch2 254nm@4.8nm
Date Acquired:	8/18/2022 6:05:01 PM EDT		
Date Processed:	8/18/2022 6:14:46 PM EDT		

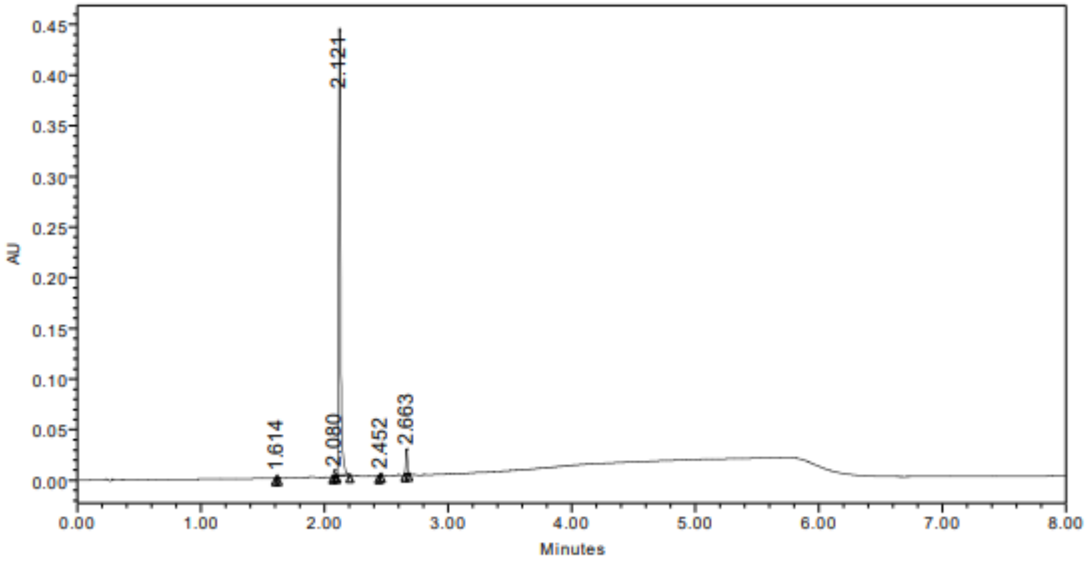

	RT	Area	% Area
1	1.614	1752	0.34
2	2.080	4221	0.81
3	2.121	490663	94.37
4	2.452	1393	0.27
5	2.663	21929	4.22

Reported by User: chrisshrader (Chris Shrader)
Report Method: Reporting Method v1
Report Method ID 15325
Page: 1 of 1

Project Name: Sphingosine
Date Printed:
8/18/2022
6:15:19 PM US/Eastern

Figure S108. HPLC chromatogram for **6g**

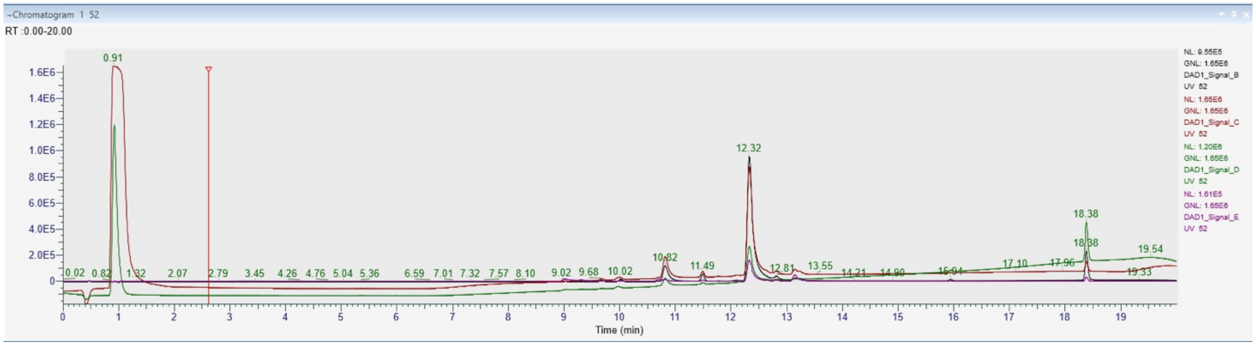

Figure S109. HPLC chromatogram for **6h**

Empower 3

Reporting Method v1

SAMPLE INFORMATION

Sample Name:	NB-9-20	Acquired By:	ChrisShrader
Sample Type:	Unknown	Sample Set Name:	NB_9
Vial:	1:B,6	Acq. Method Set:	Justin Peak Purity
Injection #:	1	Processing Method	Mary
Injection Volume:	1.00 ul	Channel Name:	PDA Ch2 254nm@4.8nm
Run Time:	18.0 Minutes	Proc. Chnl. Descr.:	PDA Ch2 254nm@4.8nm
Date Acquired:	7/6/2022 2:30:42 PM EDT		
Date Processed:	7/6/2022 3:03:32 PM EDT		

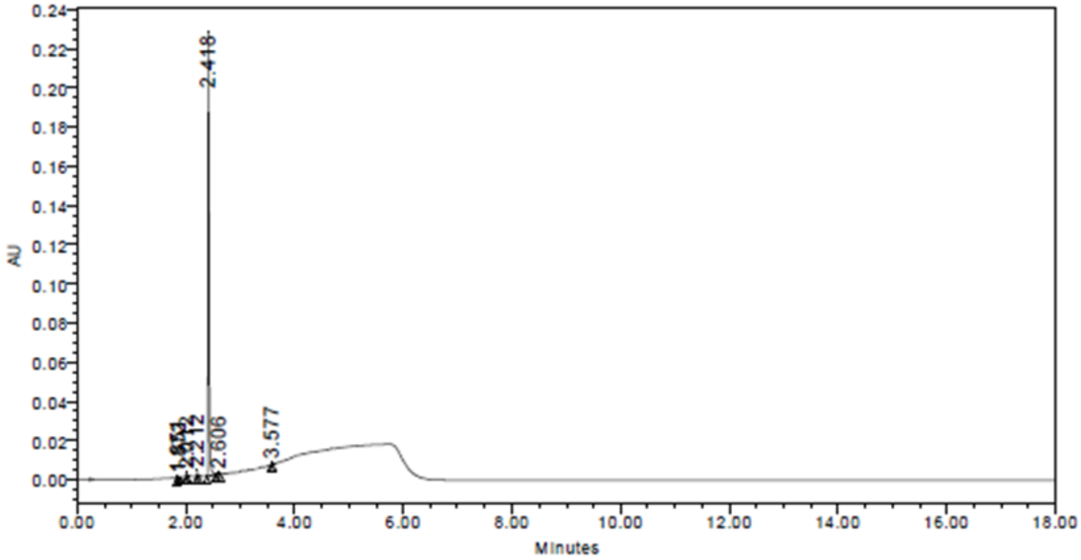

	RT	Area	% Area
1	1.851	794	0.30
2	1.872	54	0.02
3	2.012	1828	0.70
4	2.212	2718	1.04
5	2.418	253023	96.35
6	2.606	2996	1.14
7	3.577	1183	0.45

Reported by User: chrisshrader (ChrisShrader)
Report Method: Reporting Method v1
Report Method ID 12720
Page: 1 of 1

Project Name: Sphingosine
Date Printed:
7/6/2022
3:04:29 PM US/Eastern

Figure S110. HPLC chromatogram for **6i**

Empower 3

Reporting Method v1

SAMPLE INFORMATION

Sample Name:	NB_9_31_2,NB_9_34_2 1	Acquired By:	ChrisShrader
Sample Type:	Unknown	Sample Set Name:	NB_9_342
Vial:	1:B,7	Acq. Method Set:	Justin Peak Purity
Injection #:	1	Processing Method	Justin Peak Purity
Injection Volume:	1.00 ul	Channel Name:	PDA Ch2 254nm@4.8nm
Run Time:	8.0 Minutes	Proc. Chnl. Descr.:	PDA Ch2 254nm@4.8nm
Date Acquired:	8/18/2022 5:37:20 PM EDT		
Date Processed:	8/18/2022 5:51:08 PM EDT		

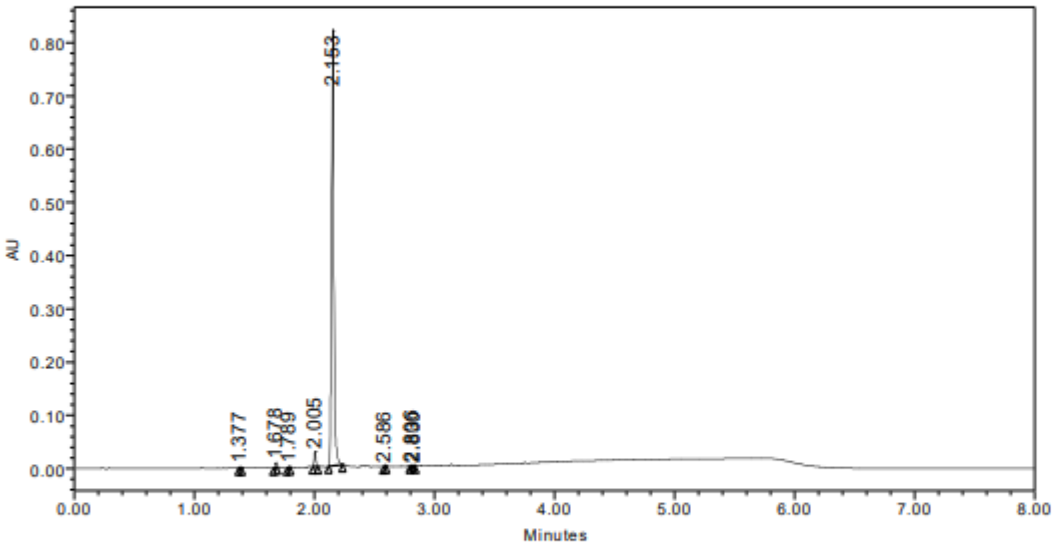

	RT	Area	% Area
1	1.377	1330	0.13
2	1.678	6589	0.62
3	1.789	1725	0.16
4	2.005	27347	2.58
5	2.153	1020272	96.10
6	2.586	1483	0.14
7	2.806	1710	0.16
8	2.830	1185	0.11

Reported by User: chrisshrader (ChrisShrader)
Report Method: Reporting Method v1
Report Method ID:15325
Page: 1 of 1

Project Name: Sphingosine
Date Printed:
8/18/2022
5:52:50 PM US/Eastern

Figure S111. HPLC chromatogram for **6j**

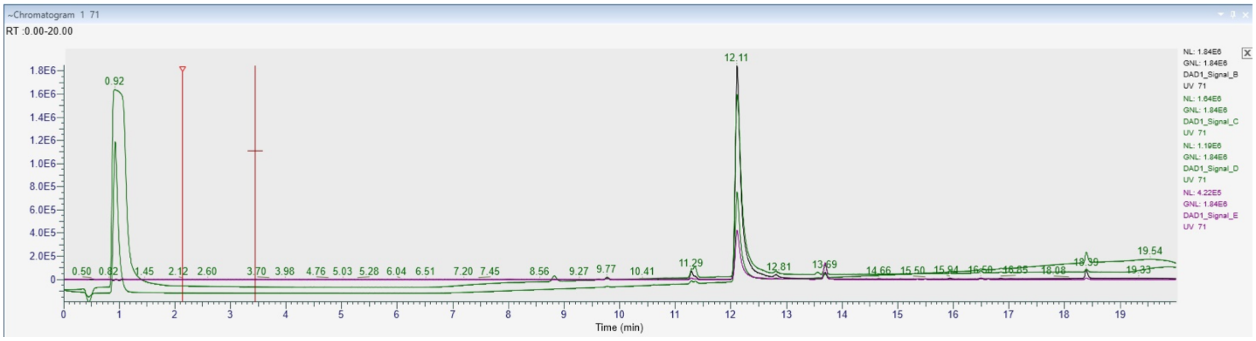

Figure S112. HPLC chromatogram for **6k**

Empower 3

Reporting Method v1

SAMPLE INFORMATION

Sample Name:	NB-9-Blank, NB-9-41 2	Acquired By:	Chris Shrader
Sample Type:	Unknown	Sample Set Name:	NB_9_41
Vial:	1:F,3	Acq. Method Set:	Justin Peak Purity
Injection #:	1	Processing Method	NB_9_31
Injection Volume:	2.00 ul	Channel Name:	PDA Ch2 254nm@4.8nm
Run Time:	8.0 Minutes	Proc. Chnl. Descr.:	PDA Ch2 254nm@4.8nm
Date Acquired:	10/10/2022 2:38:58 PM EDT		
Date Processed:	10/10/2022 2:51:33 PM EDT		

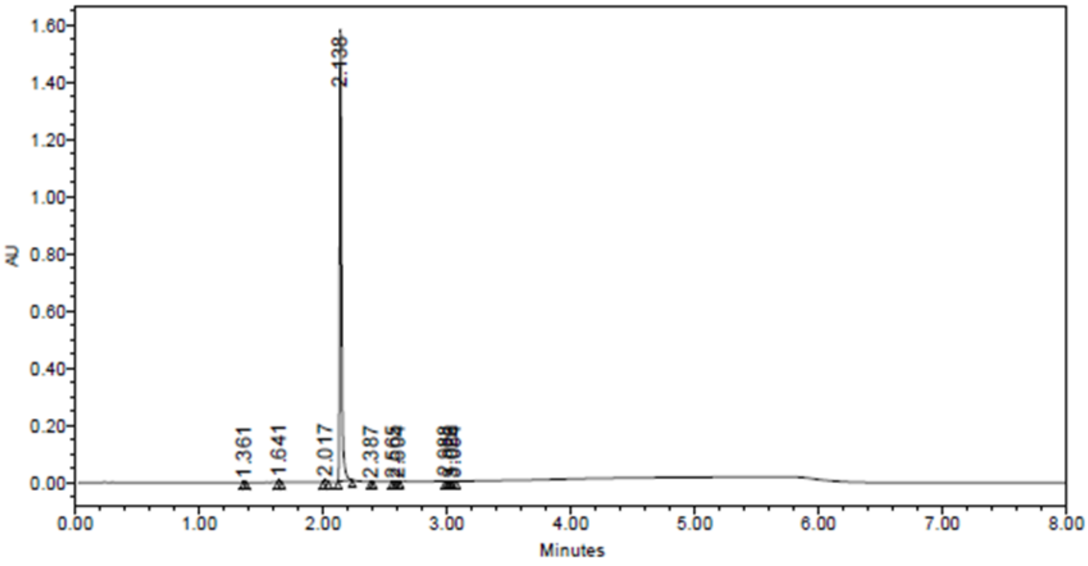

	RT	Area	% Area
1	1.361	1962	0.11
2	1.641	7732	0.44
3	2.017	9063	0.52
4	2.138	1721902	98.70
5	2.387	543	0.03
6	2.565	1372	0.08
7	2.604	757	0.04
8	2.988	634	0.04
9	3.026	252	0.01

	RT	Area	% Area
10	3.054	422	0.02

Reported by User: chrisshrader (Chris Shrader)
Report Method: Reporting Method v1
Report Method ID 15325
Page: 1 of 1

Project Name: Sphingosine
Date Printed:
10/10/2022
2:51:54 PM US/Eastern

Figure S113. HPLC chromatogram for **6l**

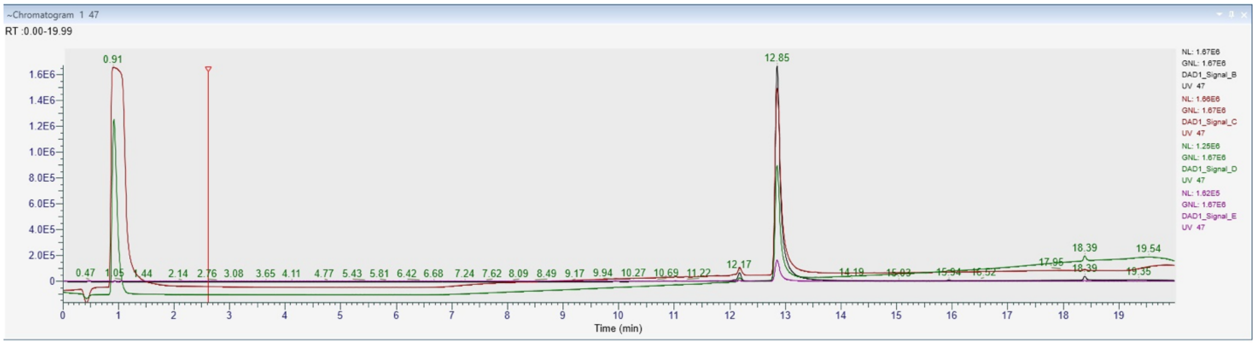

Figure S114. HPLC chromatogram for **6m**

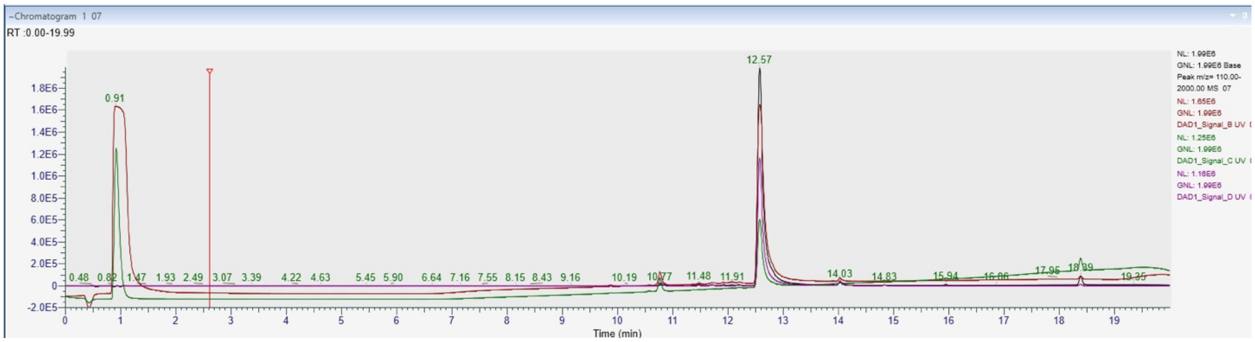

Figure S115. HPLC chromatogram for **6n**

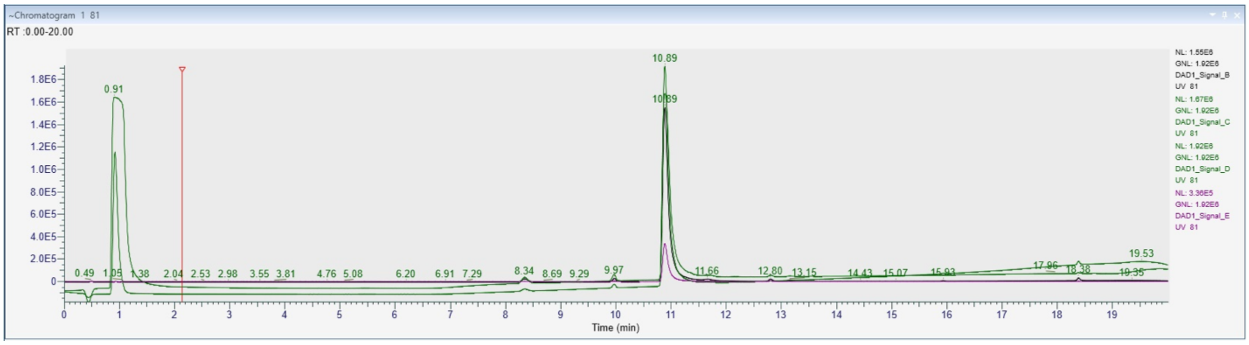

Figure S116. HPLC chromatogram for **6o**

Empower 3

Reporting Method v1

SAMPLE INFORMATION

Sample Name:	Blank, NB-9-47-2 2	Acquired By:	ChrisShrader
Sample Type:	Unknown	Sample Set Name:	NB_9_47_2
Vial:	1:C,8	Acq. Method Set:	Justin Peak Purity
Injection #:	1	Processing Method	NB_9_31
Injection Volume:	2.00 ul	Channel Name:	PDA Ch2 254nm@4.8nm
Run Time:	8.0 Minutes	Proc. Chnl. Descr.:	PDA Ch2 254nm@4.8nm
Date Acquired:	10/20/2022 5:03:42 PM EDT		
Date Processed:	10/20/2022 5:31:09 PM EDT		

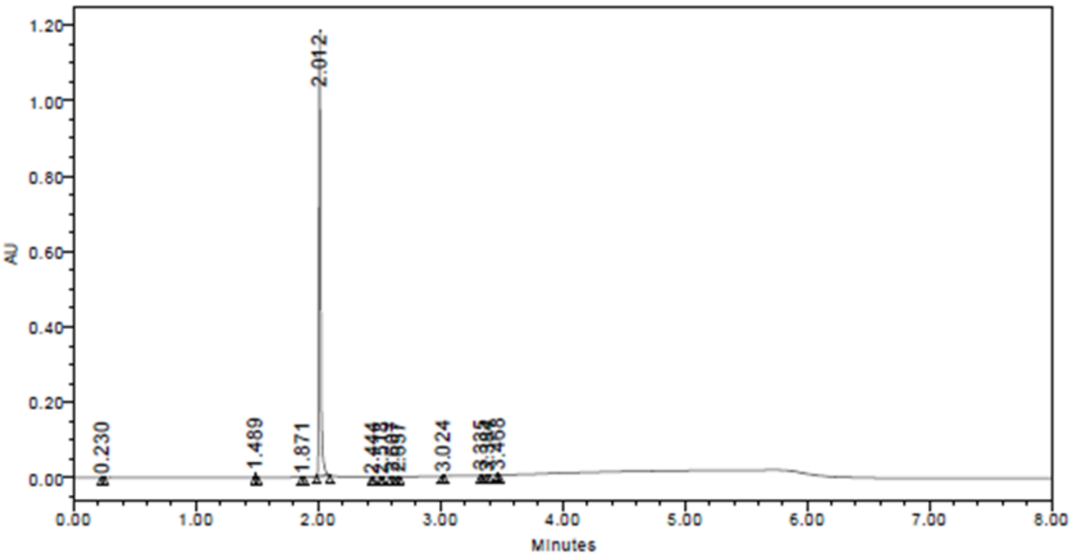

	RT	Area	% Area
1	0.230	1364	0.11
2	1.489	8377	0.68
3	1.871	71	0.01
4	2.012	1224673	98.89
5	2.444	21	0.00
6	2.518	50	0.00
7	2.607	522	0.04
8	2.657	419	0.03
9	3.024	567	0.05

	RT	Area	% Area
10	3.335	915	0.07
11	3.384	303	0.02
12	3.468	1173	0.09

Reported by User: chrisshrader (ChrisShrader)
Report Method: Reporting Method v1
Report Method ID 15325
Page: 1 of 1

Project Name: Sphingosine
Date Printed:
10/20/2022
5:31:28 PM US/Eastern

Figure S117. HPLC chromatogram for **6p**

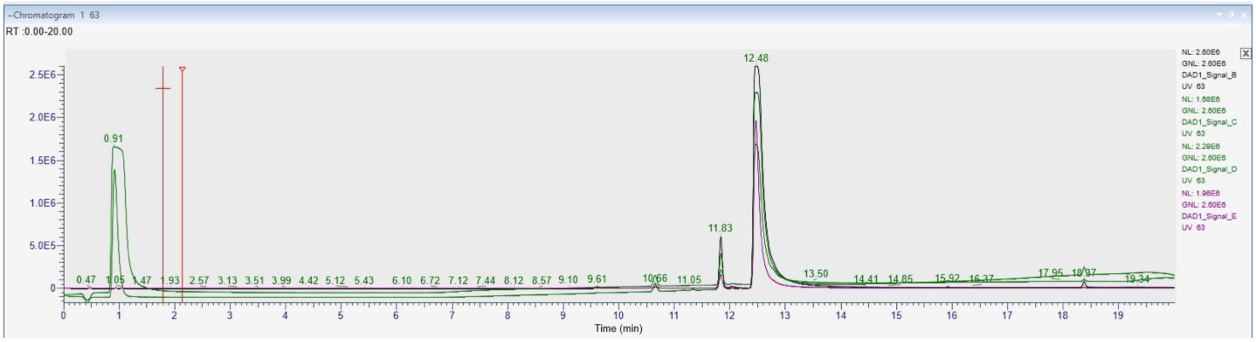

Figure S118. HPLC chromatogram for **6q**

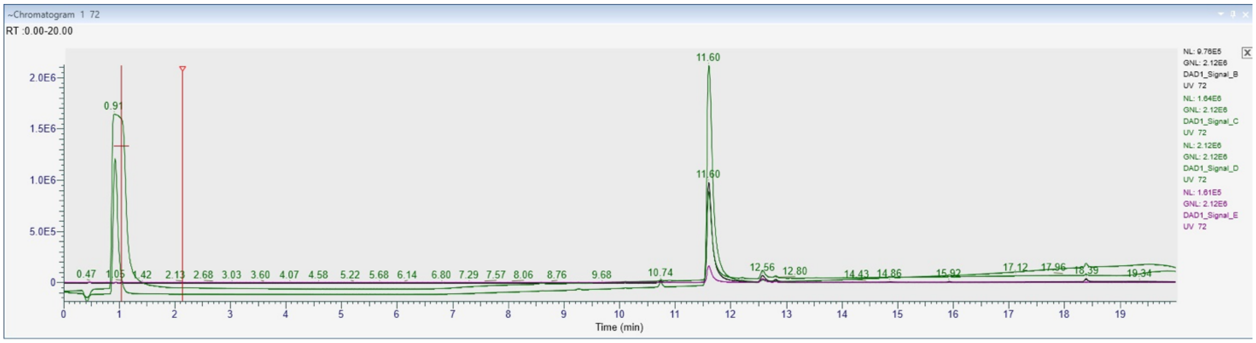

Figure S119. HPLC chromatogram for **6r**

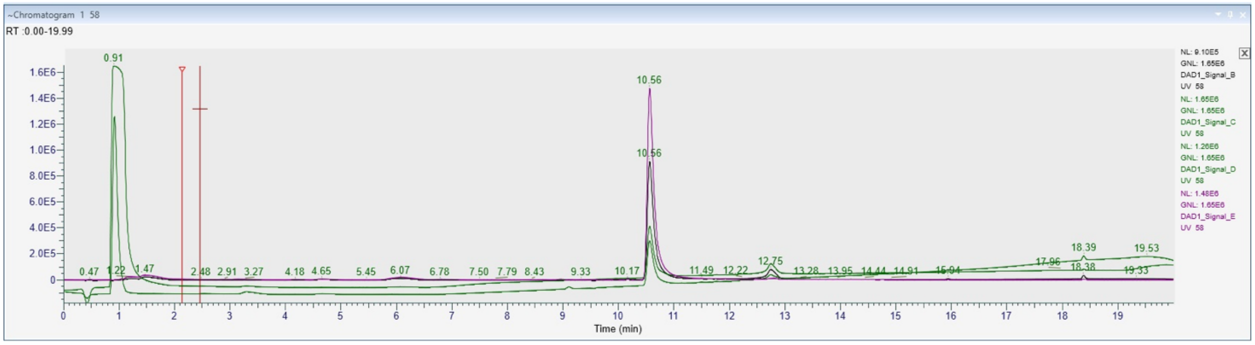

Figure S120. HPLC chromatogram for **6s**

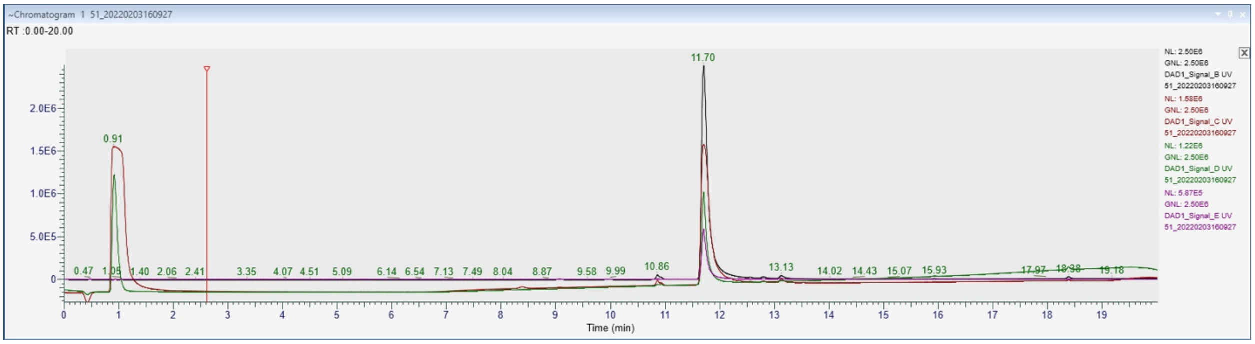

Figure S121. HPLC chromatogram for **6t**

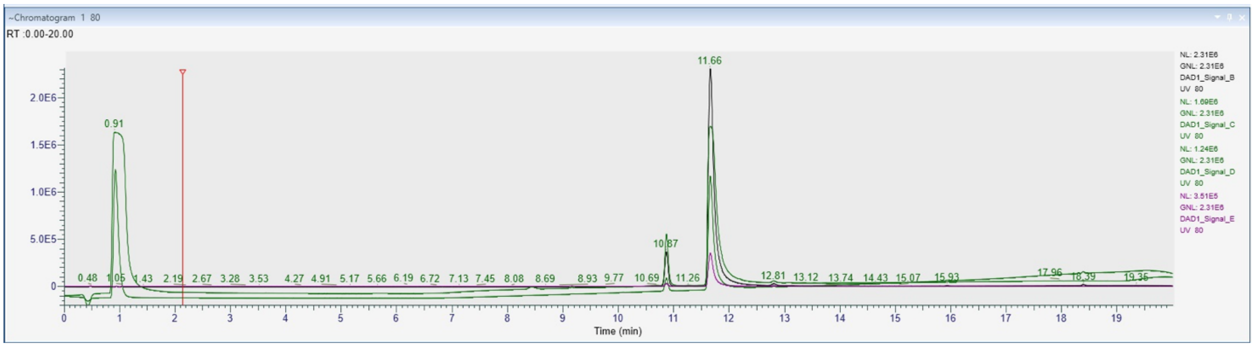

Figure S122. HPLC chromatogram for **6u**

Empower 3

Reporting Method v1

SAMPLE INFORMATION

Sample Name:	NB-9-Blank, NB-9-40 2	Acquired By:	Chris Shrader
Sample Type:	Unknown	Sample Set Name:	NB_9_40
Vial:	1:F,3	Acq. Method Set:	Justin Peak Purity
Injection #:	1	Processing Method	NB_9_31
Injection Volume:	2.00 ul	Channel Name:	PDA Ch2 254nm@4.8nm
Run Time:	8.0 Minutes	Proc. Chnl. Descr.:	PDA Ch2 254nm@4.8nm
Date Acquired:	10/10/2022 2:05:11 PM EDT		
Date Processed:	10/10/2022 2:24:31 PM EDT		

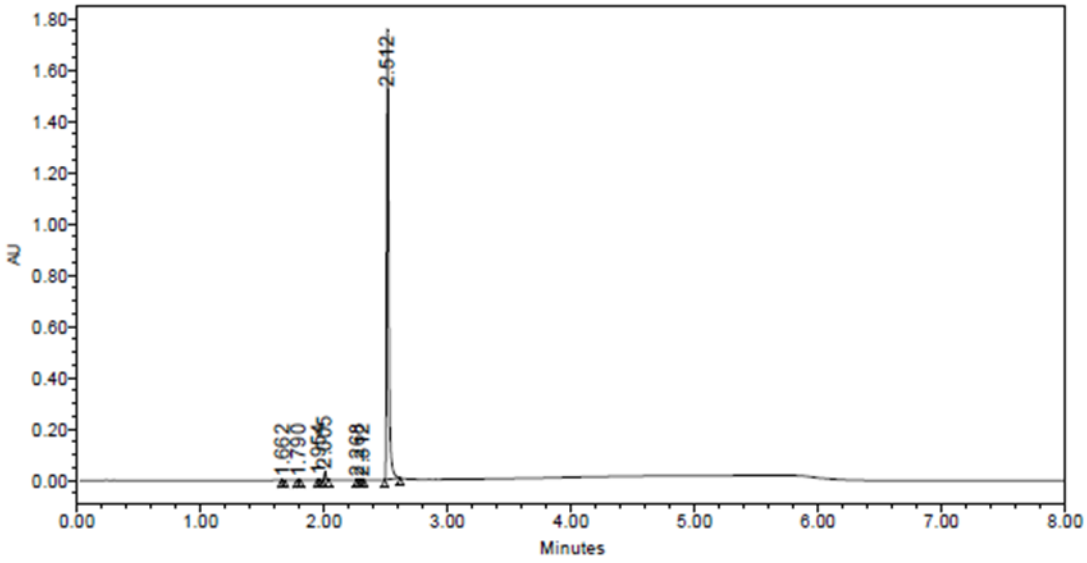

	RT	Area	% Area
1	1.662	327	0.02
2	1.790	827	0.04
3	1.954	3118	0.15
4	2.005	28340	1.39
5	2.268	865	0.04
6	2.312	1318	0.06
7	2.512	2005527	98.29

Reported by User: chrisshrader (Chris Shrader)
Report Method: Reporting Method v1
Report Method ID 15325
Page: 1 of 1

Project Name: Sphingosine
Date Printed:
10/10/2022
2:24:58 PM US/Eastern

Figure S123. HPLC chromatogram for **6v**

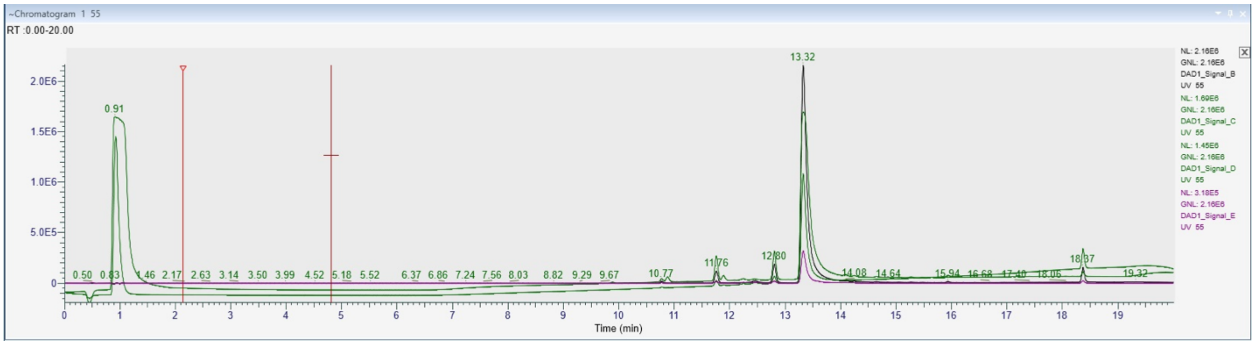

Figure S124. HPLC chromatogram for **6w**

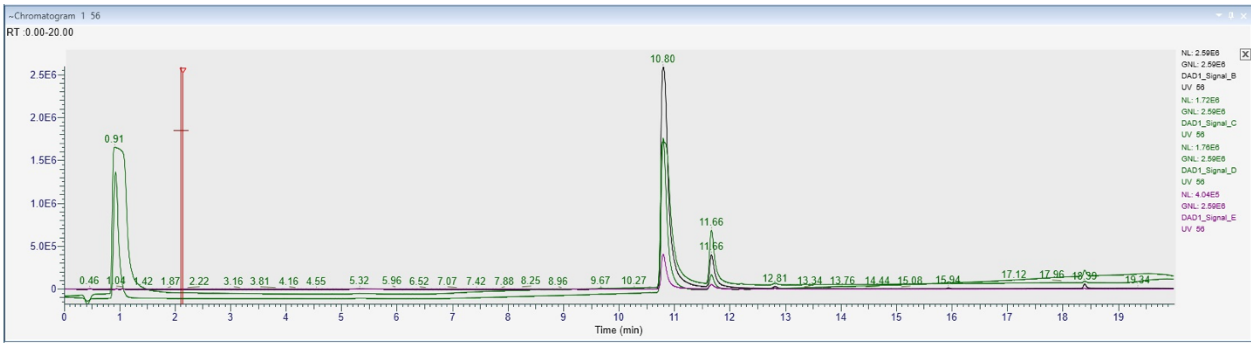

Figure S125. HPLC chromatogram for **6x**

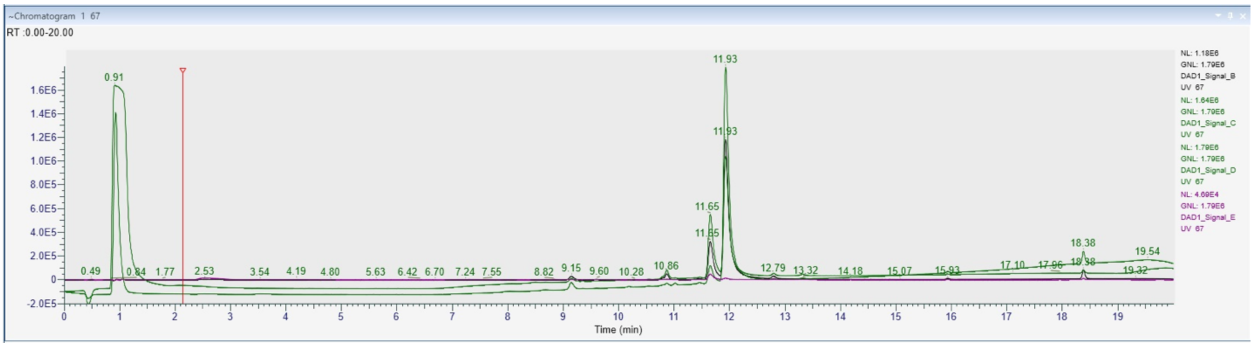

Figure S126. HPLC chromatogram for **6y**

10) PRELIMINARY ANTIMICROBIAL ASSAYS

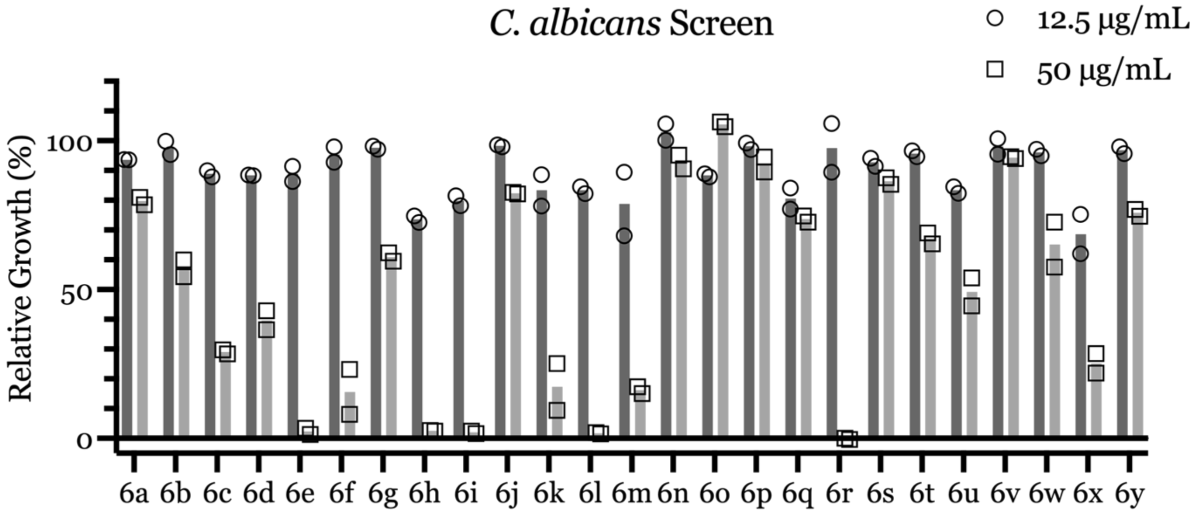

Figure S127. Preliminary screening results against *C. albicans*

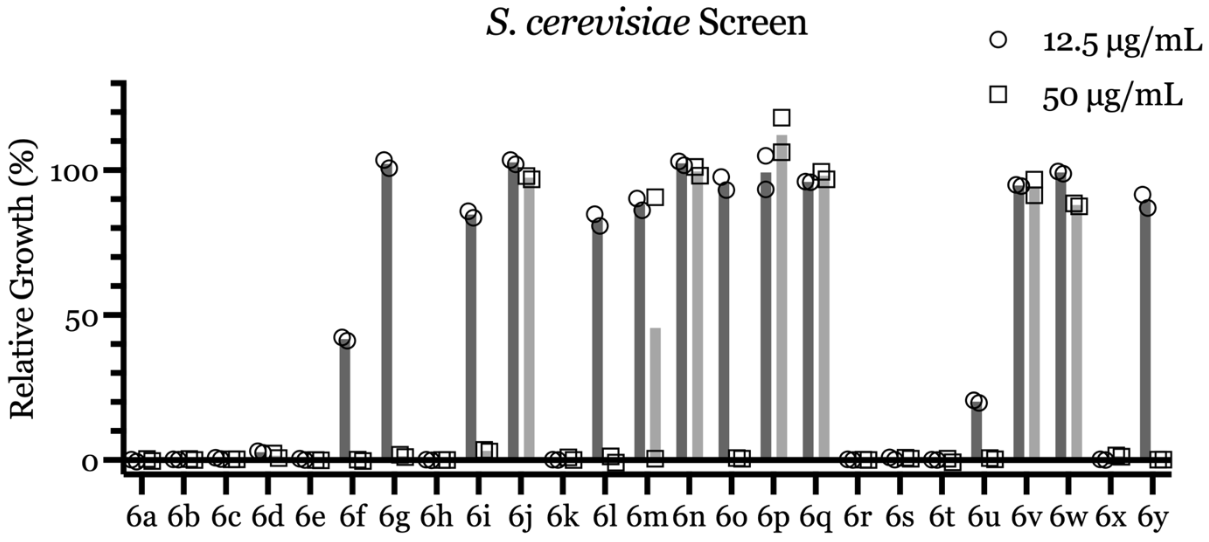

Figure S128. Preliminary screening results against *S. cerevisiae*

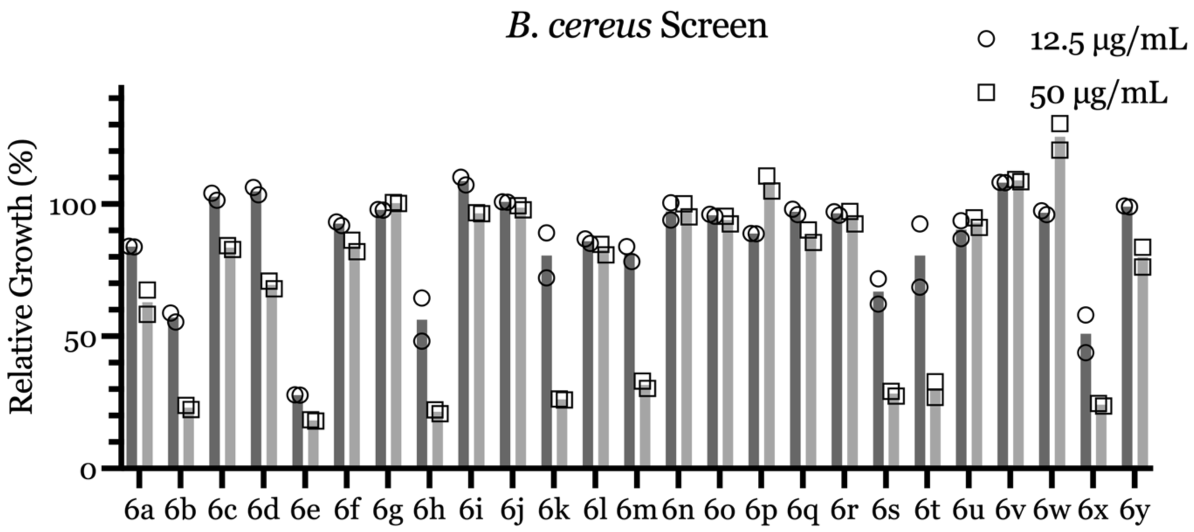

Figure S129. Preliminary screening results against *B. cereus*

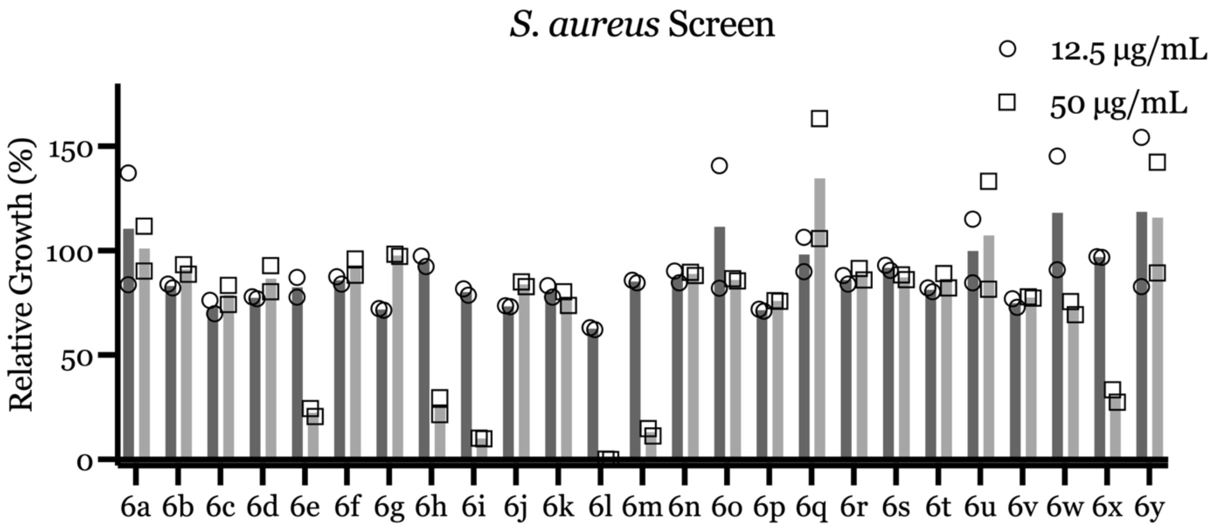

Figure S130. Preliminary screening results against *S. aureus*

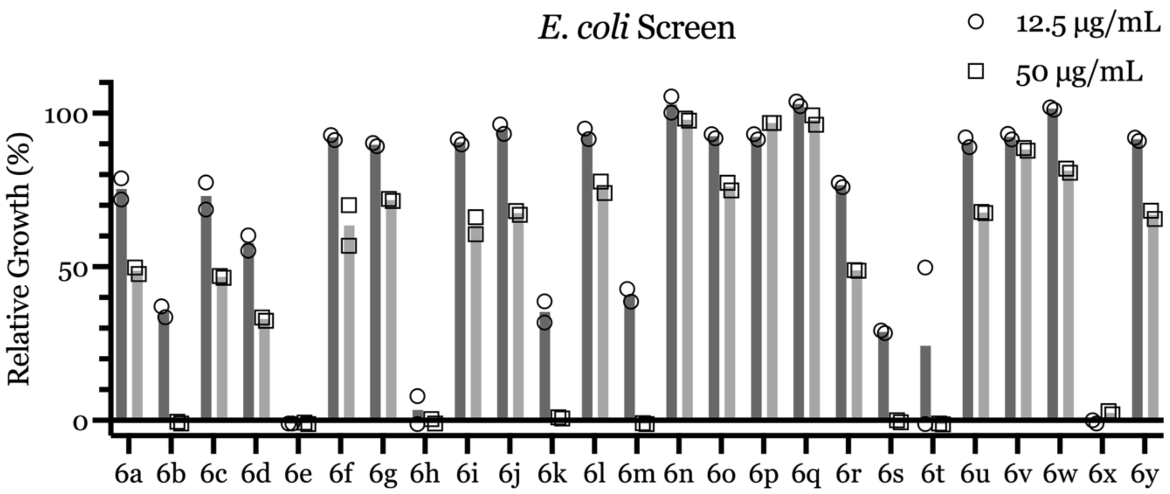

Figure S131. Preliminary screening results against *E. coli*

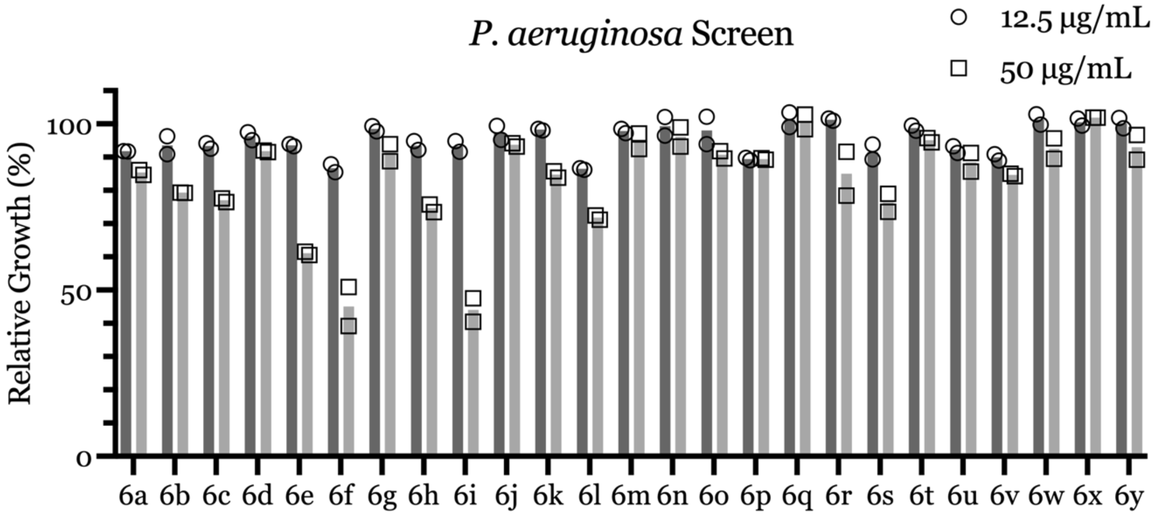

Figure S132. Preliminary screening results against *P. aeruginosa*

11) REPRESENTATIVE LIQUID DOSE-DEPENDENT ASSAYS

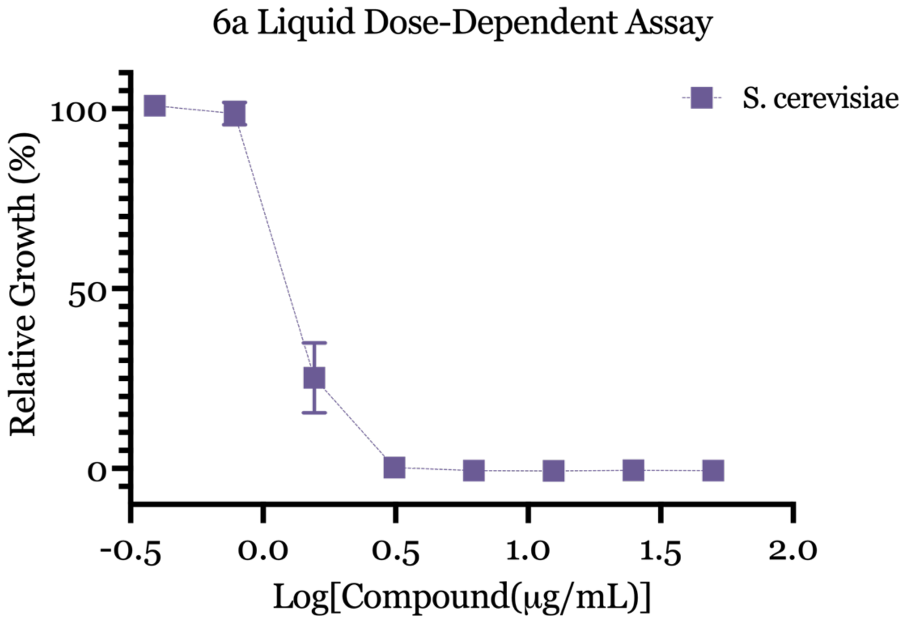

Figure S133. Representative biological replicate of the dose-dependent assay for **6a**

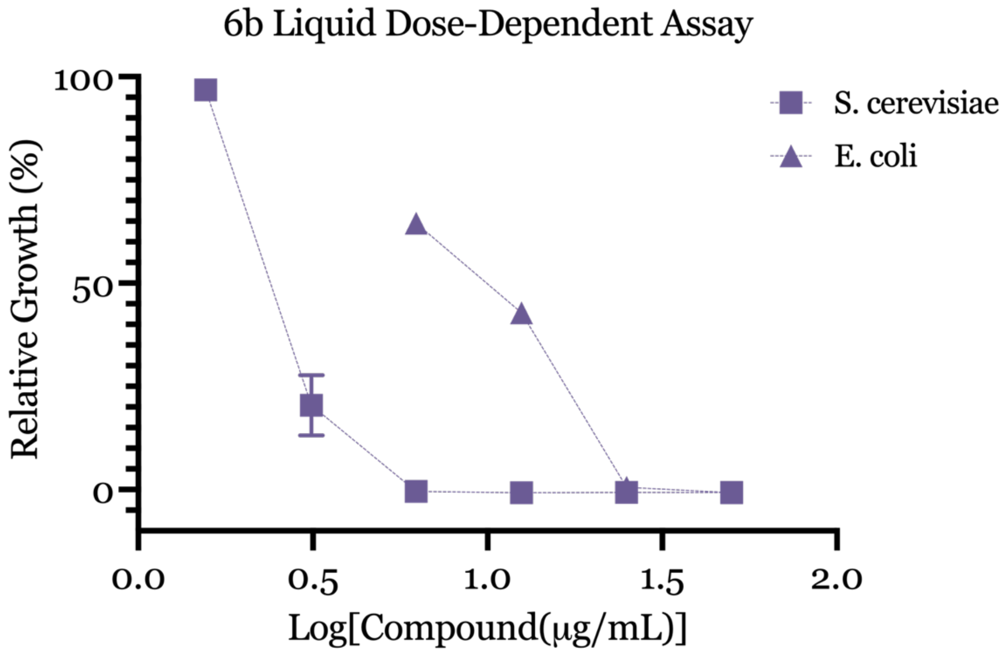

Figure S134. Representative biological replicate of the dose-dependent assay for **6b**

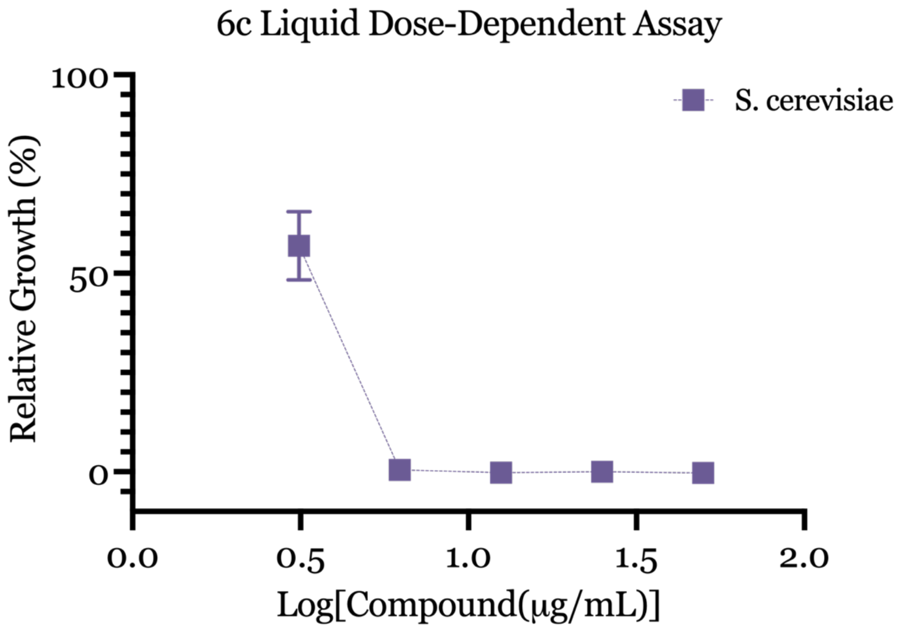

Figure S135. Representative biological replicate of the dose-dependent assay for **6c**

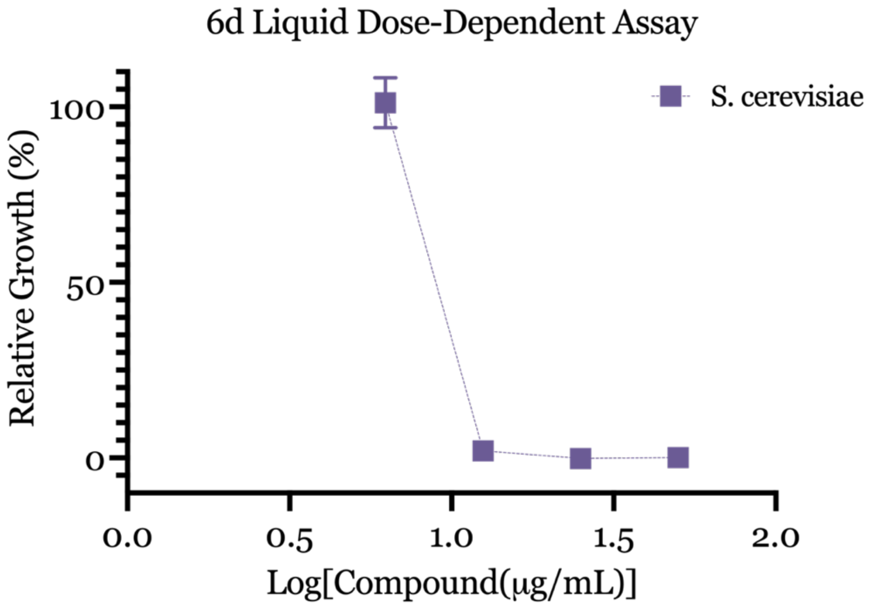

Figure S136. Representative biological replicate of the dose-dependent assay for **6b**d

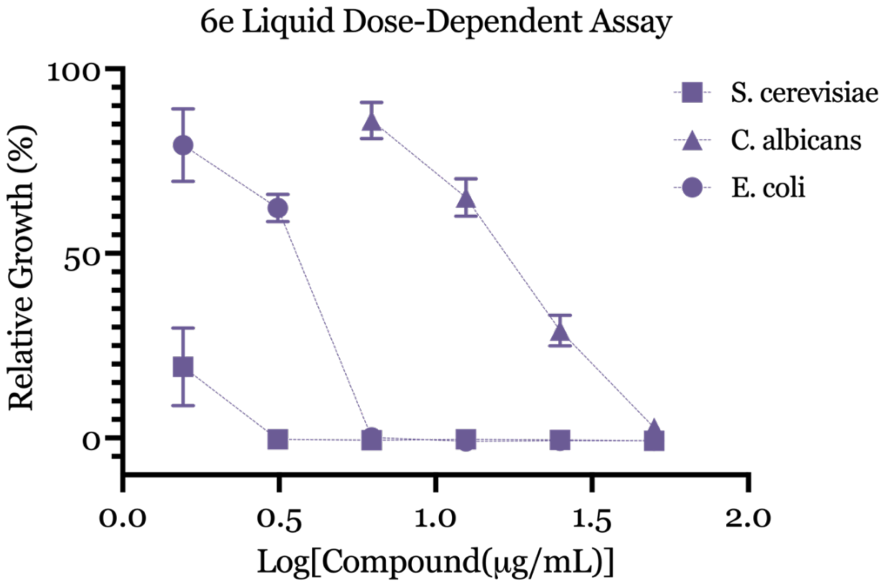

Figure S137. Representative biological replicate of the dose-dependent assay for **6e**

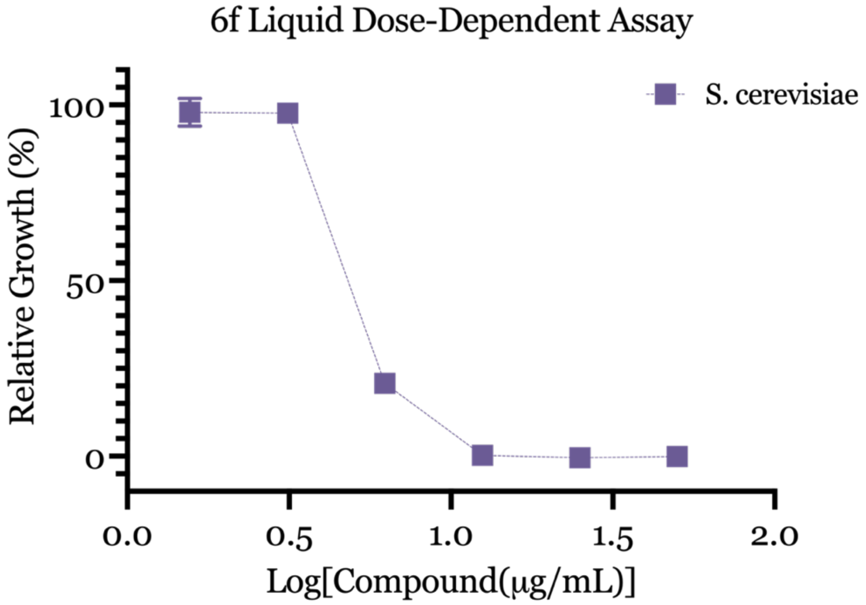

Figure S138. Representative biological replicate of the dose-dependent assay for **6f**

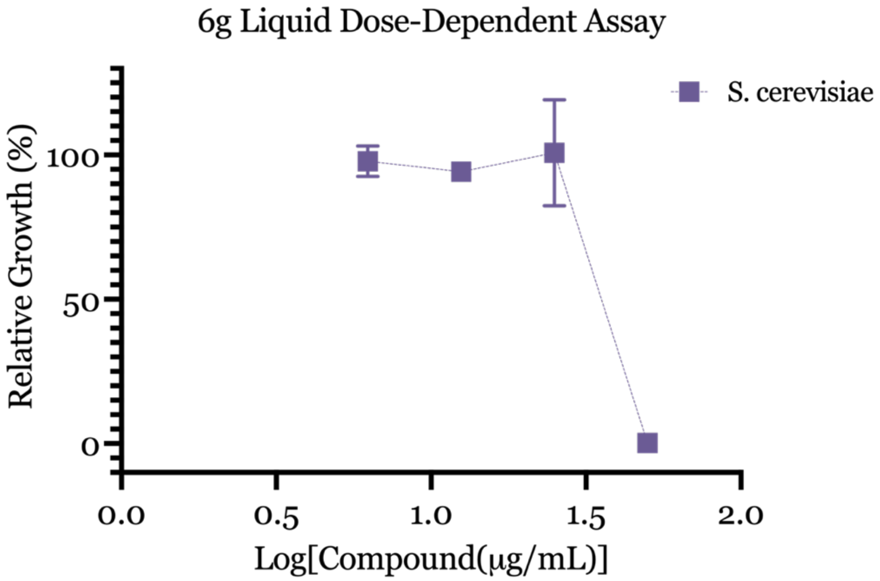

Figure S139. Representative biological replicate of the dose-dependent assay for **6**g

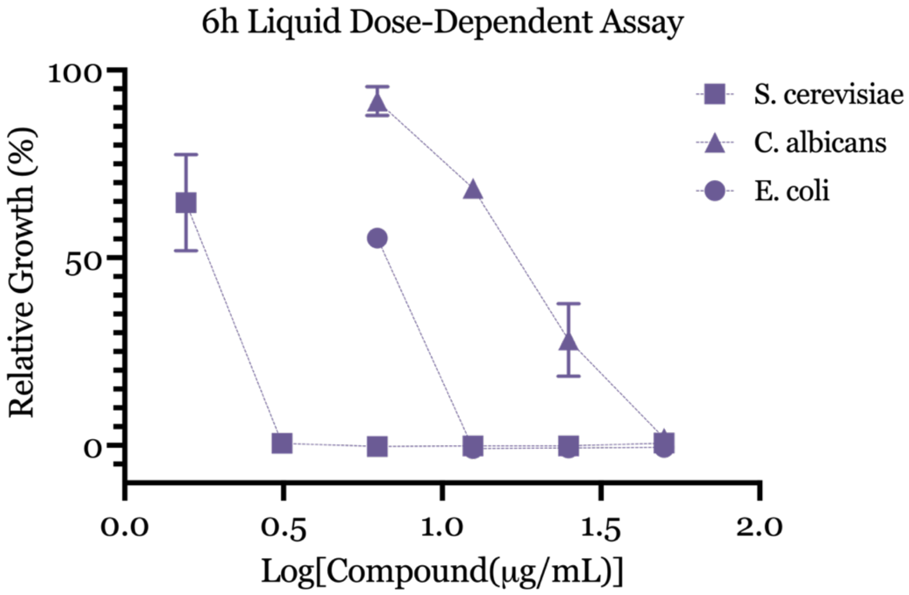

Figure S140. Representative biological replicate of the dose-dependent assay for **6h**

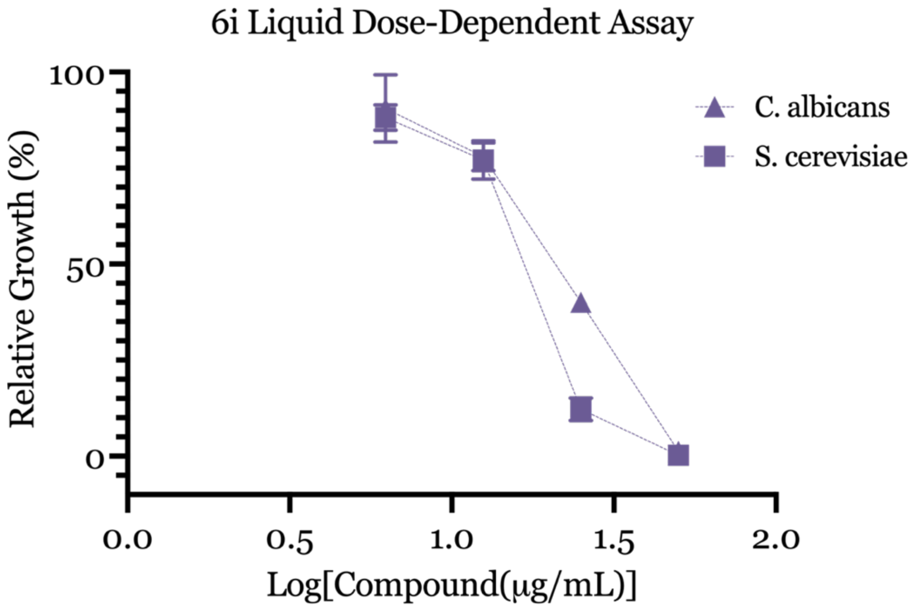

Figure S141. Representative biological replicate of the dose-dependent assay for **6i**

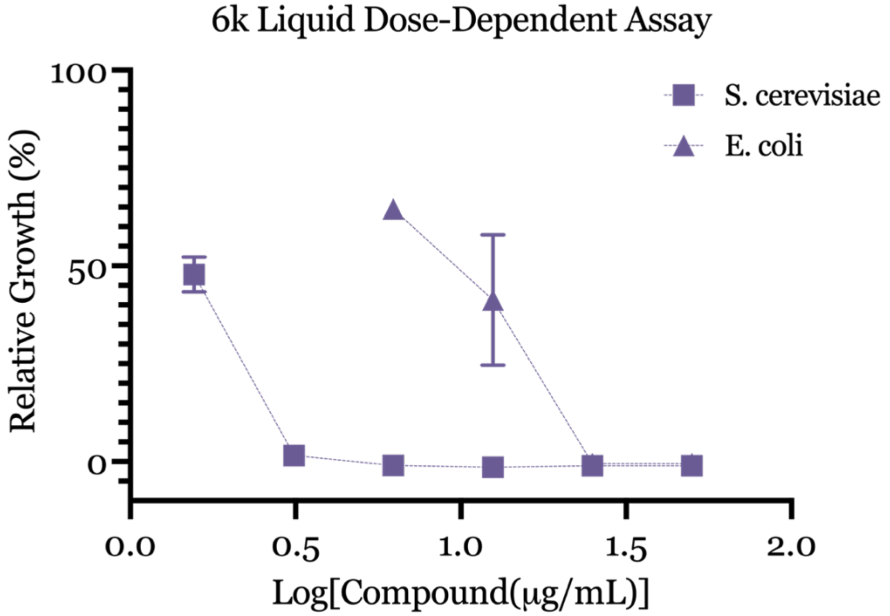

Figure S142. Representative biological replicate of the dose-dependent assay for **6k**

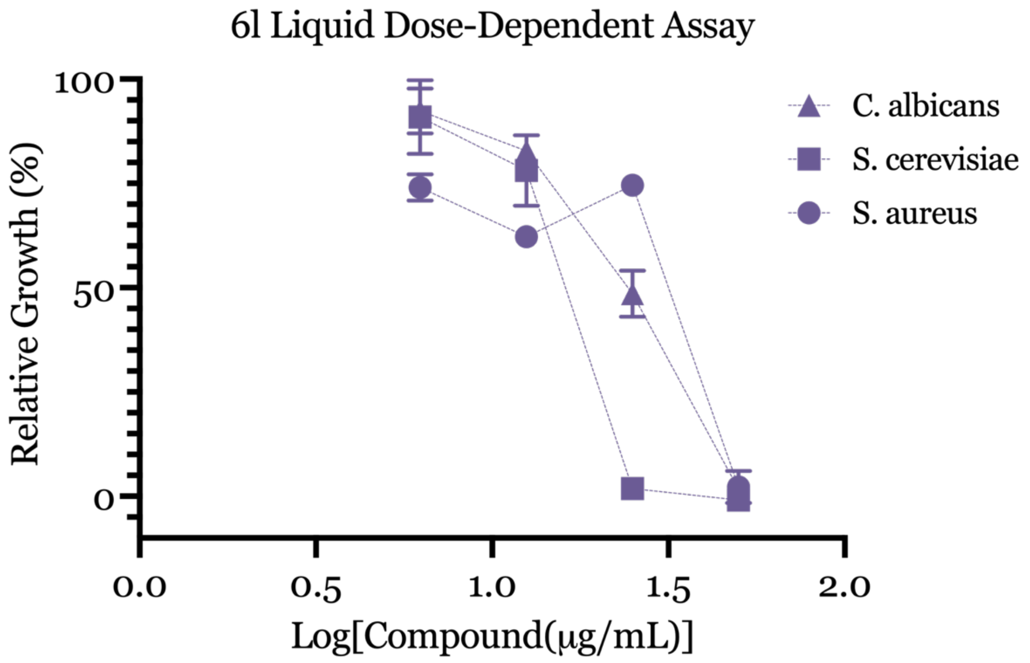

Figure S143. Representative biological replicate of the dose-dependent assay for **6l**

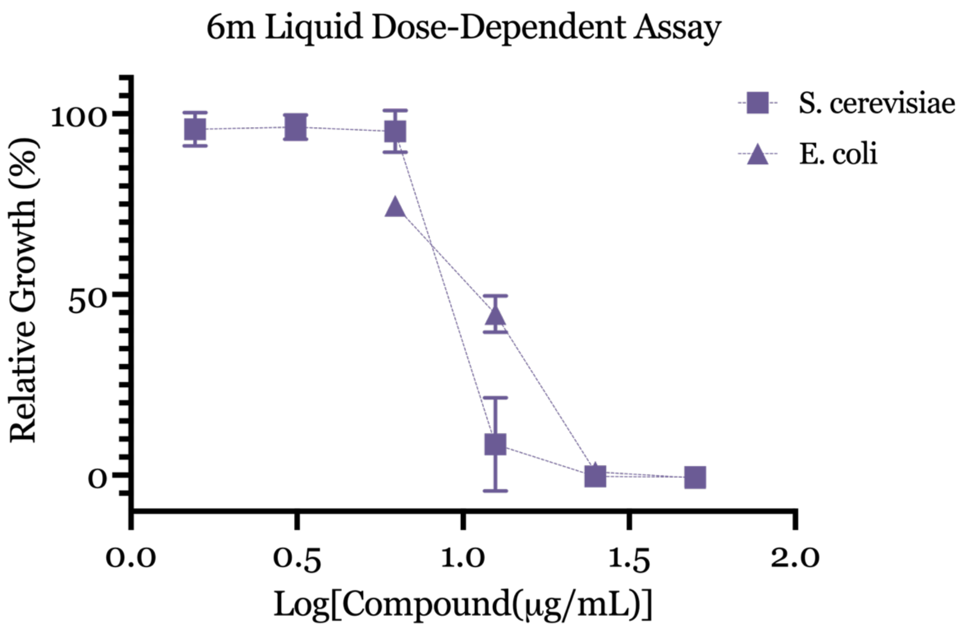

Figure S144. Representative biological replicate of the dose-dependent assay for **6m**

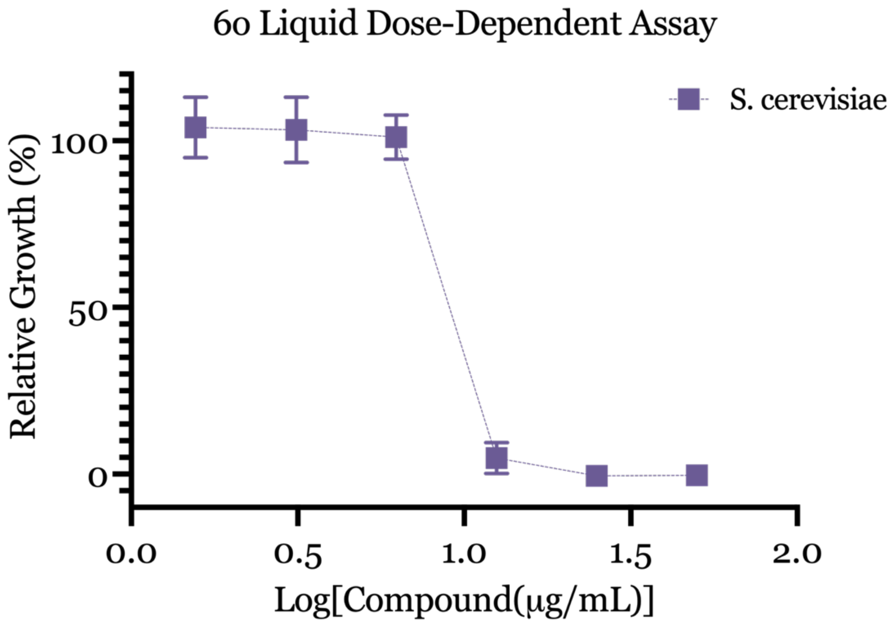

Figure S145. Representative biological replicate of the dose-dependent assay for **6o**

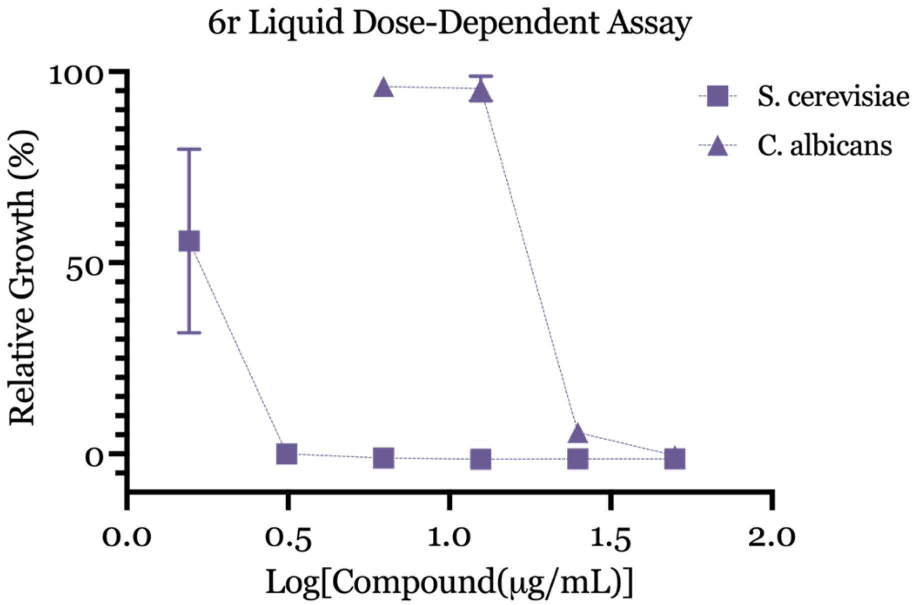

Figure S146. Representative biological replicate of the dose-dependent assay for **6r**

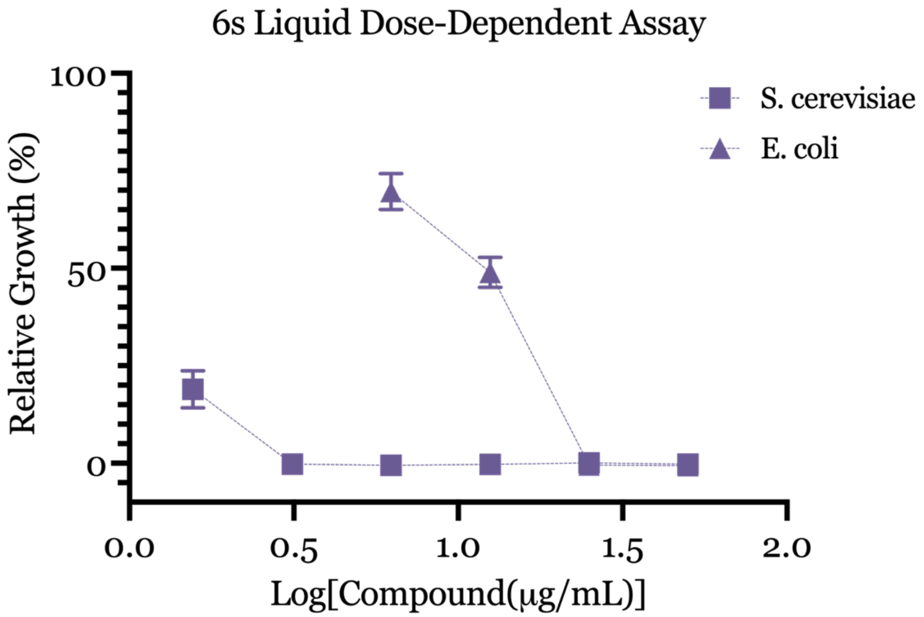

Figure S147. Representative biological replicate of the dose-dependent assay for **6s**

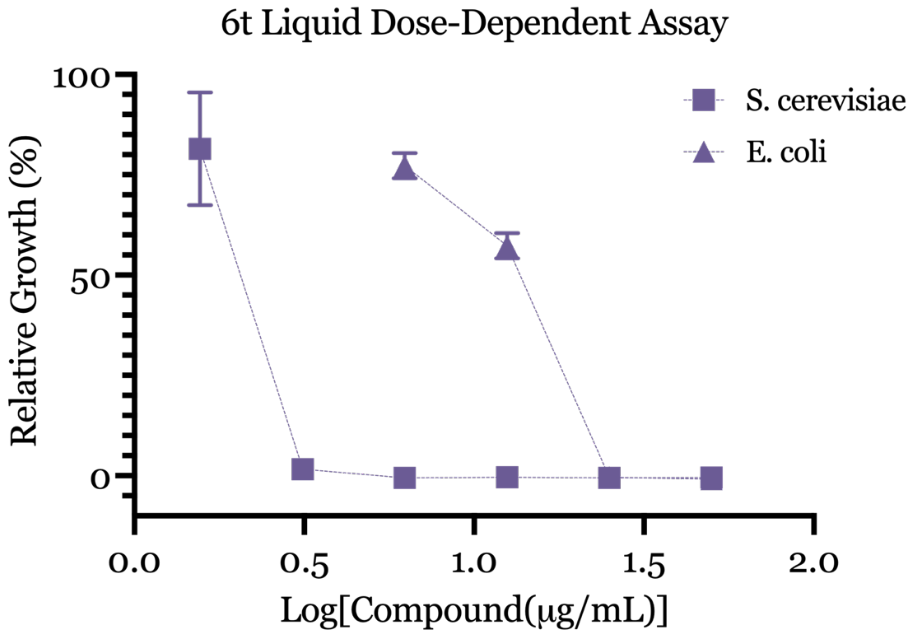

Figure S148. Representative biological replicate of the dose-dependent assay for **6t**

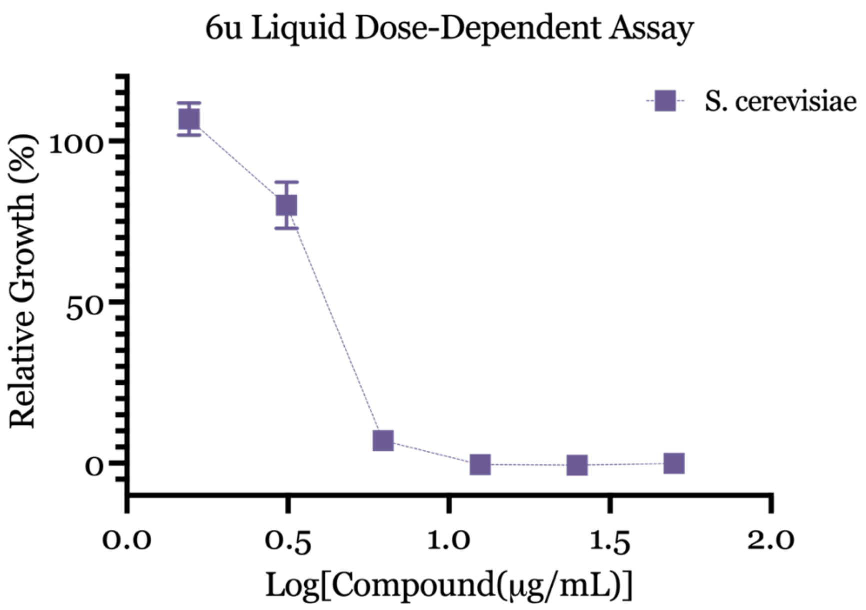

Figure S149. Representative biological replicate of the dose-dependent assay for **6u**

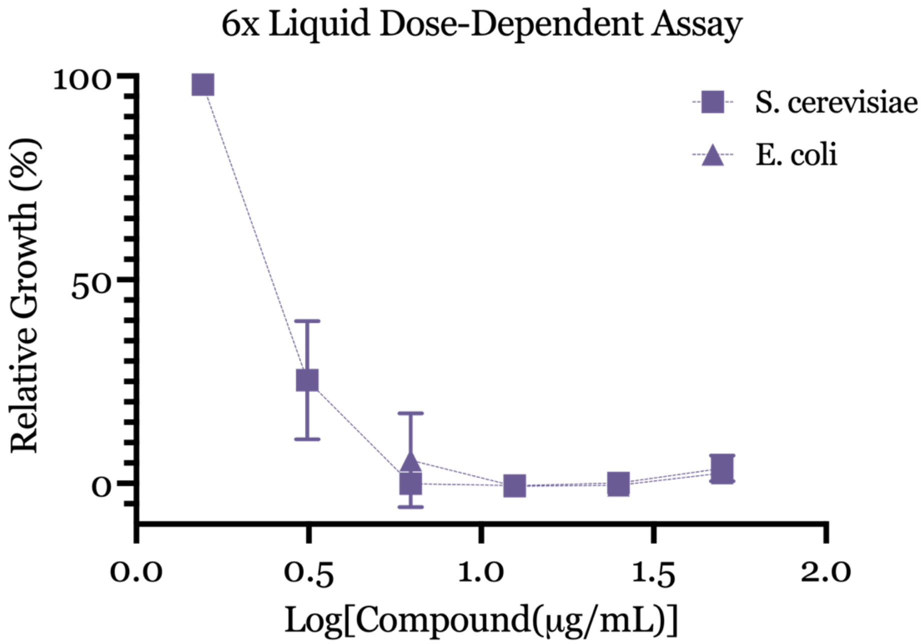

Figure S150. Representative biological replicate of the dose-dependent assay for **6x**

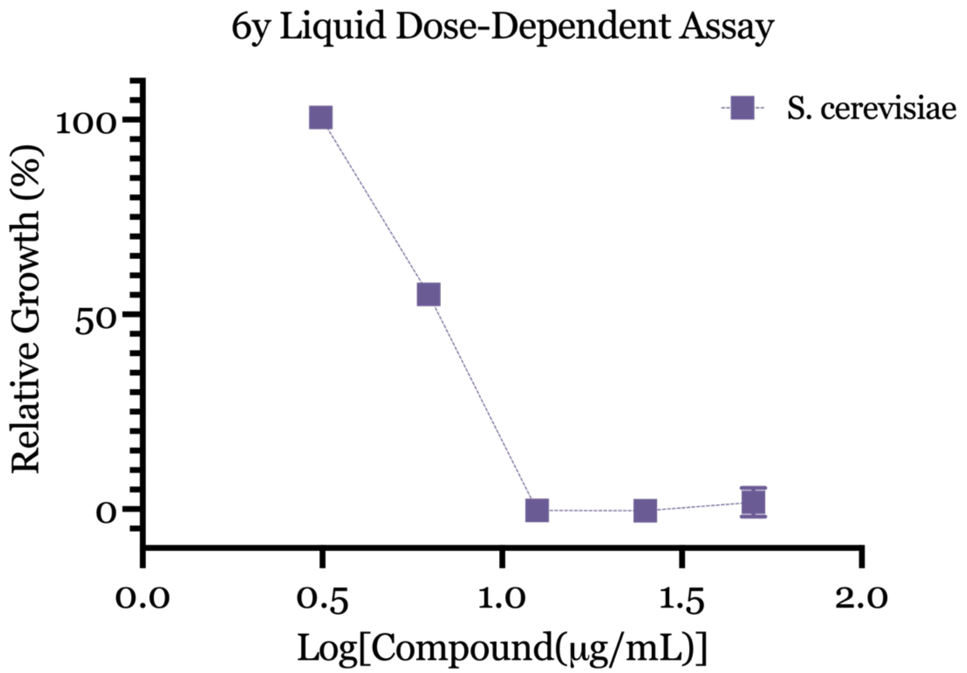

Figure S151. Representative biological replicate of the dose-dependent assay for **6y**

12) SOLID MIC ASSAYS

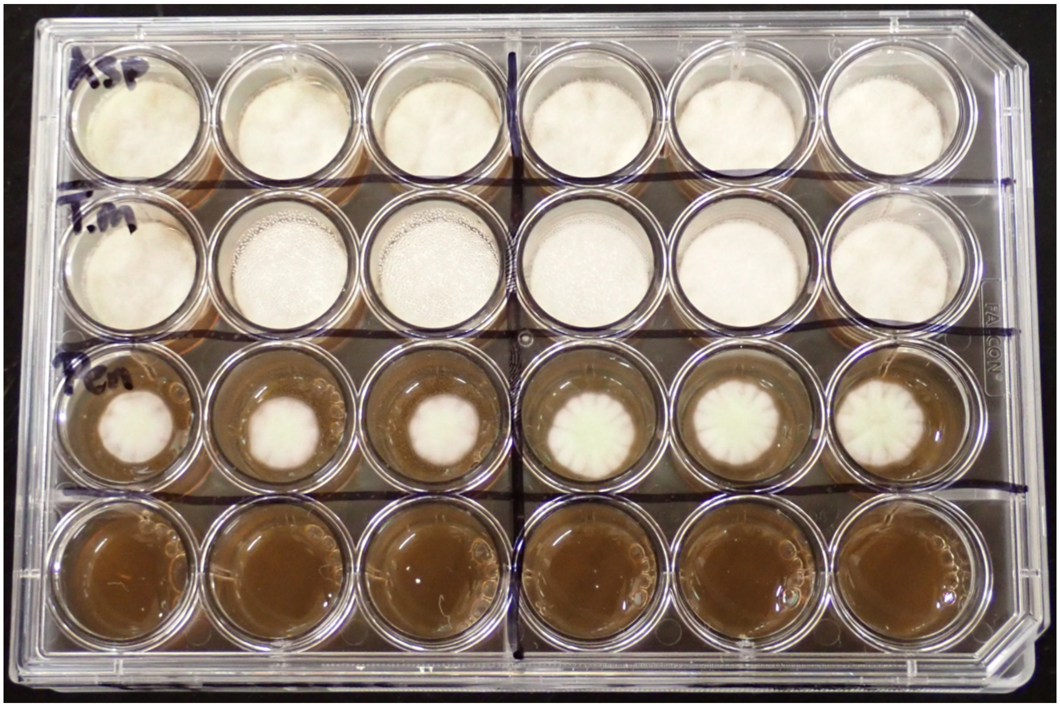

Figure S152. Control plate of *A. flavus* (top), *T. mentagrophytes* (second from the top), *P. chrysogenum* (third from the top) and media control (bottom) with blank DMSO added

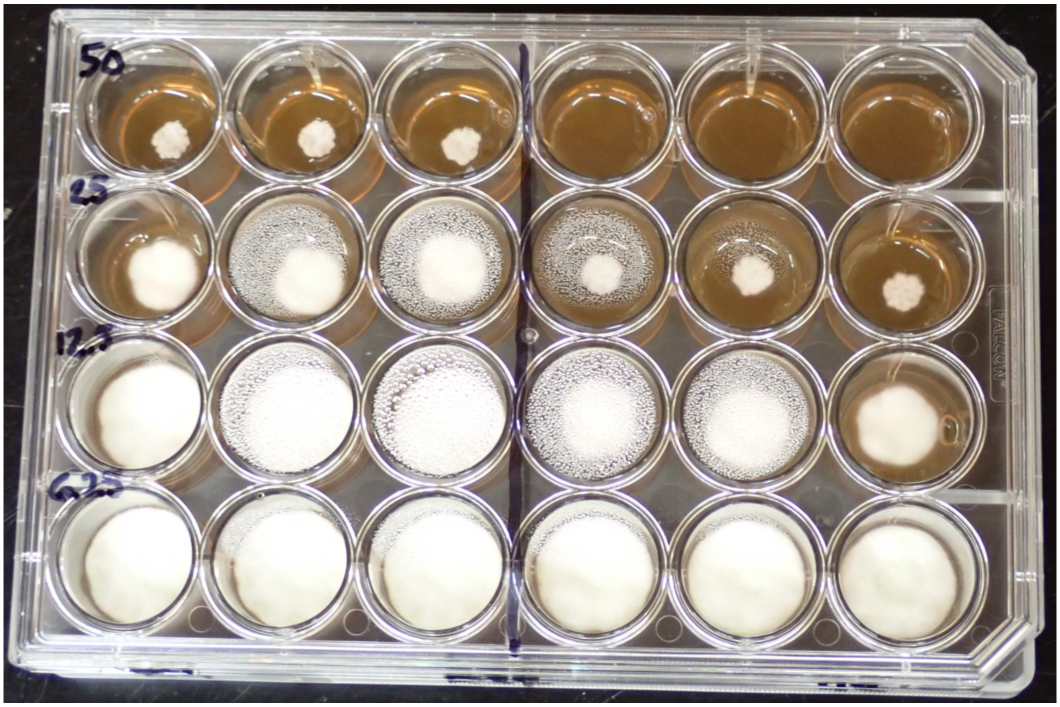

Figure S153. Compounds **6b** (left half) and **6e** (right half) at 50 (top row), 25, 12.5, and 6.25 (bottom row) μg/mL against *A. flavus*

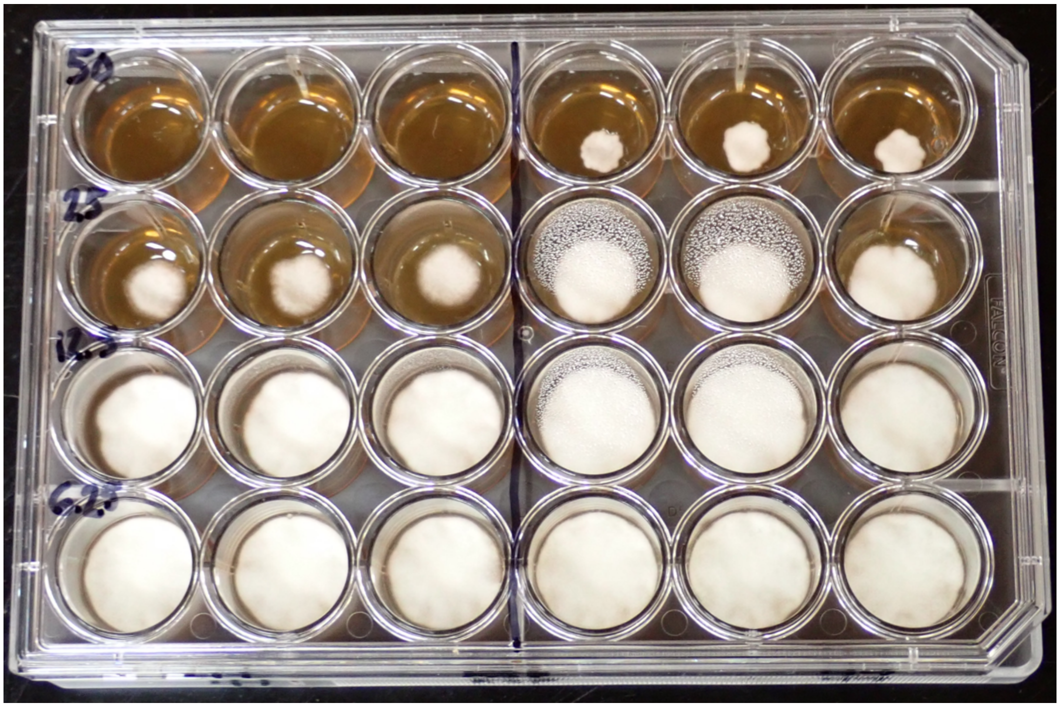

Figure S154. Compounds **6m** (left half) and **6a** (right half) at 50 (top row), 25, 12.5, and 6.25 (bottom row) μg/mL against *A. flavus*

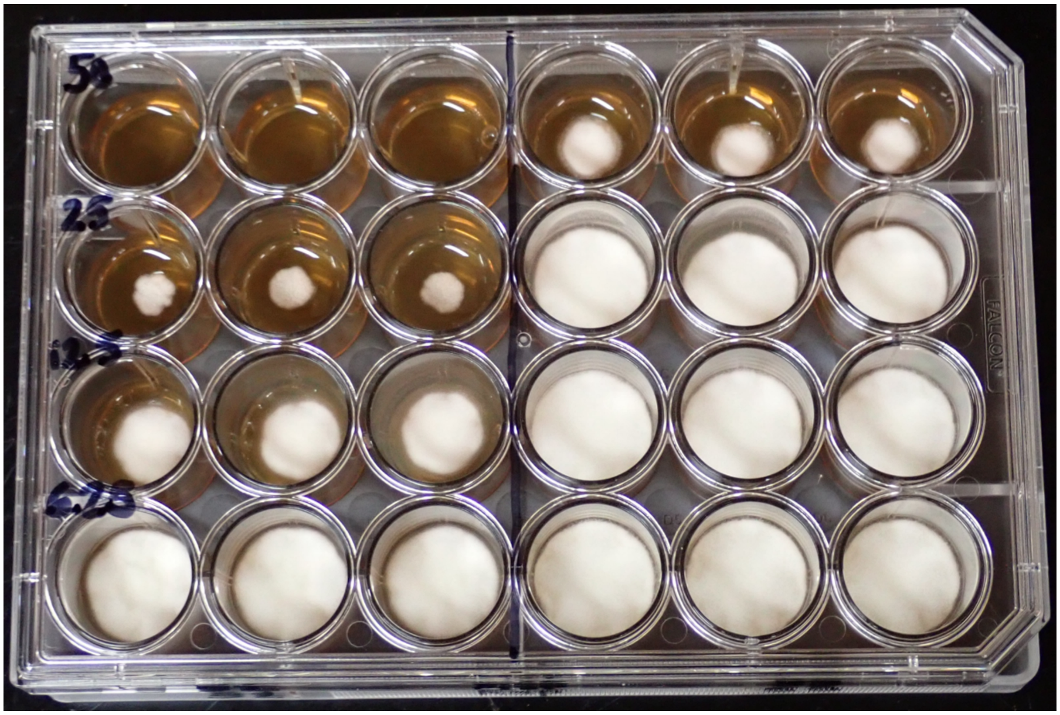

Figure S155. Compounds **6h** (left half) and **6w** (right half) at 50 (top row), 25, 12.5, and 6.25 (bottom row) μg/mL against *A. flavus*

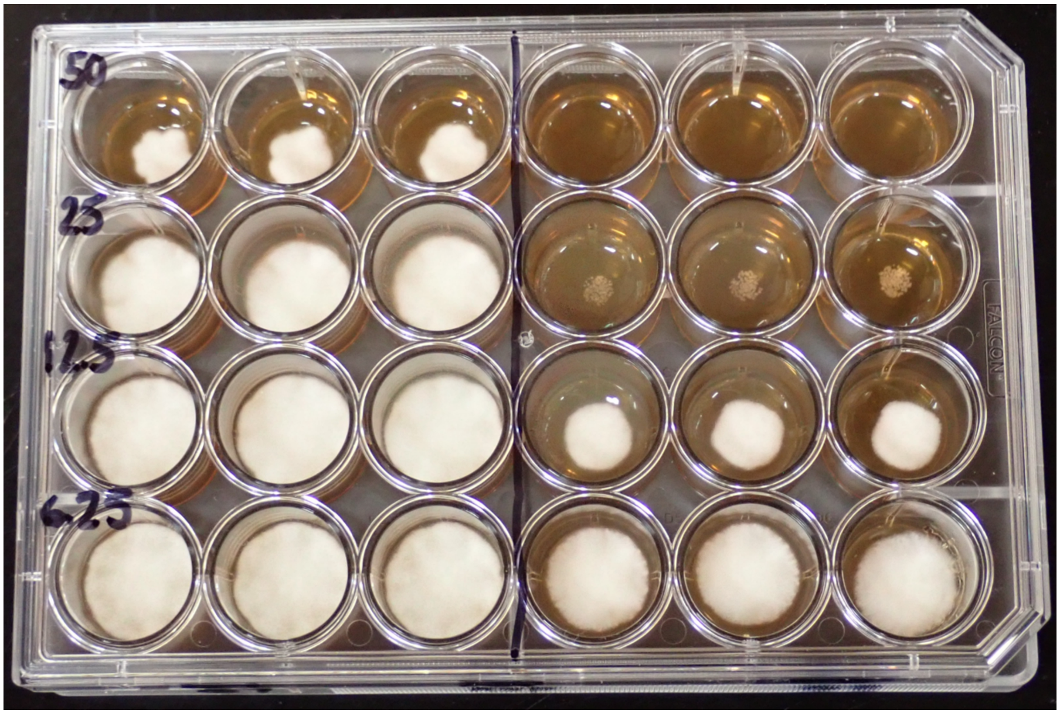

Figure S156. Compounds **6y** (left half) and **6f** (right half) at 50 (top row), 25, 12.5, and 6.25 (bottom row) μg/mL against *A. flavus*

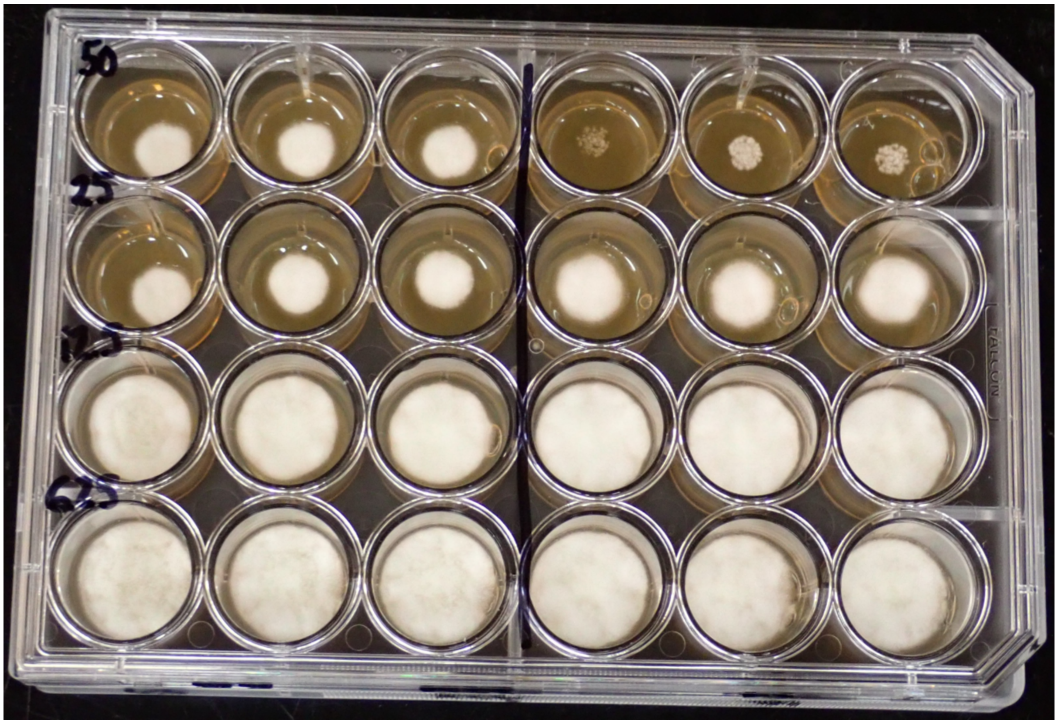

Figure S157. Compounds **6q** (left half) and **6k** (right half) at 50 (top row), 25, 12.5, and 6.25 (bottom row) μg/mL against *A. flavus*

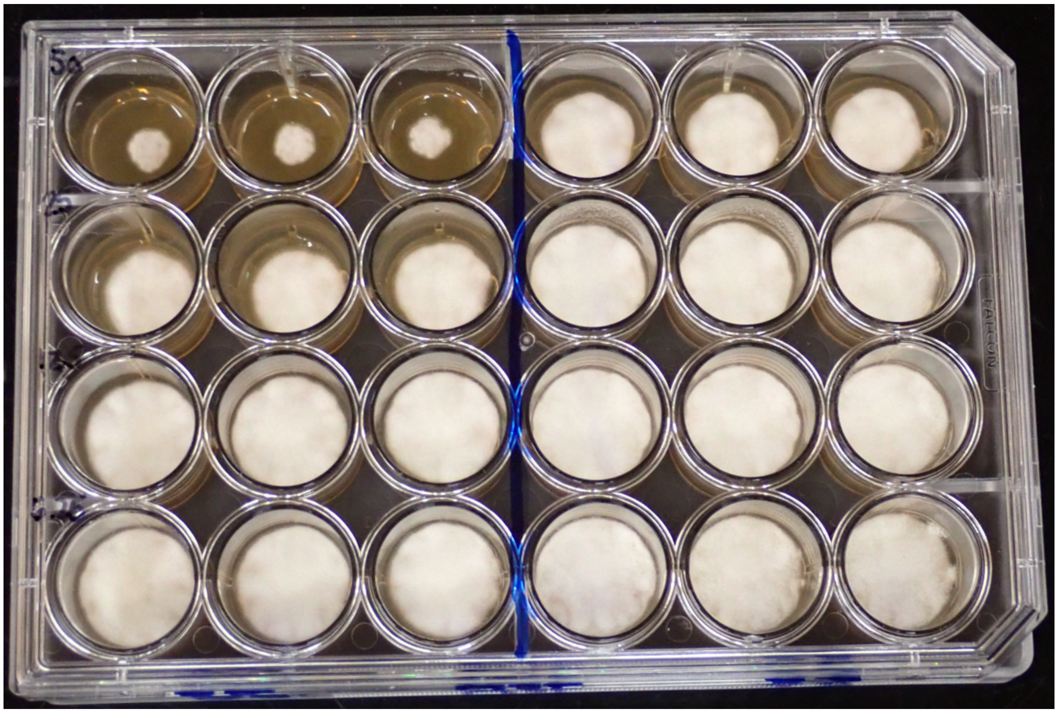

Figure S158. Compounds **6x** (left half) and **6s** (right half) at 50 (top row), 25, 12.5, and 6.25 (bottom row) µg/mL against *A. flavus*

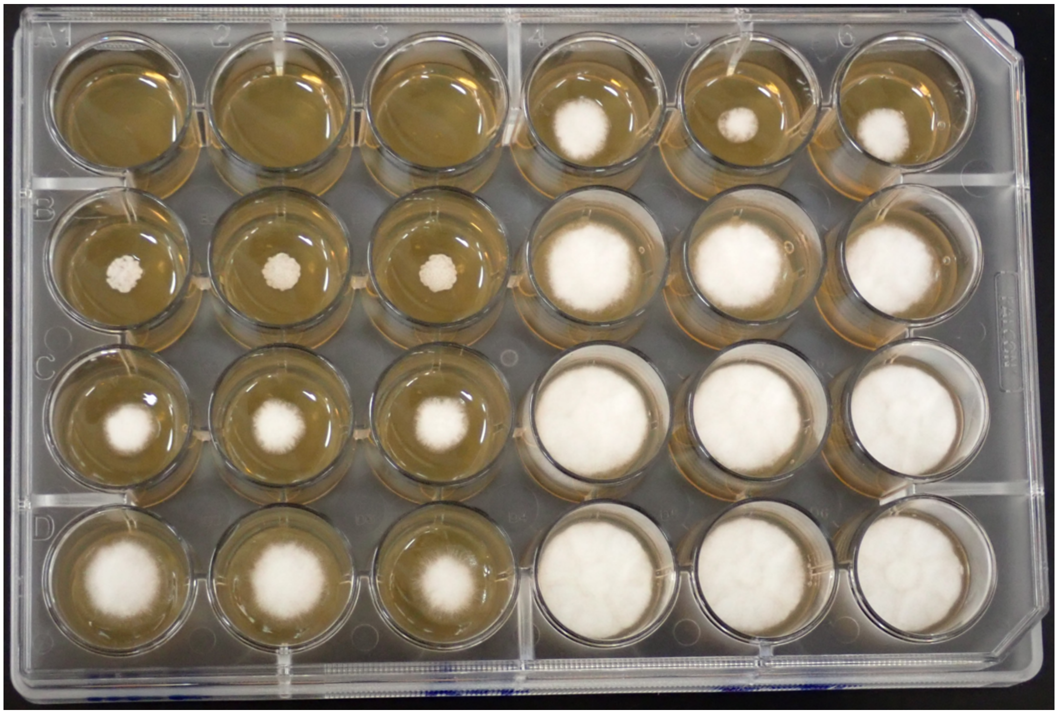

Figure S159. Compounds **6c** (left half) and **6j** (right half) at 50 (top row), 25, 12.5, and 6.25 (bottom row) µg/mL against *A. flavus*

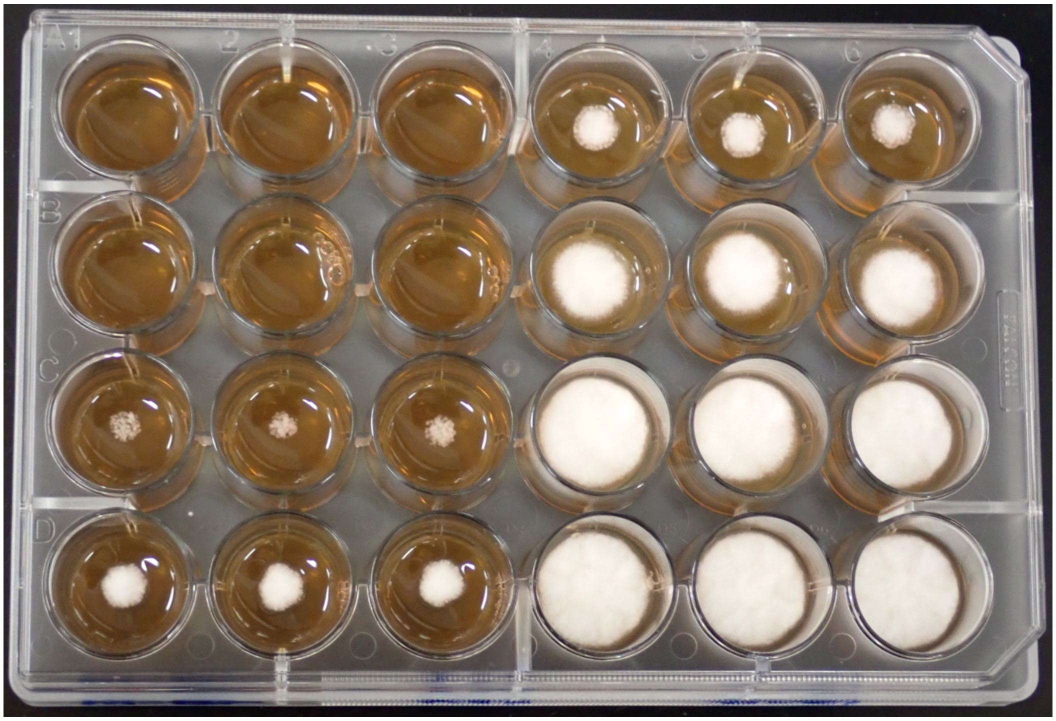

Figure S160. Compounds **6i** (left half) and **6g** (right half) at 50 (top row), 25, 12.5, and 6.25 (bottom row) µg/mL against *A. flavus*

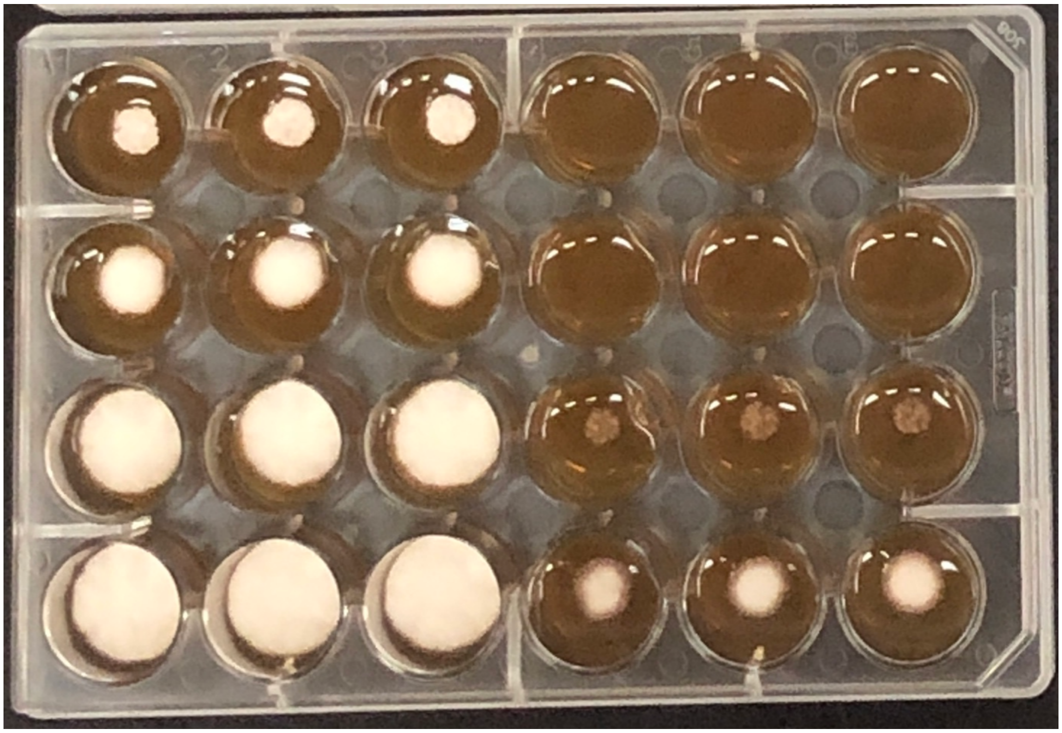

Figure S161. Compounds **6v** (left half) and **6l** (right half) at 50 (top row), 25, 12.5, and 6.25 (bottom row) µg/mL against *A. flavus*

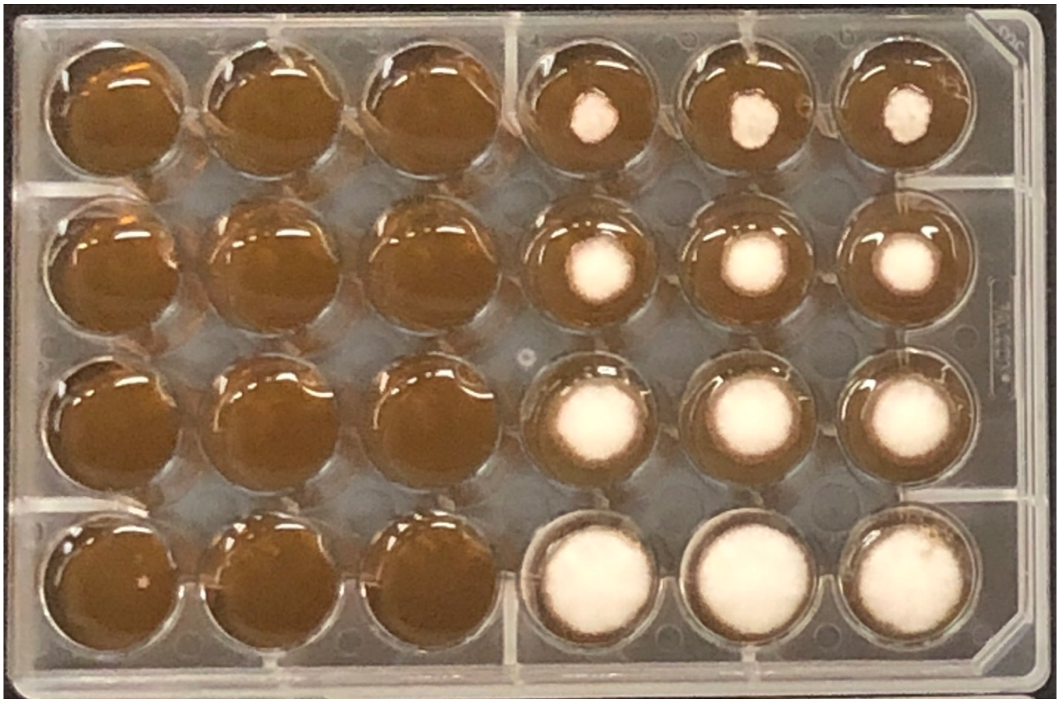

Figure S162. Compounds **1** (left half) and **6p** (right half) at 50 (top row), 25, 12.5, and 6.25 (bottom row) µg/mL against *A. flavus*

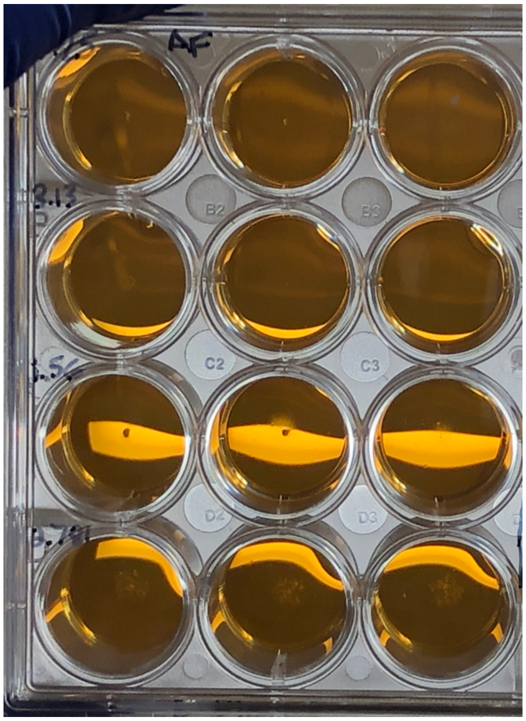

Figure S163. Follow-up solid assay of compound **1** at concentrations 6.25 (top row), 3.13, 1.56 and 0.78 (bottom row) µg/mL against *A. flavus*

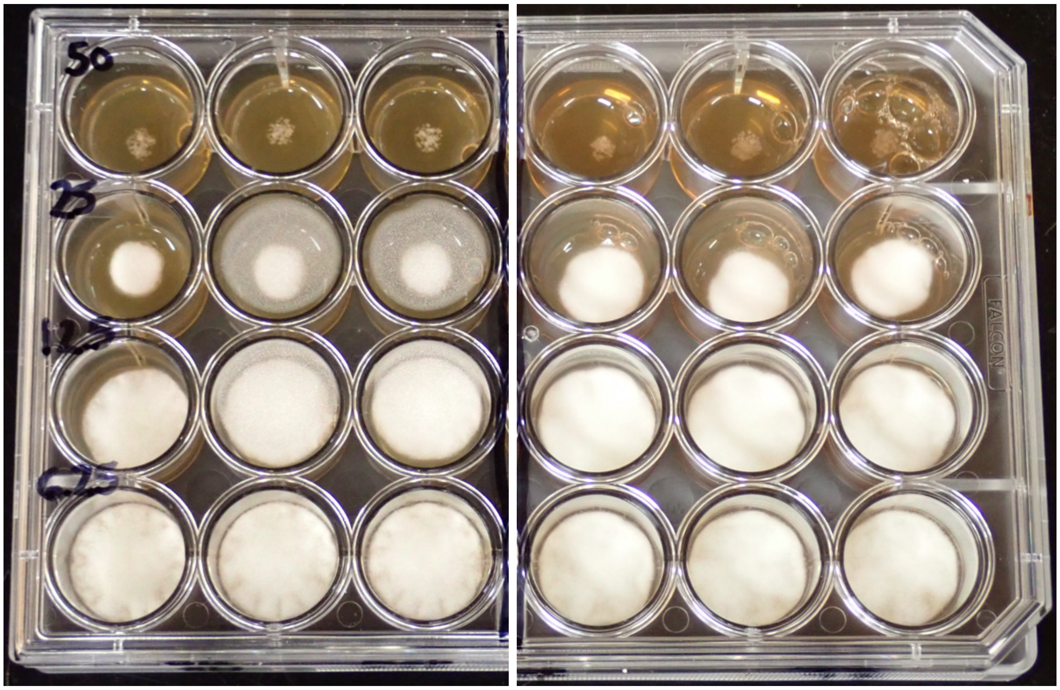

Figure S164. Compounds **6u** (left) and **6r** (right) at 50 (top row), 25, 12.5, and 6.25 (bottom row) µg/mL against *A. flavus*

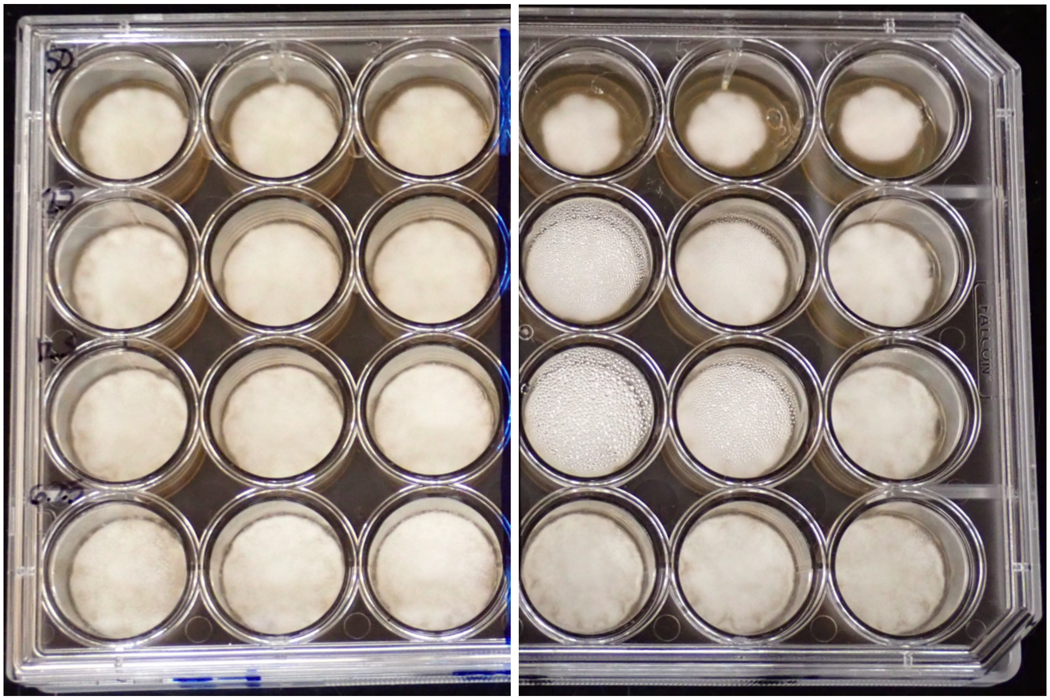

Figure S165. Compounds **6n** (left) and **6o** (right) at 50 (top row), 25, 12.5, and 6.25 (bottom row) µg/mL against *A. flavus*

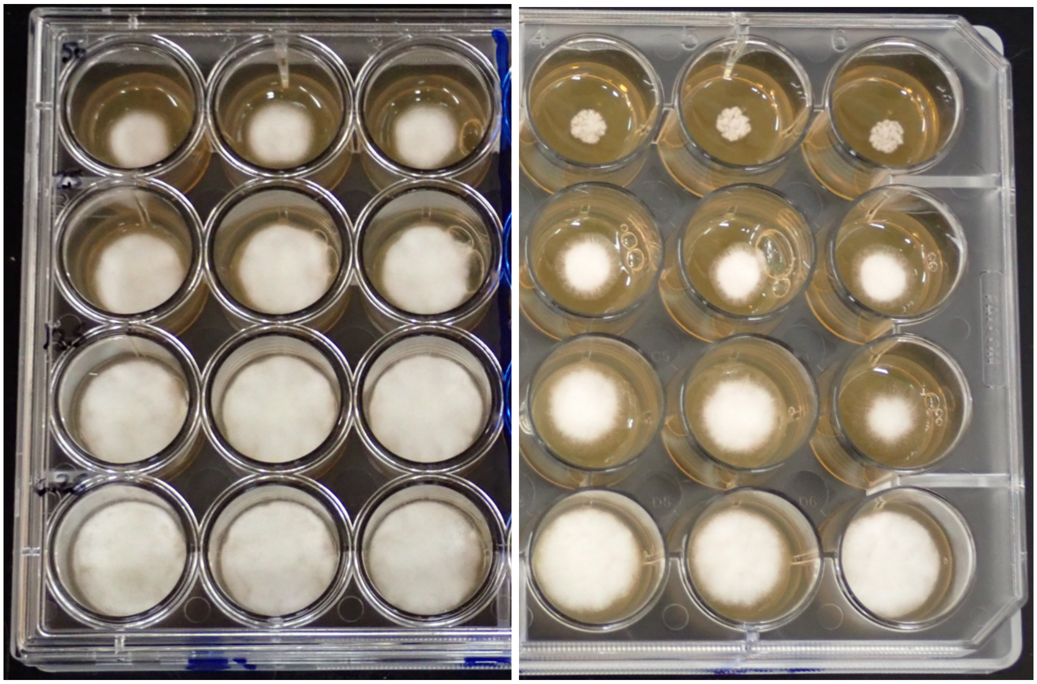

Figure S166. Compounds **6t** (left) and **6d** (right) at 50 (top row), 25, 12.5, and 6.25 (bottom row) µg/mL against *A. flavus*

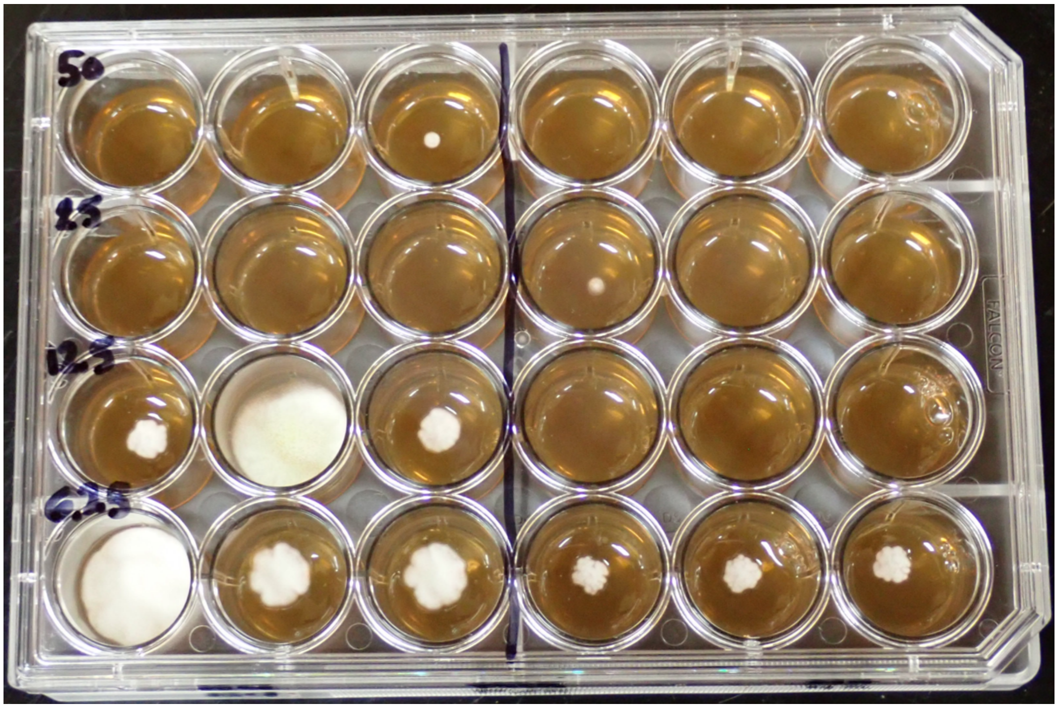

Figure S167. Compounds **6b** (left half) and **6e** (right half) at 50 (top row), 25, 12.5, and 6.25 (bottom row) µg/mL against *P. chrysogenum*. Contamination observed in wells A3, B4, C2, and D1

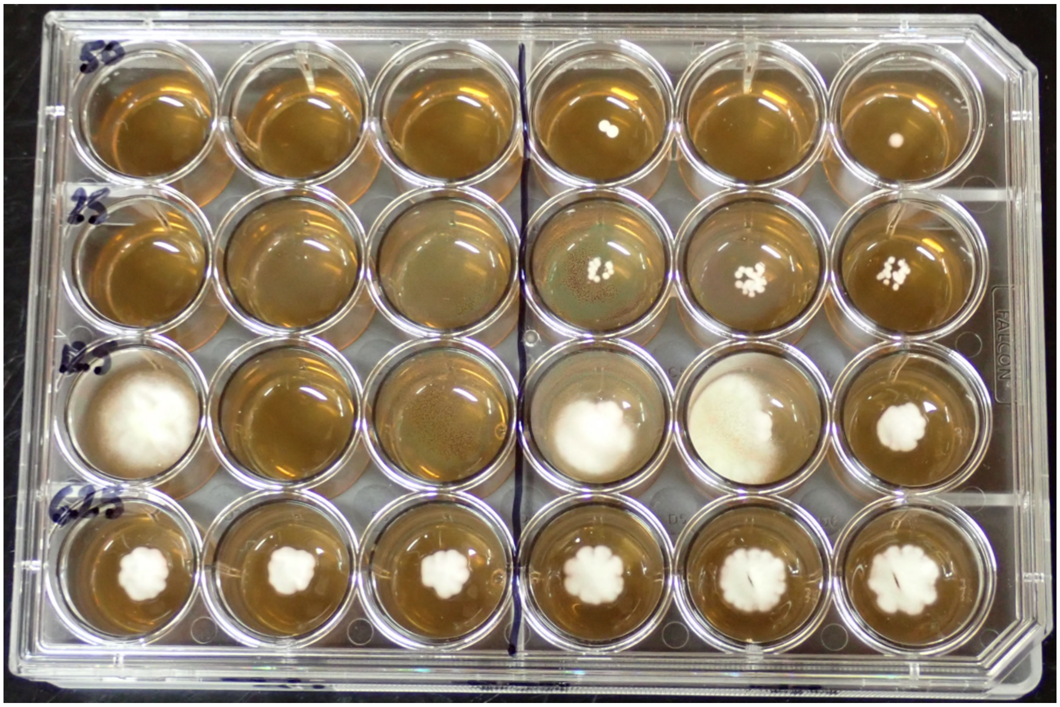

Figure S168. Compounds **6m** (left half) and **6a** (right half) at 50 (top row), 25, 12.5, and 6.25 (bottom row) µg/mL against *P. chrysogenum*. Contamination observed in wells C1,4-5

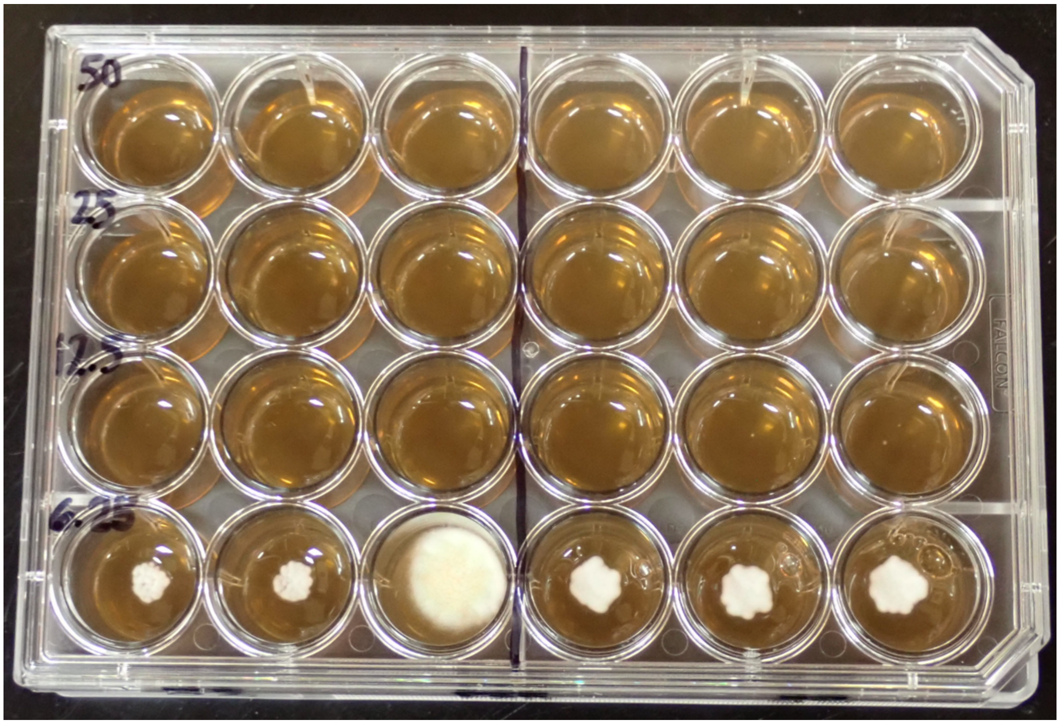

Figure S169. Compounds **6h** (left half) and **6w** (right half) at 50 (top row), 25, 12.5, and 6.25 (bottom row) µg/mL against *P. chrysogenum*. Contamination observed in well D3

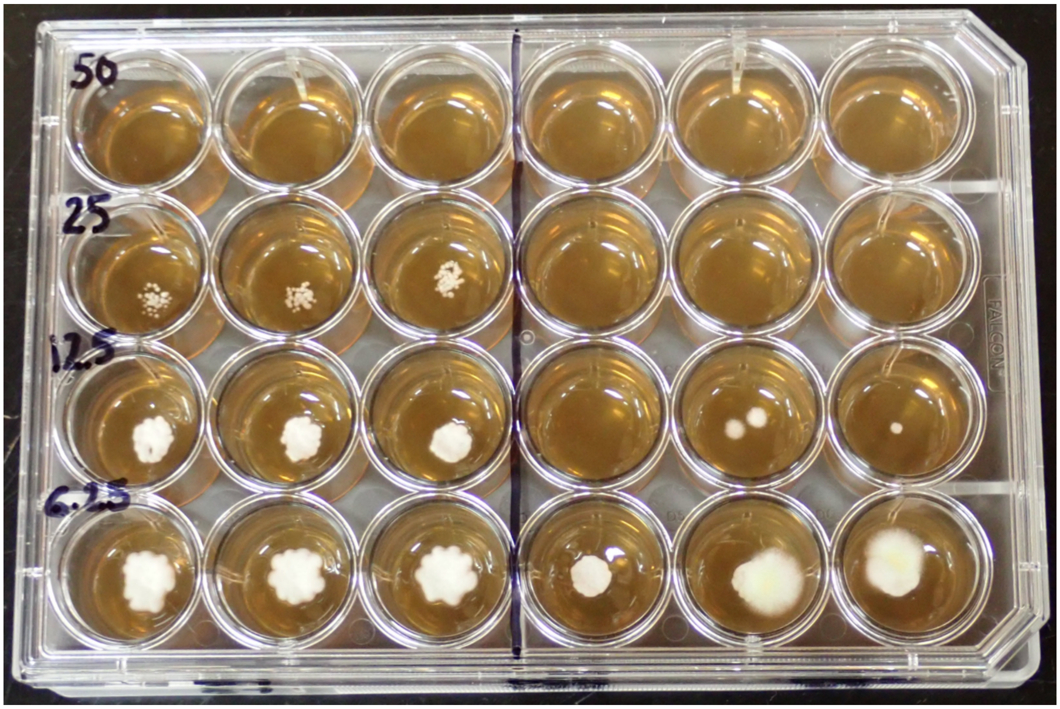

Figure S170. Compounds **6y** (left half) and **6f** (right half) at 50 (top row), 25, 12.5, and 6.25 (bottom row) µg/mL against *P. chrysogenum*. Contamination observed in wells D5-6

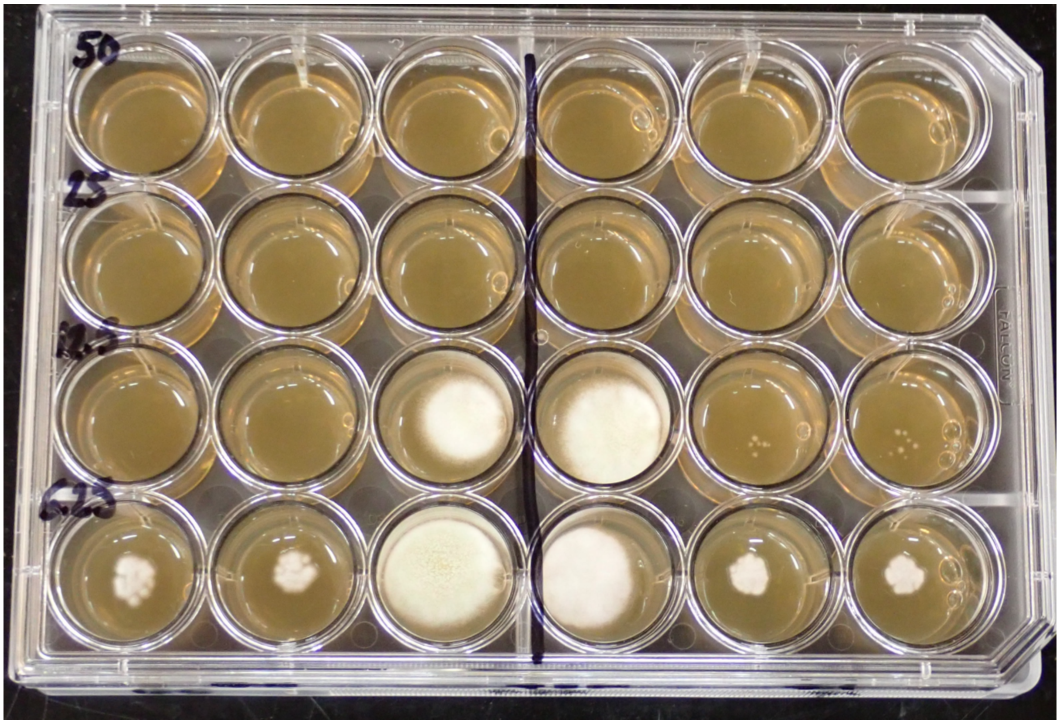

Figure S171. Compounds **6q** (left half) and **6k** (right half) at 50 (top row), 25, 12.5, and 6.25 (bottom row) µg/mL against *P. chrysogenum*. Contamination observed in wells C3-4 and D3-4

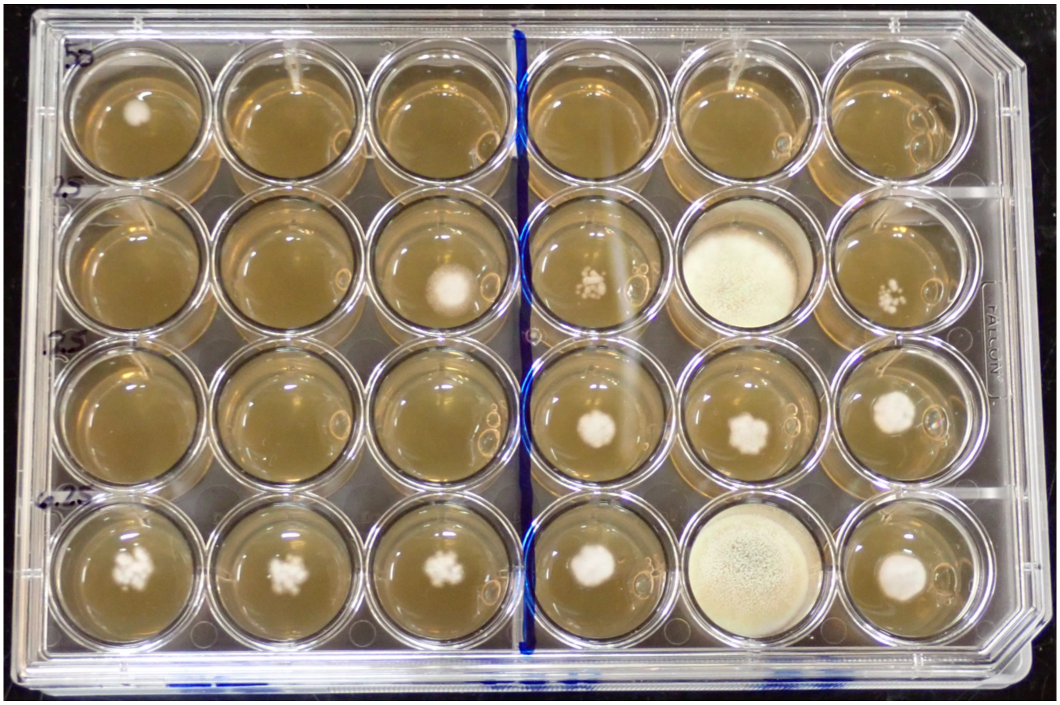

Figure S172. Compounds **6x** (left half) and **6s** (right half) at 50 (top row), 25, 12.5, and 6.25 (bottom row) µg/mL against *P. chrysogenum*. Contamination observed in wells A1, B3,5, and D5

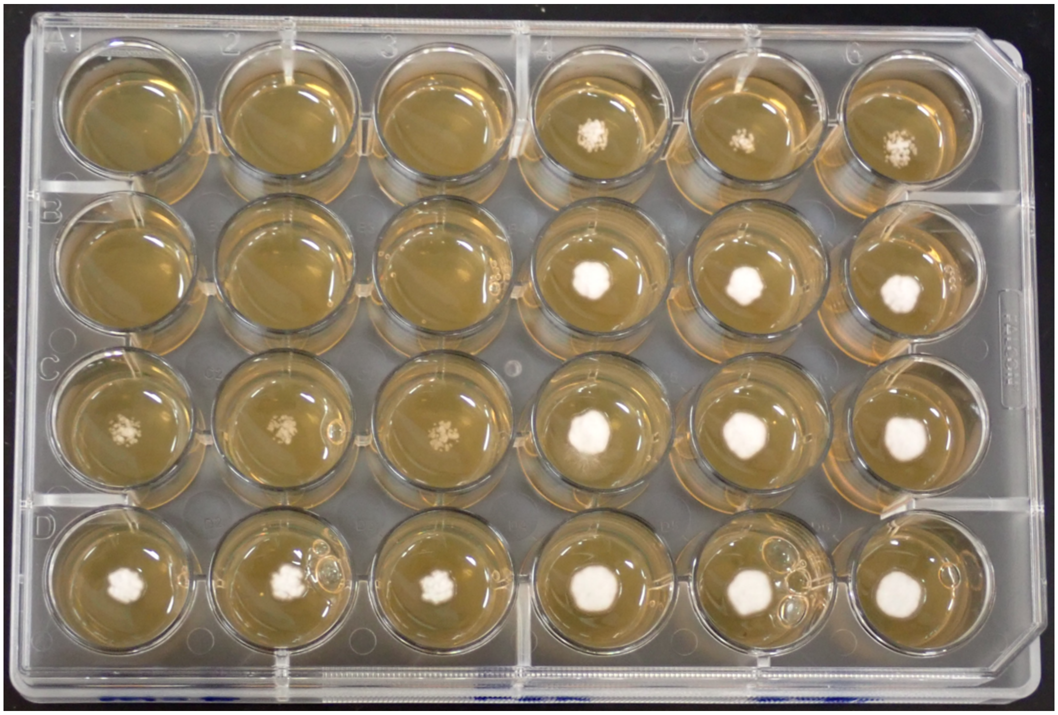

Figure S173. Compounds **6c** (left half) and **6j** (right half) at 50 (top row), 25, 12.5, and 6.25 (bottom row) µg/mL against *P. chrysogenum*

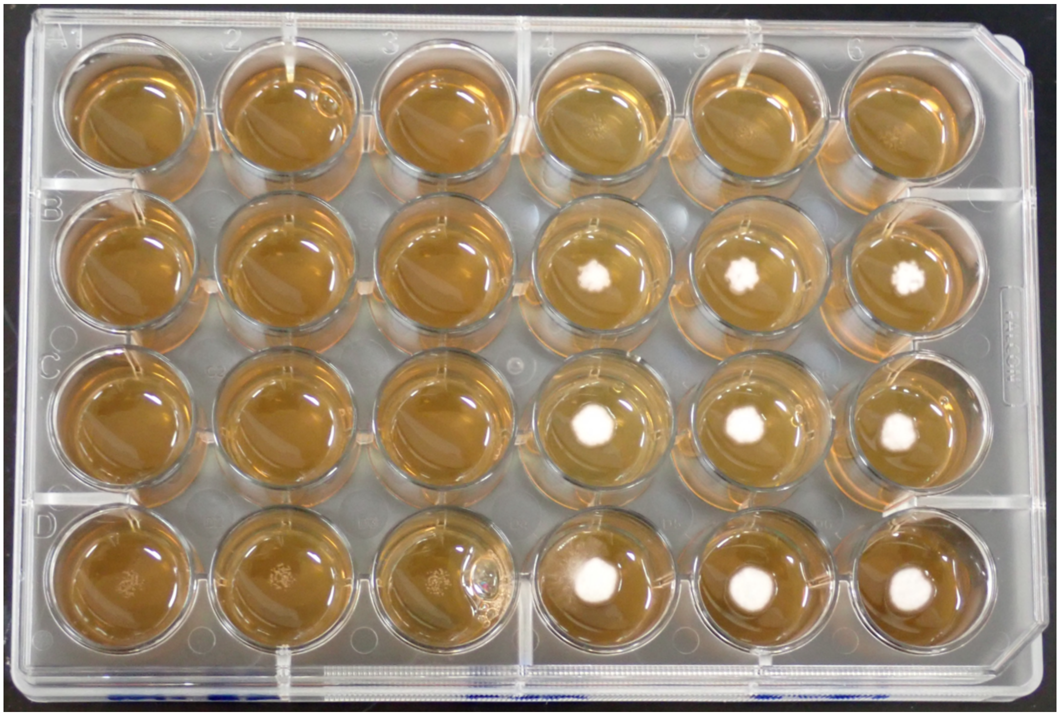

Figure S174. Compounds **6i** (left half) and **6g** (right half) at 50 (top row), 25, 12.5, and 6.25 (bottom row) μg/mL against *P. chrysogenum*

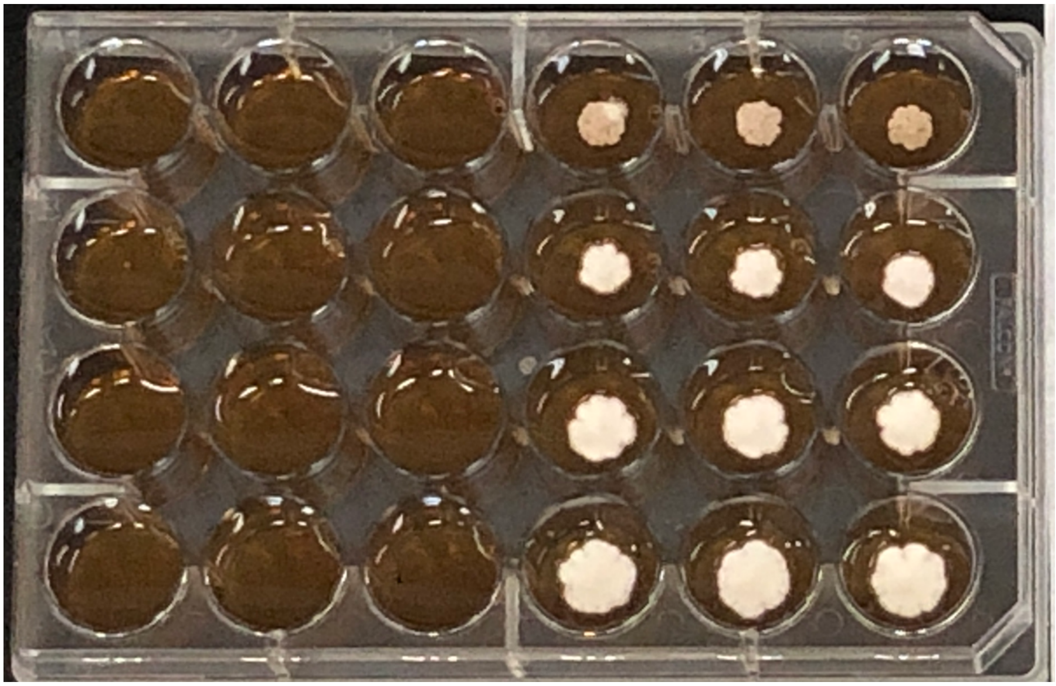

Figure S175. Compounds **1** (left half) and **6p** (right half) at 50 (top row), 25, 12.5, and 6.25 (bottom row) μg/mL against *P. chrysogenum*

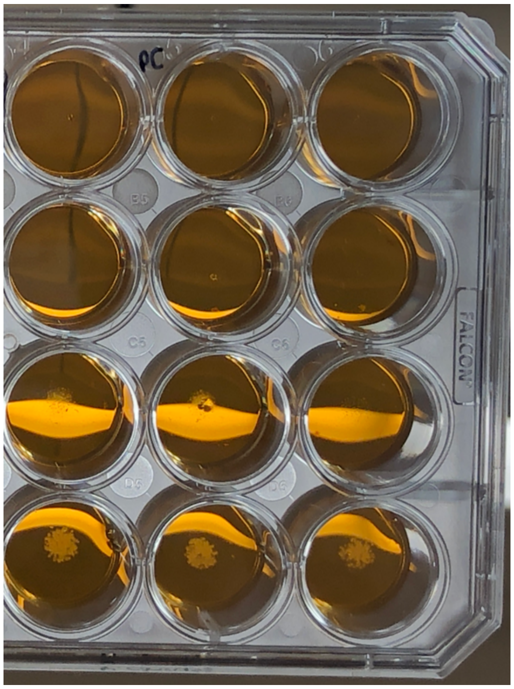

Figure S176. Follow-up solid assay of compound **1** at concentrations 6.25 (top row), 3.13, 1.56 and 0.78 (bottom row) µg/mL against *P. chrysogenum*

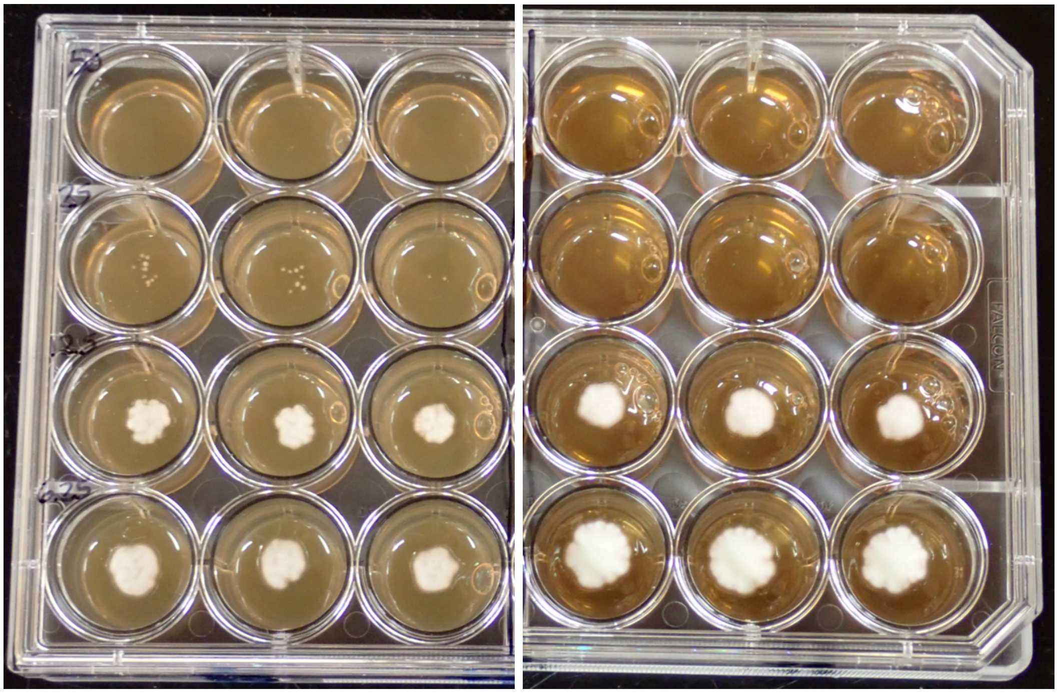

Figure S177. Compounds **6u** (left) and **6r** (right) at 50 (top row), 25, 12.5, and 6.25 (bottom row) µg/mL against *P. chrysogenum*

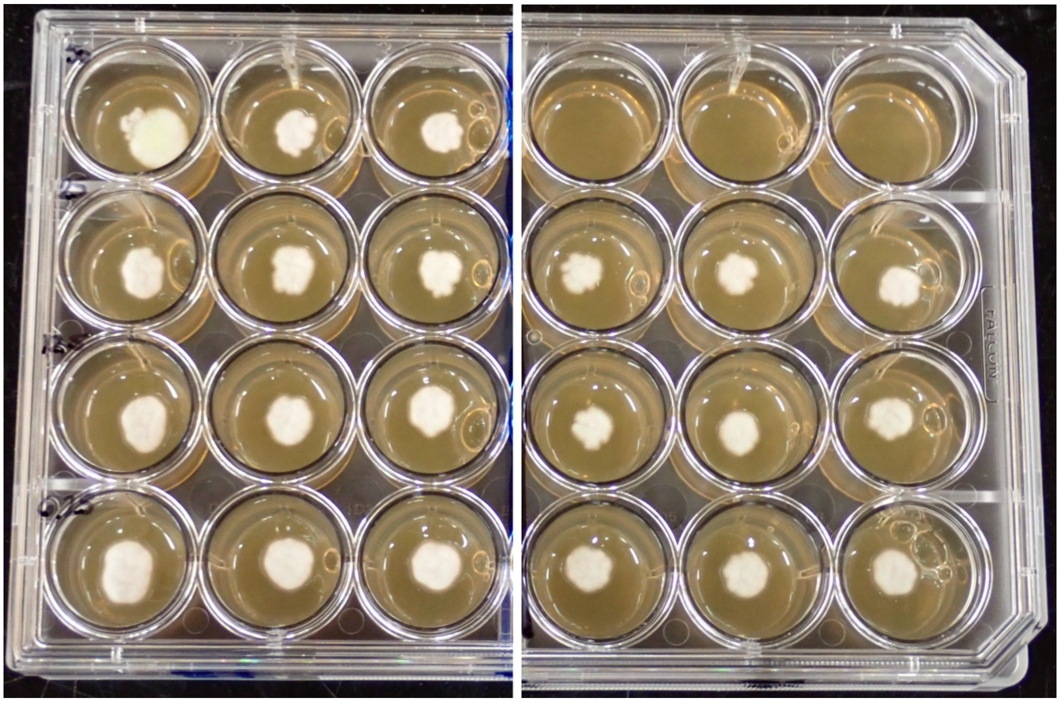

Figure S178. Compounds **6n** (left) and **6o** (right) at 50 (top row), 25, 12.5, and 6.25 (bottom row) µg/mL against *P. chrysogenum*. Contamination observed in well A1

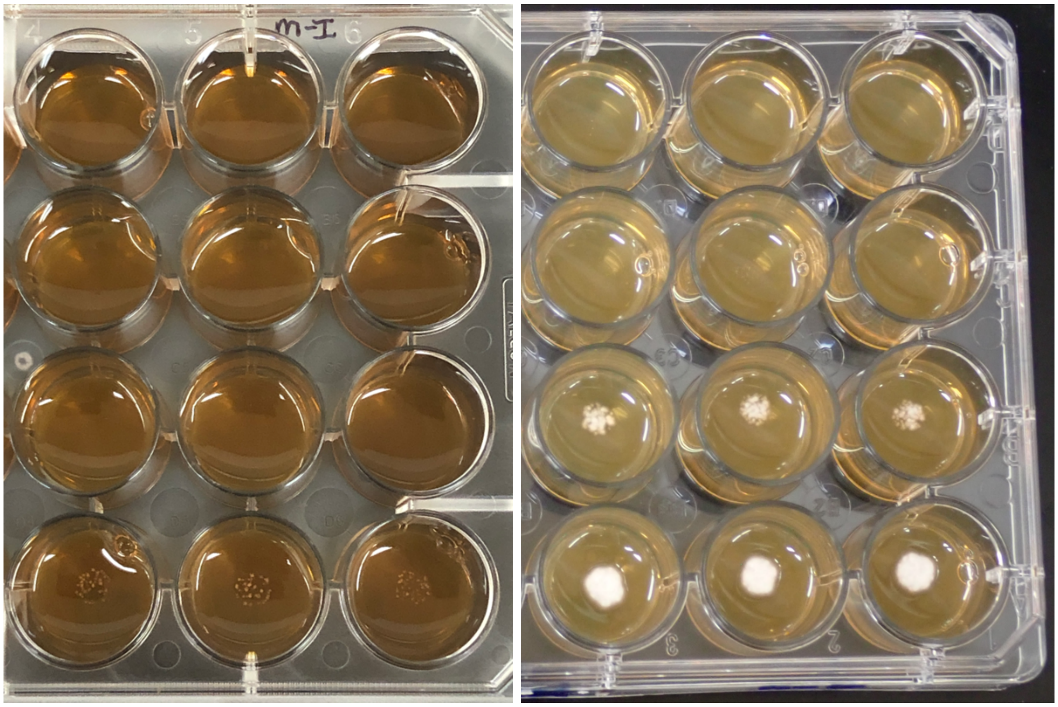

Figure S179. Compounds **6l** (left) and **6d** (right) at 50 (top row), 25, 12.5, and 6.25 (bottom row) μg/mL against *P. chrysogenum*

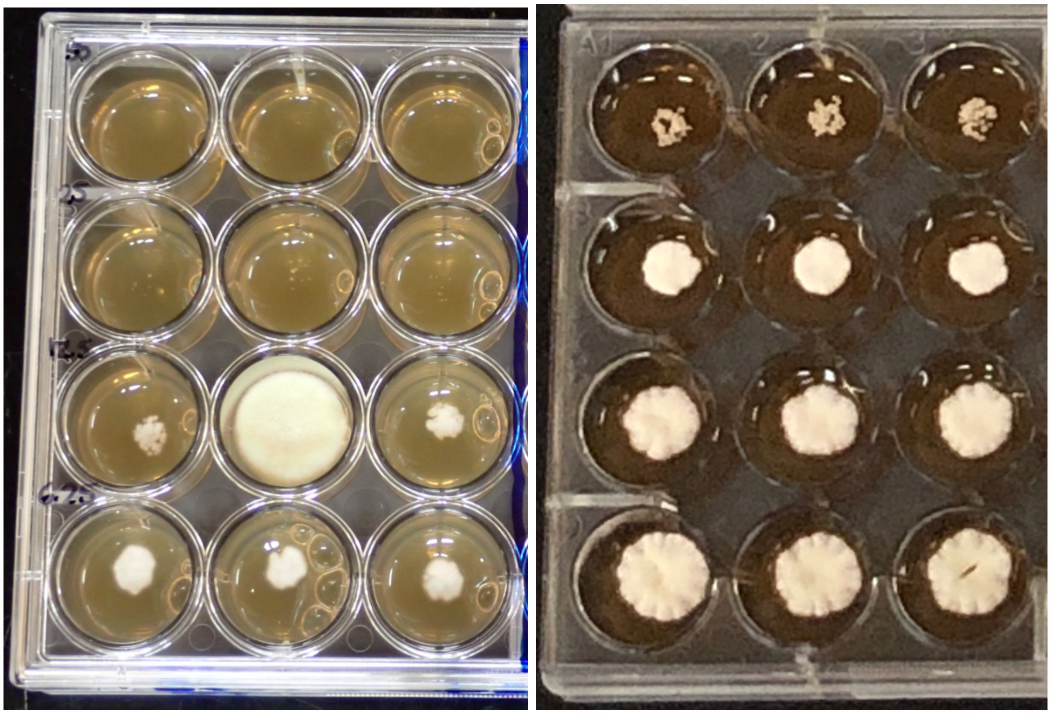

Figure S180. Compounds **6t** (left) and **6w** (right) at 50 (top row), 25, 12.5, and 6.25 (bottom row) μg/mL against *P. chrysogenum*. Contamination observed in well C2 (left)

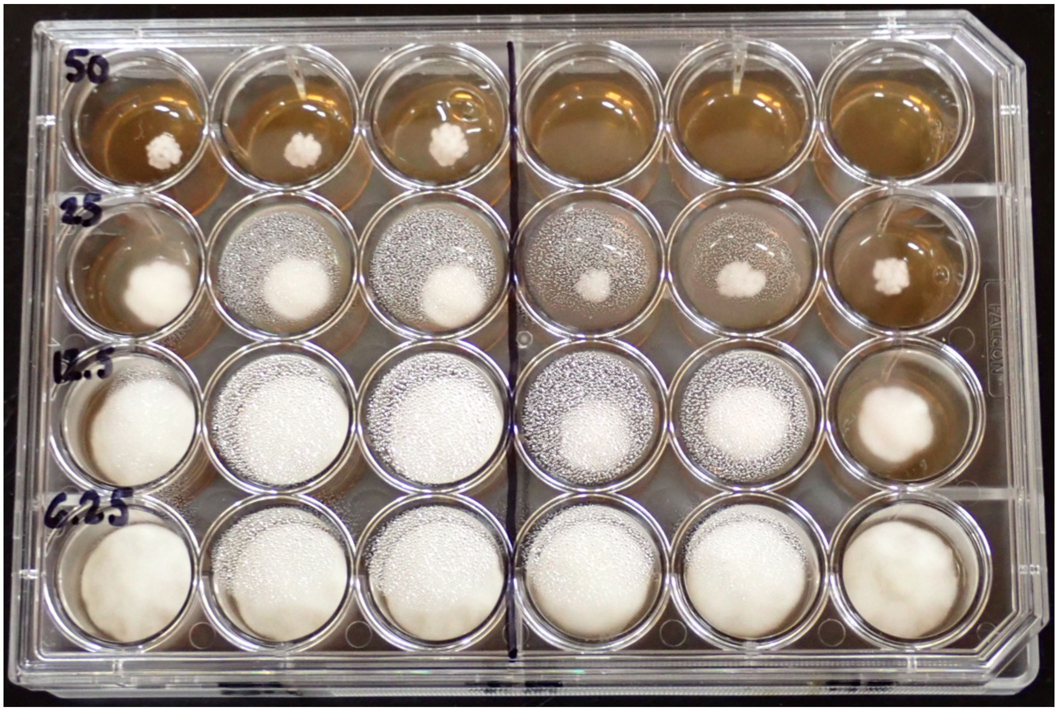

Figure S181. Compounds **6b** (left half) and **6e** (right half) at 50 (top row), 25, 12.5, and 6.25 (bottom row) μg/mL against *T. mentagrophytes*

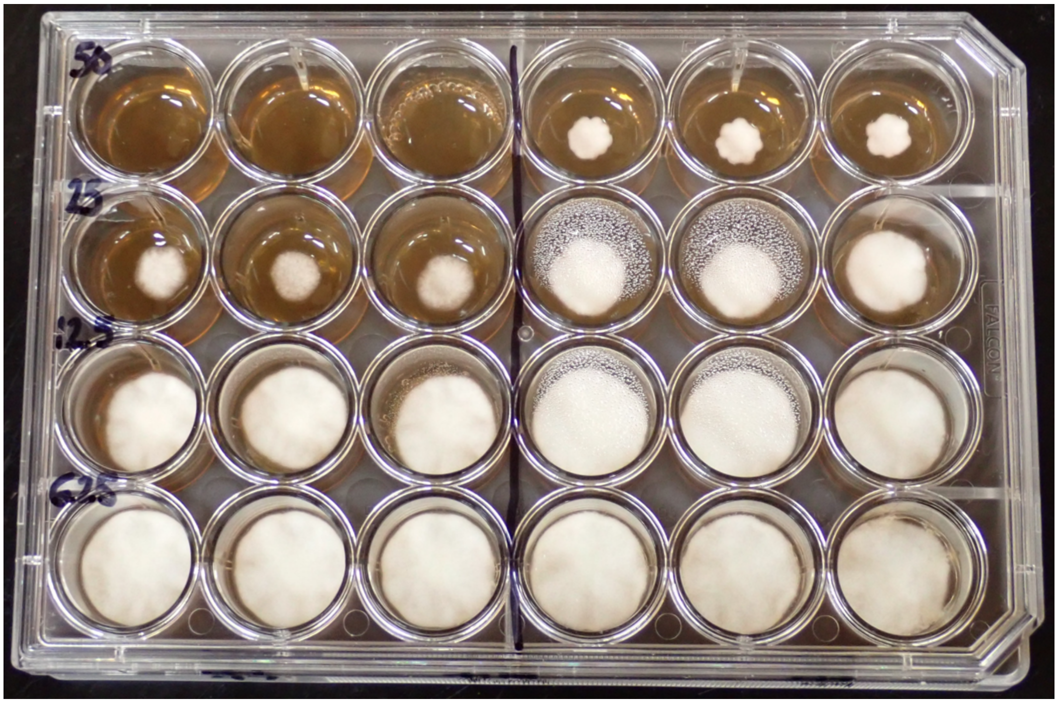

Figure S182. Compounds **6m** (left half) and **6a** (right half) at 50 (top row), 25, 12.5, and 6.25 (bottom row) μg/mL against *T. mentagrophytes*

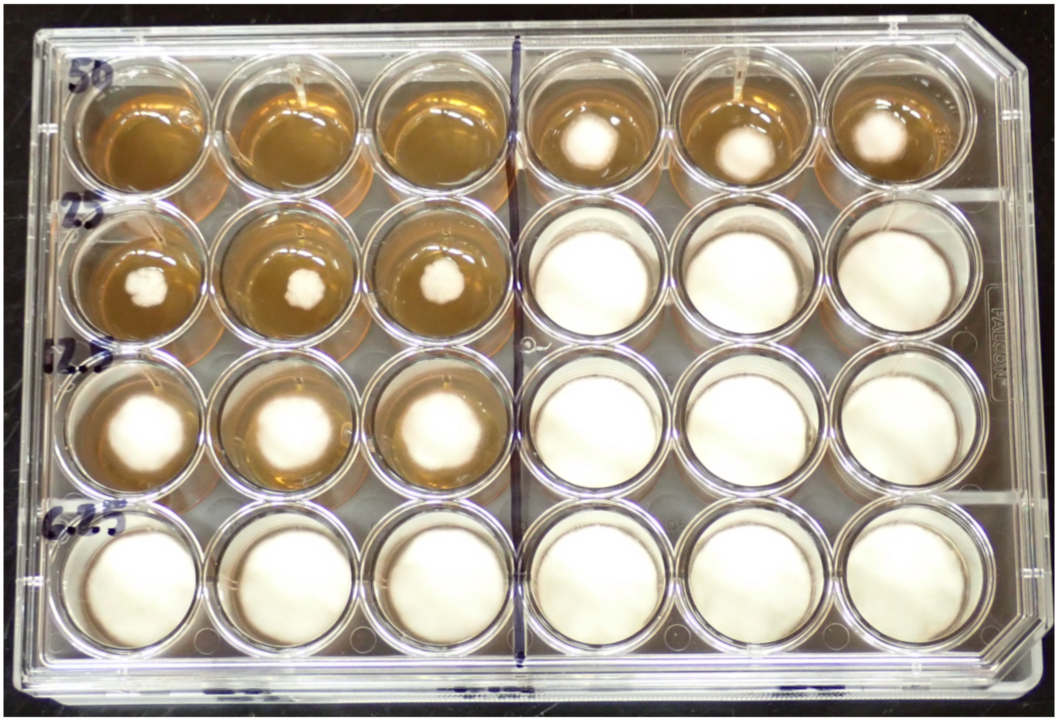

Figure S183. Compounds **6h** (left half) and **6w** (right half) at 50 (top row), 25, 12.5, and 6.25 (bottom row) µg/mL against *T. mentagrophytes*

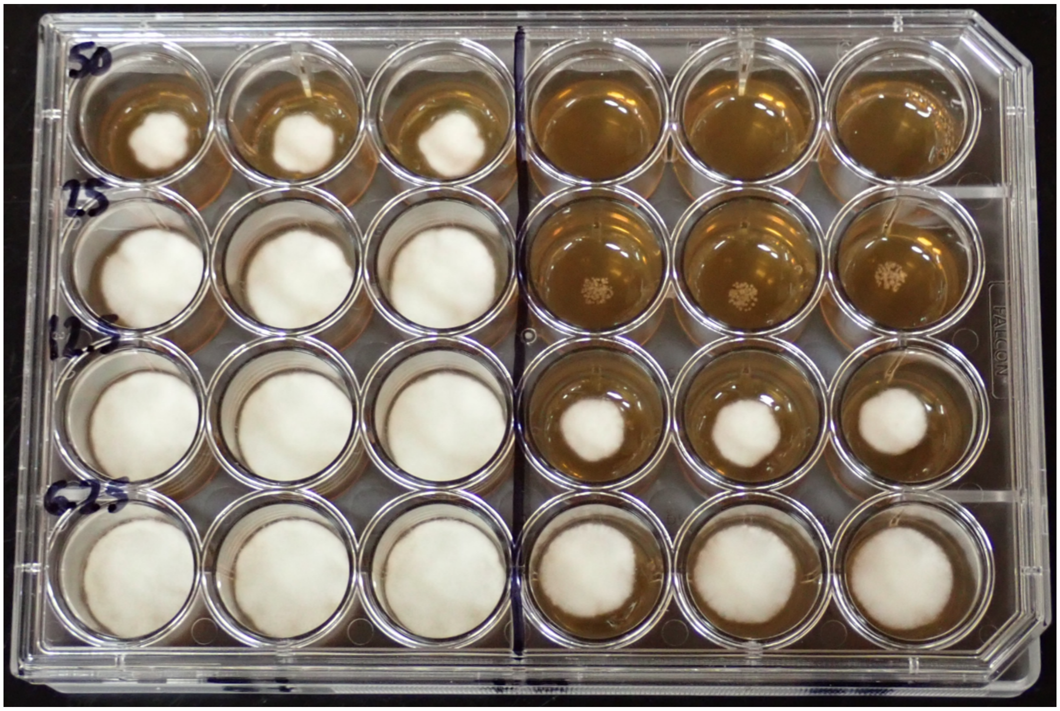

Figure S184. Compounds **6y** (left half) and **6f** (right half) at 50 (top row), 25, 12.5, and 6.25 (bottom row) µg/mL against *T. mentagrophytes*

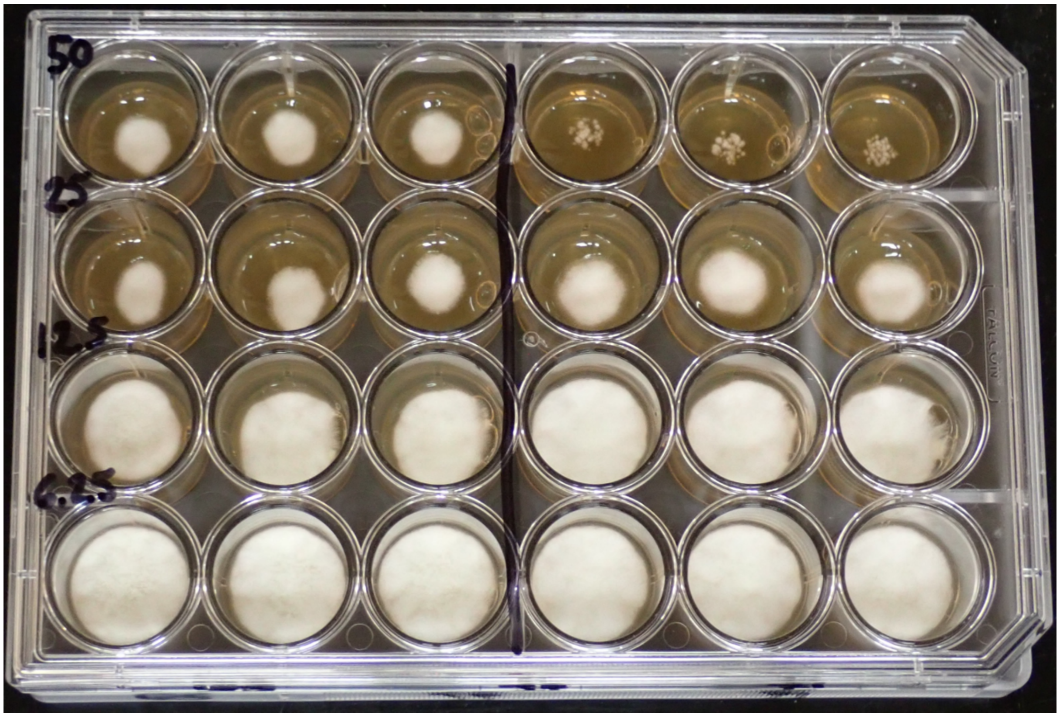

Figure S185. Compounds **6q** (left half) and **6k** (right half) at 50 (top row), 25, 12.5, and 6.25 (bottom row) µg/mL against *T. mentagrophytes*

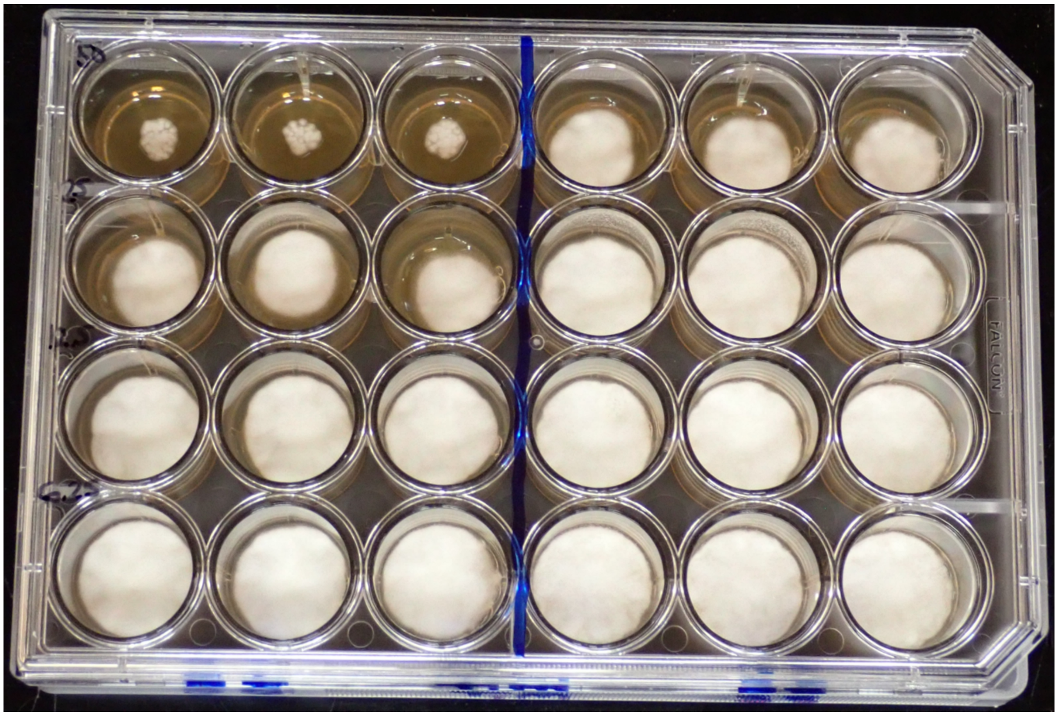

Figure S186. Compounds **6x** (left half) and **6s** (right half) at 50 (top row), 25, 12.5, and 6.25 (bottom row) µg/mL against *T. mentagrophytes*

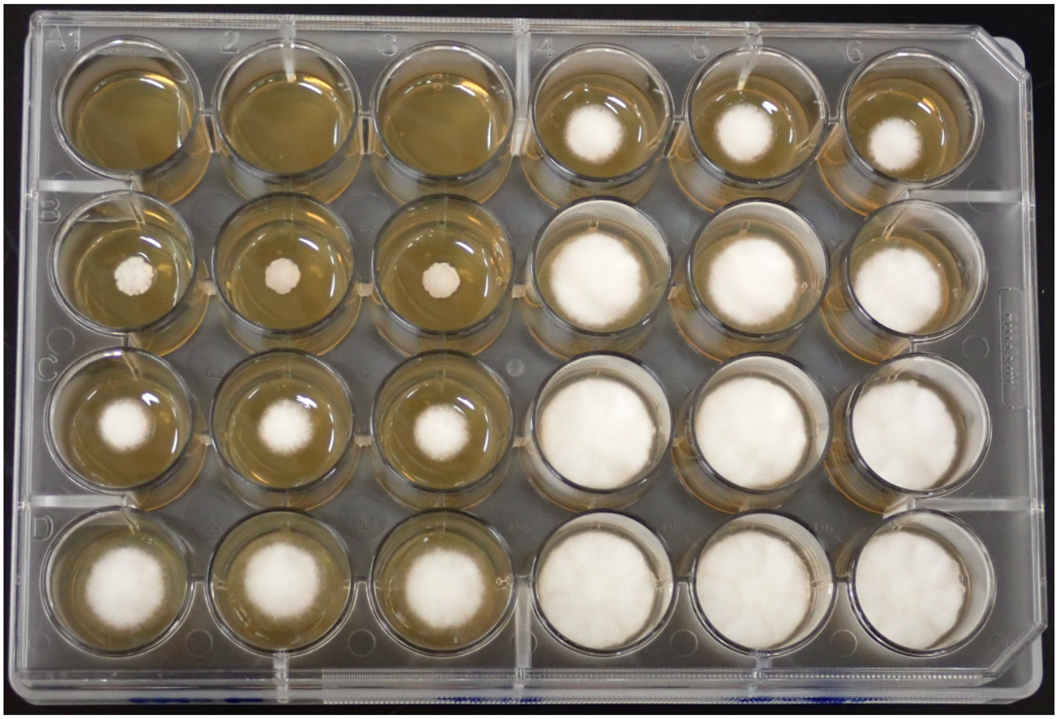

Figure S187. Compounds **6c** (left half) and **6j** (right half) at 50 (top row), 25, 12.5, and 6.25 (bottom row) µg/mL against *T. mentagrophytes*

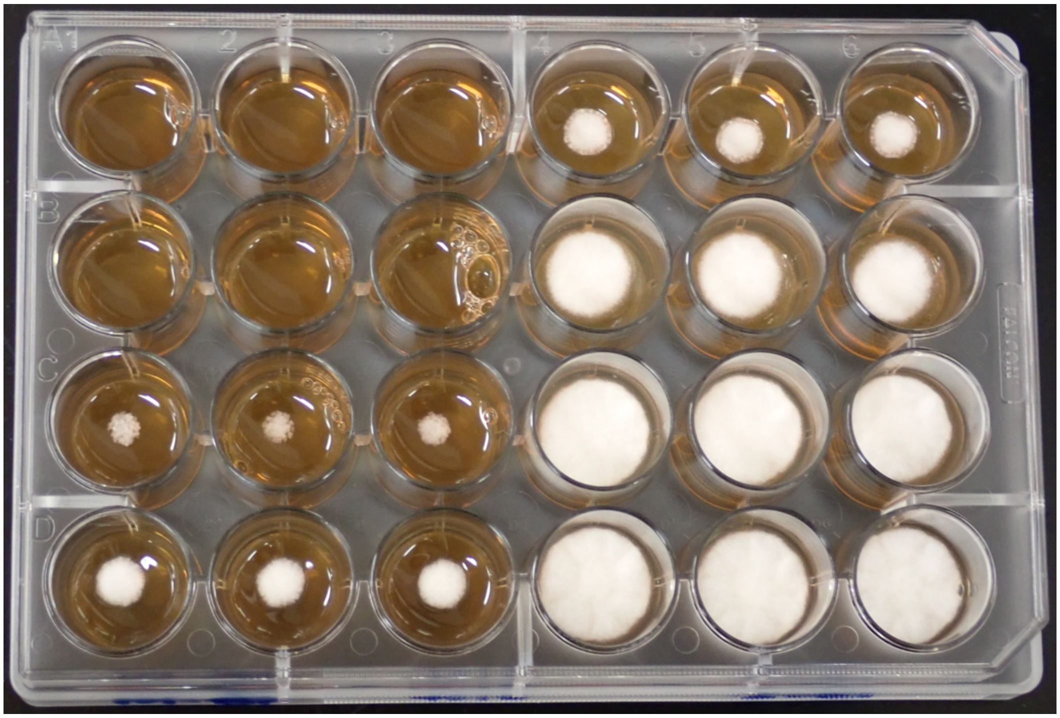

Figure S188. Compounds **6i** (left half) and **6g** (right half) at 50 (top row), 25, 12.5, and 6.25 (bottom row) µg/mL against *T. mentagrophytes*

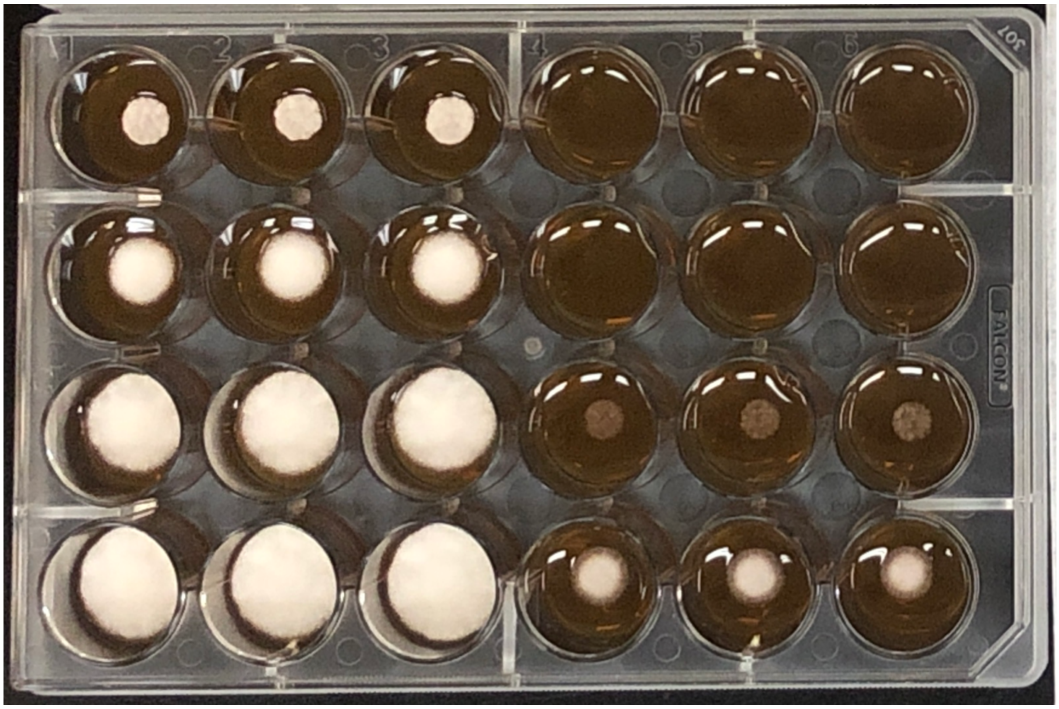

Figure S189. Compounds **6v** (left half) and **6l** (right half) at 50 (top row), 25, 12.5, and 6.25 (bottom row) µg/mL against *T. mentagrophytes*

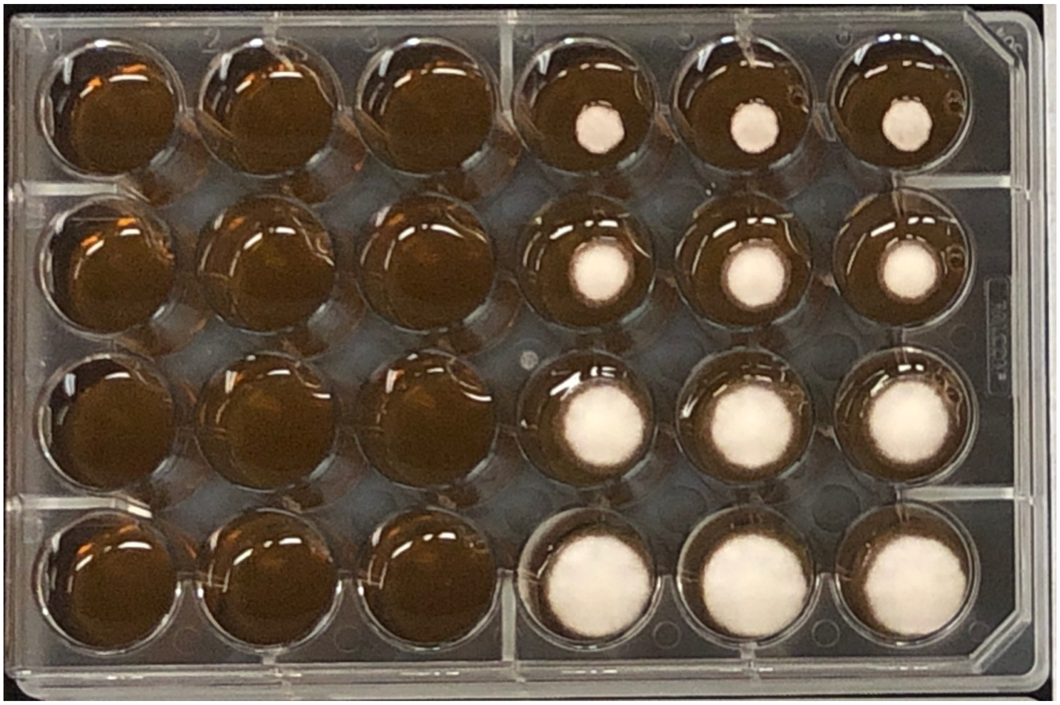

Figure S190. Compounds **1** (left half) and **6p** (right half) at 50 (top row), 25, 12.5, and 6.25 (bottom row) µg/mL against *T. mentagrophytes*

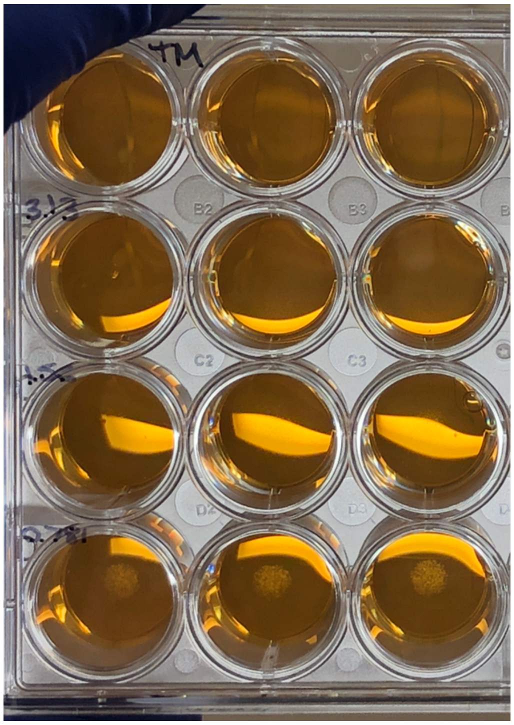

Figure S191. Follow-up solid assay of compound **1** at concentrations 6.25 (top row), 3.13, 1.56 and 0.78 (bottom row) μg/mL against *T. mentagrophytes*

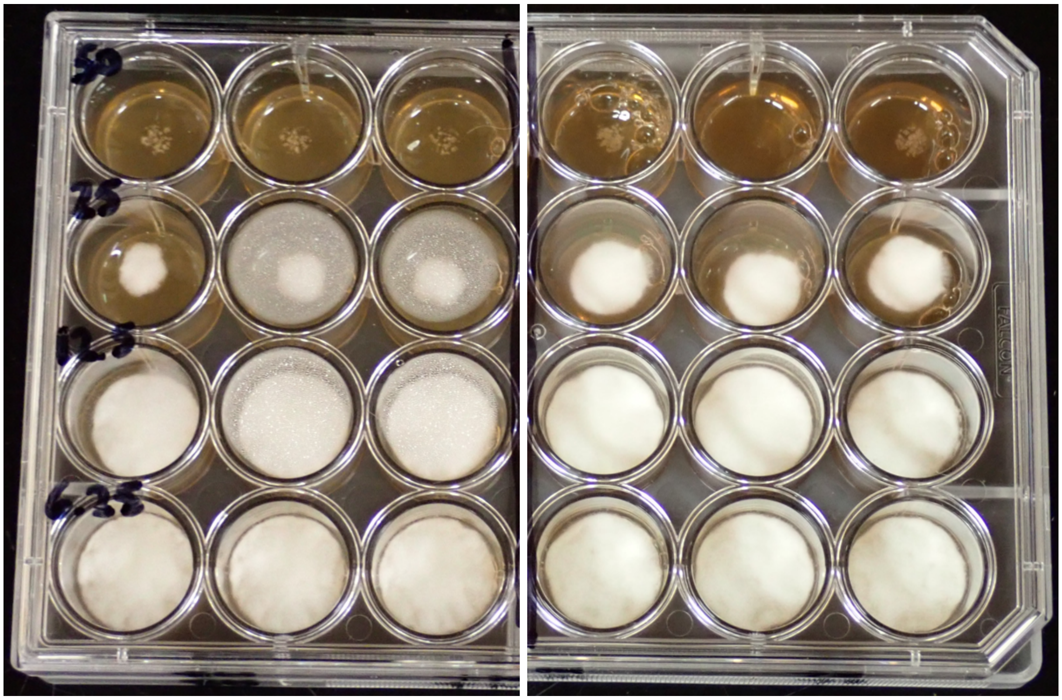

Figure S192. Compounds **6u** (left) and **6r** (right) at 50 (top row), 25, 12.5, and 6.25 (bottom row) μg/mL against *T. mentagrophytes*

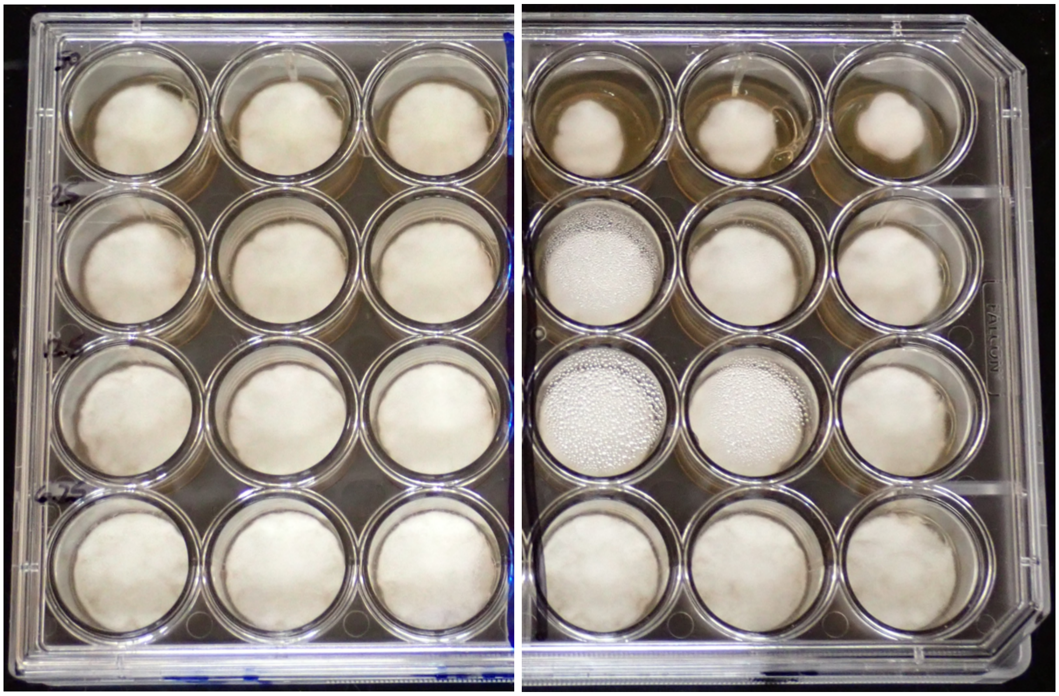

Figure S193. Compounds **6n** (left) and **6o** (right) at 50 (top row), 25, 12.5, and 6.25 (bottom row) μg/mL against *T. mentagrophytes*

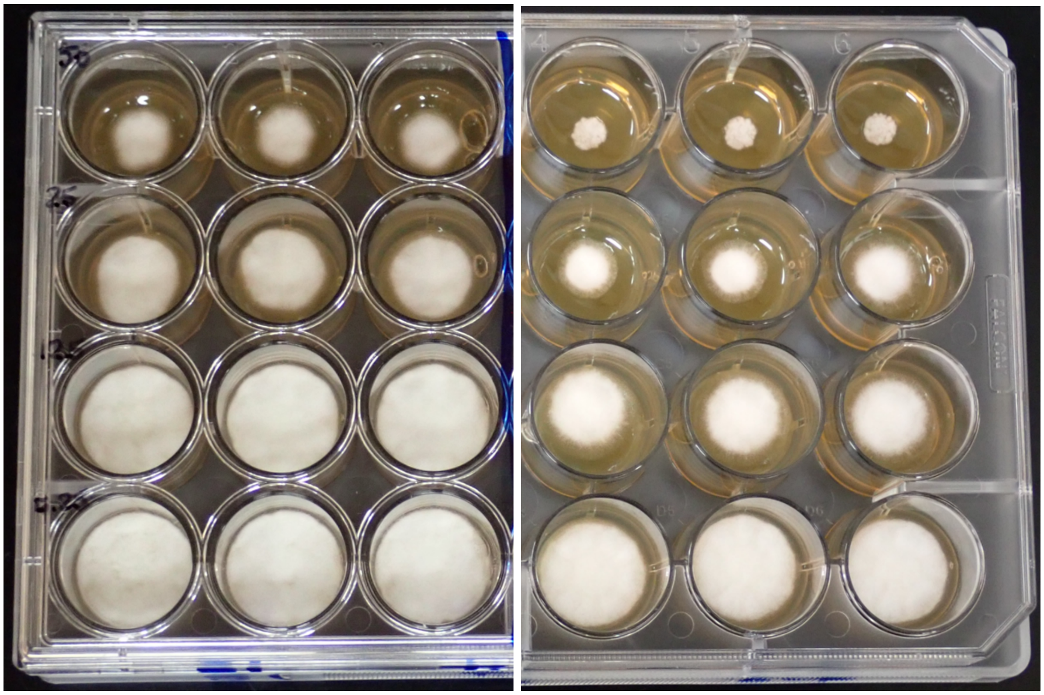

Figure S194. Compounds **6t** (left) and **6d** (right) at 50 (top row), 25, 12.5, and 6.25 (bottom row) μg/mL against *T. mentagrophytes*
